# Supplementary material for: A facile synthesis of α,β-unsaturated imines via palladium-catalyzed dehydrogenation
Source: Nat Commun. 2024 May 21;15:4329. doi: 10.1038/s41467-024-48737-9 (PMC11109338; doi:10.1038/s41467-024-48737-9)
Supplement: Supplementary file 1 — Supplementary Information [file 41467_2024_48737_MOESM1_ESM.pdf]

Supplementary Information

A Facile Synthesis of *α,β*-Unsaturated Imines via Palladium-Catalyzed Dehydrogenation

Chunyang Zhao,[1,#] Rongwan Gao,[2,#] Wenxuan Ma,[1] Miao Li,[1] Yifei Li,[1] Qian Zhang,[1] Wei Guan,*[,2] and Junkai Fu*[,1]

[1] Jilin Province Key Laboratory of Organic Functional Molecular Design & Synthesis and Institute of Functional Material Chemistry, Department of Chemistry, Northeast Normal University, Changchun 130024, China

[2] Institute of Functional Material Chemistry, Department of Chemistry, Northeast Normal University, Changchun 130024, China

[#] These authors contributed equally to this work

* Correspondence: guanw580@nenu.edu.cn (W. G.), fujk109@nenu.edu.cn (J. F.)

Table of Contents

Part 1: Supplementary methods --S2

Part 2: DFT calculations --S3

Part 3: Supplementary Notes ---S6

Part 3.1: Procedure and characteristic data for substrates **16s**, **18s**, **21s**, **25s**, **27s**, **30s**, **39s**, **43s**, **44s**, **D-4s**, and **65** ---S6

Part 3.2: Procedure and characteristic data for products **2-46** ----------------------S16

Part 3.3: Procedure and characteristic data for compound **47-61** ------------------S34

Part 3.4: Procedure and characteristic data for control experiments --------------S44

Part 3.5: NMR spectra ---S53

Part 4: Supplementary references ---S140

Part 1: Supplementary methods

Unless otherwise noted, all reactions were carried out under an argon atmosphere as well as anhydrous conditions. Commercial reagents were purchased from Adamas-beta®, Aldrich, TCI, Energy Chemical, Macklin, Bide, Leyan.com and J&K chemical, and were used as received. Anhydrous 1,4-dioxane, 1,2-dichloroethane (DCE), ethyl acetate (EtOAc), tetrahydrofuran (THF), dimethyl sulfoxide (DMSO), and acetone were purchased from Energy Chemical. The 2-butanol (2-BuOH) was distilled from sodium. Anhydrous acetonitrile (CH_3CN) was distilled from calcium hydride. Anhydrous toluene (Tol.) was distilled from sodium.

Reactions were monitored by Thin Layer Chromatography (TLC) on plates (GF254) supplied by Yantai Chemicals (China) visualized by UV or stained with ethanolic solution of phosphomolybdic acid and basic solution of $KMnO_4$. The products were purified by column chromatography over silica gel (300 - 400 size).

NMR spectra were recorded on a Brüker Advance 600 (^{1}H: 600 MHz, ^{13}C:150 MHz, ^{19}F: 565 MHz), Brüker Advance 500 (^{1}H: 500 MHz, ^{13}C: 125 MHz, ^{19}F: 470 MHz), and TMS was used as internal standard. The following abbreviations were used to explain the multiplicities: s = singlet, d = doublet, t = triplet, q = quartet, dd = doublet of doublets, m = multiplet, br = broad.

IR spectra were recorded on an IRPrestige-21 FTIR spectrometer. High resolution mass spectrometric (HRMS) data was recorded on Brüker Apex IV RTMS by using ESI method.

Part 2. DFT calculations

All DFT calculations were performed with the Gaussian 16 program.[1] Geometry optimizations were carried out using M06 hybrid functional. The LanL2DZ basis set was applied for Pd atom. The standard 6-31G(d,p) basis set was used for the other main-group elements. The vibrational frequency analysis was calculated at the same level as the geometry optimizations to guarantee the minimum without imaginary frequency but only one imaginary frequency for the saddle point. The intrinsic reaction coordinate (IRC)[2] was conducted to ensure the transition states actually connect with the correct reactants and products. In addition, the single-point energies of all studied systems were performed at the SMD(dioxane)/M06[6-311++G(d,p)/SDD(Pd)] level. The translational entropy was corrected with the method developed by Whitesides et.al.[3]

Correction of translational entropy in solution

We evaluated the electronic energy (E_{sol}) with zero-point energy correction in solution. For each species, the E_{sol} is defined through supplementary equation 1:

$$E_{sol} = E_{sol}^{pot} + E_{gas}^{v_0} \quad (1)$$

the E_{sol}^{pot} is the potential energy including non-electrostatic energy in solution and $E_{gas}^{v_0}$ denotes the zero-point vibrational energy in the gas phase. In a bimolecular process, such as the coordination of Pd(0) center to *N*-fluoro-sulfonamide or the dissociation of palladium(II) hydrofluoride, the entropy change must be taken into consideration because the entropy considerably decreases in the coordination and considerably increases in the dissociation. In such case, Gibbs energy (G_{sol}^{o}) need be computed as follows:

$$\begin{aligned} G_{sol}^{o} &= H_0 - T(S_r^o + S_v^o + S_t^o) \\ &= E^T + P\mathit{\Delta} V - T(S_r^o + S_v^o + S_t^o) \\ &= E_{sol} + E_{therm} - T(S_r^o + S_v^o + S_t^o) \quad (2) \end{aligned}$$

where $\mathit{\Delta} V$ is 0 in solution, E_{therm} is the thermal correction by translational, vibrational,

and rotational movements, and S_r^o, S_v^o, and S_t^o are rotational, vibrational, and translational entropies, respectively. In general, the Sackur-Tetrode equation is used to evaluate translational entropy S_t^o. In solution, however, the usual Sackur-Tetrode equation cannot be directly applied to the evaluation of S_t^o, because the translation movement is suppressed very much in solution.[4-6] In this context, the translational entropy was corrected with the method developed by Whitesides et al., where the rotational entropy was evaluated in a normal manner. Thermal correction and entropy contributions of vibration movements to the Gibbs energy were evaluated with the frequencies calculated at 343.15 K and 1 atm.

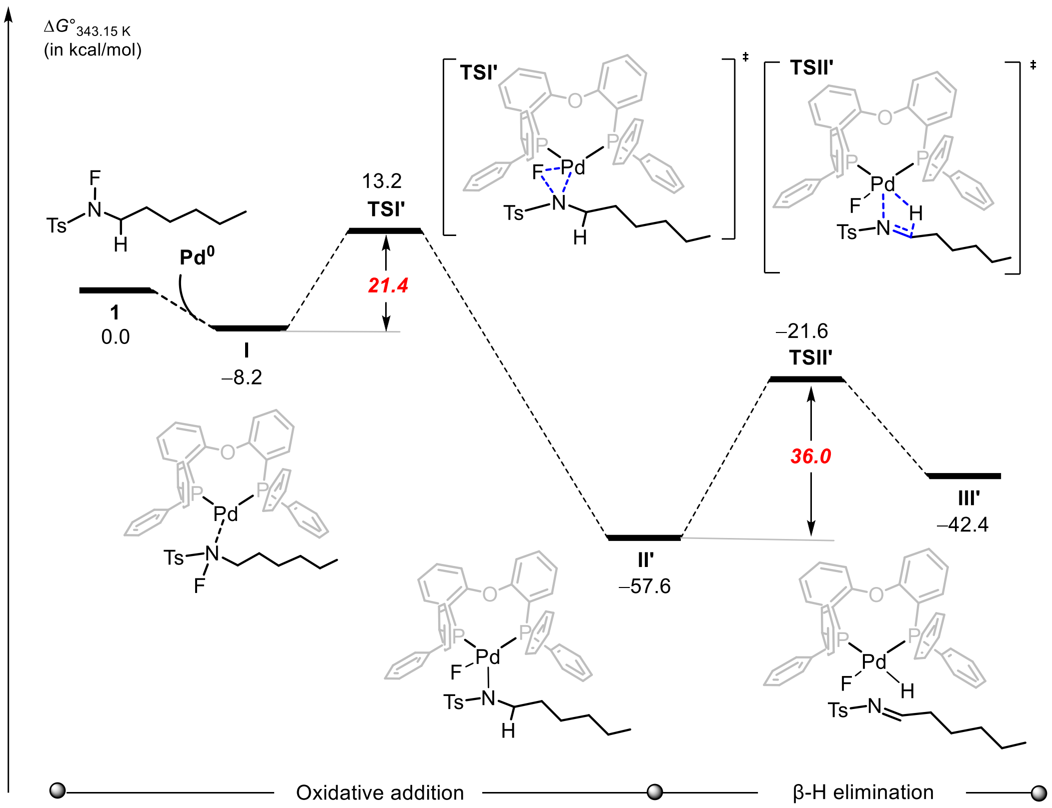

Supplementary Figure 1. Gibbs energy profiles of the stepwise oxidative addition followed by *β*-H elimination.

As shown in Supplementary Figure 1, the stepwise oxidative addition followed by *β*-H elimination requires the energy barriers of 21.4 and 36.0 kcal/mol, which is much larger than that of the concerted oxidative H–F elimination of **I** → **II** (Figure 6).

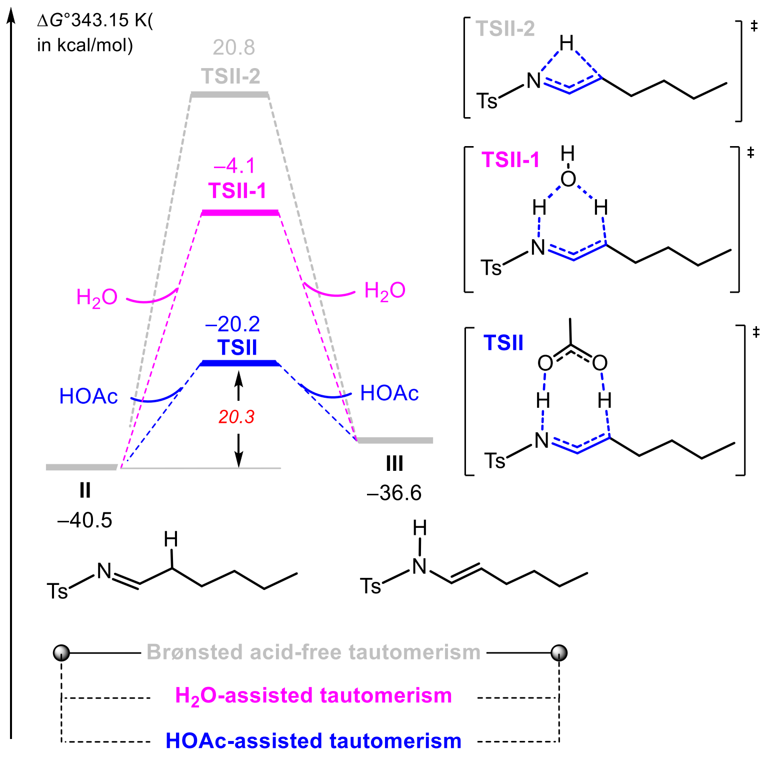

Supplementary Figure 2. Gibbs energy profiles of the Brønsted acid-free, H_2O-assisted and HOAc-assisted tautomerisms.

As shown in Supplementary Figure 2, the Brønsted acid-free and water-assisted tautomerisms are much less favorable than the HOAc-assisted tautomerism. With the assistance of additional HOAc, the imine intermediate **II** undergoes a proton transfer to isomerize into an enamine intermediate **III** though an eight-membered-ring transition state **TSII**. Such tautomerism requires a moderate energy barrier of 20.3 kcal/mol, which is much smaller than the Brønsted acid-free and water-assisted pathways ($\Delta G^{\circ\ddagger}$ = 61.3 and 36.4 kcal/mol).

Part 3: Supplementary Notes

Part 3.1: Procedure and characteristic data for substrates **16s, 18s, 21s, 25s, 27s, 30s, 39s, 43s, 44s, D-4s,** and **65**

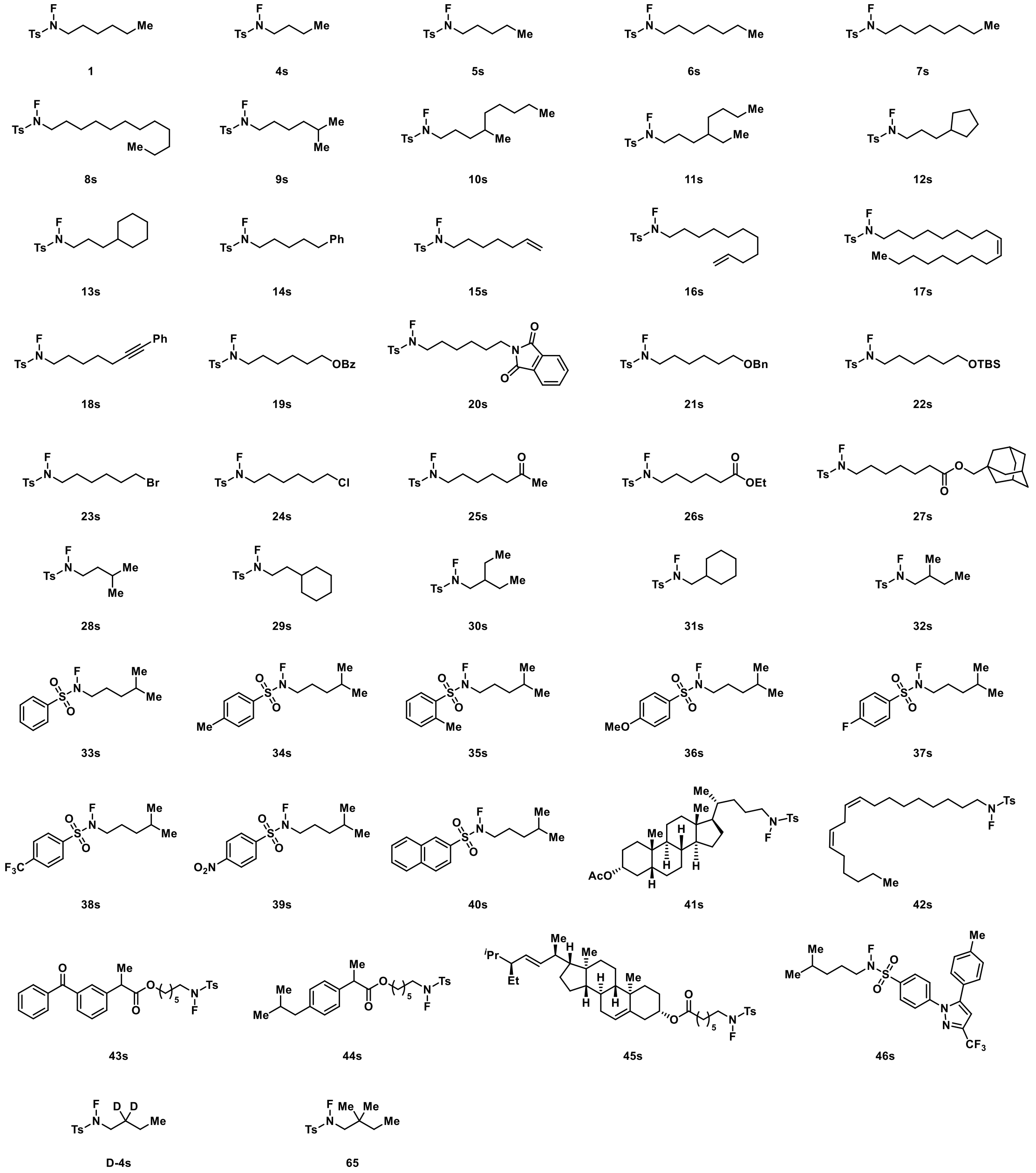

Supplementary Figure 3. The *N*-fluoroamides employed in this paper.

Substrates **1, 5s-12s, 15s, 19s, 23s, 29s, 33s-38s, 40s, 41s, 46s** are prepared according to ref. 7; Substrates **4s, 13s, 20s** are prepared according to ref. 8; Substrate **14s** is prepared according to ref. 9; Substrates **17s, 32s, 42s, 45s** are prepared according to ref. 10; Substrate **22s** is prepared according

to ref. 11; Substrate **24s** is prepared according to ref. 12; Substrate **26s** is prepared according to ref. 13; Substrate **28s** is prepared according to ref. 14; Substrate **31s** is prepared according to ref. 15.

Procedure A

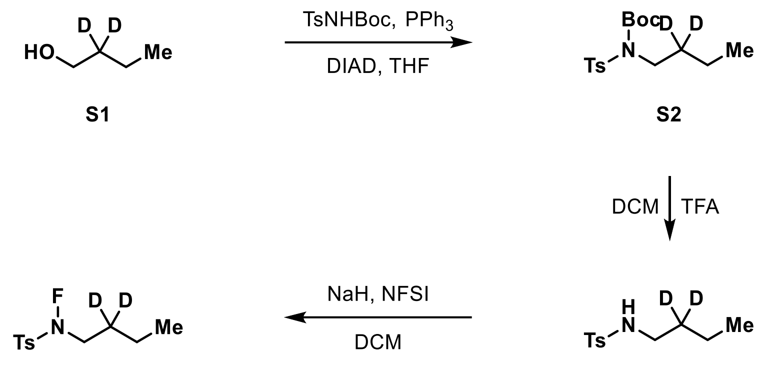

S1 **S2** **S3** **D-4s**

Synthesis of compound **S3**

Step 1: Triphenylphosphine (PPh_3, 5.77 g, 22.0 mmol, 1.1 equiv), alcohol **S1**[16] (1.52 g, 20.0 mmol, 1.0 equiv), and tert-butyl tosylcarbamate (5.97 g, 22.0 mmol, 1.1 equiv) were dissolved in anhydrous THF (40 mL). The mixture was stirred for 10 min at room temperature, and then cooled to 0 °C under nitrogen atmosphere. Diisopropyl azodicarboxylate (DIAD, 4.33 mL, 22.0 mmol, 1.1 equiv) was added dropwise at 0 °C. The reaction was stirred at room temperature for 12 h. Once completion, the reaction was quenched with cold water (40 mL), and the mixture was extracted with EtOAc (30 mL × 3). The combined organic layers were washed with brine, dried over anhydrous Na_2SO_4, and filtered. The solvent was removed under reduced pressure and the residue was washed through a short column chromatography (PE/EA = 1:1) to afford the crude compound **S2**, which was used in the next step without further purification.

Step 2: The crude compound **S2** was dissolved in DCM (15 mL). Trifluoroacetic acid (TFA, 3.0 mL) was added at room temperature. The solution was stirred for 3 h until the complete consumption of compound **S2** (monitored by TLC). Excess acid was quenched with saturated $NaHCO_3$ solution (~40 mL). The organic phase was collected and the aqueous solution was extracted with DCM (30 mL × 3). The combined organic fractions were dried over anhydrous $MgSO_4$, and filtered. The solvent was removed under reduced pressure and the residue was purified by a flash column chromatography (PE/EA = 5:1) afforded the desired product **S3** as colorless oil (2.98 g, 13.0 mmol, 65% from **S1**). **^{1}H NMR** (500 MHz, $CDCl_3$) δ 7.77 (d, J = 8.5 Hz, 2H), 7.30 (d, J = 8.5 Hz, 2H), 4.98 (t, J = 6.0 Hz, 1H), 2.90 (d, J = 6.0 Hz, 2H), 2.42 (s, 3H), 1.27 (q, J = 7.5 Hz, 2H), 0.83 (t, J = 7.5 Hz, 3H). **^{13}C NMR** (125 MHz, $CDCl_3$) δ 143.3, 137.1, 129.7, 127.2, 42.8, 30.8 (quin, J = 20.0 Hz), 21.6, 19.5, 13.5. **IR** ν_{max} (film): 3285 2960 2873 1599 1496 1457 1428 1324

1161 1094 815 665 569 551 cm^{-1}. **HRMS** (ESI) m/z calcd for $C_{11}H_{15}D_2NNaO_2S$ $[M+Na]^+$: 252.0998; found: 252.0987.

Synthesis of compound **D-4s**

A flame dried Schlenk equipped with a stirrer bar was charged with NaH (1.20 g, 30.0 mmol, 3.0 equiv, 60% in oil) and anhydrous DCM (10 mL). A solution of **S3** (2.29 g, 10.0 mmol, 1.0 equiv) in anhydrous DCM (10 mL) was slowly added at 0 °C. The mixture was allowed to stir for 30 min at room temperature under nitrogen atmosphere. Then N-Fluorobenzenesulfonimide (NFSI, 9.46 g, 30.0 mmol, 3.0 equiv) in anhydrous DCM (20 mL) was added dropwise to the mixture and the resulting slurry was stirred for another 12 h. The reaction was quenched by saturated $NaHCO_3$ solution (20 mL) at 0 °C, and then extracted with DCM (20 mL × 3). The combined organic layers were washed sequentially with hydrochloric acid (1.0 M) and brine, dried over anhydrous Na_2SO_4, and filtered. The solvent was removed under reduced pressure and the residue was purified by a flash column chromatography (PE/EA = 20:1) to afford the desired product **D-4s** as yellow oil (1.70 g, 6.9 mmol, 69%). **^{1}H NMR** (600 MHz, $CDCl_3$) δ 7.82 (d, J = 8.4 Hz, 2H), 7.36 (d, J = 8.4 Hz, 2H), 3.20 (d, J = 40.8 Hz, 2H), 2.48 (s, 3H), 1.41 (q, J = 7.8 Hz, 2H), 0.92 (t, J = 7.8 Hz, 3H). **^{13}C NMR** (150 MHz, $CDCl_3$) δ 146.3, 130.1, 130.0, 129.1, 53.4 (d, J = 12.0 Hz), 27.7 (quin, J = 19.5 Hz), 21.9, 19.7, 13.6. **^{19}F NMR** (565 MHz, $CDCl_3$) δ -49.97 (t, J = 41.2 Hz). **IR** ν_{max} (film): 2961 2875 1597 1493 1457 1431 1375 1175 814 664 569 550 cm^{-1}. **HRMS** (ESI) m/z calcd for $C_{11}H_{14}D_2FNNaO_2S$ $[M+Na]^+$: 270.0904; found: 270.0898.

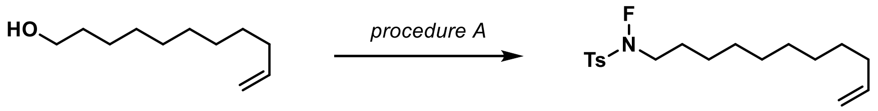

16s

Substrate **16s** was prepared in 52% overall yield as yellow solid following procedure A from commercially available undec-10-en-1-ol. **^{1}H NMR** (600 MHz, $CDCl_3$) δ 7.81 (d, J = 8.4 Hz, 2H), 7.40 (d, J = 8.4 Hz, 2H), 5.80 (ddt, J = 15.0, 10.8, 6.6 Hz, 1H), 5.00 - 4.91 (m, 2H), 3.20 (dt, J = 40.8, 7.2 Hz, 2H), 2.47 (s, 3H), 2.03 (dt, J = 15.0, 6.6 Hz, 2H), 1.72 - 1.67 (m, 2H), 1.41 - 1.34 (m, 4H), 1.30 - 1.27 (m, 8H). **^{13}C NMR** (150 MHz, $CDCl_3$) δ 146.2, 139.2, 130.0, 129.1, 114.2, 53.9 (d, J = 12.0 Hz), 33.8, 29.41, 29.39, 29.14, 29.12, 29.0, 26.6, 26.4, 21.8. **^{19}F NMR** (565 MHz, $CDCl_3$) δ -49.82 (t, J = 40.7 Hz). **IR** ν_{max} (film): 3075 2939 2859 1674 1643 1507 1489 1457 1378 1176 814 650 576 518 cm^{-1}. **HRMS** (ESI) m/z calcd for $C_{18}H_{28}FNNaO_2S$ $[M+Na]^+$: 364.1717; found: 364.1720.

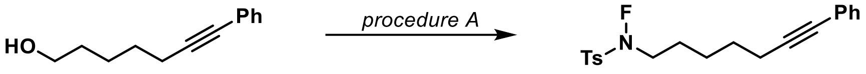

18s

Substrate **18s** was prepared in 52% overall yield as yellow solid following procedure A from 7-phenylhept-6-yn-1-ol[17]. **^{1}H NMR** (600 MHz, $CDCl_3$) δ 7.82 (d, J = 8.4 Hz, 2H), 7.39 - 7.37 (m, 4H), 7.28 - 7.26 (m, 3H), 3.24 (dt, J = 40.2, 7.2 Hz, 2H), 2.47 (s, 3H), 2.41 (t, J = 6.6 Hz, 2H), 1.80 - 1.75 (m, 2H), 1.64 - 1.56 (m, 4H). **^{13}C NMR** (150 MHz, $CDCl_3$) δ 146.3, 131.7, 130.09, 130.07, 129.2, 128.3, 127.7, 124.1, 89.8, 81.1, 53.7 (d, J = 12.0 Hz), 28.3, 26.1, 26.0, 21.9, 19.4. **^{19}F NMR** (565 MHz, $CDCl_3$) δ -49.65 (t, J = 40.7 Hz). **IR** ν_{max} (film): 2970 2879 2238 1507 1489 1442 1374 1175 814 650 578 502 cm^{-1}. **HRMS** (ESI) m/z calcd for $C_{20}H_{22}FNNaO_2S$ $[M+Na]^+$: 382.1247; found: 382.1257.

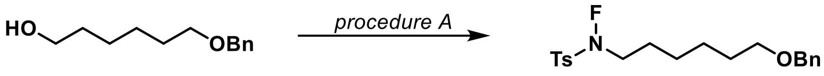

21s

Substrate **21s** was prepared in 46% overall yield as white solid following procedure A from 6-(benzyloxy)hexan-1-ol[18]. **^{1}H NMR** (600 MHz, $CDCl_3$) δ 7.80 (d, J = 7.8 Hz, 2H), 7.37 (d, J = 7.8 Hz, 2H), 7.32 - 7.31 (m, 4H), 7.27 - 7.23 (m, 1H), 4.47 (s, 2H), 3.45 (t, J = 6.6 Hz, 2H), 3.18 (dt, J = 40.8, 7.2 Hz, 2H), 2.45 (s, 3H), 1.72 - 1.67 (m, 2H), 1.62 - 1.58 (m, 2H), 1.41 - 1.38 (m, 4H). **^{13}C NMR** (150 MHz, $CDCl_3$) δ 146.2, 138.7, 130.0, 129.9, 128.9, 128.4, 127.6, 127.5, 72.9, 70.2, 53.8 (d, J = 12.0 Hz), 29.6, 26.4, 26.3, 25.8, 21.8. **^{19}F NMR** (565 MHz, $CDCl_3$) δ -49.83 (t, J = 40.7 Hz). **IR** ν_{max} (film): 2941 2862 1596 1495 1453 1375 1174 1091 815 650 576 550 cm^{-1}. **HRMS** (ESI) m/z calcd for $C_{20}H_{26}FNNaO_3S$ $[M+Na]^+$: 402.1510; found: 402.1510.

Procedure B

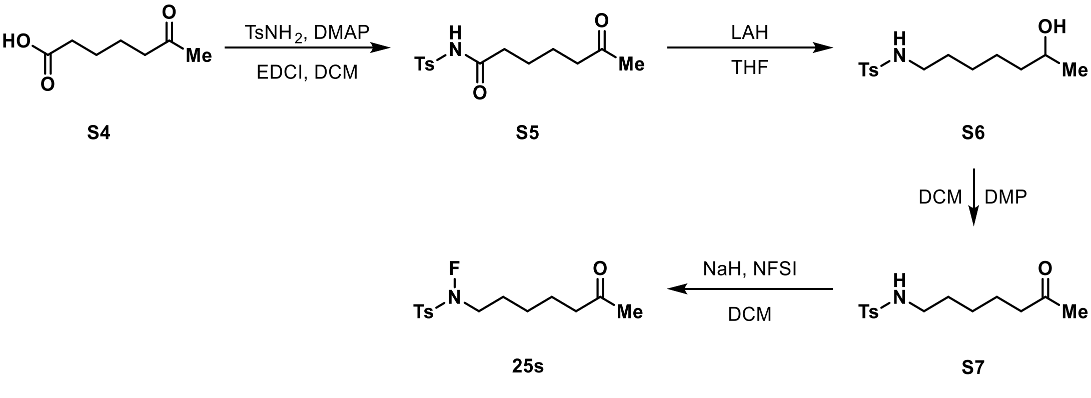

Synthesis of compound **S5**

To a solution of $TsNH_2$ (5.13 g, 30.0 mmol, 1.0 equiv), 1-ethyl-3-(3-dimethylaminopropyl) carbodiimide hydrochloride (EDCI, 6.90 g, 36.0 mmol, 1.2 equiv), and 4-dimethylaminopyridine (DMAP, 0.37 g, 3.0 mmol, 0.10 equiv) in DCM (100 mL) was added commercially available 6-oxoheptanoic acid **S4** (4.32 g, 30.0 mmol, 1.0 equiv) at room temperature under nitrogen atmosphere. The reaction mixture was stirred at room temperature for 12 h. The reaction was quenched by H_2O (20 mL) at 0 °C, and then extracted with DCM (20 mL × 3). The organic phase was dried over anhydrous $MgSO_4$, and filtered. The solvent was removed under reduced pressure and the residue was washed through a short column chromatography (PE/EA = 1:1) to afford the crude compound **S5** (about 25.0 mmol, 83% yield, determinate by ^{1}H NMR), which was used in the next step without further purification.

Synthesis of compound **S6**

To a solution of crude **S5** (7.43 g, 25.0 mmol, 1.0 equiv) in anhydrous THF (50 mL) was added $LiAlH_4$ (LAH, 2.37 g, 62.5 mmol, 2.5 equiv) in 3 portions at -10 °C. The reaction mixture was stirred at room temperature until completion (monitored by TLC), and then cooled to -10 °C. H_2O (3.0 mL) was added dropwise. After 10 min, NaOH (10 wt% in H_2O, 3.0 mL) was added dropwise. The mixture was stirred for an additional 10 min, and then extracted with EtOAc (20 mL × 3). The organic phase was dried over anhydrous $MgSO_4$, and filtered. The solvent was removed under reduced pressure and the residue was purified by a flash column chromatography (PE/EA = 10:1) afforded the desired product **S6** as yellow oil (5.28 g, 18.5 mmol, 74%). **^{1}H NMR** (600 MHz, $CDCl_3$) δ 7.75 (d, J = 7.8 Hz, 2H), 7.29 (d, J = 7.8 Hz, 2H), 5.28 - 5.19 (m, 1H), 3.75 - 3.72 (m, 1H), 2.92 - 2.88 (m, 2H), 2.42 (s, 3H), 2.06 - 1.95 (m, 1H), 1.48 - 1.43 (m, 2H), 1.39 - 1.23 (m, 6H), 1.15 - 1.13 (m, 3H). **^{13}C NMR** (150 MHz, $CDCl_3$) δ 143.3, 137.1, 129.7, 127.1, 67.8, 43.1, 39.0, 29.4, 26.4, 25.1, 23.5, 21.5. **IR** ν_{max} (film): 3284 2966 2867 1564 1496 1456 1375 1154 1096 815 661 577 550 cm^{-1}. **HRMS** (ESI) m/z calcd for $C_{14}H_{23}NNaO_3S$ $[M+Na]^+$: 308.1291; found: 308.1289.

Synthesis of compound **S7**

To a solution of **S6** (5.28 g, 18.5 mmol, 1.0 equiv) in anhydrous DCM (70 mL) was added Dess-Martin periodinane (DMP, 15.69 g, 37.0 mmol, 2.0 equiv) in 5 portions at 0 °C. The reaction mixture was stirred at room temperature until completion (monitored by TLC), and then cooled to 0 °C. H_2O (10 mL) was added dropwise. After 10 min, NaOH (10 wt% in H_2O, 15 mL) was added dropwise. The mixture was stirred for an additional 10 min, and then extracted with EtOAc (20 mL

× 3). The organic phase was dried over anhydrous $MgSO_4$, and filtered. The solvent was removed under reduced pressure and the residue was purified by a flash column chromatography (PE/EA = 10:1) afforded the desired product **S7** as yellow oil (3.72 g, 13.1 mmol, 71%). **^{1}H NMR** (600 MHz, $CDCl_3$) δ 7.75 (d, J = 7.8 Hz, 2H), 7.30 (d, J = 7.8 Hz, 2H), 5.23 (t, J = 6.0 Hz, 1H), 2.90 (dt, J = 13.2, 6.0 Hz, 2H), 2.42 (s, 3H), 2.37 (t, J = 7.2 Hz, 2H), 2.10 (s, 3H), 1.51 - 1.43 (m, 4H), 1.28 - 1.23 (m, 2H). **^{13}C NMR** (150 MHz, $CDCl_3$) δ 209.1, 143.2, 137.1, 129.6, 127.0, 43.3, 42.9, 29.9, 29.2, 25.9, 23.0, 21.5. **IR** ν_{max} (film): 3284 2940 2867 1710 1598 1495 1424 1360 1159 1094 816 663 572 551 cm^{-1}. **HRMS** (ESI) m/z calcd for $C_{14}H_{21}NNaO_3S$ $[M+Na]^+$: 306.1134; found: 306.1142.

Synthesis of compound **25s**

A flame dried Schlenk equipped with a stirrer bar was charged with NaH (1.20 g, 30.0 mmol, 3.0 equiv, 60% in oil) and anhydrous DCM (10 mL). A solution of **S7** (2.83 g, 10.0 mmol, 1.0 equiv) in anhydrous DCM (10 mL) was slowly added at 0 °C. The mixture was allowed to stir for 30 min at room temperature under nitrogen atmosphere. Then NFSI (9.46 g, 30.0 mmol, 3.0 equiv) in anhydrous DCM (20 mL) was added dropwise to the mixture and the resulting slurry was stirred for another 12 h. The reaction was quenched by saturated $NaHCO_3$ solution (20 mL) at 0 °C, and then extracted with DCM (20 mL × 3). The combined organic layers were washed sequentially with hydrochloric acid (1.0 M) and brine, dried over anhydrous Na_2SO_4, and filtered. The solvent was removed under reduced pressure, and the residue was purified by a flash column chromatography (PE/EA = 15:1) to afford the desired product **25s** as yellow oil (2.11 g, 7.0 mmol, 70%). **^{1}H NMR** (600 MHz, $CDCl_3$) δ 7.81 (d, J = 7.8 Hz, 2H), 7.41 (d, J = 7.8 Hz, 2H), 3.20 (dt, J = 40.2, 7.2 Hz, 2H), 2.48 (s, 3H), 2.43 (t, J = 7.8 Hz, 2H), 2.12 (s, 3H), 1.74 - 1.69 (m, 2H), 1.61 - 1.56 (m, 2H), 1.43 - 1.38 (m, 2H). **^{13}C NMR** (150 MHz, $CDCl_3$) δ 208.7, 146.4, 130.1, 129.0, 53.6 (d, J = 12.0 Hz), 43.5, 30.0, 26.3, 26.2, 23.3, 21.9. **^{19}F NMR** (565 MHz, $CDCl_3$) δ -49.69 (t, J = 40.7 Hz). **IR** ν_{max} (film): 2951 2839 1716 1596 1507 1457 1375 1174 816 650 575 549 cm^{-1}. **HRMS** (ESI) m/z calcd for $C_{14}H_{20}FNNaO_3S$ $[M+Na]^+$: 324.1040; found: 324.1048.

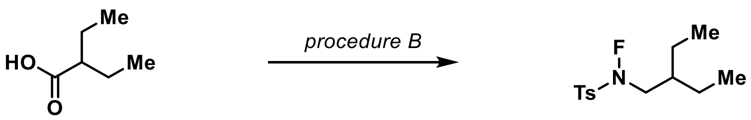

30s

Substrate **30s** was prepared in 41% overall yield as yellow oil following procedure B from commercially available 2-ethylbutanoic acid. **^{1}H NMR** (600 MHz, $CDCl_3$) δ 7.82 (d, J = 8.4 Hz, 2H), 7.40 (d, J = 8.4 Hz, 2H), 3.11 (dd, J = 42.6, 6.6 Hz, 2H), 2.48 (s, 3H), 1.68 - 1.64 (m, 1H),

1.46 - 1.40 (m, 4H), 0.86 (t, *J* = 7.2 Hz, 6H). ^{13}**C NMR** (150 MHz, $CDCl_3$) δ 146.2, 130.03, 130.02, 129.4, 56.6 (d, *J* = 12.0 Hz), 38.4, 23.7, 21.9, 10.6. ^{19}**F NMR** (565 MHz, $CDCl_3$) δ -46.45 (t, *J* = 42.4 Hz). **IR** ν_{max} (film): 2965 2878 1597 1494 1459 1377 1176 815 653 577 552 cm^{-1}. **HRMS** (ESI) m/z calcd for $C_{13}H_{20}FNNaO_2S$ $[M+Na]^+$: 296.1091; found: 296.1082.

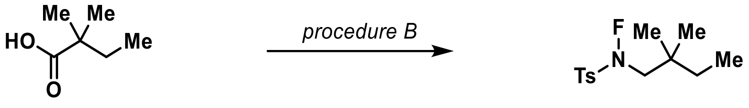

65

Substrate **65** was prepared in 43% overall yield as yellow oil following procedure B from commercially available 2,2-dimethylbutanoic acid. ^{1}**H NMR** (600 MHz, $CDCl_3$) δ 7.82 (d, *J* = 7.8 Hz, 2H), 7.40 (d, *J* = 7.8 Hz, 2H), 3.01 (d, *J* = 44.4 Hz, 2H), 2.48 (s, 3H), 1.37 (q, *J* = 7.2 Hz, 2H), 0.94 (s, 6H), 0.83 (t, *J* = 7.2 Hz, 3H). ^{13}**C NMR** (150 MHz, $CDCl_3$) δ 146.2, 130.1, 130.0, 129.8, 62.9 (d, *J* = 9.0 Hz), 34.5, 32.7, 25.2, 21.9, 8.2. ^{19}**F NMR** (565 MHz, $CDCl_3$) δ -36.36 (t, *J* = 44.6 Hz). **IR** ν_{max} (film): 2966 2877 1597 1494 1464 1376 1176 815 653 577 552 cm^{-1}. **HRMS** (ESI) m/z calcd for $C_{13}H_{20}FNNaO_2S$ $[M+Na]^+$: 296.1091; found: 296.1095.

Procedure C

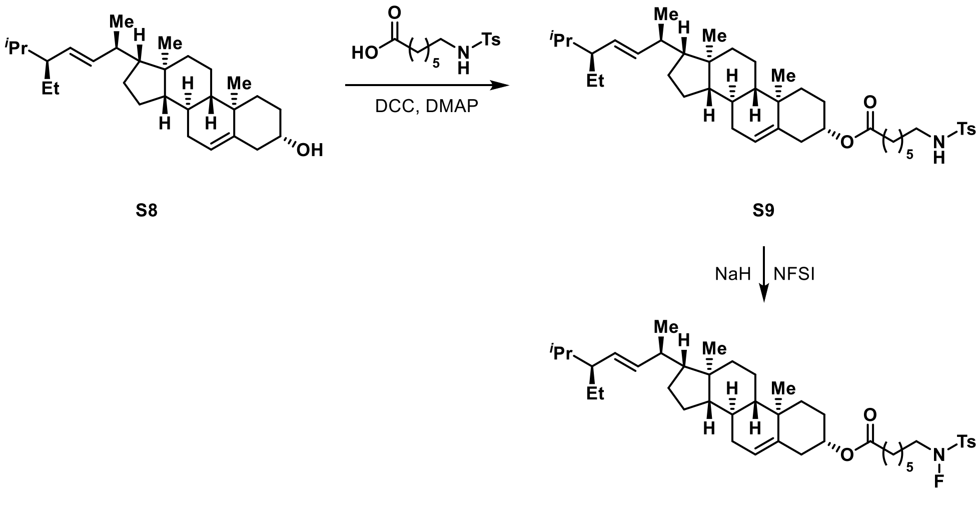

45s

Synthesis of compound **S9**

To a solution of 7-((4-methylphenyl)sulfonamido)heptanoic acid[10] (5.98 g, 20.0 mmol, 1.0 equiv) in DCM (100 mL) at 25 °C was added stigmasterol **S8** (9.07 g, 22.0 mmol, 1.1 equiv), followed by DMAP (0.24 g, 2.0 mmol, 0.10 equiv) and dicyclohexylcarbodiimide (DCC, 4.54 g, 22.0 mmol, 1.1 equiv). The reaction mixture was stirred at 25 °C for 12 h under nitrogen

atmosphere. Upon completion, the reaction mixture was filtered through celite and the filter cake was washed with DCM (25 mL × 3). The resultant filtrate was removed under reduced pressure, and the residue was purified by a flash column chromatography (PE/EA = 5:1) to afford the desired product **S9**[10] as white solid (9.71 g, 14.0 mmol, 70%).

Synthesis of compound **45s**

A flame dried Schlenk equipped with a stirrer bar was charged with NaH (1.68 g, 42.0 mmol, 3.0 equiv, 60% in oil) and anhydrous DCM (15 mL). A solution of **S9** (9.71 g, 14.0 mmol, 1.0 equiv) in anhydrous DCM (15 mL) was slowly added at 0 °C. The mixture was allowed to stir for 30 min at room temperature under nitrogen atmosphere. Then NFSI (13.24 g, 42.0 mmol, 3.0 equiv) in anhydrous DCM (30 mL) was added dropwise to the mixture and the resulting slurry was stirred for another 12 h. The reaction was quenched by saturated $NaHCO_3$ solution (30 mL) at 0 °C, and then extracted with DCM (20 mL × 3). The combined organic layers were washed sequentially with hydrochloric acid (1.0 M) and brine, dried over anhydrous Na_2SO_4, and filtered. The solvent was removed under reduced pressure, and the residue was purified by a flash column chromatography (PE/EA = 10:1) to afford the desired product **45s**[10] as yellow solid (5.38 g, 7.6 mmol, 54%).

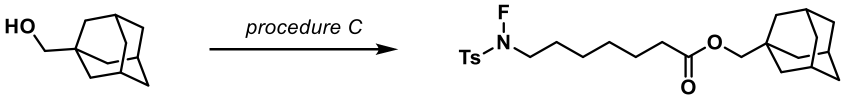

27s

Substrate **27s** was prepared in 60% overall yield as yellow oil following procedure C from commercially available 1-adamantanemethanol. **^{1}H NMR** (600 MHz, $CDCl_3$) δ 7.82 (d, J = 8.4 Hz, 2H), 7.41 (d, J = 8.4 Hz, 2H), 3.66 (s, 2H), 3.20 (dt, J = 40.8, 7.2 Hz, 2H), 2.48 (s, 3H), 2.31 (t, J = 7.2 Hz, 2H), 1.98 - 1.97 (m, 3H), 1.74 - 1.70 (m, 5H), 1.66 - 1.62 (m, 5H), 1.53 - 1.52 (m, 6H), 1.45 - 1.40 (m, 2H), 1.38 - 1.34 (m, 2H). **^{13}C NMR** (150 MHz, $CDCl_3$) δ 173.9, 146.3, 130.0, 129.0, 74.0, 53.7 (d, J = 12.0 Hz), 39.4, 37.1, 34.3, 33.2, 28.7, 28.1, 26.3, 26.2, 24.9, 21.8. **^{19}F NMR** (565 MHz, $CDCl_3$) δ -49.86 (t, J = 40.7 Hz). **IR** ν_{max} (film): 2905 2849 1732 1597 1454 1378 1261 1176 815 650 576 551 cm^{-1}. **HRMS** (ESI) m/z calcd for $C_{25}H_{36}FNNaO_4S$ $[M+Na]^+$: 488.2241; found: 488.2252.

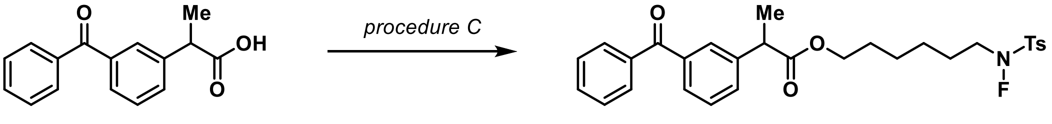

43s

Substrate **43s** was prepared in 65% overall yield as yellow oil following procedure C from *N*-(6-hydroxyhexyl)-4-methylbenzenesulfonamide[13] and commercially available ketoprofen. **^{1}H NMR** (600 MHz, $CDCl_3$) δ 7.81 - 7.78 (m, 4H), 7.76 (s, 1H), 7.66 (d, *J* = 7.8 Hz, 1H), 7.59 (t, *J* = 7.2 Hz, 1H), 7.54 (d, *J* = 7.8 Hz, 1H), 7.48 (t, *J* = 7.8 Hz, 2H), 7.43 (t, *J* = 7.8 Hz, 1H), 7.40 (d, *J* = 7.8 Hz, 2H), 4.06 (t, *J* = 6.6 Hz, 2H), 3.79 (q, *J* = 7.2 Hz, 1H), 3.17 (dt, *J* = 40.8, 7.2 Hz, 2H), 2.47 (s, 3H), 1.67 - 1.63 (m, 2H), 1.60 - 1.55 (m, 2H), 1.53 (d, *J* = 7.2 Hz, 3H), 1.39 - 1.34 (m, 2H), 1.30 - 1.25 (m, 2H). **^{13}C NMR** (150 MHz, $CDCl_3$) δ 196.5, 174.1, 146.3, 141.0, 137.9, 137.6, 132.6, 131.6, 130.1, 130.01, 129.99, 129.2, 129.0, 128.9, 128.6, 128.4, 64.8, 53.6 (d, *J* = 12.0 Hz), 45.5, 28.4, 26.20, 26.17, 25.4, 21.8, 18.5. **^{19}F NMR** (565 MHz, $CDCl_3$) δ -49.83 (t, *J* = 40.7 Hz). **IR** ν_{max} (film): 2945 2867 1732 1660 1597 1448 1375 1282 1175 817 664 576 551 cm^{-1}. **HRMS** (ESI) m/z calcd for $C_{29}H_{32}FNNaO_5S$ $[M+Na]^+$: 548.1877; found: 548.1883

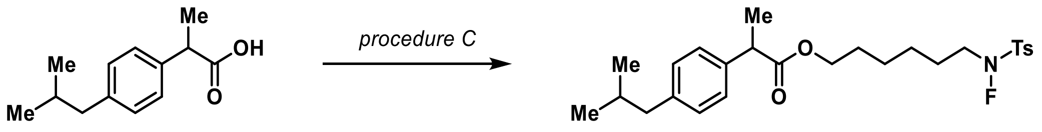

44s

Substrate **44s** was prepared in 65% overall yield as yellow oil following procedure C from *N*-(6-hydroxyhexyl)-4-methylbenzenesulfonamide[19] and commercially available lbuprofen. **^{1}H NMR** (600 MHz, $CDCl_3$) δ 7.82 (d, *J* = 8.4 Hz, 2H), 7.40 (d, *J* = 8.4 Hz, 2H), 7.18 (d, *J* = 8.4 Hz, 2H), 7.08 (d, *J* = 8.4 Hz, 2H), 4.07 - 4.01 (m, 2H), 3,67 (q, *J* = 7.2 Hz, 1H), 3.17 (dt, *J* = 40.8, 7.2 Hz, 2H), 2.48 (s, 3H), 2.43 (d, *J* = 7.2 Hz, 2H), 1.87 - 1.80 (m, 1H), 1.67 - 1.62 (m, 2H), 1.58 - 1.54 (m, 2H), 1.47 (d, *J* = 7.2 Hz, 3H), 1.38 - 1.33 (m, 2H), 1.27 - 1.22 (m, 2H), 0.88 (d, *J* = 7.2 Hz, 6H). **^{13}C NMR** (150 MHz, $CDCl_3$) δ 174.9, 146.3, 140.6, 138.0, 130.07, 130.05, 129.4, 129.1, 127.3, 64.5, 53.6 (d, *J* = 12.0 Hz), 45.3, 45.1, 30.3, 28.5, 26.29, 26.25, 25.5, 22.5, 21.9, 18.5. **^{19}F NMR** (565 MHz, $CDCl_3$) δ -49.81 (t, *J* = 40.7 Hz). **IR** ν_{max} (film): 2954 2869 1732 1597 1457 1376 1260 1175 815 650 576 550 cm^{-1}. **HRMS** (ESI) m/z calcd for $C_{26}H_{36}FNNaO_4S$ $[M+Na]^+$: 500.2241; found: 500.2258

Procedure D

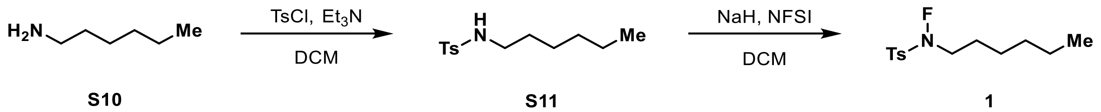

S10 S11 1

Synthesis of compound **S11**

The commercially available hexan-1-amine **S10** (1.01 g, 10.0 mmol, 1.0 equiv) was dissolved in anhydrous DCM (20 mL). Triethylamine (2.08 mL, 15.0 mmol, 1.5 equiv) and 4-toluolsulfonyl chloride (2.10 g, 11.0 mmol, 1.1 equiv) were added at 0 °C. The solution was stirred overnight at 25 °C. H_2O (20 mL) was added and the aqueous layer was extracted with DCM (20 mL × 3). The combined organic layers were dried over anhydrous Na_2SO_4, and filtered. The solvent was removed under reduced pressure and the residue was purified by a flash column chromatography (PE/EA = 10:1) to afford the desired product **S11**[20] as yellow solid (2.30 g, 9.0 mmol, 90%).

Synthesis of compound **1**

A flame dried Schlenk equipped with a stirrer bar was charged with NaH (1.08 g, 27.0 mmol, 3.0 equiv, 60% in oil) and anhydrous DCM (10 mL). A solution of **S11** (2.30 g, 9.0 mmol, 1.0 equiv) in anhydrous DCM (10 mL) was slowly added at 0 °C. The mixture was allowed to stir for 30 min at room temperature under nitrogen atmosphere. Then NFSI (8.51 g, 27.0 mmol, 3.0 equiv) in anhydrous DCM (20 mL) was added dropwise to the mixture and the resulting slurry was stirred for another 12 h. The reaction was quenched by saturated $NaHCO_3$ solution (20 mL) at 0 °C, and then extracted with DCM (20 mL × 3). The combined organic layers were washed sequentially with hydrochloric acid (1.0 M) and brine, dried over anhydrous Na_2SO_4, and filtered. The solvent was removed under reduced pressure, and the residue was purified by a flash column chromatography (PE/EA = 20:1) to afford the product **1**[7] as yellow oil (2.04 g, 7.5 mmol, 83%).

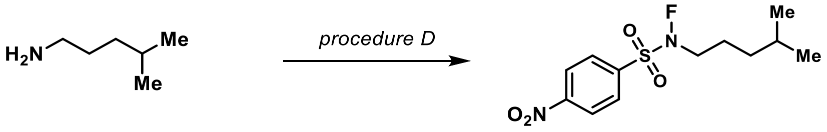

39s

Substrate **39s** was prepared in 70% overall yield as yellow oil following procedure D with 4-methylpentan-1-amine[21] and commercially available 4-nitrobenzenesulfonyl chloride. **^{1}H NMR** (600 MHz, $CDCl_3$) δ 8.45 (d, J = 9.0 Hz, 2H), 8.16 (d, J = 9.0 Hz, 2H), 3.30 (dt, J = 40.2, 7.2 Hz, 2H), 1.77 - 1.72 (m, 2H), 1.59 - 1.56 (m, 1H), 1.31 - 1.27 (m, 2H), 0.89 (d, J = 6.6 Hz, 6H). **^{13}C NMR** (150 MHz, $CDCl_3$) δ 151.6, 138.4, 131.4, 124.5, 53.7 (d, J = 13.5 Hz), 35.8, 27.8, 24.3, 22.5. **^{19}F NMR** (565 MHz, $CDCl_3$) δ -49.25 (t, J = 39.6 Hz). **IR** ν_{max} (film): 2961 2876 1597 1495 1463 1375 1174 816 650 576 551 cm^{-1}. **HRMS** (ESI) m/z calcd for $C_{12}H_{17}FN_2NaO_4S$ $[M+Na]^+$: 327.0785; found: 327.0774.

Part 3.2: Procedure and characteristic data for products **2-46**

The general procedure A (for products 2, 4-17, 19-29, 33-46):

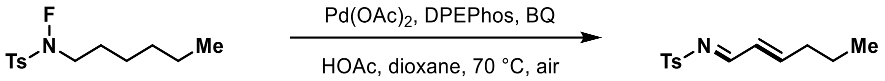

1 **2**

To a dry Schlenk flask were added **1** (54.6 mg, 0.20 mmol, 1.0 equiv), $Pd(OAc)_2$ (4.5 mg, 0.02 mmol, 0.10 equiv), bis[2-(diphenylphosphino)phenyl]ether (DPEPhos, 11.8 mg, 0.022 mmol, 0.11 equiv), 1,4-benzoquinone (BQ, 21.6 mg, 0.20 mmol, 1.0 equiv), anhydrous dioxane (1.0 mL), and HOAc (11 μL, 0.20 mmol, 1.0 equiv). The mixture was stirred at 70 °C (oil bath) for 15 min under air. Once completion, the reaction was cooled to room temperature. The reaction mixture was filtered by celite, and the filtrate was concentrated *in vacuo*. Further purification by a flash column chromatography using eluents (PE/EA = 20:1) afforded the desired product **2** as colourless oil (42.2 mg, 0.17 mmol, 84%, *E/Z* > 20:1). **^{1}H NMR** (500 MHz, acetone-d_6) δ 8.62 (d, *J* = 9.5 Hz, 1H), 7.79 (d, *J* = 8.5 Hz, 2H), 7.43 (d, *J* = 8.5 Hz, 2H), 7.13 (dt, *J* = 15.5, 7.0 Hz, 1H), 6.36 (dd, *J* = 15.5, 9.5 Hz, 1H), 2.43 (s, 3H), 2.35 (dt, *J* = 15.5, 7.0 Hz, 2H), 1.57 - 1.49 (m, 2H), 0.93 (t, *J* = 7.5 Hz, 3H). **^{13}C NMR** (125 MHz, acetone-d_6) δ 172.5, 161.9, 145.2, 136.9, 130.6, 129.1, 128.6, 36.0, 21.8, 21.5, 13.9. **IR** ν_{max} (film): 2960 2932 1636 1586 1302 1160 1090 905 815 778 705 676 536 cm^{-1}. **HRMS** (ESI) m/z calcd for $C_{13}H_{17}NNaO_2S$ $[M+Na]^+$: 274.0872; found: 274.0865.

Precaution: It would be better to finish the purification by flash column chromatography within one hour to avoid the product decomposition.

Lagre-scale synthesis of 2:

To a dry Schlenk flask were added **1** (1.37 g, 5.0 mmol, 1.0 equiv), $Pd(OAc)_2$ (112 mg, 0.50 mmol, 0.10 equiv), DPEPhos (296 mg, 0.55 mmol, 0.11 equiv), BQ (540 mg, 5.0 mmol, 1.0 equiv), anhydrous dioxane (25 mL), and HOAc (286 μL, 5.0 mmol, 1.0 equiv). The mixture was stirred at 70 °C (oil bath) for 15 min under air. Once completion, the reaction was cooled to room temperature. The reaction mixture was filtered by celite, and the filtrate was concentrated *in vacuo*. Further purification by a flash column chromatography using eluents (PE/EA = 20:1) afforded the desired product **2** as colourless oil (929 mg, 3.7 mmol, 74%, *E/Z* > 20:1).

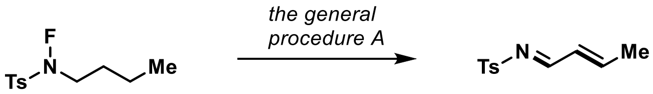

4s **4**

Product **4** was prepared by the general procedure A. Purification using column chromatography (PE/EA = 20:1) afforded **4** as yellow oil (33.5 mg, 0.15 mmol, 75%, *E/Z* > 20:1). **^{1}H NMR** (600 MHz, acetone-d_6) δ 8.61 (d, J = 9.6 Hz, 1H), 7.78 (d, J = 8.4 Hz, 2H), 7.44 (d, J = 8.4 Hz, 2H), 7.13 (dq, J = 15.6, 6.6 Hz, 1H), 6.38 (dd, J = 15.6, 9.6 Hz, 1H), 2.43 (s, 3H), 2.04 (d, J = 6.6 Hz, 3H). **^{13}C NMR** (150 MHz, acetone-d_6) δ 172.4, 157.4, 145.3, 137.0, 130.6, 130.4, 128.6, 21.5, 19.6. **IR** ν_{max} (film): 2965 2923 1646 1534 1301 1157 1098 905 816 704 670 535 cm^{-1}. **HRMS** (ESI) m/z calcd for $C_{11}H_{13}NNaO_2S$ [M+Na]$^+$: 246.0559; found: 246.0563.

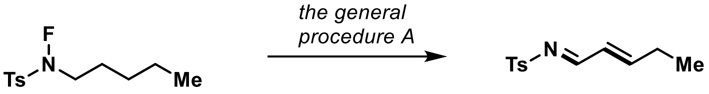

5s **5**

Product **5** was prepared by the general procedure A. Purification using column chromatography (PE/EA = 20:1) afforded **5** as yellow oil (41.7 mg, 0.18 mmol, 88%, *E/Z* > 20:1). **^{1}H NMR** (600 MHz, acetone-d_6) δ 8.62 (d, J = 9.6 Hz, 1H), 7.78 (d, J = 8.4 Hz, 2H), 7.44 (d, J = 8.4 Hz, 2H), 7.17 (dt, J = 15.6, 6.0 Hz, 1H), 6.35 (dd, J = 15.6, 9.6 Hz, 1H), 2.43 (s, 3H), 2.41 - 2.38 (m, 2H), 1.09 (t, J = 7.2 Hz, 3H). **^{13}C NMR** (150 MHz, acetone-d_6) δ 172.6, 163.3, 145.3, 137.0, 130.6, 128.6, 128.1, 27.2, 21.5, 12.2. **IR** ν_{max} (film): 2959 2932 1646 1529 1300 1160 1095 905 816 706 671 531 cm^{-1}. **HRMS** (ESI) m/z calcd for $C_{12}H_{15}NNaO_2S$ [M+Na]$^+$: 260.0716; found: 260.0711.

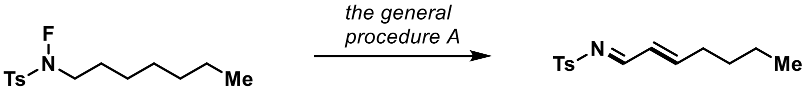

6s **6**

Product **6** was prepared by the general procedure A. Purification using column chromatography (PE/EA = 20:1) afforded **6** as yellow oil (44.5 mg, 0.17 mmol, 84%, *E/Z* > 20:1). **^{1}H NMR** (600 MHz, acetone-d_6) δ 8.62 (d, J = 9.6 Hz, 1H), 7.78 (d, J = 8.4 Hz, 2H), 7.43 (d, J = 8.4 Hz, 2H), 7.13 (dt, J = 15.0, 7.2 Hz, 1H), 6.36 (dd, J = 15.0, 9.6 Hz, 1H), 2.43 (s, 3H), 2.38 (dt, J = 15.0, 7.2 Hz, 2H), 1.52 - 1.47 (m, 2H), 1.39 - 1.32 (m, 2H), 0.90 (t, J = 7.2 Hz, 3H). **^{13}C NMR** (150 MHz, acetone-d_6) δ 172.5, 162.1, 145.2, 137.0, 130.6, 129.0, 128.6, 33.8, 30.7, 22.8, 21.5, 14.0. **IR** ν_{max} (film): 2961 2926 1647 1529 1301 1161 1098 905 816 704 670 535 cm^{-1}. **HRMS** (ESI) m/z calcd for $C_{14}H_{19}NNaO_2S$ [M+Na]$^+$: 288.1029; found: 288.1023.

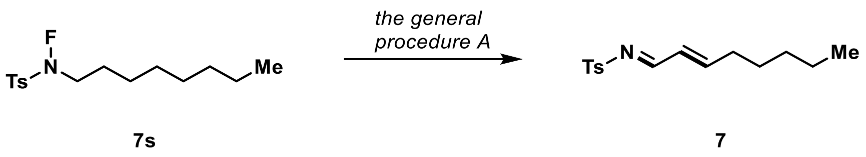

Product **7** was prepared by the general procedure A. Purification using column chromatography (PE/EA = 20:1) afforded **7** as yellow oil (43.0 mg, 0.15 mmol, 77%, *E/Z* > 20:1). **^{1}H NMR** (600 MHz, acetone-*d*$_6$) *δ* 8.62 (d, *J* = 9.6 Hz, 1H), 7.79 (d, *J* = 8.4 Hz, 2H), 7.43 (d, *J* = 8.4 Hz, 2H), 7.13 (dt, *J* = 15.6, 7.2 Hz, 1H), 6.36 (dd, *J* = 15.6, 9.6 Hz, 1H), 2.42 (s, 3H), 2.37 (dt, *J* = 15.6, 7.2 Hz, 2H), 1.52 - 1.48 (m, 2H), 1.34 - 1.30 (m, 4H), 0.88 (t, *J* = 7.2 Hz, 3H). **^{13}C NMR** (150 MHz, acetone-*d*$_6$) *δ* 172.4, 162.1, 145.2, 136.9, 130.6, 129.0, 128.6, 34.1, 32.0, 28.2, 23.0, 21.5, 14.2. **IR** ν_{max} (film): 2956 2927 1657 1526 1298 1157 1094 900 812 704 664 533 cm^{-1}. **HRMS** (ESI) m/z calcd for $C_{15}H_{21}NNaO_2S$ [M+Na]$^+$: 302.1185; found: 302.1181.

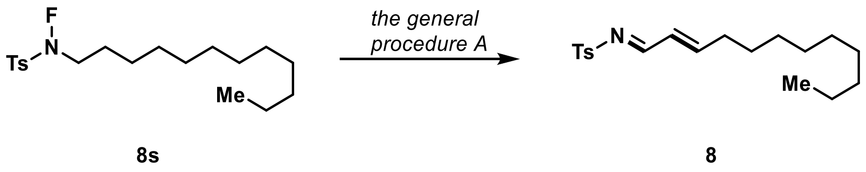

Product **8** was prepared by the general procedure A. Purification using column chromatography (PE/EA = 20:1) afforded **8** as yellow oil (42.2 mg, 0.13 mmol, 63%, *E/Z* > 20:1). **^{1}H NMR** (600 MHz, acetone-*d*$_6$) *δ* 8.62 (d, *J* = 9.0 Hz, 1H), 7.78 (d, *J* = 8.4 Hz, 2H), 7.43 (d, *J* = 8.4 Hz, 2H), 7.14 (dt, *J* = 15.6, 7.2 Hz, 1H), 6.37 (dd, *J* = 15.6, 9.0 Hz, 1H), 2.43 (s, 3H), 2.38 (dt, *J* = 15.6, 7.2 Hz, 2H), 1.54 - 1.49 (m, 2H), 1.34 - 1.27 (m, 12H), 0.87 (t, *J* = 7.2 Hz, 3H). **^{13}C NMR** (150 MHz, acetone-*d*$_6$) *δ* 172.5, 162.1, 145.2, 137.0, 130.6, 129.0, 128.6, 34.1, 32.6, 30.2, 30.1, 30.0, 29.8, 28.6, 23.3, 21.5, 14.4. **IR** ν_{max} (film): 2925 2854 1693 1529 1302 1161 1098 905 815 704 535 cm^{-1}. **HRMS** (ESI) m/z calcd for $C_{19}H_{29}NNaO_2S$ [M+Na]$^+$: 358.1811; found: 358.1802.

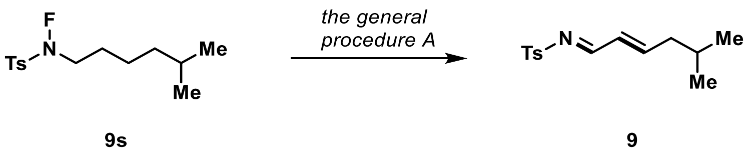

Product **9** was prepared by the general procedure A. Purification using column chromatography (PE/EA = 20:1) afforded **9** as yellow oil (45.1 mg, 0.17 mmol, 85%, *E/Z* > 20:1). **^{1}H NMR** (600 MHz, $CDCl_3$) *δ* 8.59 (d, *J* = 9.6 Hz, 1H), 7.82 (d, *J* = 8.4 Hz, 2H), 7.33 (d, *J* = 8.4 Hz, 2H), 6.85 (dd, *J* = 15.6, 7.2 Hz, 1H), 6.35 (dd, *J* = 15.6, 9.6 Hz, 1H), 2.43 (s, 3H), 2.23 (dd, *J* = 6.6, 6.6 Hz, 2H), 1.83 - 1.79 (m, 1H), 0.93 (d, *J* = 6.6 Hz, 6H). **^{13}C NMR** (150 MHz, $CDCl_3$) *δ* 171.2, 159.7, 144.5,

135.4, 129.9, 129.5, 128.0, 42.8, 28.1, 22.4, 21.7. **IR** ν_{max} (film): 2956 2926 1636 1527 1301 1160 1096 903 814 704 667 535 cm^{-1}. **HRMS** (ESI) m/z calcd for $C_{14}H_{19}NNaO_2S$ [M+Na]$^+$: 288.1029; found: 288.1036.

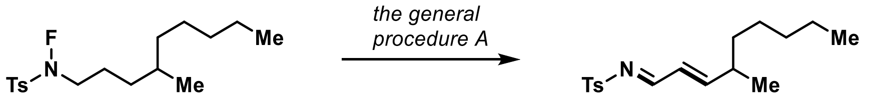

10s **10**

Product **10** was prepared by the general procedure A. Purification using column chromatography (PE/EA = 20:1) afforded **10** as yellow oil (47.9 mg, 0.16 mmol, 78%, *E/Z* > 20:1). **^{1}H NMR** (600 MHz, acetone-d_6) δ 8.63 (d, J = 9.6 Hz, 1H), 7.79 (d, J = 8.4 Hz, 2H), 7.43 (d, J = 8.4 Hz, 2H), 7.02 (dd, J = 15.6, 8.4 Hz, 1H), 6.33 (dd, J = 15.6, 9.6 Hz, 1H), 2.51 - 2.46 (m, 1H), 2.42 (s, 3H), 1.45 - 1.40 (m, 2H), 1.31 - 1.25 (m, 6H), 1.08 (d, J = 7.2 Hz, 3H), 0.86 (t, J = 6.6 Hz, 3H). **^{13}C NMR** (150 MHz, acetone-d_6) δ 172.5, 167.1, 145.2, 136.9, 130.6, 128.6, 127.3, 38.5, 36.5, 32.5, 27.5, 23.1, 21.5, 19.4, 14.3. **IR** ν_{max} (film): 2928 2858 1696 1527 1298 1157 1096 902 812 703 664 532 cm^{-1}. **HRMS** (ESI) m/z calcd for $C_{17}H_{25}NNaO_2S$ [M+Na]$^+$: 330.1498; found: 330.1498.

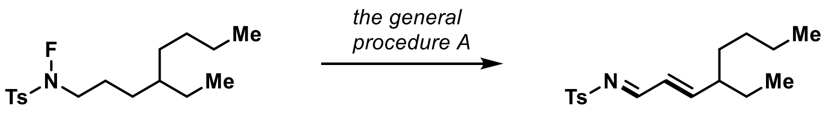

11s **11**

Product **11** was prepared by the general procedure A. Purification using column chromatography (PE/EA = 20:1) afforded **11** as yellow oil (48.5 mg, 0.16 mmol, 79%, *E/Z* > 20:1). **^{1}H NMR** (600 MHz, acetone-d_6) δ 8.64 (d, J = 9.6 Hz, 1H), 7.79 (d, J = 8.4 Hz, 2H), 7.43 (d, J = 8.4 Hz, 2H), 6.93 (dd, J = 15.6, 9.6 Hz, 1H), 6.33 (dd, J = 15.6, 9.6 Hz, 1H), 2.43 (s, 3H), 2.31 - 2.25 (m, 1H), 1.57 - 1.50 (m, 2H), 1.44 - 1.38 (m, 2H), 1.31 - 1.22 (m, 4H), 0.86 (t, J = 7.2 Hz, 6H). **^{13}C NMR** (150 MHz, acetone-d_6) δ 172.3, 166.2, 145.2, 136.9, 130.6, 129.0, 128.6, 46.3, 34.3, 30.1, 27.8, 23.3, 21.5, 14.2, 11.9. **IR** ν_{max} (film): 2958 2925 1692 1525 1299 1154 1094 902 811 703 670 533 cm^{-1}. **HRMS** (ESI) m/z calcd for $C_{17}H_{25}NNaO_2S$ [M+Na]$^+$: 330.1498; found: 330.1488.

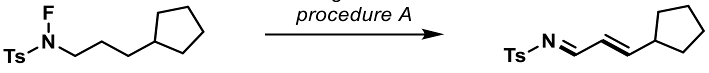

12s **12**

Product **12** was prepared by the general procedure A. Purification using column chromatography (PE/EA = 20:1) afforded **12** as yellow oil (44.3 mg, 0.16 mmol, 80%, *E/Z* > 20:1). **^{1}H NMR** (500 MHz, acetone-d_6) δ 8.61 (d, J = 9.5 Hz, 1H), 7.78 (d, J = 8.5 Hz, 2H), 7.43 (d, J = 8.5 Hz, 2H), 7.09 (dd, J = 15.5, 8.5 Hz, 1H), 6.33 (ddd, J = 15.5, 9.5, 1.0 Hz, 1H), 2.80 - 2.74 (m, 1H), 2.43 (s, 3H), 1.91 - 1.84 (m, 2H), 1.74 - 1.67 (m, 2H), 1.66 - 1.58 (m, 2H), 1.49 - 1.41 (m, 2H). **^{13}C NMR** (125 MHz, acetone-d_6) δ 172.6, 166.1, 145.2, 137.0, 130.6, 128.6, 127.2, 44.8, 33.0, 25.9, 21.5. **IR** ν_{max} (film): 2956 2869 1693 1527 1300 1159 1096 902 814 750 704 665 534 cm^{-1}. **HRMS** (ESI) m/z calcd for $C_{15}H_{19}NNaO_2S$ [M+Na]$^+$: 300.1029; found: 300.1018.

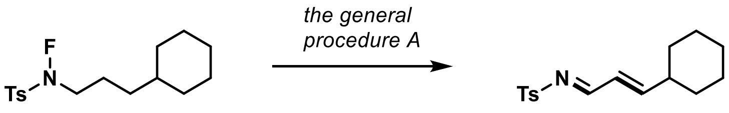

13s **13**

Product **13** was prepared by the general procedure A. Purification using column chromatography (PE/EA = 20:1) afforded **13** as yellow oil (48.3 mg, 0.17 mmol, 83%, *E/Z* > 20:1). **^{1}H NMR** (600 MHz, acetone-d_6) δ 8.61 (d, J = 9.6 Hz, 1H), 7.79 (d, J = 8.4 Hz, 2H), 7.43 (d, J = 8.4 Hz, 2H), 7.05 (dd, J = 15.6, 7.2 Hz, 1H), 6.30 (dd, J = 15.6, 9.6 Hz, 1H), 2.43 (s, 3H), 2.36 - 2.30 (m, 1H), 1.81 - 1.78 (m, 2H), 1.77 - 1.72 (m, 2H), 1.67 - 1.64 (m, 1H), 1.36 - 1.33 (m, 1H), 1.32 - 1.29 (m, 1H), 1.24 - 1.17 (m, 3H). **^{13}C NMR** (150 MHz, acetone-d_6) δ 172.8, 166.5, 145.2, 136.9, 130.6, 128.6, 126.7, 42.3, 32.1, 26.5, 26.2, 21.5. **IR** ν_{max} (film): 2925 2852 1696 1527 1301 1160 1097 902 815 750 704 668 535 cm^{-1}. **HRMS** (ESI) m/z calcd for $C_{16}H_{21}NNaO_2S$ [M+Na]$^+$: 314.1185; found: 314.1175.

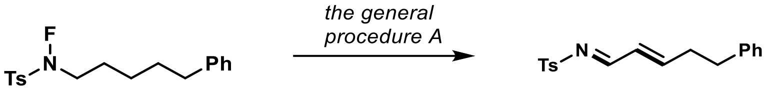

14s **14**

Product **14** was prepared by the general procedure A. Purification using column chromatography (PE/EA = 20:1) afforded **14** as yellow oil (50.7 mg, 0.16 mmol, 81%, *E/Z* > 20:1). **^{1}H NMR** (600 MHz, acetone-d_6) δ 8.61 (d, J = 9.6 Hz, 1H), 7.77 (d, J = 8.4 Hz, 2H), 7.43 (d, J = 8.4 Hz, 2H), 7.29 - 7.24 (m, 4H), 7.19 - 7.13 (m, 2H), 6.38 (dd, J = 15.6, 9.6 Hz, 1H), 2.85 (t, J = 7.8 Hz, 2H), 2.71 (dt, J = 15.6, 7.8 Hz, 2H), 2.43 (s, 3H). **^{13}C NMR** (150 MHz, acetone-d_6) δ 172.4, 160.8, 145.3, 141.7, 136.9, 130.7, 129.4, 129.3, 129.2, 128.6, 127.0, 35.8, 34.6, 21.5. **IR** ν_{max} (film): 2959 2926 1694 1527 1302 1161 1097 904 813 704 671 535 cm^{-1}. **HRMS** (ESI) m/z calcd for $C_{18}H_{19}NNaO_2S$ [M+Na]$^+$: 336.1029; found: 336.1036.

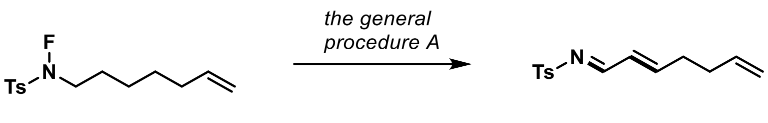

15s **15**

Product **15** was prepared by the general procedure A. Purification using column chromatography (PE/EA = 20:1) afforded **15** as yellow oil (29.5 mg, 0.11 mmol, 56%, *E/Z* > 20:1). **^{1}H NMR** (600 MHz, $CDCl_3$) δ 8.58 (d, J = 9.6 Hz, 1H), 7.82 (d, J = 8.4 Hz, 2H), 7.33 (d, J = 8.4 Hz, 2H), 6.86 (dt, J = 15.6, 7.2 Hz, 1H), 6.37 (dd, J = 15.6, 9.6 Hz, 1H), 5.77 (ddt, J = 14.4, 10.2, 6.6 Hz, 1H), 5.07 - 5.02 (m, 2H), 2.44 (dt, J = 14.4, 7.2 Hz, 2H), 2.43 (s, 3H), 2.26 (dt, J = 14.4, 7.2 Hz, 2H). **^{13}C NMR** (150 MHz, $CDCl_3$) δ 171.2, 159.4, 144.6, 136.5, 135.4, 129.9, 128.9, 128.1, 116.2, 32.8, 31.9, 21.8. **IR** ν_{max} (film): 2957 2924 1692 1656 1526 1301 1163 1099 904 814 703 670 535 cm^{-1}. **HRMS** (ESI) m/z calcd for $C_{14}H_{17}NNaO_2S$ $[M+Na]^+$: 286.0872; found: 286.0867.

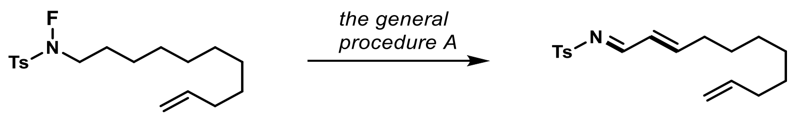

16s **16**

Product **16** was prepared by the general procedure A. Purification using column chromatography (PE/EA = 20:1) afforded **16** as yellow oil (46.6 mg, 0.15 mmol, 73%, *E/Z* > 20:1). **^{1}H NMR** (600 MHz, acetone-d_6) δ 8.62 (d, J = 9.6 Hz, 1H), 7.78 (d, J = 8.4 Hz, 2H), 7.42 (d, J = 8.4 Hz, 2H), 7.12 (dt, J = 15.6, 7.2 Hz, 1H), 6.36 (dd, J = 15.6, 9.6 Hz, 1H), 5.80 (ddt, J = 14.4, 10.2, 6.6 Hz, 1H), 4.99 - 4.89 (m, 2H), 2.43 (s, 3H), 2.38 (dt, J = 15.6, 7.2 Hz, 2H), 2.03 (dt, J = 14.4, 7.2 Hz, 2H), 1.54 - 1.49 (m, 2H), 1.39 - 1.33 (m, 6H). **^{13}C NMR** (150 MHz, acetone-d_6) δ 172.4, 162.0, 145.2, 139.7, 137.0, 130.6, 129.0, 128.6, 114.7, 34.4, 34.1, 29.6, 29.51, 29.48, 28.5, 21.5. **IR** ν_{max} (film): 2921 2851 1701 1641 1525 1291 1156 1090 907 813 702 678 534 cm^{-1}. **HRMS** (ESI) m/z calcd for $C_{18}H_{25}NNaO_2S$ $[M+Na]^+$: 342.1498; found: 342.1505.

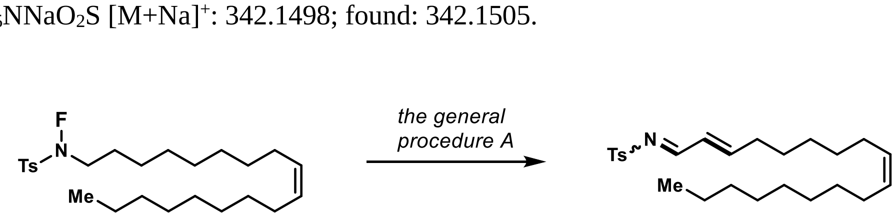

17s **17**

Product **17** was prepared by the general procedure A. Purification using column chromatography (PE/EA = 20:1) afforded **17** as yellow oil (54.2 mg, 0.13 mmol, 65%, *E/Z* = 9:1). **^{1}H NMR** (600 MHz, acetone-d_6) δ 9.51 (d, J = 7.8 Hz, 0.1H), 8.61 (d, J = 9.6 Hz, 0.9H), 7.77 (d, J = 8.4 Hz, 2H), 7.41 (d, J = 8.4 Hz, 2H), 7.11 (dt, J = 15.6, 7.2 Hz, 0.9H), 6.95 (dt, J = 15.6, 6.6 Hz, 0.1H), 6.35

(dd, J = 15.6, 9.6 Hz, 0.9H), 6.07 (dd, J = 15.6, 7.8 Hz, 0.1H), 5.37 - 5.31 (m, 2H), 2.42 (s, 3H), 2.37 (dt, J = 14.4, 7.2 Hz, 2H), 2.03 (dt, J = 14.4, 7.2 Hz, 4H), 1.54 - 1.51 (m, 2H), 1.39 - 1.36 (m, 4H), 1.30 - 1.28 (m, 12H), 0.87 (t, J = 6.6 Hz, 3H). **^{13}C NMR** (150 MHz, acetone-d_6) δ 194.0, 172.31, 172.29, 161.9, 161.8, 159.1, 145.1, 137.0, 133.7, 131.2, 130.8, 130.7, 130.6, 130.3, 129.02, 129.00, 128.7, 128.6, 34.12, 34.10, 33.2, 32.6, 30.43, 30.37, 30.36, 30.35, 30.34, 30.32, 30.22, 30.19, 30.15, 30.14, 30.06, 30.04, 30.01, 29.95, 29.4, 28.6, 28.5, 28.4, 27.8, 27.7, 27.6, 23.3, 21.5, 14.4. **IR** ν_{max} (film): 2925 2854 1691 1637 1589 1325 1158 1091 970 815 705 677 553 cm^{-1}. **HRMS** (ESI) m/z calcd for $C_{25}H_{39}NNaO_2S$ [M+Na]$^+$: 440.2594; found: 440.2596.

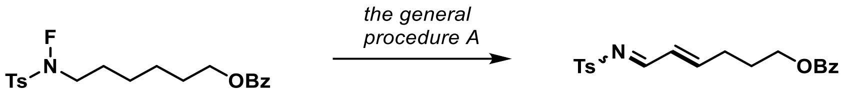

19s **19**

Product **19** was prepared by the general procedure A. Purification using column chromatography (PE/EA = 10:1) afforded **19** as yellow oil (52.7 mg, 0.14 mmol, 71%, E/Z = 9:1). **^{1}H NMR** (600 MHz, acetone-d_6) δ 9.51 (d, J = 7.8 Hz, 0.1H), 8.62 (d, J = 9.6 Hz, 0.9H), 8.01 - 7.99 (m, 2.2H), 7.76 (d, J = 8.4 Hz, 1.8H), 7.60 (t, J = 7.2 Hz, 1H), 7.49 - 7.46 (m, 2.2H), 7.40 (d, J = 8.4 Hz, 1.8H), 7.18 (dt, J = 15.6, 6.6 Hz, 0.9H), 7.02 (dt, J = 15.6, 6.6 Hz, 0.1H), 6.42 (dd, J = 15.6, 9.6 Hz, 0.9H), 6.13 (dd, J = 15.6, 7.8 Hz, 0.1H), 4.37 - 4.33 (m, 2H), 2.57 (dt, J = 15.6, 7.2 Hz, 2H), 2.40 (s, 3H), 2.03 - 1.98 (m, 2H). **^{13}C NMR** (150 MHz, acetone-d_6) δ 194.1, 172.3, 166.6, 160.8, 158.1, 145.3, 136.9, 134.0, 133.8, 131.3, 131.2, 130.6, 130.1, 129.4, 128.6, 64.7, 30.9, 27.8, 21.5. **IR** ν_{max} (film): 2961 2839 1717 1601 1583 1317 1163 1071 979 814 714 667 557 cm^{-1}. **HRMS** (ESI) m/z calcd for $C_{20}H_{21}NNaO_4S$ [M+Na]$^+$: 394.1083; found: 394.1089.

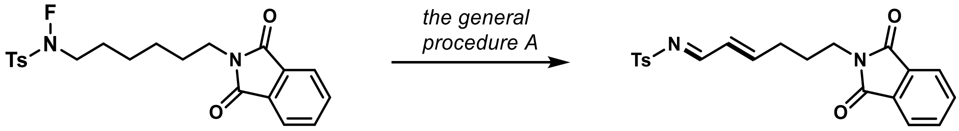

20s **20**

Product **20** was prepared by the general procedure A. Purification using column chromatography (PE/EA = 10:1) afforded **20** as yellow oil (49.9 mg, 0.13 mmol, 63%, E/Z > 20:1). **^{1}H NMR** (600 MHz, CD_2Cl_2) δ 8.51 (d, J = 9.6 Hz, 1H), 7.82 (dd, J = 6.0, 3.0 Hz, 2H), 7.76 (d, J = 7.8 Hz, 2H), 7.72 (dd, J = 6.0, 3.0 Hz, 2H), 7.35 (d, J = 7.8 Hz, 2H), 6.68 (dt, J = 15.6, 7.2 Hz, 1H), 6.38 (dd, J = 15.6, 9.6 Hz, 1H), 3.70 (t, J = 7.2 Hz, 2H), 2.43 - 2.39 (m, 5H), 1.92 - 1.87 (m, 2H). **^{13}C NMR** (150 MHz, CD_2Cl_2) δ 171.4, 168.6, 159.0, 145.1, 135.7, 134.4, 132.5, 130.2, 129.3, 128.2, 123.5, 37.5, 31.2, 27.2, 21.8. **IR** ν_{max} (film): 3265 2949 2838 1713 1654 1650 1399 1164 1097 980 815

722 664 541 cm^{-1}. **HRMS** (ESI) m/z calcd for $C_{21}H_{20}N_2NaO_4S$ $[M+Na]^+$: 419.1036; found: 419.1050.

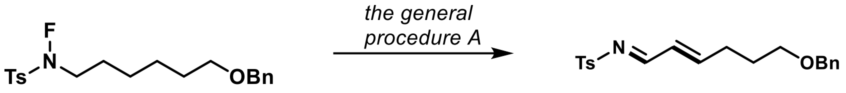

21s 21

Product **21** was prepared by the general procedure A. Purification using column chromatography (PE/EA = 10:1) afforded **21** as yellow oil (51.4 mg, 0.14 mmol, 72%, *E/Z* > 20:1). ^{1}**H NMR** (600 MHz, acetone-d_6) δ 8.61 (d, J = 9.6 Hz, 1H), 7.79 (d, J = 7.8 Hz, 2H), 7.42 (d, J = 7.8 Hz, 2H), 7.34 - 7.30 (m, 4H), 7.28 - 7.24 (m, 1H), 7.12 (dt, J = 15.6, 7.2 Hz, 1H), 6.37 (dd, J = 15.6, 9.6 Hz, 1H), 4.80 (s, 2H), 3.51 (t, J = 6.0 Hz, 2H), 2.48 (dt, J = 15.6, 7.2 Hz, 2H), 2.42 (s, 3H), 1.83 - 1.78 (m, 2H). ^{13}**C NMR** (150 MHz, acetone-d_6) δ 172.4, 161.7, 145.2, 139.8, 137.0, 130.6, 129.1, 129.0, 128.6, 128.3, 128.1, 73.2, 69.8, 31.1, 28.8, 21.5. **IR** ν_{max} (film): 2877 2836 1600 1552 1337 1164 1098 904 814 742 664 541 cm^{-1}. **HRMS** (ESI) m/z calcd for $C_{20}H_{23}NNaO_3S$ $[M+Na]^+$: 380.1291; found: 380.1280.

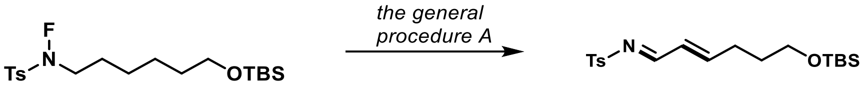

22s 22

Product **22** was prepared by the general procedure A. Purification using column chromatography (PE/EA = 10:1) afforded **22** as yellow oil (53.4 mg, 0.14 mmol, 70%, *E/Z* > 20:1). ^{1}**H NMR** (600 MHz, acetone-d_6) δ 8.61 (d, J = 9.6 Hz, 1H), 7.78 (d, J = 7.8 Hz, 2H), 7.44 (d, J = 7.8 Hz, 2H), 7.17 (dt, J = 15.6, 6.6 Hz, 1H), 6.37 (dd, J = 15.6, 9.6 Hz, 1H), 3.69 (t, J = 6.0 Hz, 2H), 2.48 (dt, J = 15.6, 7.2 Hz, 2H), 2.43 (s, 3H), 1.76 - 1.71 (m, 2H), 0.88 (s, 9H), 0.05 (s, 6H). ^{13}**C NMR** (150 MHz, acetone-d_6) δ 172.5, 162.0, 145.2, 137.0, 130.6, 129.0, 128.6, 62.8, 31.8, 30.9, 26.3, 21.5, 18.8, -5.2. **IR** ν_{max} (film): 2954 2858 1637 1589 1306 1158 1091 961 814 776 705 553 cm^{-1}. **HRMS** (ESI) m/z calcd for $C_{19}H_{31}NNaO_3SSi$ $[M+Na]^+$: 404.1686; found: 404.1687.

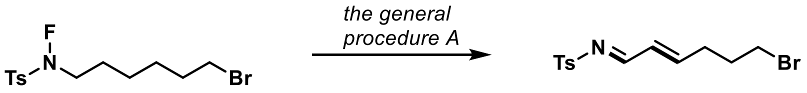

23s 23

Product **23** was prepared by the general procedure A. Purification using column chromatography (PE/EA = 20:1) afforded **23** as yellow oil (42.1 mg, 0.13 mmol, 64%, *E/Z* > 20:1). ^{1}**H NMR** (500

MHz, acetone-*d*$_6$) *δ* 8.62 (d, *J* = 9.5 Hz, 1H), 7.78 (d, *J* = 8.5 Hz, 2H), 7.44 (d, *J* = 8.5 Hz, 2H), 7.14 (dt, *J* = 15.5, 7.0 Hz, 1H), 6.42 (dd, *J* = 15.5, 9.5 Hz, 1H), 3.55 (t, *J* = 6.5 Hz, 2H), 2.56 (dt, *J* = 15.5, 7.0 Hz, 2H), 2.43 (s, 3H), 2.12 - 2.06 (m, 2H). **^{13}C NMR** (125 MHz, acetone-*d*$_6$) *δ* 172.3, 159.8, 145.3, 136.8, 130.7, 129.7, 128.6, 33.7, 32.5, 31.6, 21.5. **IR** ν_{max} (film): 2954 2885 1636 1596 1334 1159 1091 996 814 777 705 664 556 cm^{-1}. **HRMS** (ESI) m/z calcd for $C_{13}H_{16}BrNNaO_2S$ [M+Na]$^+$: 351.9977; found: 351.9973.

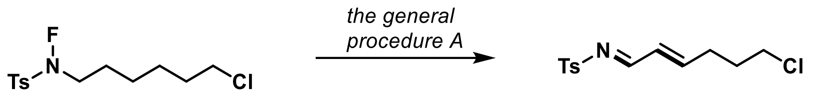

24s **24**

Product **24** was prepared by the general procedure A. Purification using column chromatography (PE/EA = 20:1) afforded **24** as yellow oil (37.6 mg, 0.13 mmol, 66%, *E/Z* > 20:1). **^{1}H NMR** (600 MHz, acetone-*d*$_6$) *δ* 8.62 (d, *J* = 9.0 Hz, 1H), 7.78 (d, *J* = 8.4 Hz, 2H), 7.43 (d, *J* = 8.4 Hz, 2H), 7.14 (dt, *J* = 15.6, 7.2 Hz, 1H), 6.41 (dd, *J* = 15.6, 9.0 Hz, 1H), 3.66 (t, *J* = 6.6 Hz, 2H), 2.55 (dt, *J* = 15.6, 7.2 Hz, 2H), 2.43 (s, 3H), 2.03 - 1.98 (m, 2H). **^{13}C NMR** (150 MHz, acetone-*d*$_6$) *δ* 172.3, 159.9, 145.3, 136.9, 130.7, 129.7, 128.6, 44.9, 31.5, 31.3, 21.5. **IR** ν_{max} (film): 2953 2884 1635 1594 1332 1157 1090 964 813 776 704 660 553 cm^{-1}. **HRMS** (ESI) m/z calcd for $C_{13}H_{16}ClNNaO_2S$ [M+Na]$^+$: 308.0482; found: 308.0471.

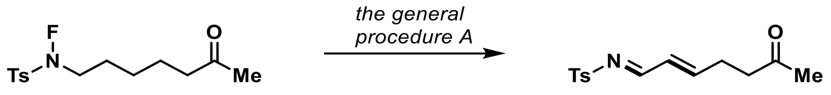

25s **25**

Product **25** was prepared by the general procedure A. Purification using column chromatography (PE/EA = 20:1) afforded **25** as yellow oil (40.2 mg, 0.14 mmol, 72%, *E/Z* > 20:1). **^{1}H NMR** (600 MHz, acetone-*d*$_6$) *δ* 8.59 (d, *J* = 9.6 Hz, 1H), 7.78 (d, *J* = 8.4 Hz, 2H), 7.43 (d, *J* = 8.4 Hz, 2H), 7.10 (dt, *J* = 15.6, 7.2 Hz, 1H), 6.35 (dd, *J* = 15.6, 9.6 Hz, 1H), 2.74 (t, *J* = 7.2 Hz, 2H), 2.57 (dt, *J* = 15.6, 7.2 Hz, 2H), 2.43 (s, 3H), 2.12 (s, 3H). **^{13}C NMR** (150 MHz, acetone-*d*$_6$) *δ* 206.5, 172.4, 160.8, 145.3, 136.8, 130.6, 129.1, 128.6, 41.3, 29.7, 28.0, 21.5. **IR** ν_{max} (film): 2960 2838 1716 1637 1586 1320 1156 1089 969 816 777 676 585 556 cm^{-1}. **HRMS** (ESI) m/z calcd for $C_{14}H_{17}NNaO_3S$ [M+Na]$^+$: 302.0821; found: 302.0831.

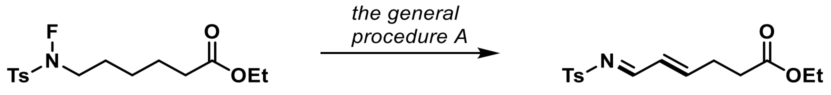

26s **26**

Product **26** was prepared by the general procedure A. Purification using column chromatography (PE/EA = 10:1) afforded **26** as yellow oil (42.0 mg, 0.14 mmol, 68%, *E/Z* > 20:1). **^{1}H NMR** (600 MHz, acetone-*d$_6$*) δ 8.61 (d, *J* = 9.6 Hz, 1H), 7.78 (d, *J* = 8.4 Hz, 2H), 7.44 (d, *J* = 8.4 Hz, 2H), 7.13 (dt, *J* = 15.6, 6.6 Hz, 1H), 6.40 (dd, *J* = 15.6, 9.6 Hz, 1H), 4.09 (q, *J* = 7.2 Hz, 2H), 2.66 (dt, *J* = 15.6, 7.2 Hz, 2H), 2.55 (t, *J* = 7.2 Hz, 2H), 2.43 (s, 3H), 1.19 (t, *J* = 7.2 Hz, 3H). **^{13}C NMR** (150 MHz, acetone-*d$_6$*) δ 172.4, 172.3, 159.7, 145.3, 136.8, 130.7, 129.4, 128.7, 60.9, 32.6, 29.2, 21.5, 14.5. **IR** ν_{max} (film): 2985 2880 1732 1685 1478 1341 1169 1035 963 815 776 661 540 cm^{-1}. **HRMS** (ESI) m/z calcd for $C_{15}H_{19}NNaO_4S$ [M+Na]$^+$: 332.0927; found: 332.0921.

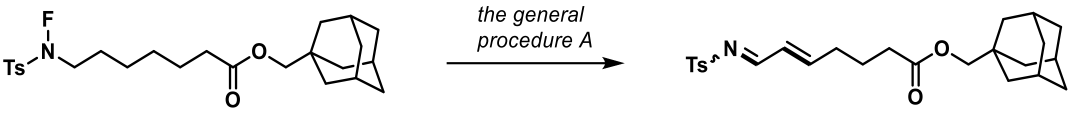

27s **27**

Product **27** was prepared by the general procedure A. Purification using column chromatography (PE/EA = 10:1) afforded **27** as yellow oil (63.8 mg, 0.14 mmol, 72%, *E/Z* = 9:1). **^{1}H NMR** (600 MHz, acetone-*d$_6$*) δ 9.53 (d, *J* = 7.8 Hz, 0.1H), 8.63 (d, *J* = 9.6 Hz, 0.9H), 7.78 (d, *J* = 8.4 Hz, 2H), 7.42 (d, *J* = 8.4 Hz, 2H), 7.11 (dt, *J* = 15.6, 6.6 Hz, 0.9H), 6.97 (dt, *J* = 15.6, 6.6 Hz, 0.1H), 6.38 (dd, *J* = 15.6, 9.6 Hz, 0.9H), 6.10 (dd, *J* = 15.6, 7.8 Hz, 0.1H), 3.67 (s, 0.2H), 3.65 (s, 1.8H), 2.44 (dt, *J* = 15.6, 7.2 Hz, 2H), 2.43 (s, 3H), 2.40 (t, *J* = 7.2 Hz, 2H), 1.95 - 1.93 (m, 3H), 1.87 - 1.82 (m, 2H), 1.73 - 1.71 (m, 3H), 1.66 - 1.64 (m, 3H), 1.56 - 1.55 (m, 1H), 1.54 - 1.53 (m, 5H). **^{13}C NMR** (150 MHz, acetone-*d$_6$*) δ 194.0, 173.1, 172.2, 160.7, 158.1, 145.2, 136.9, 134.1, 130.6, 129.5, 128.6, 74.1, 39.88, 39.86, 37.6, 33.87, 33.84, 33.78, 33.74, 33.4, 32.5, 28.91, 28.89, 23.9, 21.5. **IR** ν_{max} (film): 2904 2849 1732 1654 1598 1336 1164 1097 910 813 667 557 541cm^{-1}. **HRMS** (ESI) m/z calcd for $C_{25}H_{33}NNaO_4S$ [M+Na]$^+$: 466.2023; found: 466.2025.

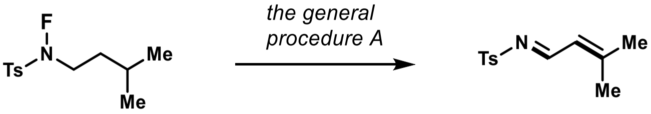

28s **28**

Product **28** was prepared by the general procedure A. Purification using column chromatography (PE/EA = 20:1) afforded **28** as yellow oil (30.8 mg, 0.13 mmol, 65%, *E/Z* > 20:1). **^{1}H NMR** (600 MHz, acetone-*d$_6$*) δ 8.94 (d, *J* = 10.2 Hz, 1H), 7.78 (d, *J* = 8.4 Hz, 2H), 7.43 (d, *J* = 8.4 Hz, 2H), 6.15 (d, *J* = 10.2 Hz, 1H), 2.43 (s, 3H), 2.17 (s, 3H), 2.06 (s, 3H). **^{13}C NMR** (150 MHz, acetone-*d$_6$*) δ 168.1, 165.8, 145.1, 137.3, 130.6, 128.5, 124.2, 27.6, 21.5, 19.9. **IR** ν_{max} (film): 2956 2926 1636

1527 1301 1160 1096 903 814 704 667 535 cm^{-1}. **HRMS** (ESI) m/z calcd for $C_{12}H_{15}NNaO_2S$ $[M+Na]^+$: 260.0716; found: 260.0720.

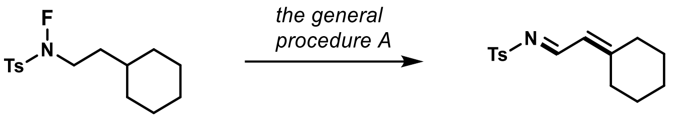

29s **29**

Product **29** was prepared by the general procedure A. Purification using column chromatography (PE/EA = 20:1) afforded **29** as yellow oil (39.4 mg, 0.14 mmol, 71%, *E/Z* > 20:1). **^{1}H NMR** (600 MHz, acetone-d_6) δ 9.01 (d, J = 10.2 Hz, 1H), 7.78 (d, J = 8.4 Hz, 2H), 7.42 (d, J = 8.4 Hz, 2H), 6.10 (d, J = 10.2 Hz, 1H), 2.66 (t, J = 6.0 Hz, 2H), 2.42 (s, 3H), 2.39 (t, J = 6.0 Hz, 2H), 1.72 - 1.68 (m, 4H), 1.66 - 1.63 (m, 2H). **^{13}C NMR** (150 MHz, acetone-d_6) δ 172.9, 167.3, 145.0, 137.3, 130.6, 128.5, 121.3, 39.0, 31.1, 29.3, 29.0, 26.7, 21.5. **IR** ν_{max} (film): 2925 2852 1696 1531 1304 1165 1097 902 815 750 706 668 535 cm^{-1}. **HRMS** (ESI) m/z calcd for $C_{15}H_{19}NNaO_2S$ $[M+Na]^+$: 300.1029; found: 300.1025.

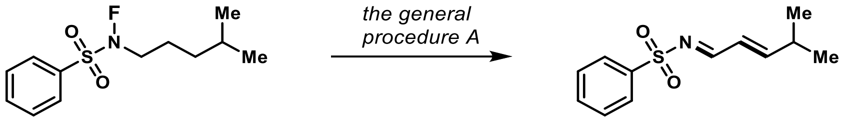

33s **33**

Product **33** was prepared by the general procedure A. Purification using column chromatography (PE/EA = 20:1) afforded **33** as yellow oil (34.1 mg, 0.14 mmol, 72%, *E/Z* > 20:1). **^{1}H NMR** (600 MHz, acetone-d_6) δ 8.65 (d, J = 9.6 Hz, 1H), 7.92 (d, J = 7.8 Hz, 2H), 7.72 (t, J = 7.8 Hz, 1H), 7.64 (t, J = 7.8 Hz, 2H), 7.11 (dd, J = 15.6, 6.6 Hz, 1H), 6.33 (dd, J = 15.6, 9.6 Hz, 1H), 2.67 - 2.61 (m, 1H), 1.10 (d, J = 6.6 Hz, 6H). **^{13}C NMR** (150 MHz, acetone-d_6) δ 173.2, 168.1, 139.9, 134.3, 130.1, 128.5, 126.4, 32.9, 21.2. **IR** ν_{max} (film): 2964 2927 1633 1586 1318 1157 1088 947 829 752 687 560 cm^{-1}. **HRMS** (ESI) m/z calcd for $C_{12}H_{15}NNaO_2S$ $[M+Na]^+$: 260.0716; found: 260.0719.

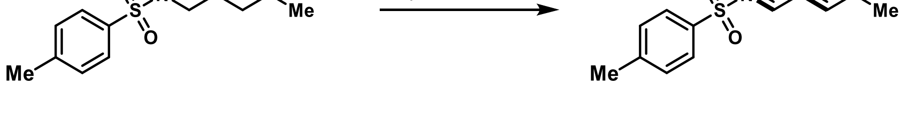

34s **34**

Product **34** was prepared by the general procedure A. Purification using column chromatography

(PE/EA = 20:1) afforded **34** as yellow oil (38.2 mg, 0.15 mmol, 76%, *E/Z* > 20:1). **^{1}H NMR** (600 MHz, $CDCl_3$) δ 8.59 (d, *J* = 9.6 Hz, 1H), 7.82 (d, *J* = 8.4 Hz, 2H), 7.33 (d, *J* = 8.4 Hz, 2H), 6.84 (dd, *J* = 15.6, 6.6 Hz, 1H), 6.32 (dd, *J* = 15.6, 9.6 Hz, 1H), 2.62 - 2.56 (m, 1H), 2.43 (s, 3H), 1.09 (d, *J* = 7.2 Hz, 6H). **^{13}C NMR** (150 MHz, $CDCl_3$) δ 171.6, 166.6, 144.5, 135.4, 129.8, 128.0, 125.8, 32.3, 21.7, 21.0. **IR** ν_{max} (film): 2965 2936 1633 1586 1459 1318 1159 1073 947 889 798 752 560 cm^{-1}. **HRMS** (ESI) m/z calcd for $C_{13}H_{17}NNaO_2S$ $[M+Na]^+$: 274.0872; found: 274.0880.

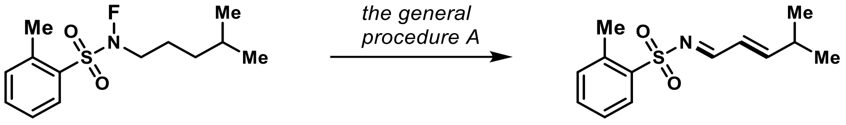

35s **35**

Product **35** was prepared by the general procedure A. Purification using column chromatography (PE/EA = 20:1) afforded **35** as yellow oil (41.7 mg, 0.17 mmol, 83%, *E/Z* > 20:1). **^{1}H NMR** (600 MHz, acetone-*d*$_6$) δ 8.69 (d, *J* = 9.6 Hz, 1H), 7.99 (d, *J* = 7.2 Hz, 1H), 7.58 (t, *J* = 7.2 Hz, 1H), 7.43 (t, *J* = 7.2 Hz, 2H), 7.13 (dd, *J* = 15.6, 6.6 Hz, 1H), 6.35 (dd, *J* = 15.6, 9.6 Hz, 1H), 2.67 - 2.64 (m, 1H), 2.63 (s, 3H), 1.10 (d, *J* = 6.6 Hz, 6H). **^{13}C NMR** (150 MHz, acetone-*d*$_6$) δ 173.3, 168.2, 139.2, 138.0, 134.4, 133.3, 129.6, 127.3, 126.4, 32.9, 21.2, 20.6. **IR** ν_{max} (film): 2969 2933 1634 1463 1323 1161 1065 898 805 750 597 552 cm^{-1}. **HRMS** (ESI) m/z calcd for $C_{13}H_{17}NNaO_2S$ $[M+Na]^+$: 274.0872; found: 274.0879.

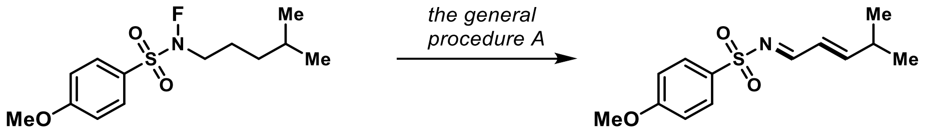

36s **36**

Product **36** was prepared by the general procedure A. Purification using column chromatography (PE/EA = 20:1) afforded **36** as yellow oil (38.5 mg, 0.14 mmol, 72%, *E/Z* > 20:1). **^{1}H NMR** (600 MHz, acetone-*d*$_6$) δ 8.59 (d, *J* = 9.0 Hz, 1H), 7.84 (d, *J* = 9.0 Hz, 2H), 7.13 (d, *J* = 9.0 Hz, 2H), 7.04 (dd, *J* = 15.6, 6.6 Hz, 1H), 6.30 (dd, *J* = 15.6, 9.0 Hz, 1H), 3.90 (s, 3H), 2.64 - 2.58 (m, 1H), 1.09 (d, *J* = 6.6 Hz, 6H). **^{13}C NMR** (150 MHz, acetone-*d*$_6$) δ 172.0, 167.2, 164.5, 131.1, 130.8, 126.3, 115.3, 56.2, 32.8, 21.2. **IR** ν_{max} (film): 2965 2871 1635 1464 1322 1154 1092 970 807 773 595 564 cm^{-1}. **HRMS** (ESI) m/z calcd for $C_{13}H_{17}NNaO_3S$ $[M+Na]^+$: 290.0821; found: 290.0834.

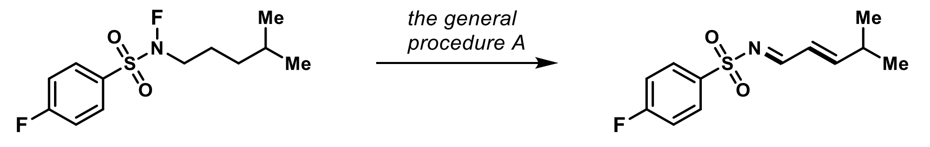

37s **37**

Product **37** was prepared by the general procedure A. Purification using column chromatography (PE/EA = 20:1) afforded **37** as yellow oil (37.8 mg, 0.15 mmol, 74%, *E/Z* > 20:1). **^{1}H NMR** (600 MHz, acetone-d_6) δ 8.65 (d, J = 9.6 Hz, 1H), 8.00 - 7.98 (m, 2H), 7.43 - 7.39 (m, 2H), 7.13 (dd, J = 15.6, 6.6 Hz, 1H), 6.33 (dd, J = 15.6, 9.6 Hz, 1H), 2.68 - 2.62 (m, 1H), 1.10 (d, J = 7.2 Hz, 6H). **^{13}C NMR** (150 MHz, acetone-d_6) δ 173.4, 168.4, 166.4 (d, J = 252.0 Hz), 136.2 (d, J = 3.0 Hz), 131.7 (d, J = 10.5 Hz), 126.3, 117.3 (d, J = 22.5 Hz), 33.0, 21.2. **^{19}F NMR** (565 MHz, $CDCl_3$) δ -101.13 - (-101.18) (m). **IR** ν_{max} (film): 2966 2926 1646 1457 1338 1168 1093 984 837 670 555 541 cm^{-1}. **HRMS** (ESI) m/z calcd for $C_{12}H_{14}FNNaO_2S$ $[M+Na]^+$: 278.0621; found: 278.0614.

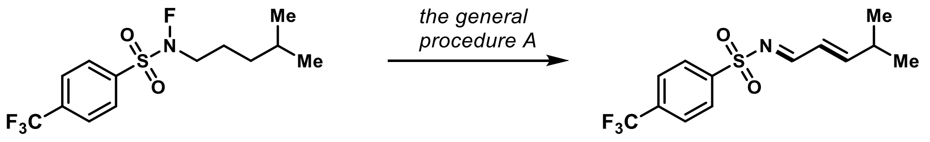

38s **38**

Product **38** was prepared by the general procedure A. Purification using column chromatography (PE/EA = 20:1) afforded **38** as yellow oil (45.2 mg, 0.15 mmol, 74%, *E/Z* > 20:1). **^{1}H NMR** (600 MHz, acetone-d_6) δ 8.73 (d, J = 9.0 Hz, 1H), 8.15 (d, J = 8.4 Hz, 2H), 8.00 (d, J = 8.4 Hz, 2H), 7.19 (dd, J = 15.6, 6.6 Hz, 1H), 6.36 (dd, J = 15.6, 9.0 Hz, 1H), 2.69 - 2.63 (m, 1H), 1.11 (d, J = 7.2 Hz, 6H). **^{13}C NMR** (150 MHz, acetone-d_6) δ 174.7, 169.4, 143.9, 135.1 (q, J = 33.0 Hz), 129.5, 127.3 (q, J = 4.5 Hz), 126.4, 124.5 (q, J = 270.0 Hz), 33.1, 21.2. **^{19}F NMR** (565 MHz, acetone-d_6) δ -63.71 (s). **IR** ν_{max} (film): 2967 2925 1634 1467 1323 1164 1091 970 831 773 716 601 554 cm^{-1}. **HRMS** (ESI) m/z calcd for $C_{13}H_{14}F_3NNaO_2S$ $[M+Na]^+$: 328.0590; found: 328.0603.

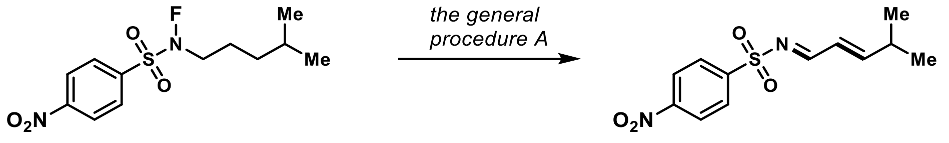

39s **39**

Product **39** was prepared by the general procedure A. Purification using column chromatography (PE/EA = 20:1) afforded **39** as yellow oil (30.5 mg, 0.11 mmol, 54%, *E/Z* > 20:1). **^{1}H NMR** (600 MHz, acetone-d_6) δ 8.75 (d, J = 9.0 Hz, 1H), 8.48 (d, J = 9.0 Hz, 2H), 8.20 (d, J = 9.0 Hz, 2H), 7.23

(dd, J = 15.6, 6.6 Hz, 1H), 6.38 (dd, J = 15.6, 9.0 Hz, 1H), 2.70 - 2.65 (m, 1H), 1.12 (d, J = 7.2 Hz, 6H). **^{13}C NMR** (150 MHz, acetone-d_6) δ 175.2, 167.0, 151.7, 145.4, 130.1, 126.4, 125.4, 33.1, 21.1. **IR** ν_{max} (film): 2966 2924 1646 1456 1348 1165 1090 987 814 738 615 546 cm^{-1}. **HRMS** (ESI) m/z calcd for $C_{12}H_{14}N_2NaO_4S$ [M+Na]$^+$: 305.0566; found: 305.0563.

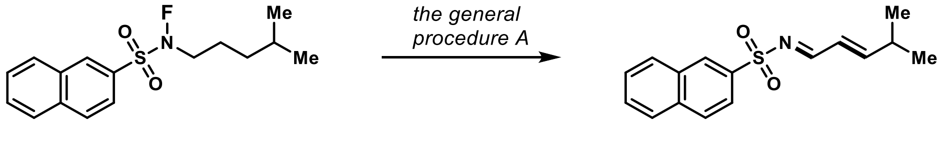

40s **40**

Product **40** was prepared by the general procedure A. Purification using column chromatography (PE/EA = 20:1) afforded **40** as yellow oil (40.2 mg, 0.14 mmol, 70%, *E/Z* > 20:1). **^{1}H NMR** (600 MHz, acetone-d_6) δ 8.70 (d, J = 9.0 Hz, 1H), 8.57 (s, 1H), 8.17 (d, J = 7.8 Hz, 1H), 8.13 (d, J = 7.8 Hz, 1H), 8.05 (d, J = 7.8 Hz, 1H), 7.88 (dd, J = 9.0, 1.8 Hz, 1H), 7.74 - 7.72 (m, 1H), 7.71 - 7.68 (m, 1H), 7.10 (dd, J = 15.6, 7.2 Hz, 1H), 6.33 (dd, J = 15.6, 9.0 Hz, 1H), 2.64 - 2.58 (m, 1H), 1.08 (d, J = 6.6 Hz, 6H). **^{13}C NMR** (150 MHz, acetone-d_6) δ 173.2, 168.1, 136.8, 136.0, 133.1, 130.3, 130.2, 130.1, 129.9, 128.8, 128.6, 126.4, 123.7, 32.9, 21.2. **IR** ν_{max} (film): 2963 2925 1632 1464 1347 1154 1073 968 823 773 670 552 cm^{-1}. **HRMS** (ESI) m/z calcd for $C_{16}H_{17}NNaO_2S$ [M+Na]$^+$: 310.0872; found: 310.0867.

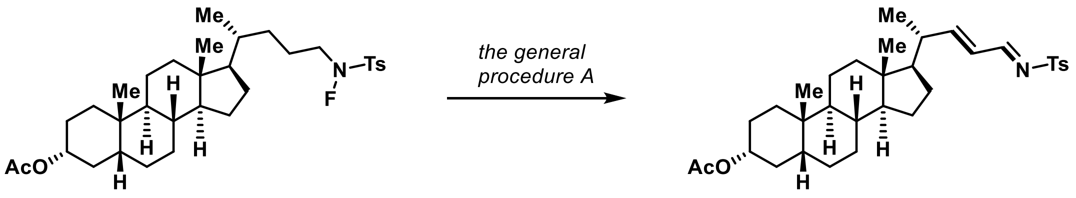

41s **41**

Product **41** was prepared by the general procedure A. Purification using column chromatography (PE/EA = 10:1) afforded **41** as yellow oil (83.0 mg, 0.15 mmol, 75%, *E/Z* > 20:1). **^{1}H NMR** (600 MHz, CD_2Cl_2) δ 8.53 (d, J = 9.6 Hz, 1H), 7.77 (d, J = 7.8 Hz, 2H), 7.35 (d, J = 7.8 Hz, 2H), 6.77 (dd, J = 15.6, 8.4 Hz, 1H), 6.27 (dd, J = 15.6, 9.6 Hz, 1H), 4.71 - 4.65 (m, 1H), 2.44 - 2.40 (m, 4H), 1.99 - 1.95 (m, 4H), 1.89 - 1.85 (m, 1H), 1.84 - 1.80 (m, 2H), 1.68 - 1.65 (m, 2H), 1.60 - 1.56 (m, 1H), 1.53 - 1.51 (m, 1H), 1.46 - 1.40 (m, 6H), 1.31 - 1.21 (m, 6H), 1.11 (d, J = 6.6 Hz, 3H), 1.08 - 1.01 (m, 3H), 0.94 (s, 3H), 0.69 (s, 3H). **^{13}C NMR** (150 MHz, CD_2Cl_2) δ 172.2, 170.6, 166.8, 145.0, 135.9, 130.1, 128.2, 126.5, 74.6, 56.6, 55.5, 43.6, 42.3, 41.5, 40.8, 40.4, 36.2, 35.4, 35.0, 32.6, 28.5, 27.4, 27.0, 26.7, 24.6, 23.5, 21.8, 21.6, 21.2, 19.1, 12.4. **IR** ν_{max} (film): 2938 2868 1732 1633 1449 1362 1158 1091 906 815 780 737 679 555 cm^{-1}. **HRMS** (ESI) m/z calcd for

$C_{33}H_{47}NNaO_4S$ $[M+Na]^+$: 576.3118; found: 576.3112.

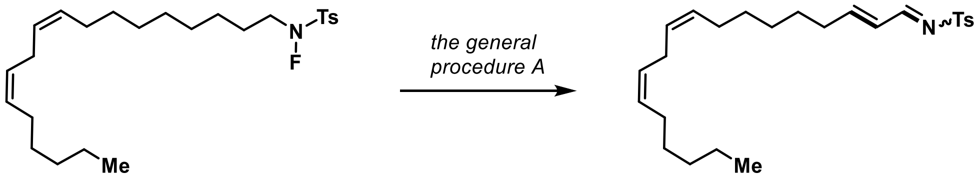

42s 42

Product **42** was prepared by the general procedure A. Purification using column chromatography (PE/EA = 20:1) afforded **42** as yellow oil (55.6 mg, 0.13 mmol, 67%, *E/Z* = 9:1). **^{1}H NMR** (600 MHz, acetone-d_6) δ 9.52 (d, J = 7.8 Hz, 0.1H), 8.62 (d, J = 9.6 Hz, 0.9H), 7.78 (d, J = 8.4 Hz, 2H), 7.43 (d, J = 8.4 Hz, 2H), 7.12 (dt, J = 15.6, 7.2 Hz, 0.9H), 6.97 (dt, J = 15.6, 6.6 Hz, 0.1H), 6.37 (dd, J = 15.6, 9.6 Hz, 0.9H), 6.08 (dd, J = 15.6, 7.8 Hz, 0.1H), 5.41 - 5.30 (m, 4H), 2.79 (t, J = 7.2 Hz, 2H), 2.43 (s, 3H), 2.39 (dt, J = 15.6, 7.2 Hz, 2H), 2.10 - 2.06 (m, 4H), 1.56 - 1.53 (m, 2H), 1.41 - 1.36 (m, 6H), 1.33 - 1.29 (m, 4H), 0.89 (t, J = 7.2 Hz, 3H). **^{13}C NMR** (150 MHz, acetone-d_6) δ 194.0, 172.3, 161.9, 159.2, 145.2, 137.0, 133.7, 130.7, 130.6, 130.5, 129.1, 128.9, 128.7, 128.6, 34.1, 32.2, 30.1, 30.0, 29.4, 28.5, 27.8, 27.7, 26.2, 23.2, 21.5, 14.4. **IR** ν_{max} (film): 2931 2860 1636 1457 1323 1159 1090 972 815 780 677 554 cm^{-1}. **HRMS** (ESI) m/z calcd for $C_{25}H_{37}NNaO_2S$ $[M+Na]^+$: 438.2437; found: 438.2433.

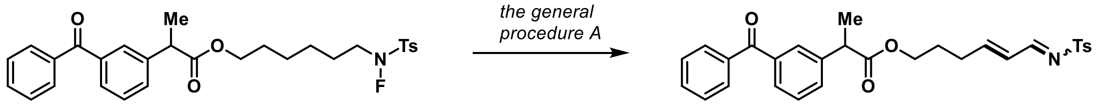

43s 43

Product **43** was prepared by the general procedure A. Purification using column chromatography (PE/EA = 10:1) afforded **43** as yellow oil (72.5 mg, 0.14 mmol, 72%, *E/Z* = 9:1). **^{1}H NMR** (600 MHz, acetone-d_6) δ 9.48 (d, J = 7.8 Hz, 0.1H), 8.60 (d, J = 9.6 Hz, 0.9H), 7.80 - 7.77 (m, 5H), 7.66 - 7.61 (m, 3H), 7.54 - 7.50 (m, 3H), 7.41 (d, J = 8.4 Hz, 2H), 7.04 (dt, J = 15.6, 7.2 Hz, 0.9H), 6.90 (dt, J = 15.6, 7.2 Hz, 0.1H), 6.30 (dd, J = 15.6, 9.6 Hz, 0.9H), 6.02 (dd, J = 15.6, 7.8 Hz, 0.1H), 4.14 - 4.09 (m, 2H), 3.91 (q, J = 7.2 Hz, 1H), 2.41 (s, 3H), 2.35 (dt, J = 15.6, 7.2 Hz, 2H), 1.82 - 1.78 (m, 2H), 1.51 (d, J = 7.2 Hz, 0.3H), 1.49 (d, J = 7.2 Hz, 2.7H). **^{13}C NMR** (150 MHz, acetone-d_6) δ 196.2, 194.0, 174.2, 172.2, 160.4, 157.9, 145.2, 142.2, 138.7, 138.4, 136.8, 133.9, 133.3, 132.3, 130.6, 130.5, 129.54, 129.47, 129.4, 129.3, 129.2, 128.6, 64.3, 45.8, 30.5, 27.6, 21.5, 18.7. **IR** ν_{max} (film): 2979 2927 1733 1659 1448 1320 1164 1096 975 815 768 666 557 cm^{-1}. **HRMS** (ESI) m/z calcd for $C_{29}H_{29}NNaO_5S$ $[M+Na]^+$: 526.1659; found: 526.1655.

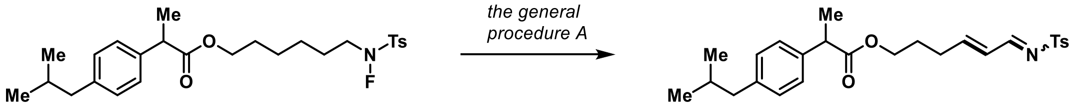

44s **44**

Product **44** was prepared by the general procedure A. Purification using column chromatography (PE/EA = 10:1) afforded **44** as yellow oil (61.9 mg, 0.14 mmol, 68%, *E/Z* = 19:1). **^{1}H NMR** (600 MHz, acetone-d_6) δ 9.48 (d, J = 7.8 Hz, 0.05H), 8.59 (d, J = 9.6 Hz, 0.95H), 7.79 (d, J = 8.4 Hz, 2H), 7.42 (d, J = 7.8 Hz, 2H), 7.22 (d, J = 7.8 Hz, 2H), 7.12 (d, J = 8.4 Hz, 2H), 7.00 (dt, J = 15.6, 7.2 Hz, 0.95H), 6.88 (dt, J = 15.6, 7.2 Hz, 0.05H), 6.26 (dd, J = 15.6, 9.6 Hz, 0.95H), 6.00 (dd, J = 15.6, 7.8 Hz, 0.05H), 4.13 - 4.09 (m, 1H), 4.07 - 4.03 (m, 1H), 3.73 (q, J = 7.2 Hz, 1H), 2.44 (d, J = 7.2 Hz, 2H), 2.42 (s, 3H), 2.30 (dt, J = 15.6, 7.2 Hz, 2H), 1.86 - 1.81 (m, 1H), 1.80 - 1.75 (m, 2H), 1.44 (d, J = 7.2 Hz, 0.15H), 1.42 (d, J = 7.2 Hz, 2.85H), 0.88 (d, J = 7.2 Hz, 0.3H), 0.87 (d, J = 7.2 Hz, 5.7H). **^{13}C NMR** (150 MHz, acetone-d_6) δ 194.0, 174.6, 172.2, 160.4, 157.9, 145.2, 141.1, 139.2, 136.9, 134.0, 130.6, 130.0, 129.3, 128.6, 128.0, 64.0, 45.6, 45.5, 30.8, 30.5, 27.7, 22.6, 21.5, 18.8. **IR** ν_{max} (film): 2957 2870 1732 1657 1456 1338 1165 1097 979 814 749 664 557 cm^{-1}. **HRMS** (ESI) m/z calcd for $C_{26}H_{33}NNaO_4S$ [M+Na]$^+$: 478.2023; found: 478.2029.

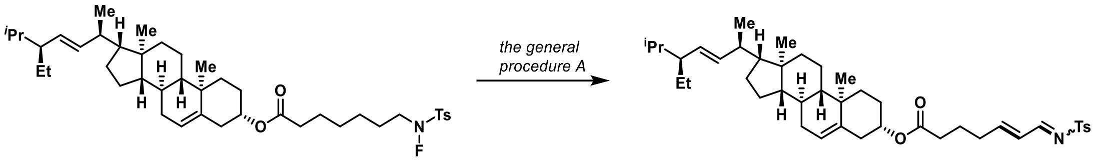

45s **45**

Product **45** was prepared by the general procedure A. Purification using column chromatography (PE/EA = 10:1) afforded **45** as yellow oil (100.7 mg, 0.15 mmol, 73%, *E/Z* = 19:1). **^{1}H NMR** (600 MHz, CD_2Cl_2) δ 9.50 (d, J = 7.8 Hz, 0.05H), 8.56 (d, J = 9.0 Hz, 0.95H), 7.78 (d, J = 8.4 Hz, 2H), 7.35 (d, J = 8.4 Hz, 2H), 6.88 (dt, J = 15.6, 6.6 Hz, 0.95H), 6.83 (dt, J = 15.6, 6.6 Hz, 0.05H), 6.37 (dd, J = 15.6, 9.0 Hz, 0.95H), 6.11 (dd, J = 15.6, 7.8 Hz, 0.05H), 5.39 - 5.38 (m, 1H), 5.19 (dd, J = 15.6, 9.0 Hz, 1H), 5.06 (dd, J = 15.6, 9.0 Hz, 1H), 4.60 - 4.55 (m, 1H), 2.43 (s, 3H), 2.39 (dt, J = 15.6, 7.2 Hz, 2H), 2.32 - 2.29 (m, 4H), 2.10 - 2.05 (m, 1H), 2.03 - 1.96 (m, 2H), 1.89 - 1.82 (m, 4H), 1.75 - 1.71 (m, 1H), 1.60 - 1.53 (m, 6H), 1.50 - 1.42 (m, 3H), 1.31 - 1.28 (m, 1H), 1.24 - 1.16 (m, 4H), 1.12 - 1.09 (m, 1H), 1.05 (d, J = 6.6 Hz, 3H), 1.03 (s, 3H), 1.02 - 0.92 (m, 2H), 0.87 (d, J = 6.0 Hz, 3H), 0.82 (d, J = 7.2 Hz, 6H), 0.73 (s, 3H). **^{13}C NMR** (150 MHz, CD_2Cl_2) δ 193.9, 172.4, 171.5, 159.6, 157.6, 145.0, 140.2, 138.8, 135.9, 133.8, 130.2, 129.8, 129.3, 128.3, 123.0, 74.4, 57.3, 56.5, 51.7, 50.6, 42.7, 41.0, 40.1, 38.6, 37.5, 37.0, 34.0, 33.2, 32.38, 32.35, 32.33, 29.4,

28.2, 25.9, 24.8, 23.6, 21.8, 21.53, 21.49, 21.3, 19.6, 19.3, 12.5, 12.3. **IR** ν_{max} (film): 2955 2868 1732 1637 1458 1316 1158 1090 972 810 738 679 554 cm^{-1}. **HRMS** (ESI) m/z calcd for $C_{43}H_{63}NNaO_4S$ $[M+Na]^+$: 712.4370; found: 712.4372.

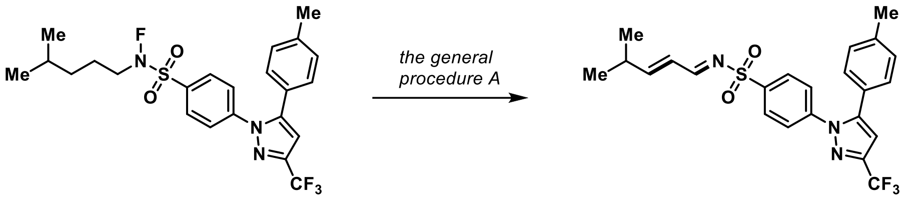

46s **46**

Product **46** was prepared by the general procedure A. Purification using column chromatography (PE/EA = 10:1) afforded **46** as yellow oil (61.8 mg, 0.13 mmol, 67%, *E/Z* > 20:1). **^{1}H NMR** (600 MHz, acetone-*d*$_6$) δ 8.54 (d, *J* = 9.6 Hz, 1H), 7.84 (d, *J* = 8.4 Hz, 2H), 7.49 (d, *J* = 8.4 Hz, 2H), 7.12 (d, *J* = 8.4 Hz, 2H), 7.10 (d, *J* = 8.4 Hz, 2H), 7.02 (dd, *J* = 15.6, 6.6 Hz, 1H), 6.86 (s, 1H), 6.21 (dd, *J* = 15.6, 9.6 Hz, 1H), 2.55 - 2.49 (m, 1H), 2.21 (s, 3H), 0.97 (d, *J* = 6.6 Hz, 6H). **^{13}C NMR** (150 MHz, acetone-*d*$_6$) δ 173.9, 168.9, 146.5, 144.2 (q, *J* = 37.5 Hz), 144.1, 140.5, 139.5, 130.4, 129.8, 129.7, 126.80, 126.78, 126.4, 122.4 (q, *J* = 265.5 Hz), 107.1, 107.0, 33.0, 21.3, 21.2. **^{19}F NMR** (565 MHz, acetone-*d*$_6$) δ -62.86. **IR** ν_{max} (film): 2969 2875 1731 1633 1449 1329 1161 1096 975 786 647 568 cm^{-1}. **HRMS** (ESI) m/z calcd for $C_{23}H_{22}F_3N_3NaO_2S$ $[M+Na]^+$: 484.1277; found: 484.1268.

The general procedure B (for products 18, 30, 31, 32a, 32b):

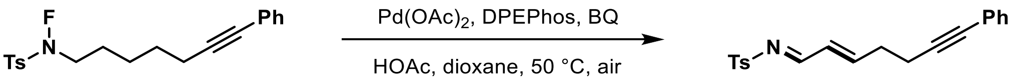

18s **18**

To a dry Schlenk flask were added **18s** (71.8 mg, 0.20 mmol, 1.0 equiv), $Pd(OAc)_2$ (4.5 mg, 0.02 mmol, 0.10 equiv), DPEPhos (11.8 mg, 0.022 mmol, 0.11 equiv), BQ (21.6 mg, 0.20 mmol, 1.0 equiv), anhydrous dioxane (1.0 mL), and HOAc (11 µL, 0.20 mmol, 1.0 equiv). The mixture was stirred at 50 °C (oil bath) for 30 min under air. Once completion, the reaction was cooled to room temperature. The reaction mixture was filtered by celite, and the filtrate was concentrated *in vacuo*. Further purification by a flash column chromatography using eluents (PE/EA = 20:1) afforded the desired product **18** as yellow oil (36.4 mg, 0.11 mmol, 54%, *E/Z* > 20:1). **^{1}H NMR** (600 MHz, acetone-*d*$_6$) δ 8.67 (d, *J* = 9.0 Hz, 1H), 7.79 (d, *J* = 8.4 Hz, 2H), 7.42 (d, *J* = 8.4 Hz, 2H), 7.37 - 7.35 (m, 2H), 7.32 - 7.29 (m, 3H), 7.25 - 7.20 (m, 1H), 6.51 (dd, *J* = 15.6, 9.0 Hz, 1H), 2.70

- 2.68 (m, 4H), 2.44 (s, 3H). **^{13}C NMR** (150 MHz, acetone-d_6) δ 172.3, 159.3, 145.4, 136.9, 132.3, 130.7, 130.0, 129.2, 128.74, 128.67, 124.6, 89.2, 82.4, 33.2, 21.5, 18.5. **IR** ν_{max} (film): 2955 2913 2239 1690 1639 1586 1321 1153 1090 964 815 692 676 552 cm^{-1}. **HRMS** (ESI) m/z calcd for $C_{20}H_{19}NNaO_2S$ [M+Na]$^+$: 360.1029; found: 360.1038.

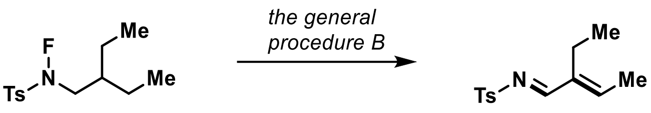

30s **30**

Product **30** was prepared by the general procedure B except that the reaction was run at 70 °C (oil bath) for 30 min. Purification using column chromatography (PE/EA = 20:1) afforded **30** as yellow oil (26.1 mg, 0.10 mmol, 52%, *E/Z* > 20:1). **^{1}H NMR** (600 MHz, acetone-d_6) δ 8.49 (s, 1H), 7.79 (d, J = 8.4 Hz, 2H), 7.43 (d, J = 8.4 Hz, 2H), 6.81 (q, J = 7.2 Hz, 1H), 2.44 (s, 3H), 2.37 (q, J = 7.2 Hz, 2H), 2.02 (d, J = 7.2 Hz, 3H), 0.93 (t, J = 7.2 Hz, 3H). **^{13}C NMR** (150 MHz, acetone-d_6) δ 174.3, 152.9, 145.1, 142.6, 137.4, 130.6, 128.5, 21.5, 18.8, 15.4, 13.1. **IR** ν_{max} (film): 2966 2878 1624 1568 1276 1103 1081 904 829 765 703 664 538 cm^{-1}. **HRMS** (ESI) m/z calcd for $C_{13}H_{17}NNaO_2S$ [M+Na]$^+$: 274.0872; found: 274.0882.

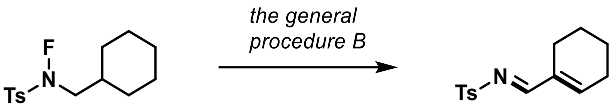

31s **31**

Product **31** was prepared by the general procedure B except that the reaction was run at 70 °C (oil bath) for 30 min. Purification using column chromatography (PE/EA = 20:1) afforded **31** as yellow oil (29.5 mg, 0.11 mmol, 56%, *E/Z* > 20:1). **^{1}H NMR** (600 MHz, acetone-d_6) δ 8.52 (s, 1H), 7.78 (d, J = 8.4 Hz, 2H), 7.43 (d, J = 8.4 Hz, 2H), 7.05 - 7.03 (m, 1H), 2.43 (s, 3H), 2.40 - 2.37 (m, 2H), 2.21 - 2.18 (m, 2H), 1.66 - 1.64 (m, 4H). **^{13}C NMR** (150 MHz, acetone-d_6) δ 173.3, 155.0, 145.2, 137.8, 137.3, 130.6, 128.6, 27.9, 23.4, 22.4, 22.1, 21.5. **IR** ν_{max} (film): 2946 2867 1627 1567 1318 1156 1089 930 807 777 697 654 568 cm^{-1}. **HRMS** (ESI) m/z calcd for $C_{14}H_{17}NNaO_2S$ [M+Na]$^+$: 286.0872; found: 286.0873.

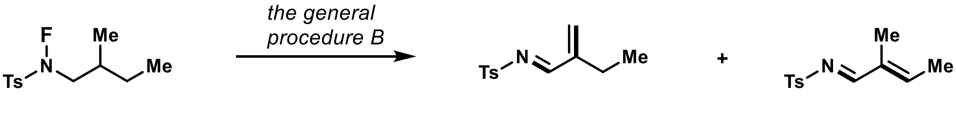

32s **32a** **32b**

Product **32a** and **32b** were prepared by the general procedure B except that the reaction was run at 70 °C (oil bath) for 30 min. Purification using column chromatography (PE/EA = 20:1) afforded **32a** as yellow oil (5.7 mg, 0.02 mmol, 12%, *E/Z* > 20:1) and **32b** as yellow oil (23.7 mg, 0.10 mmol, 50%, *E/Z* > 20:1).

32a: **^{1}H NMR** (600 MHz, acetone-d_6) δ 8.65 (s, 1H), 7.81 (d, *J* = 7.8 Hz, 2H), 7.45 (d, *J* = 7.8 Hz, 2H), 6.23 (s, 1H), 6.22 (s, 1H), 2.45 (s, 3H), 2.32 (q, *J* = 7.2 Hz, 2H), 1.05 (t, *J* = 7.2 Hz, 3H). **^{13}C NMR** (150 MHz, acetone-d_6) δ 173.3, 148.6, 145.5, 136.7, 135.9, 130.8, 128.7, 23.4, 21.5, 12.4. **IR** ν_{max} (film): 3065 2963 2927 1625 1574 1320 1163 1090 948 827 767 705 672 557 cm^{-1}. **HRMS** (ESI) m/z calcd for $C_{12}H_{15}NNaO_2S$ [M+Na]$^+$: 260.0716; found: 260.0718.

32b: **^{1}H NMR** (600 MHz, acetone-d_6) δ 8.54 (s, 1H), 7.79 (d, *J* = 8.4 Hz, 2H), 7.43 (d, *J* = 8.4 Hz, 2H), 6.85 (q, *J* = 7.2 Hz, 1H), 2.44 (s, 3H), 2.00 (d, *J* = 7.2 Hz, 3H), 1.80 (s, 3H). **^{13}C NMR** (150 MHz, acetone-d_6) δ 174.8, 153.1, 145.2, 137.4, 136.7, 130.7, 128.6, 21.5, 15.7, 10.6. **IR** ν_{max} (film): 3064 2962 2926 1627 1564 1313 1162 1087 947 838 788 704 672 555 cm^{-1}. **HRMS** (ESI) m/z calcd for $C_{12}H_{15}NNaO_2S$ [M+Na]$^+$: 260.0716; found: 260.0721.

Part 3.3. Procedure and characteristic data for compound **47-61**

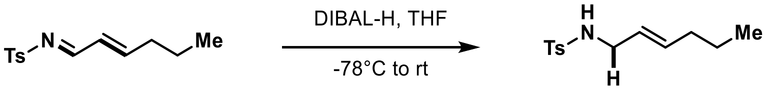

2 **47**

To a stirred solution of the imine **2** (50.2 mg, 0.20 mmol, 1.0 equiv) in anhydrous THF (4.0 mL) at -78 °C was added DIBAL-H (1.0 M in toluene, 0.30 mL, 0.30 mmol, 1.5 equiv) under argon atmosphere. The resulting mixture was warmed to room temperature and stirred for 12 h. The reaction was sequentially quenched with H_2O (3.0 mL) and NaOH (10 wt% in H_2O, 3.0 mL) at 0 °C. The resulting mixture was warmed to room temperature and stirred for 15 min, then extracted with Et_2O (10 mL × 3). The combined organic layers were washed with brine (10 mL), then dried over anhydrous Na_2SO_4, and filtered. The solvent was removed under reduced pressure, and the residue was purified by a flash column chromatography using eluents (PE/EA = 10:1) to afford the desired compound **47** as colourless oil (43.0 mg, 0.17 mmol, 85%). **^{1}H NMR** (600 MHz, $CDCl_3$) δ 7.75 (d, *J* = 8.4 Hz, 2H), 7.31 (d, *J* = 8.4 Hz, 2H), 5.54 (dt, *J* = 15.0, 6.6 Hz, 1H), 5.31 (dt, *J* = 15.0, 6.6 Hz, 1H), 4.41 (t, *J* = 6.6 Hz, 1H), 3.52 (t, *J* = 6.6 Hz, 2H), 2.43 (s, 3H), 1.91 (dt, *J* = 15.0, 7.2 Hz, 2H), 1.32 - 1.29 (m, 2H), 0.84 (t, *J* = 7.2 Hz, 3H). **^{13}C NMR** (150 MHz, $CDCl_3$) δ

143.5, 137.2, 135.1, 129.8, 127.3, 124.6, 45.5, 34.3, 22.2, 21.7, 13.7. **IR** ν_{max} (film): 2959 2872 1625 1567 1328 1161 1095 971 814 749 707 662 551 cm^{-1}. **HRMS** (ESI) m/z calcd for $C_{13}H_{19}NNaO_2S$ $[M+Na]^+$: 276.1029; found: 276.1020.

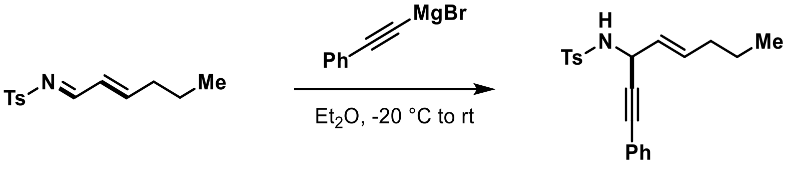

2 48

To a stirred solution of the imine **2** (50.2 mg, 0.20 mmol, 1.0 equiv) in anhydrous Et_2O (1.0 mL) at -20 °C was added (phenylethynyl)magnesium bromide[22] (0.5 M in THF, 1.2 mL, 0.60 mmol, 3.0 equiv) under argon atmosphere. The resulting mixture was warmed to room temperature and stirred for 12 h. The reaction was quenched with saturated NH_4Cl solution (5.0 mL) and extracted with Et_2O (10 mL × 3). The combined organic layers were washed with brine (10 mL), then dried over anhydrous Na_2SO_4, and filtered. The solvent was removed under reduced pressure, and the residue was purified by a flash column chromatography using eluents (PE/EA = 10:1) to afford the desired compound **48** as colourless oil (55.8 mg, 0.16 mmol, 79%). **^{1}H NMR** (600 MHz, $CDCl_3$) δ 7.80 (d, J = 8.4 Hz, 2H), 7.29 - 7.26 (m, 1H), 7.24 - 7.22 (m, 4H), 7.09 (d, J = 7.2 Hz, 2H), 5.94 (dt, J = 15.0, 7.2 Hz, 1H), 5.49 (dd, J = 15.0, 5.4 Hz, 1H), 4.93 - 4.91 (m, 1H), 4.71 (d, J = 7.2 Hz, 1H), 2.30 (s, 3H), 2.01 (dt, J = 15.0, 7.2 Hz, 2H), 1.39 - 1.36 (m, 2H), 0.88 (t, J = 7.2 Hz, 3H). **^{13}C NMR** (150 MHz, $CDCl_3$) δ 143.6, 137.7, 134.8, 131.7, 129.6, 128.6, 128.2, 127.7, 126.1, 122.3, 86.2, 85.4, 47.8, 34.0, 22.1, 21.5, 13.8. **IR** ν_{max} (film): 2961 2878 1621 1564 1335 1152 1042 967 794 760 691 620 558 cm^{-1}. **HRMS** (ESI) m/z calcd for $C_{21}H_{23}NNaO_2S$ $[M+Na]^+$: 376.1342; found: 376.1332.

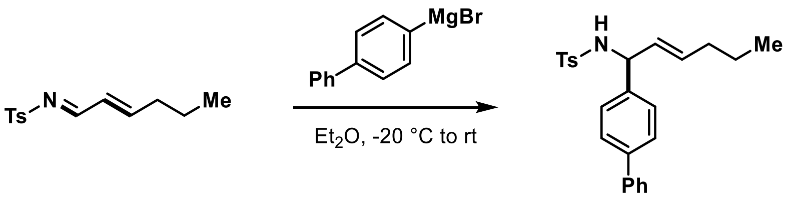

2 49

To a stirred solution of the imine **2** (50.2 mg, 0.20 mmol, 1.0 equiv) in anhydrous Et_2O (1.0 mL) at -20 °C was added [1,1'-biphenyl]-4-ylmagnesium bromide (0.5 M in THF, 1.2 mL, 0.60 mmol, 3.0 equiv) under argon atmosphere. The resulting mixture was warmed to room temperature and stirred for 12 h. The reaction was quenched with saturated NH_4Cl solution (5.0 mL) and extracted with Et_2O (10 mL × 3). The combined organic layers were washed with brine (10 mL), then dried

over anhydrous Na_2SO_4, and filtered. The solvent was removed under reduced pressure, and the residue was purified by a flash column chromatography using eluents (PE/EA = 10:1) to afford the desired compound **49** as colourless oil (64.8 mg, 0.16 mmol, 80%). **^{1}H NMR** (600 MHz, $CDCl_3$) δ 7.63 (d, J = 8.4 Hz, 2H), 7.51 (d, J = 7.8 Hz, 2H), 7.43 - 7.40 (m, 4H), 7.35 - 7.32 (m, 1H), 7.20 (d, J = 8.4 Hz, 2H),7.16 (d, J = 7.8 Hz, 2H), 5.52 - 5.42 (m, 2H), 5.07 (d, J = 7.2 Hz, 1H), 4.96 - 4.94 (m, 1H), 2.34 (s, 3H), 1.89 (dt, J = 13.8, 6.6 Hz, 2H), 1.29 - 1.25 (m, 2H), 0.82 (t, J = 7.2 Hz, 3H). **^{13}C NMR** (150 MHz, $CDCl_3$) δ 143.2, 140.7, 140.6, 139.4, 138.0, 133.9, 129.4, 129.0, 128.9, 127.6, 127.5, 127.4, 127.3, 127.1, 59.5, 34.3, 22.1, 21.6, 13.8. **IR** ν_{max} (film): 2962 2872 1624 1563 1335 1161 1046 968 813 764 698 618 547 cm^{-1}. **HRMS** (ESI) m/z calcd for $C_{25}H_{27}NNaO_2S$ $[M+Na]^+$: 428.1655; found: 428.1644.

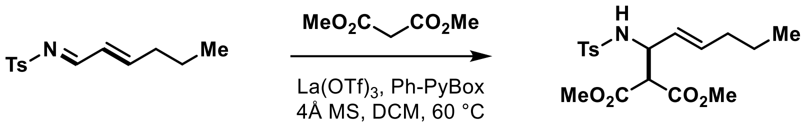

2 50

To a dry Schlenk flask were added the imine **2** (50.2 mg, 0.20 mmol, 1.0 equiv), dimethyl malonate (69 μL, 0.60 mmol, 3.0 equiv), $La(OTf)_3$ (11.7 mg, 0.02 mmol, 0.10 equiv), 2,6-bis[(4S)-4- phenyl-4,5-dihydro-1,3-oxazol-2-yl]pyridine (Ph-PyBox, 7.4 mg, 0.02 mmol, 0.10 equiv), 4Å MS (20 mg), and anhydrous DCM (2.0 mL). The mixture was stirred at 60 °C (oil bath) for 12 h under nitrogen atmosphere. Once completion, the reaction was cooled to room temperature. The reaction mixture was filtered by celite, and the filtrate was concentrated *in vacuo.* Further purification by a flash column chromatography using eluents (PE/EA = 10:1) afforded the desired compound **50** as yellow oil (55.2 mg, 0.14 mmol, 72%). **^{1}H NMR** (600 MHz, $CDCl_3$) δ 7.70 (d, J = 8.4 Hz, 2H), 7.26 (d, J = 8.4 Hz, 2H), 5.62 - 5.61 (m, 1H), 5.41 (dt, J = 15.0, 7.2 Hz, 1H), 5.27 (dd, J = 15.0, 7.2 Hz, 1H), 4.43 - 4.39 (m, 1H), 3.71 (s, 3H), 3.65 (s, 3H), 3.64 (d, J = 4.8 Hz, 1H), 2.40 (s, 3H), 1.76 (dt, J = 15.0, 6.6 Hz, 2H), 1.19 - 1.14 (m, 2H), 0.76 (t, J = 7.2 Hz, 3H). **^{13}C NMR** (150 MHz, $CDCl_3$) δ 168.1, 167.4, 143.3, 138.3, 135.2, 129.6, 127.3, 125.9, 56.6, 55.4, 52.8, 52.7, 34.1, 21.9, 21.6, 13.6. **IR** ν_{max} (film): 2959 2874 1743 1665 1563 1339 1165 1096 971 814 748 700 666 539 cm^{-1}. **HRMS** (ESI) m/z calcd for $C_{18}H_{25}NNaO_6S$ $[M+Na]^+$: 406.1295; found: 406.1304.

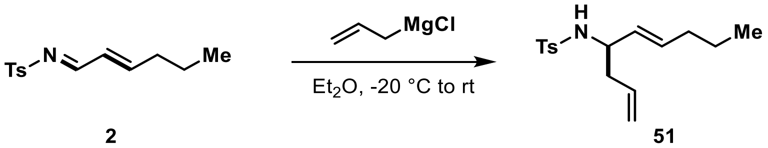

2 51

To a stirred solution of the imine **2** (50.2 mg, 0.20 mmol, 1.0 equiv) in anhydrous Et_2O (1.0 mL) at -20 °C was added allylmagnesium chloride (1.0 M in THF, 0.60 mL, 0.60 mmol, 3.0 equiv) under argon atmosphere. The resulting mixture was warmed to room temperature and stirred for 12 h. The reaction was quenched with saturated NH_4Cl solution (5.0 mL) and extracted with Et_2O (10 mL × 3). The combined organic layers were washed with brine (10 mL), then dried over anhydrous Na_2SO_4, and filtered. The solvent was removed under reduced pressure, and the residue was purified by a flash column chromatography using eluents (PE/EA = 10:1) to afford the desired compound **51** as colourless oil (48.1 mg, 0.16 mmol, 82%). **^{1}H NMR** (600 MHz, $CDCl_3$) δ 7.72 (d, *J* = 8.4 Hz, 2H), 7.27 (d, *J* = 8.4 Hz, 2H), 5.64 - 5.57 (m, 1H), 5.39 (dt, *J* = 15.6, 6.6 Hz, 1H), 5.13 (dd, *J* = 15.6, 6.6 Hz, 1H), 5.07 - 5.02 (m, 2H), 4.61 (d, *J* = 7.2 Hz, 1H), 3.88 - 3.79 (m, 1H), 2.41 (s, 3H), 2.25 - 2.19 (m, 2H), 1.82 (dt, *J* = 15.6, 7.2 Hz, 2H), 1.26 - 1.20 (m, 2H), 0.80 (t, *J* = 7.2 Hz, 3H). **^{13}C NMR** (150 MHz, $CDCl_3$) δ 143.2, 138.3, 133.3, 132.9, 129.5, 129.0, 127.4, 119.0, 55.2, 40.5, 34.2, 22.1, 21.6, 13.7. **IR** ν_{max} (film): 2962 2873 1625 1566 1338 1163 1051 968 814 749 664 576 548 cm^{-1}. **HRMS** (ESI) m/z calcd for $C_{16}H_{23}NNaO_2S$ $[M+Na]^+$: 316.1342; found: 316.1331.

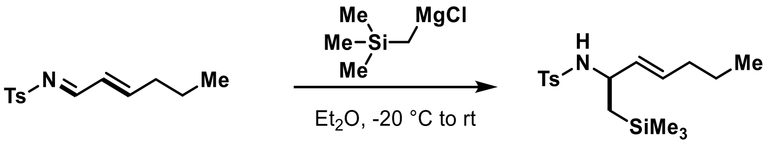

2 **52**

To a stirred solution of the imine **2** (50.2 mg, 0.20 mmol, 1.0 equiv) in anhydrous Et_2O (1.0 mL) at -20 °C was added [(trimethylsilyl)methyl]magnesium chloride (1.3 M in THF, 0.46 mL, 0.60 mmol, 3.0 equiv) under argon atmosphere. The resulting mixture was warmed to room temperature and stirred for 12 h. The reaction was quenched with saturated NH_4Cl solution (5.0 mL) and extracted with Et_2O (10 mL × 3). The combined organic layers were washed with brine (10 mL), then dried over anhydrous Na_2SO_4, and filtered. The solvent was removed under reduced pressure, and the residue was purified by a flash column chromatography using eluents (PE/EA = 10:1) to afford the desired compound **52** as colourless oil (57.7 mg, 0.17 mmol, 85%). **^{1}H NMR** (600 MHz, $CDCl_3$) δ 7.85 (d, *J* = 8.4 Hz, 2H), 7.40 (d, *J* = 8.4 Hz, 2H), 5.45 (dt, *J* = 15.6, 6.6 Hz, 1H), 5.14 (dd, *J* = 15.6, 7.2 Hz, 1H), 4.67 (d, *J* = 7.2 Hz, 1H), 4.04 - 3.99 (m, 1H), 2.55 (s, 3H), 1.87 (dt, *J* = 15.6, 7.2 Hz, 2H), 1.34 - 1.30 (m, 2H), 1.05 (dd, *J* = 14.4, 6.0 Hz, 1H), 0.98 - 0.93 (m, 4H), 0.11 (s, 9H). **^{13}C NMR** (150 MHz, $CDCl_3$) δ 143.0, 138.7, 132.0, 131.4, 129.4, 127.4, 54.5, 34.1, 25.8, 22.0, 21.6, 13.9, -0.8. **IR** ν_{max} (film): 2958 2877 1626 1565 1339 1153 1097 967 813 750 662 575 546 cm^{-1}. **HRMS** (ESI) m/z calcd for $C_{17}H_{29}NNaO_2SSi$ $[M+Na]^+$: 362.1580; found: 362.1580.

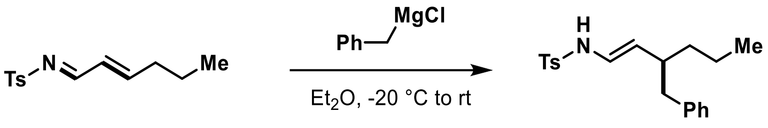

2 **53**

To a stirred solution of the imine **2** (50.2 mg, 0.20 mmol, 1.0 equiv) in anhydrous Et_2O (1.0 mL) at -20 °C was added benzylmagnesium chloride (1.3 M in THF, 0.46 mL, 0.60 mmol, 3.0 equiv) under argon atmosphere. The resulting mixture was warmed to room temperature and stirred for 12 h. The reaction was quenched with saturated NH_4Cl solution (5.0 mL) and extracted with Et_2O (10 mL × 3). The combined organic layers were washed with brine (10 mL), then dried over anhydrous Na_2SO_4, and filtered. The solvent was removed under reduced pressure, and the residue was purified by a flash column chromatography using eluents (PE/EA = 10:1) to afford the desired compound **53** as colourless oil (52.2 mg, 0.15 mmol, 76%). **^{1}H NMR** (500 MHz, acetone-*d*$_6$) δ 7.62 (d, *J* = 8.0 Hz, 2H), 7.36 (d, *J* = 8.0 Hz, 2H), 7.20 (t, *J* = 7.0 Hz, 2H), 7.14 (t, *J* = 7.0 Hz, 1H), 7.06 (d, *J* = 7.0 Hz, 2H), 5.93 (d, *J* = 14.0 Hz, 1H), 4.86 (dd, *J* = 14.0, 9.5 Hz, 1H), 2.61 (dd, *J* = 13.5, 6.0 Hz, 1H), 2.47 (dd, *J* = 13.5, 8.0 Hz, 1H), 2.42 (s, 3H), 2.27 - 2.22 (m, 1H), 1.35 - 1.31 (m, 1H), 1.21- 1.15 (m, 1H), 1.14 - 1.08 (m, 2H), 0.78 (t, *J* = 7.0 Hz, 3H). **^{13}C NMR** (125 MHz, acetone-*d*$_6$) δ 143.9, 141.3, 138.8, 130.4, 130.0, 128.8, 127.5, 126.5, 125.1, 118.7, 43.1, 42.6, 37.9, 21.4, 20.9, 14.2. **IR** ν_{max} (film): 2060 2873 1667 1564 1339 1164 1097 902 813 749 701 664 534 cm^{-1}. **HRMS** (ESI) m/z calcd for $C_{20}H_{25}NNaO_2S$ $[M+Na]^+$: 366.1498; found: 366.1503.

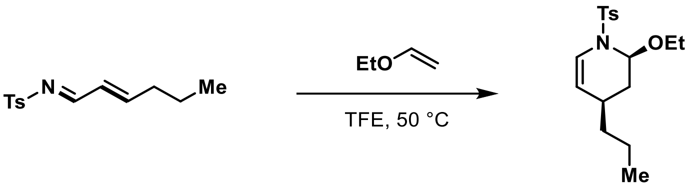

2 **54**

To a dry Schlenk flask were added the imine **2** (50.2 mg, 0.20 mmol, 1.0 equiv), ethoxyethene (0.19 mL, 2.0 mmol, 10 equiv), and trifluoroethanol (TFE, 0.20 mL). The mixture was stirred at 50 °C (oil bath) for 24 h under nitrogen atmosphere. Once completion, the reaction was cooled to room temperature. The solvent was removed under reduced pressure, and the residue was purified by a flash column chromatography using eluents (PE/EA = 10:1) to afford the desired compound **54** as colourless oil (50.4 mg, 0.16 mmol, 78%, *dr* = 13:1). **^{1}H NMR** (600 MHz, $CDCl_3$) δ 7.65 (d, *J* = 8.4 Hz, 2H), 7.28 (d, *J* = 8.4 Hz, 2H), 6.46 (dt, *J* = 8.4, 1.8 Hz, 0.93H), 5.22 - 5.26 (m, 2H), 5.01 (dt, *J* = 8.4, 1.8 Hz, 0.07H), 3.83 - 2.72 (m, 1H), 3.65 - 3.60 (m, 0.07H), 3.55 - 3.50 (m, 0.93H), 2.41 (s, 0.21H), 2.40 (s, 2.79H), 1.89 - 1.82 (m, 2H), 1.56 - 1.50 (m, 1H), 1.45 - 1.39 (m,

1H), 1.33 - 1.27 (m, 2H), 1.19 - 1.13 (m, 4H), 0.85 (t, *J* = 7.2 Hz, 3H). **^{13}C NMR** (150 MHz, $CDCl_3$) δ 143.7, 143.6, 136.7, 136.4, 129.8, 129.7, 127.00, 126.96, 121.6, 120.9, 115.6, 115.5, 82.2, 82.1, 63.3, 63.2, 37.9, 36.9, 32.4, 30.5, 29.5, 27.1, 21.6, 20.8, 20.7, 19.8, 15.1, 15.0, 14.3, 14.1. **IR** ν_{max} (film): 2958 2872 1651 1564 1339 1170 1058 917 814 750 713 653 585 cm^{-1}. **HRMS** (ESI) m/z calcd for $C_{17}H_{25}NNaO_3S$ $[M+Na]^+$: 346.1447; found: 346.1453.

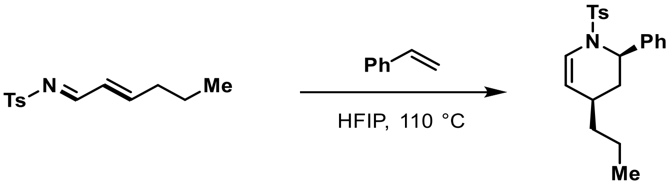

2 55

To a dry Schlenk flask were added the imine **2** (50.2 mg, 0.20 mmol, 1.0 equiv), styrene (0.23 mL, 2.0 mmol, 10 equiv), and hexafluoro-2-propanol (HFIP, 0.20 mL). The mixture was stirred at 110 °C (oil bath) for 24 h under nitrogen atmosphere. Once completion, the reaction was cooled to room temperature. The solvent was removed under reduced pressure, and the residue was purified by a flash column chromatography using eluents (PE/EA = 10:1) to afford the desired compound **55** as colourless oil (41.9 mg, 0.12 mmol, 59%, *dr* = 9:1). **^{1}H NMR** (600 MHz, $CDCl_3$) δ 7.64 - 7.61 (m, 2H), 7.27 - 7.23 (m, 6H), 7.20 - 7.18 (m, 1H), 6.86 - 6.85 (m, 1H), 5.12 (dd, *J* = 8.4, 4.2 Hz, 0.1H), 5.09 (dd, *J* = 8.4, 4.2 Hz, 0.9H), 4.93 - 4.90 (m, 1H), 2.41 (s, 2.7H), 2.40 (s, 0.3H), 1.97 (dt, *J* = 13.8, 5.4 Hz, 1H), 1.92 - 1.88 (m, 1H), 1.68 (dt, *J* = 13.8, 5.4 Hz, 1H), 1.11 - 1.05 (m, 2H), 0.83 - 0.78 (m, 1H), 0.63 (t, *J* = 7.2 Hz, 3H), 0.60 - 0.55 (m, 1H). **^{13}C NMR** (150 MHz, $CDCl_3$) δ 143.6, 143.5, 141.2, 140.8, 136.4, 136.1, 129.70, 129.67, 128.4, 128.3, 127.3, 127.12, 127.05, 127.0, 126.3, 126.0, 124.0, 123.9, 114.8, 113.5, 56.44, 56.41, 37.0, 36.8, 34.2, 33.4, 30.7, 27.4, 21.68, 21.66, 20.2, 19.8, 14.2, 13.9. **IR** ν_{max} (film): 2957 2870 1650 1562 1338 1168 1064 949 813 749 710 652 577 cm^{-1}. **HRMS** (ESI) m/z calcd for $C_{21}H_{25}NNaO_2S$ $[M+Na]^+$: 378.1498; found: 378.1504.

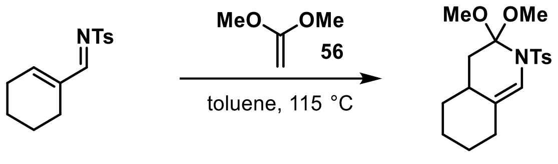

31 57m

To a dry Schlenk flask were added the imine **31** (52.6 mg, 0.20 mmol, 1.0 equiv), 1,1-dimethoxyethene **56** (88.1 mg, 2.0 mmol, 10 equiv),[23] and toluene (0.20 mL). The mixture was stirred at 115 °C (oil bath) for 12 h under nitrogen atmosphere. Once completion, the reaction

was cooled to room temperature. The solvent was removed under reduced pressure, and the residue was fast past through a short flash column chromatography (aluminium oxide basic) to afford the crude compound **57m**. **^{1}H NMR** (600 MHz, $CDCl_3$) δ 7.68 (d, J = 8.4 Hz, 2H), 7.25 (d, J = 8.4 Hz, 2H), 6.47 (s, 1H), 3.22 (s, 3H), 3.15 (s, 3H), 2.41 (s, 3H), 2.28 - 2.25 (m, 1H), 2.10 - 2.04 (m, 1H), 2.00 - 1.93 (m, 2H), 1.81 - 1.78 (m, 1H), 1.76 - 1.71 (m, 2H), 1.32 - 1.29 (m, 1H), 1.24 - 1.18 (m, 1H), 0.84 - 0.79 (m, 1H), 0.78 - 0.75 (m, 1H). **^{13}C NMR** (150 MHz, $CDCl_3$) δ 142.9, 138.5, 128.9, 127.7, 125.7, 117.4, 107.1, 49.1, 36.2, 33.9, 33.5, 31.6, 27.2, 25.7, 21.7. **IR** ν_{max} (film): 2957 2869 1650 1616 1562 1339 1238 1168 1064 949 813 749 517 cm^{-1}. **HRMS** (ESI) m/z calcd for $C_{18}H_{25}NNaO_4S$ [M+Na]$^+$: 374.1397; found: 374.1394.

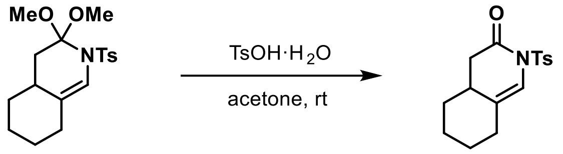

57m **57**

To a dry Schlenk flask were added the above crude **57m**, $TsOH{\cdot}H_2O$ (38.0 mg, 0.20 mmol, 1.0 equiv), and acetone (1.0 mL). The mixture was stirred at room temperature for 1 h under nitrogen atmosphere. Once completion, the solvent was removed under reduced pressure, and the residue was purified by a flash column chromatography using eluents (PE/EA = 10:1) to afford the desired compound **57** as colourless oil (38.4 mg, 0.13 mmol, 63% from **31**). **^{1}H NMR** (600 MHz, $CDCl_3$) δ 7.89 (d, J = 8.4 Hz, 2H), 7.31 (d, J = 8.4 Hz, 2H), 6.72 (s, 1H), 2.60 (dd, J = 15.6, 6.6 Hz, 1H), 2.43 (s, 3H), 2.41 - 2.34 (m, 2H), 2.26 (dd, J = 16.2, 12.0 Hz, 1H), 2.05 - 1.99 (m, 1H), 1.94 - 1.91 (m, 1H), 1.85 - 1.82 (m, 1H), 1.79 - 1.76 (m, 1H), 1.30 - 1.27 (m, 1H), 1.26 - 1.23 (m, 1H), 1.05 - 0.98 (m, 1H). **^{13}C NMR** (150 MHz, $CDCl_3$) δ 168.1, 145.1, 135.6, 129.5, 128.7, 122.7, 116.3, 40.3, 34.6, 33.3, 30.5, 26.4, 25.0, 21.8. **IR** ν_{max} (film): 2953 2870 1650 1569 1339 1165 1064 949 813 779 710 652 577 cm^{-1}. **HRMS** (ESI) m/z calcd for $C_{16}H_{19}NNaO_3S$ [M+Na]$^+$: 328.0978; found: 328.0984.

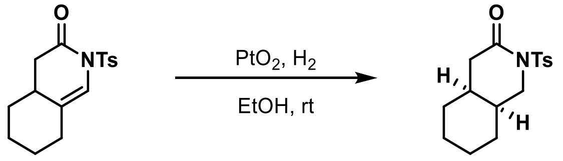

57 **58m**

To a dry Schlenk flask were added **57** (61.0 mg, 0.20 mmol, 1.0 equiv), PtO_2 (13.6 mg, 0.06 mmol, 0.30 equiv), and EtOH (12 mL). The mixture was stirred at room temperature for 12 h

under H_2 (15 Psi). Once completion, the reaction mixture was filtered by celite, and the filtrate was concentrated *in vacuo*. Further purification by a flash column chromatography using eluents (PE/EA = 7:1) afforded the desired compound **58m** as yellow oil (47.9 mg, 0.16 mmol, 78%, *dr* = 19:1). **^{1}H NMR** (600 MHz, $CDCl_3$) δ 7.89 (d, *J* = 8.4 Hz, 2H), 7.31 (d, *J* = 8.4 Hz, 2H), 4.18 (dd, *J* = 12.0, 4.8 Hz, 0.05H), 3.96 (dd, *J* = 12.0, 6.0 Hz, 0.95H), 3.81 (dd, *J* = 12.0, 6.0 Hz, 0.95H), 3.16 (dd, *J* = 12.0, 12.0 Hz, 0.05H), 2.45 - 2.35 (m, 5H), 2.13 - 2.09 (m, 1H), 2.08 - 2.05 (m, 1H), 1.65 - 1.60 (m, 1H), 1.56 - 1.48 (m, 4H), 1.45 - 1.38 (m, 2H), 1.37 - 1.34 (m, 1H). **^{13}C NMR** (150 MHz, $CDCl_3$) δ 170.3, 144.8, 136.3, 129.4, 128.8, 49.5, 36.9, 33.6, 32.2, 28.4, 26.1, 23.3, 22.1, 21.8. **IR** ν_{max} (film): 2953 2842 1646 1562 1338 1209 1168 1064 949 833 749 710 652 577 cm^{-1}. **HRMS** (ESI) m/z calcd for $C_{16}H_{21}NNaO_3S$ $[M+Na]^+$: 330.1134; found: 330.1139.

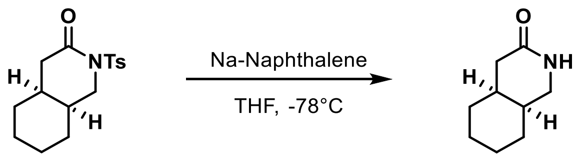

58m 58

Finely chopped sodium metal (115 mg, 3.0 mmol) and naphthalene (385 mg, 3.0 mmol) were stirred in anhydrous THF (5.0 mL) for 2 h under argon atmosphere during which a greenish black solution formed. This solution (0.6 M in THF) can be used in the next step.

To a stirred solution of compound **58m** (61.4 mg, 0.20 mmol, 1.0 equiv) in THF (2.0 mL) at -78 °C was added the above Na-naphthalene solution (0.6 M in THF, 1.0 mL, 0.60 mmol, 3.0 equiv) under argon atmosphere. The resulting mixture was stirred for 10 min at -78 °C. The reaction was quenched with H_2O (3.0 mL) and extracted with Et_2O (10 mL × 3). The combined organic layers were washed with brine (10 mL), then dried over anhydrous Na_2SO_4, and filtered. The solvent was removed under reduced pressure, and the residue was purified by a flash column chromatography using eluents ($CHCl_3$/MeOH = 50:1) to afford the desired compound **58** as colourless oil (25.4 mg, 0.17 mmol, 83%, *dr* = 19:1). **^{1}H NMR** (600 MHz, $CDCl_3$) δ 6.43 (s, 1H), 3.31 (ddd, *J* = 12.0, 5.4, 1.8 Hz, 0.94H), 3.26 (ddd, *J* = 12.0, 5.4, 1.8 Hz, 1H), 2.94 - 2.90 (m, 0.06H), 2.37 (dd, *J* = 18.0, 7.2 Hz, 1H), 2.31 (dd, *J* = 18.0, 7.2 Hz, 1H), 2.10 - 2.05 (m, 1H), 2.00 - 1.96 (m, 1H), 1.79 - 1.73 (m, 0.24H), 1.62 - 1.53 (m, 3.76H), 1.52 - 1.38 (m, 4H). **^{13}C NMR** (150 MHz, $CDCl_3$) δ 172.4, 48.3, 44.8, 38.7, 37.8, 36.9, 34.4, 33.1, 32.33, 32.31, 29.9, 28.7, 26.5, 25.58, 25.56, 23.4, 22.7. **IR** ν_{max} (film): 2960 2835 1650 1562 1339 1178 1064 949 813 749 710 652 517 cm^{-1}. **HRMS** (ESI) m/z calcd for $C_9H_{15}NNaO$ $[M+Na]^+$: 176.1046; found: 176.1049.

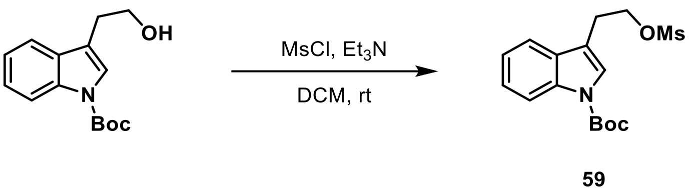

To a stirred solution of the tert-butyl 3-(2-hydroxyethyl)-1H-indole-1-carboxylate[24] (1.31 g, 5.0 mmol, 1.0 equiv) in DCM (50 mL) at 0 °C was added methanesulfonyl chloride (0.50 mL, 6.0 mmol, 1.2 equiv) and Et_3N (1.0 mL, 7.5 mmol, 1.5 equiv). The resulting mixture was stirred for 12 h at room temperature. The reaction was quenched with H_2O (10 mL) and extracted with Et_2O (10 mL × 3). The combined organic layers were washed with brine (10 mL), then dried over anhydrous Na_2SO_4, and filtered. The solvent was removed under reduced pressure, and the residue was purified by a flash column chromatography using eluents (PE/EA = 8:1) to afford the desired compound **59** as colourless oil (1.46 g, 4.3 mmol, 86%). **^{1}H NMR** (600 MHz, $CDCl_3$) δ 8.15 (s, 1H), 7.53 (d, *J* = 7.2 Hz, 1H), 7.49 (s, 1H), 7.33 (t, *J* = 7.2 Hz, 1H), 7.26 (t, *J* = 7.2 Hz, 1H), 4.48 (t, *J* = 7.2 Hz, 2H), 3.16 (t, *J* = 7.2 Hz, 2H), 2.91 (s, 3H), 1.67 (s, 9H). **^{13}C NMR** (150 MHz, $CDCl_3$) δ 149.7, 135.6, 130.1, 124.8, 124.0, 122.8, 118.7, 115.5, 115.2, 83.9, 68.9, 37.6, 28.3, 25.4. **IR** ν_{max} (film): 2957 2870 1650 1562 1338 1270 1196 1064 949 843 729 652 577 cm^{-1}. **HRMS** (ESI) m/z calcd for $C_{16}H_{21}NNaO_5S$ $[M+Na]^+$: 362.1033; found: 362.1033.

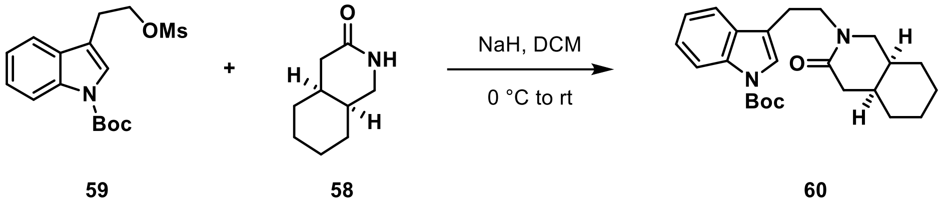

A flame dried Schlenk equipped with a stirrer bar was charged with NaH (24.0 mg, 0.60 mmol, 3.0 equiv, 60% in oil) and anhydrous DCM (0.50 mL). A solution of **58** (30.6 mg, 0.20 mmol, 1.0 equiv) in anhydrous DCM (0.50 mL) was slowly added at 0 °C. The mixture was allowed to stir for 30 min at 0 °C under nitrogen atmosphere. Then compound **59** (81.4 mg, 0.24 mmol, 1.2 equiv) in anhydrous DCM (1.0 mL) was added dropwise to the mixture and the resulting slurry was stirred for another 12 h at room temperature. The reaction was quenched by saturated $NaHCO_3$ solution (3.0 mL) at 0 °C, and then extracted with DCM (3.0 mL × 3). The combined organic layers were washed sequentially with hydrochloric acid (1.0 M) and brine, dried over anhydrous Na_2SO_4, and filtered. The solvent was removed under reduced pressure, and the residue was purified by a flash column chromatography (PE/EA = 2:1) to afford the desired compound **60** as colourless oil (54.7 mg, 0.14 mmol, 69%). **^{1}H NMR** (600 MHz, $CDCl_3$) δ 8.13 (s, 1H), 7.63 (d, *J* = 7.2 Hz, 1H), 7.41 (s, 1H),

7.31 (t, *J* = 7.2 Hz, 1H), 7.25 (t, *J* = 7.2 Hz, 1H), 3.76 - 3.71 (m, 1H), 3.54 - 3.49 (m, 1H), 3.22 - 3.19 (m, 1H), 3.12 - 3.09 (m, 1H), 2.97 (t, *J* = 7.8 Hz, 2H), 2.39 (t, *J* = 7.8 Hz, 2H), 2.03 - 2.01 (m, 1H), 1.95 - 1.93 (m, 1H), 1.66 (s, 9H), 1.50 - 1.46 (m, 4H), 1.39 - 1.36 (m, 4H). **^{13}C NMR** (150 MHz, $CDCl_3$) δ 169.5, 149.8, 130.6, 124.5, 123.4, 122.7, 119.2, 118.1, 115.4, 83.5, 51.6, 47.8, 35.3, 33.2, 32.7, 28.5, 28.4, 26.6, 23.3, 23.0, 22.8. **IR** ν_{max} (film): 2935 2870 1650 1562 1338 1270 1168 1124 949 813 749 710 693 577 cm^{-1}. **HRMS** (ESI) m/z calcd for $C_{24}H_{33}N_2O_3$ [M+H]$^+$: 397.2486; found: 397.2486.

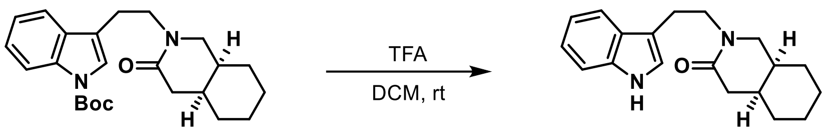

60 **61**

The compound **60** (79.2 mg, 0.20 mmol) was dissolved in DCM (2.0 mL), and then TFA (0.50 mL) was added at room temperature. The solution was stirred for 3 h until the complete consumption of compound **60** (monitored by TLC). Excess acid was quenched with saturated $NaHCO_3$ solution (~10 mL). The organic phase was collected and the aqueous solution was extracted with DCM (10 mL × 3). The combined organic fractions were dried over anhydrous $MgSO_4$, and filtered. The solvent was removed under reduced pressure, and the residue was purified by a flash column chromatography (PE/EA = 3:1) to afford the desired compound **61** as colourless oil (48.6 mg, 0.16 mmol, 82%). **^{1}H NMR** (500 MHz, $CDCl_3$) δ 7.99 (brs, 1H), 7.67 (d, *J* = 8.0 Hz, 1H), 7.36 (d, *J* = 8.0 Hz, 1H), 7.19 (t, *J* = 7.0 Hz, 1H), 7.13 (t, *J* = 7.0 Hz, 1H), 7.06 (brs, 1H), 3.76 - 3.71 (m, 1H), 3.61 - 3.56 (m, 1H), 3.21 - 3.17 (m, 1H), 3.14 - 3.10 (m, 1H), 3.05 - 3.02 (m, 2H), 2.43 - 2.33 (m, 2H), 2.03 - 2.02 (m, 1H), 1.93 - 1.90 (m, 1H), 1.48 - 1.43 (m, 4H), 1.38 - 1.34 (m, 4H). **^{13}C NMR** (125 MHz, $CDCl_3$) δ 169.9, 136.4, 127.7, 122.4, 122.2, 119.5, 118.9, 113.2, 111.3, 51.5, 48.5, 35.2, 33.1, 32.5, 28.5, 26.6, 23.2, 23.1, 22.8. **IR** ν_{max} (film): 2957 2871 1664 1562 1339 1168 1064 949 835 749 696 652 517 cm^{-1}. **HRMS** (ESI) m/z calcd for $C_{19}H_{25}N_2O$ [M+H]$^+$: 297.1961; found: 297.1969.

^1H NMR literature (chang)[25]	^1H NMR synthesized **61**	^{13}C NMR literature (chang)[25]	^{13}C NMR synthesized **61**
8.11 (brs, 1H)	7.99 (brs, 1H)	169.51	169.9
7.67 (d, *J* = 8.0 Hz, 1H)	7.67 (d, *J* = 8.0 Hz, 1H)	136.25	136.4
7.37 (d, *J* = 8.0 Hz, 1H)	7.36 (d, *J* = 8.0 Hz, 1H)	127.49	127.7
7.19 (t, *J* = 7.5 Hz, 1H)	7.19 (t, *J* = 7.0 Hz, 1H)	122.13	122.4
7.13 (t, *J* = 7.5 Hz, 1H)	7.13 (t, *J* = 7.0 Hz, 1H)	121.98	122.2
7.06 (brs, 1H)	7.06 (brs, 1H)	119.33	119.5
3.78 - 3.72 (m, 1H)	3.76 - 3.71 (m, 1H)	118.78	118.9
3.63 - 3.57 (m, 1H)	3.61 - 3.56 (m, 1H)	113.14	113.2
3.24 - 3.10 (m, 2H)	3.21 - 3.17 (m, 1H)	111.15	111.3
	3.14 - 3.10 (m, 1H)	51.21	51.5
3.07 - 3.03 (m, 2H)	3.05 - 3.02 (m, 2H)	48.22	48.5
2.46 - 2.34 (m, 2H)	2.43 - 2.33 (m, 2H)	35.17	35.2
2.04-1.96 (m, 1H)	2.03 - 2.02 (m, 1H)	32.91	33.1
1.96-1.88 (m, 1H),	1.93 - 1.90 (m, 1H)	32.41	32.5
1.59-1.28 (m, 8H)	1.48 - 1.43 (m, 4H)	28.30	28.5
	1.38 - 1.34 (m, 4H)	26.42	26.6
		23.02	23.2
		22.95	23.1
		22.61	22.8

Supplementary Figure 4. Comparison of ^1H NMR and ^{13}C NMR spectroscopic data of the literature (chang group) and our synthesized 61.

Part 3.4. Procedure and characteristic data for control experiments

Radical trapping experiments:

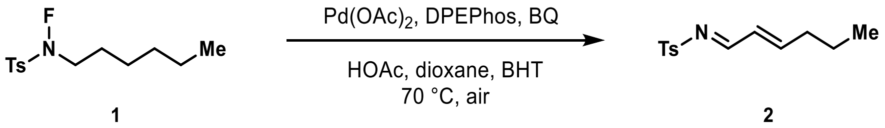

To a dry Schlenk flask were added **1** (54.6 mg, 0.20 mmol, 1.0 equiv), $Pd(OAc)_2$ (4.5 mg, 0.02 mmol, 0.10 equiv), DPEPhos (11.8 mg, 0.022 mmol, 0.11 equiv), BQ (21.6 mg, 0.20 mmol, 1.0 equiv), butylated hydroxytoluene (BHT, 88.1 mg, 0.40 mmol, 2.0 equiv), anhydrous dioxane (1.0 mL), and HOAc (11 μL, 0.20 mmol, 1.0 equiv). The mixture was stirred at 70 °C (oil bath) for 15 min under air. Once completion, the reaction was cooled to room temperature. The reaction mixture was filtered by celite, and the filtrate was concentrated *in vacuo.* Further purification by a

flash column chromatography using eluents (PE/EA = 20:1) afforded the desired product **2** as colourless oil (30.1 mg, 0.12 mmol, 60%, *E/Z* > 20:1).

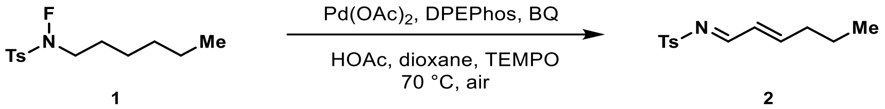

1 2

To a dry Schlenk flask were added **1** (54.6 mg, 0.20 mmol, 1.0 equiv), $Pd(OAc)_2$ (4.5 mg, 0.02 mmol, 0.10 equiv), DPEPhos (11.8 mg, 0.022 mmol, 0.11 equiv), BQ (21.6 mg, 0.20 mmol, 1.0 equiv), 2,2,6,6-tetramethylpiperidinooxy (TEMPO, 62.5 mg, 0.40 mmol, 2.0 equiv), anhydrous dioxane (1.0 mL), and HOAc (11 μL, 0.20 mmol, 1.0 equiv). The mixture was stirred at 70 °C (oil bath) for 15 min under air. Once completion, the reaction was cooled to room temperature. The reaction mixture was filtered by celite, and the filtrate was concentrated *in vacuo*. Further purification by a flash column chromatography using eluents (PE/EA = 20:1) afforded the desired product **2** as colourless oil (26.1 mg, 0.10 mmol, 52%, *E/Z* > 20:1).

The dehydrogenation reaction of imine:

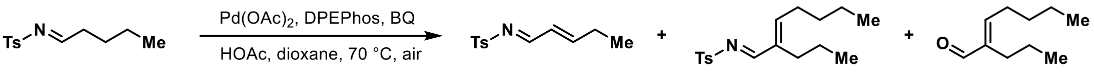

62 5 63 64

To a dry Schlenk flask were added **62**[26] (47.8 mg, 0.20 mmol, 1.0 equiv), $Pd(OAc)_2$ (4.5 mg, 0.02 mmol, 0.10 equiv), DPEPhos (11.8 mg, 0.022 mmol, 0.11 equiv), BQ (21.6 mg, 0.20 mmol, 1.0 equiv), anhydrous dioxane (1.0 mL), and HOAc (11 μL, 0.20 mmol, 1.0 equiv). The mixture was stirred at 70 °C (oil bath) for 15 min under air. Once completion, the reaction was cooled to room temperature. The reaction mixture was filtered by celite, and the filtrate was concentrated *in vacuo*. Further purification by a flash column chromatography using eluents (PE/EA = 30:1) afforded the desired product **5** as colourless oil (2.8 mg, 0.01 mmol, 6%, *E/Z* > 20:1), compound **63** (16.0 mg, 0.05 mmol, 26%, *E/Z* > 20:1), and compound **64** (3.7 mg, 0.02 mmol, 12%, *E/Z* > 20:1).

compound **63:** ^{1}**H NMR** (600 MHz, $CDCl_3$) δ 8.47 (s, 1H), 7.81 (d, *J* = 8.4 Hz, 2H), 7.31 (d, *J* = 8.4 Hz, 2H), 6.49 (t, *J* = 7.8 Hz, 1H), 2.43 (s, 3H), 2.37 - 2.31 (m, 4H), 1.49 - 1.44 (m, 2H), 1.39 - 1.35 (m, 4H), 0.92 (t, *J* = 7.2 Hz, 3H), 0.84 (t, *J* = 7.2 Hz, 3H). ^{13}**C NMR** (150 MHz, $CDCl_3$) δ 174.1, 157.7, 144.2, 139.6, 136.1, 129.8, 127.9, 30.9, 29.5, 27.4, 22.6, 21.84, 21.75, 14.1, 14.0. **IR** ν_{max} (film): 2964 2874 1651 1568 1339 1171 1058 917 807 750 712 653 543 cm^{-1}. **HRMS** (ESI) m/z calcd for $C_{17}H_{25}NNaO_2S$ $[M+Na]^+$: 330.1498; found: 330.1504.

compound **64:** ^{1}**H NMR** (600 MHz, $CDCl_3$) δ 9.37 (s, 1H), 6.45 (t, *J* = 7.2 Hz, 1H), 2.36 (dt, *J* =

15.0, 7.2 Hz, 2H), 2.22 (t, J = 7.2 Hz, 2H), 1.51 - 1.46 (m, 2H), 1.42 - 1.37 (m, 4H), 0.94 (t, J = 7.2 Hz, 3H), 0.90 (t, J = 7.2 Hz, 3H). **^{13}C NMR** (150 MHz, $CDCl_3$) δ 195.5, 155.6, 143.8, 31.0, 28.8, 26.1, 22.6, 22.1, 14.2, 14.0.

Deuterium labeling experiments:

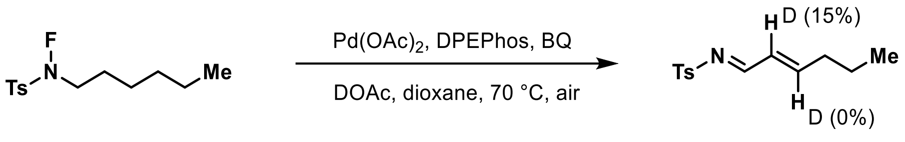

To a dry Schlenk flask were added **1** (54.6 mg, 0.20 mmol, 1.0 equiv), $Pd(OAc)_2$ (4.5 mg, 0.02 mmol, 0.10 equiv), DPEPhos (11.8 mg, 0.022 mmol, 0.11 equiv), BQ (21.6 mg, 0.20 mmol, 1.0 equiv), anhydrous dioxane (1.0 mL), and DOAc (11 μL, 0.20 mmol, 1.0 equiv). The mixture was stirred at 70 °C (oil bath) for 15 min under air. Once completion, the reaction was cooled to room temperature. The reaction mixture was filtered by celite, and the filtrate was concentrated *in vacuo.* Further purification by a flash column chromatography using eluents (PE/EA = 20:1) afforded the desired product **D-2** as colourless oil (40.2 mg, 0.16 mmol, 80%, *E/Z* > 20:1).

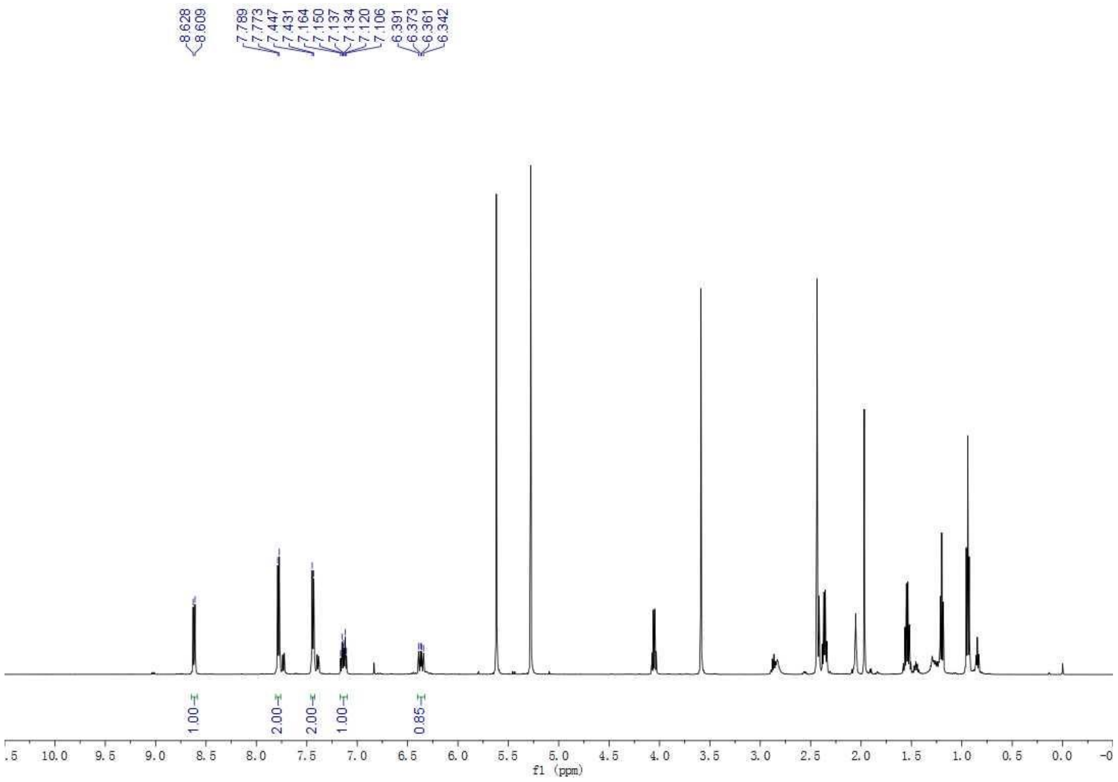

Supplementary Figure 5. ^{1}H NMR of compound D-2. The sample has been recorded in 500 MHz, acetone-d_6 at 25 °C.

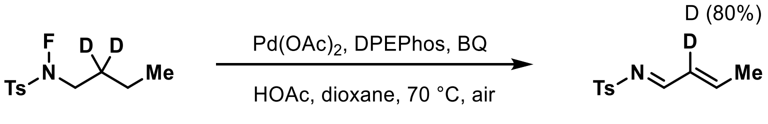

D-4s **D-4**

To a dry Schlenk flask were added **D-4s** (49.4 mg, 0.20 mmol, 1.0 equiv), $Pd(OAc)_2$ (4.5 mg, 0.02 mmol, 0.10 equiv), DPEPhos (11.8 mg, 0.022 mmol, 0.11 equiv), BQ (21.6 mg, 0.20 mmol, 1.0 equiv), anhydrous dioxane (1.0 mL), and HOAc (11 µL, 0.20 mmol, 1.0 equiv). The mixture was stirred at 70 °C (oil bath) for 15 min under air. Once completion, the reaction was cooled to room temperature. The reaction mixture was filtered by celite, and the filtrate was concentrated *in vacuo*. Further purification by a flash column chromatography using eluents (PE/EA = 20:1) afforded the desired product **D-4** as colourless oil (30.0 mg, 0.13 mmol, 67%, *E/Z* > 20:1).

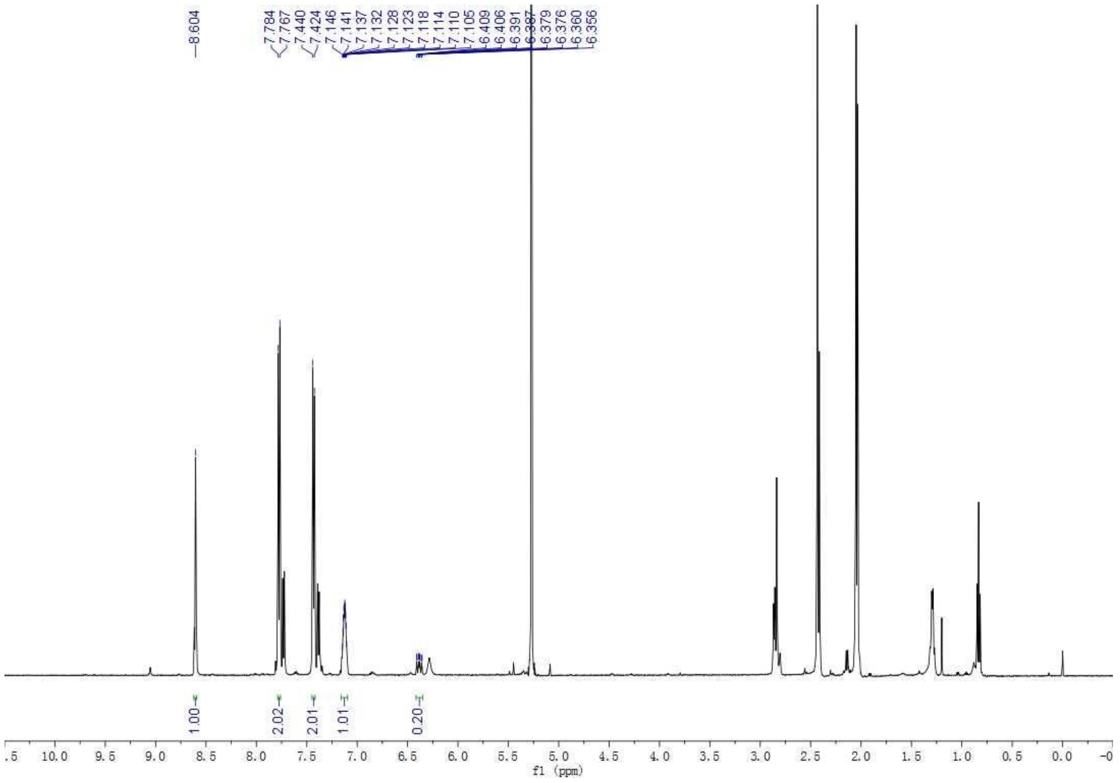

Supplementary Figure 6. ^{1}H NMR of compound D-4. The sample has been recorded in 500 MHz, acetone-d_6 at 25 °C.

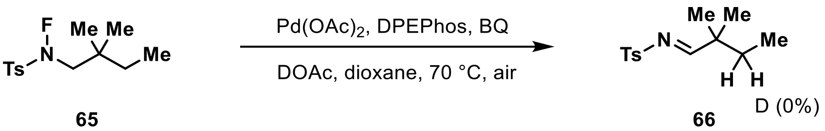

65 **66**

To a dry Schlenk flask were added **65** (54.6 mg, 0.20 mmol, 1.0 equiv), $Pd(OAc)_2$ (4.5 mg, 0.02

mmol, 0.10 equiv), DPEPhos (11.8 mg, 0.022 mmol, 0.11 equiv), BQ (21.6 mg, 0.20 mmol, 1.0 equiv), anhydrous dioxane (1.0 mL), and DOAc (11 μL, 0.20 mmol, 1.0 equiv). The mixture was stirred at 70 °C (oil bath) for 15 min under air. Once completion, the reaction was cooled to room temperature. The reaction mixture was filtered by celite, and the filtrate was concentrated *in vacuo.* Further purification by a flash column chromatography using eluents (PE/EA = 20:1) afforded the desired product **66** as colourless oil (30.9 mg, 0.12 mmol, 61%, *E/Z* > 20:1). **^{1}H NMR** (500 MHz, acetone-d_6) δ 8.42 (s, 1H), 7.78 (d, J = 8.0 Hz, 2H), 7.46 (d, J = 8.0 Hz, 2H), 2.45 (s, 3H), 1.58 (q, J = 7.5 Hz, 2H), 1.10 (s, 6H), 0.77 (t, J = 7.5 Hz, 3H). **^{13}C NMR** (125 MHz, acetone-d_6) δ 185.1, 145.6, 136.4, 130.8, 128.7, 41.7, 32.8, 23.4, 21.5, 8.9. **IR** ν_{max} (film): 2963 2874 1651 1567 1339 1163 1079 903 816 743 711 665 534 cm^{-1}. **HRMS** (ESI) m/z calcd for $C_{13}H_{19}NNaO_2S$ [M+Na]$^+$: 276.1029; found: 276.1035.

The dehydrogenation reaction of enamide:

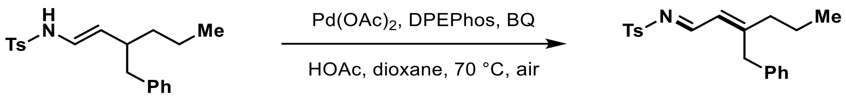

53 → 67

To a dry Schlenk flask were added **53** (68.6 mg, 0.20 mmol, 1.0 equiv), $Pd(OAc)_2$ (4.5 mg, 0.02 mmol, 0.10 equiv), DPEPhos (11.8 mg, 0.022 mmol, 0.11 equiv), BQ (21.6 mg, 0.20 mmol, 1.0 equiv), anhydrous dioxane (1.0 mL), and HOAc (11 μL, 0.20 mmol, 1.0 equiv). The mixture was stirred at 70 °C (oil bath) for 15 min under air. Once completion, the reaction was cooled to room temperature. The reaction mixture was filtered by celite, and the filtrate was concentrated *in vacuo.* Further purification by a flash column chromatography using eluents (PE/EA = 20:1) afforded the desired product **67** as colourless oil (40.9 mg, 0.12 mmol, 60%, *E/Z* = 1:1). **^{1}H NMR** (500 MHz, CD_2Cl_2) δ 9.07 (d, J = 10.0 Hz, 0.5H), 8.91 (d, J = 10.0 Hz, 0.5H), 7.78 - 7.75 (m, 2H), 7.36 - 7.29 (m, 4H), 7.27 - 7.25 (m, 1H), 7.16 - 7.13 (m, 2H), 6.34 (d, J = 10.0 Hz, 0.5H), 6.07 (d, J = 10.0 Hz, 0.5H), 3.83 (s, 1H), 3.59 (s, 1H), 2.46 - 2.43 (m, 4H), 2.21 - 2.18 (m, 1H), 1.59 - 1.55 (m, 1H), 1.53 - 1.48 (m, 1H), 0.95 (t, J = 7.5 Hz, 1.5H), 0.87 (t, J = 7.5 Hz, 1.5H). **^{13}C NMR** (125 MHz, CD_2Cl_2) δ 170.7, 169.5, 167.6, 167.2, 145.00, 144.97, 137.9, 137.5, 135.94, 135.91, 130.2, 130.1, 129.7, 129.2, 129.1, 129.0, 128.2, 128.1, 127.3, 127.2, 124.9, 124.5, 45.0, 40.5, 38.2, 34.2, 22.9, 21.8, 21.7, 21.1, 14.1, 13.9. **IR** ν_{max} (film): 2961 2872 1624 1570 1320 1157 1090 907 814 732 700 677 571 cm^{-1}. **HRMS** (ESI) m/z calcd for $C_{20}H_{23}NNaO_2S$ [M+Na]$^+$: 364.1342; found: 364.1353.

Kinetic isotope effect (KIE) studies:

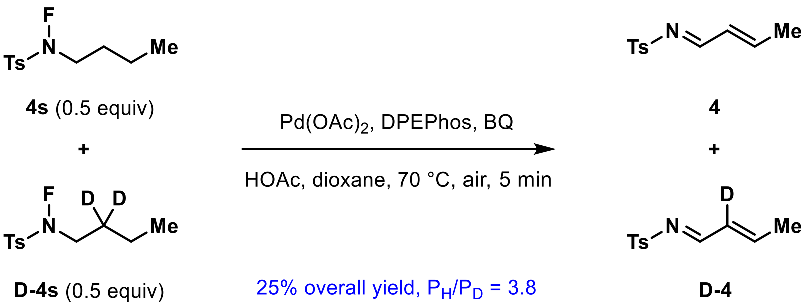

Intermolecular competition reaction: To a dry Schlenk flask were added **4s** (24.5 mg, 0.10 mmol, 0.50 equiv), **D-4s** (24.7 mg, 0.10 mmol, 0.50 equiv), $Pd(OAc)_2$ (4.5 mg, 0.02 mmol, 0.10 equiv), bis[2-(diphenylphosphino)phenyl]ether (DPEPhos, 11.8 mg, 0.022 mmol, 0.11 equiv), 1,4-benzoquinone (BQ, 21.6 mg, 0.20 mmol, 1.0 equiv), anhydrous dioxane (1.0 mL), and HOAc (11 μL, 0.20 mmol, 1.0 equiv). The mixture was stirred at 70 °C (oil bath) for 5 min under air. Once cooled to room temperature, the reaction mixture was filtered by celite, and the filtrate was concentrated *in vacuo*. Further purification by a flash column chromatography using eluents (PE/EA = 20:1) afforded a mixture of products **4** and **D-4** in 25% overall yield with a ratio of 79:21 according to ^{1}H NMR. P_H/P_D = 79/21 = 3.8

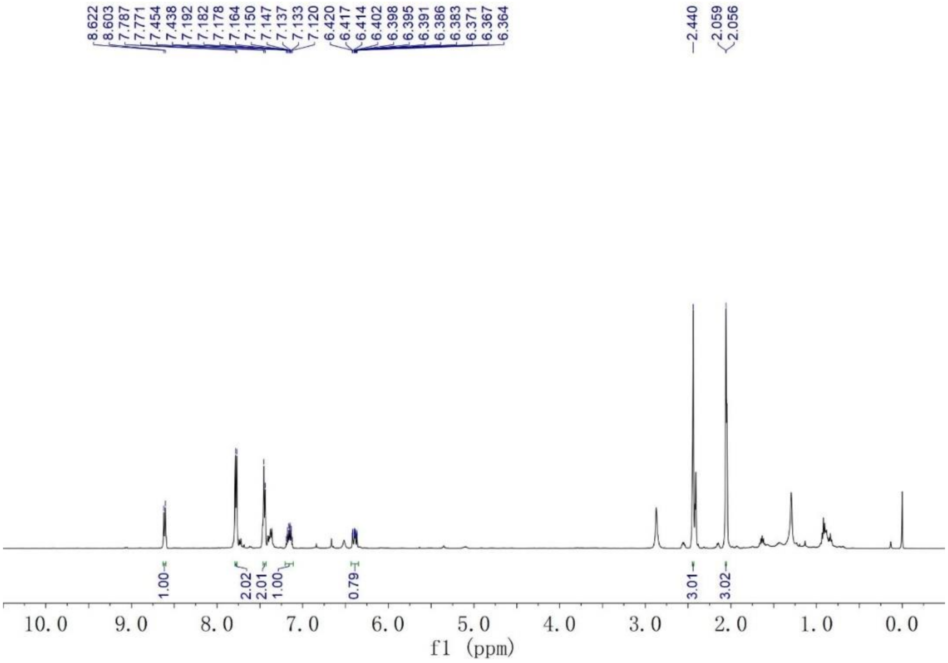

Supplementary Figure 7. ^{1}H NMR of 4 + D-4. The sample has been recorded in 500 MHz, acetone-d_6 at 25 °C.

Parallel reactions:

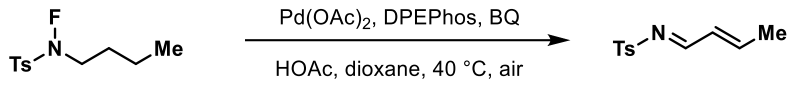

4s **4**

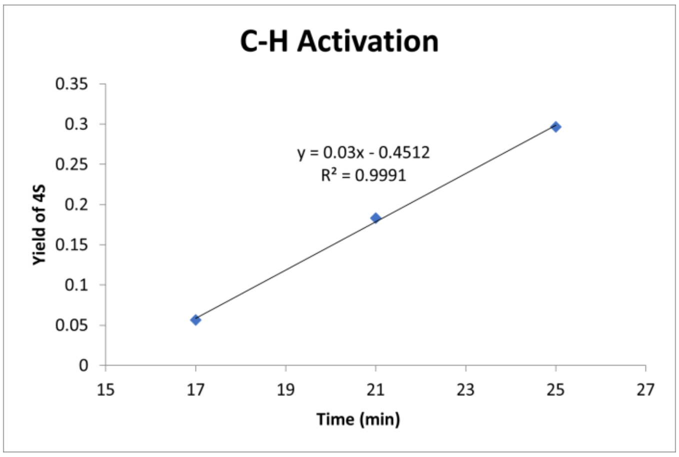

Supplementary Figure 8. C–H activation.

To a dry Schlenk flask were added **4s** (49.2 mg, 0.20 mmol, 1.0 equiv), $Pd(OAc)_2$ (4.5 mg, 0.02 mmol, 0.10 equiv), bis[2-(diphenylphosphino)phenyl]ether (DPEPhos, 11.8 mg, 0.022 mmol, 0.11 equiv), 1,4-benzoquinone (BQ, 21.6 mg, 0.20 mmol, 1.0 equiv), anhydrous dioxane (1.0 mL), and HOAc (11 µL, 0.20 mmol, 1.0 equiv). The mixture was stirred at 40 °C (oil bath) for X min under air. Once cooled to room temperature, the reaction mixture was filtered by celite, and the filtrate was concentrated *in vacuo*. Further purification by a flash column chromatography using eluents (PE/EA = 20:1) afforded the desired product **4** as colourless oil.

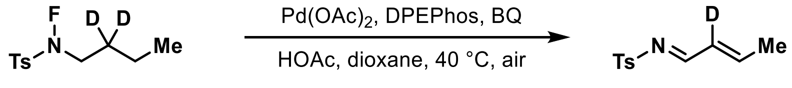

D-4s **D-4**

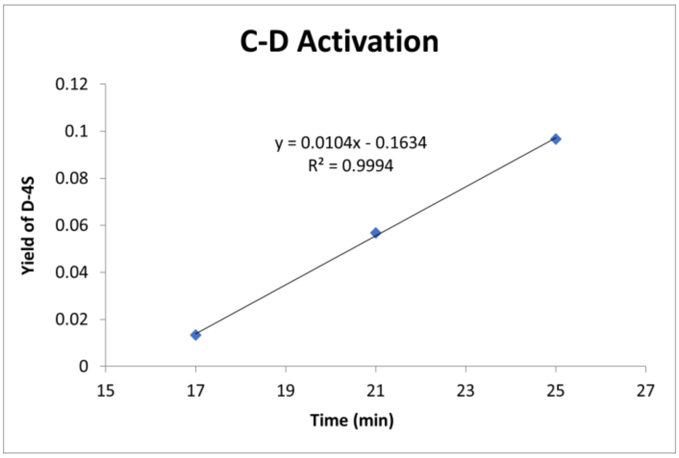

Supplementary Figure 9. C–D activation.

To a dry Schlenk flask were added **D-4s** (49.4 mg, 0.20 mmol, 1.0 equiv), $Pd(OAc)_2$ (4.5 mg, 0.02 mmol, 0.10 equiv), bis[2-(diphenylphosphino)phenyl]ether (DPEPhos, 11.8 mg, 0.022 mmol, 0.11 equiv), 1,4-benzoquinone (BQ, 21.6 mg, 0.20 mmol, 1.0 equiv), anhydrous dioxane (1.0 mL), and HOAc (11 µL, 0.20 mmol, 1.0 equiv). The mixture was stirred at 40 °C (oil bath) for X min under air. Once cooled to room temperature, the reaction mixture was filtered by celite, and the filtrate was concentrated *in vacuo*. Further purification by a flash column chromatography using eluents (PE/EA = 20:1) afforded the desired product **D-4** as colourless oil. k_H/k_D = 0.03/0.0104 = 2.9

Detection of LPd(H)(F) complex:

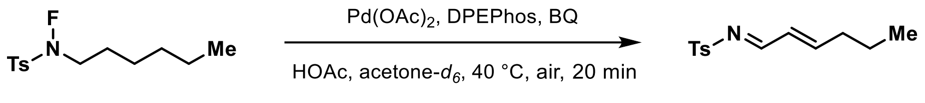

To a dry NMR tube were added **1** (13.7 mg, 0.05 mmol, 1.0 equiv), $Pd(OAc)_2$ (1.1 mg, 0.005 mmol, 0.10 equiv), bis[2-(diphenylphosphino)phenyl]ether (DPEPhos, 3.0 mg, 0.0055 mmol, 0.11 equiv), 1,4-benzoquinone (BQ, 5.4 mg, 0.05 mmol, 1.0 equiv), acetone-d_6 (0.50 mL), and HOAc (3.0 µL, 0.05 mmol, 1.0 equiv). The mixture was stirred at 40 °C (oil bath) for 20 min under air. Once completion, the reaction was cooled to room temperature. The LPd(H)(F) complex has been successfully detected by both ^{1}H (δ -6.82 ppm) and ^{19}F (δ -186.9 ppm) NMR.

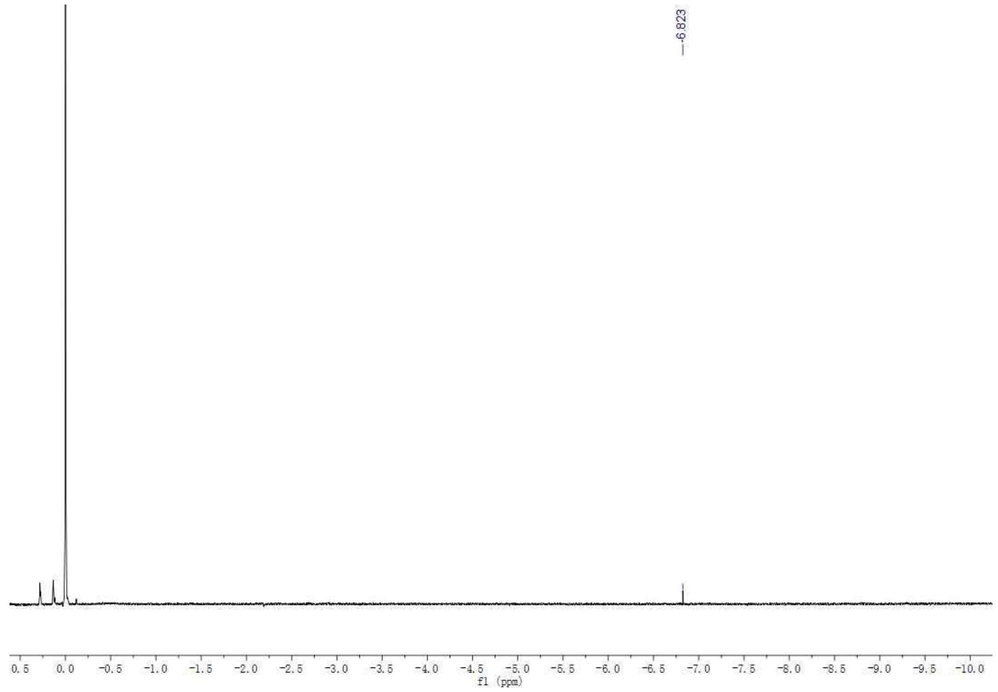

Supplementary Figure 10. ^{1}H NMR of LPd(H)(F) complex. The sample has been recorded in 500 MHz, acetone-d_6 at 25 °C.

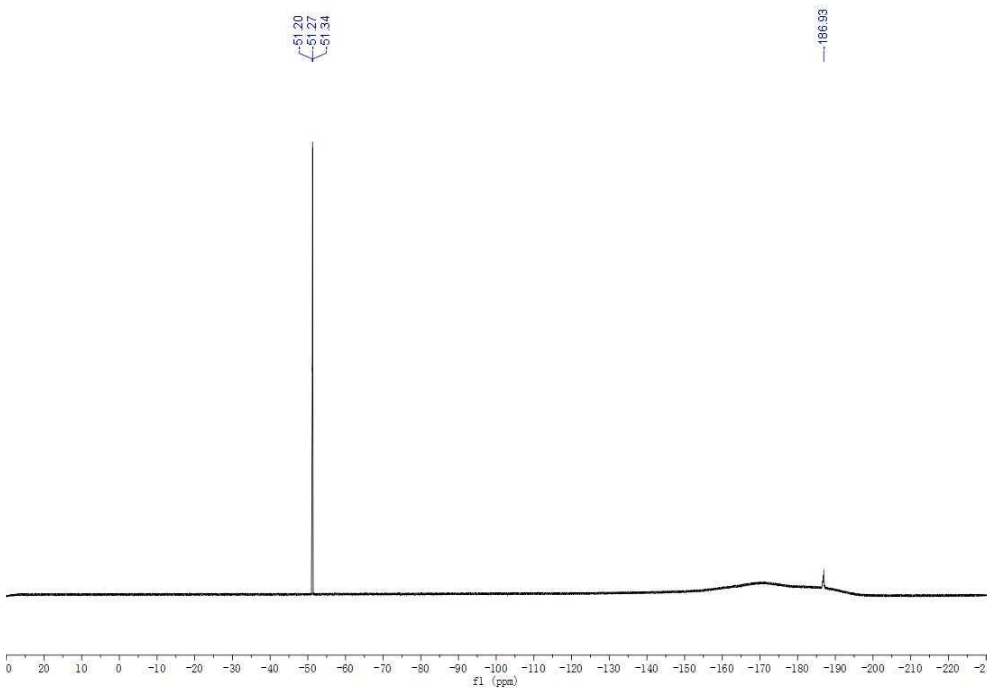

Supplementary Figure 11. ^{19}F NMR of LPd(H)(F) complex. The sample has been recorded in 565 MHz, acetone-*d_6* at 25 °C. (Note: the unreacted *N*-fluoro-sulfonamide shows a peak at δ -51.3 ppm)

Part 3.5. NMR spectra.

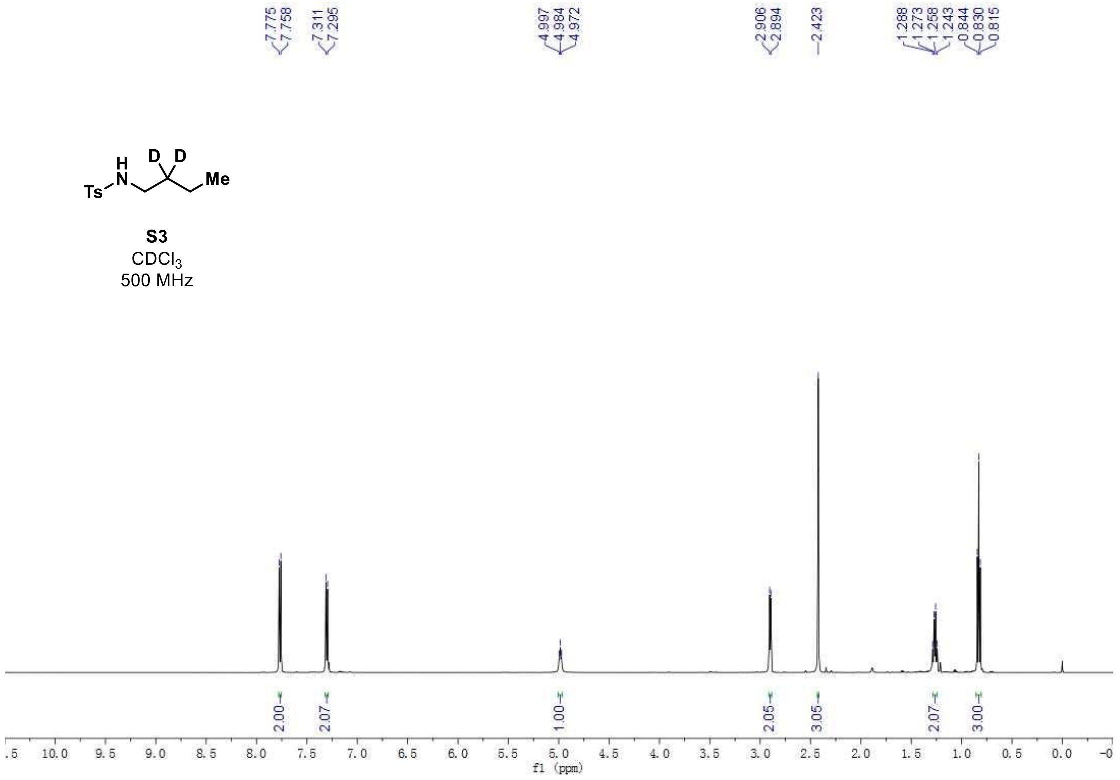

Supplementary Figure 12. ^{1}H NMR of compound S3. The sample has been recorded in 500 MHz, $CDCl_3$ at 25 °C.

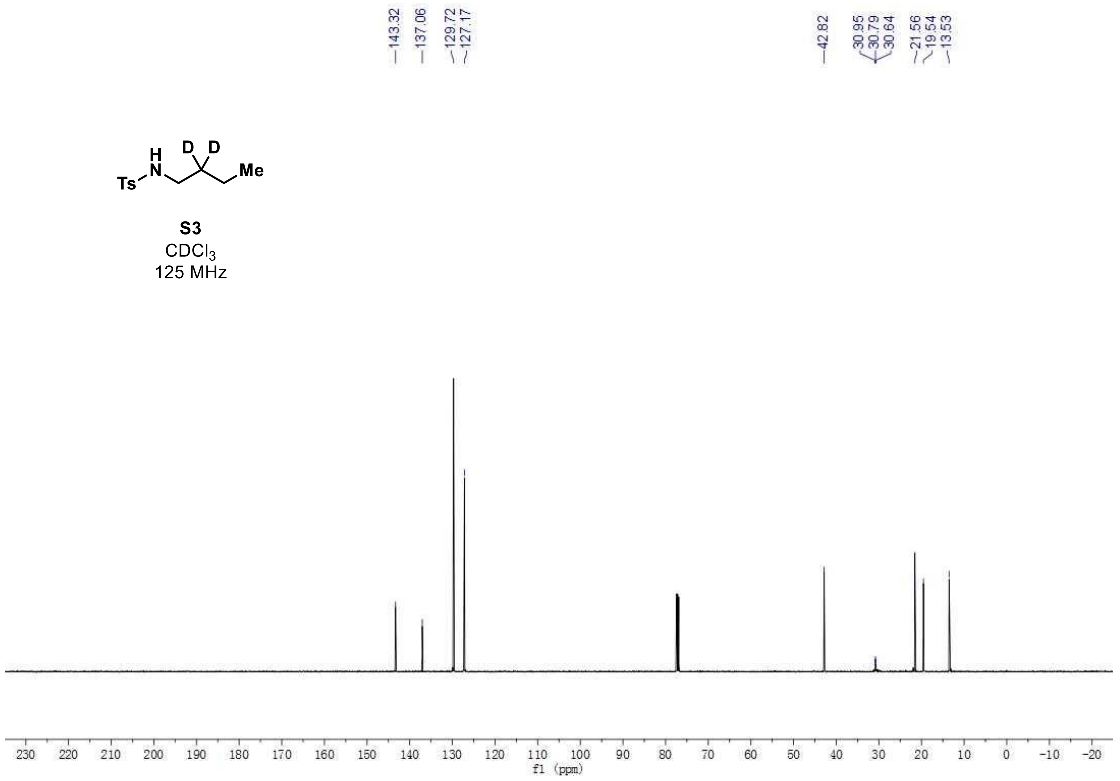

Supplementary Figure 13. ^{13}C NMR of compound S3. The sample has been recorded in 125 MHz, $CDCl_3$ at 25 °C.

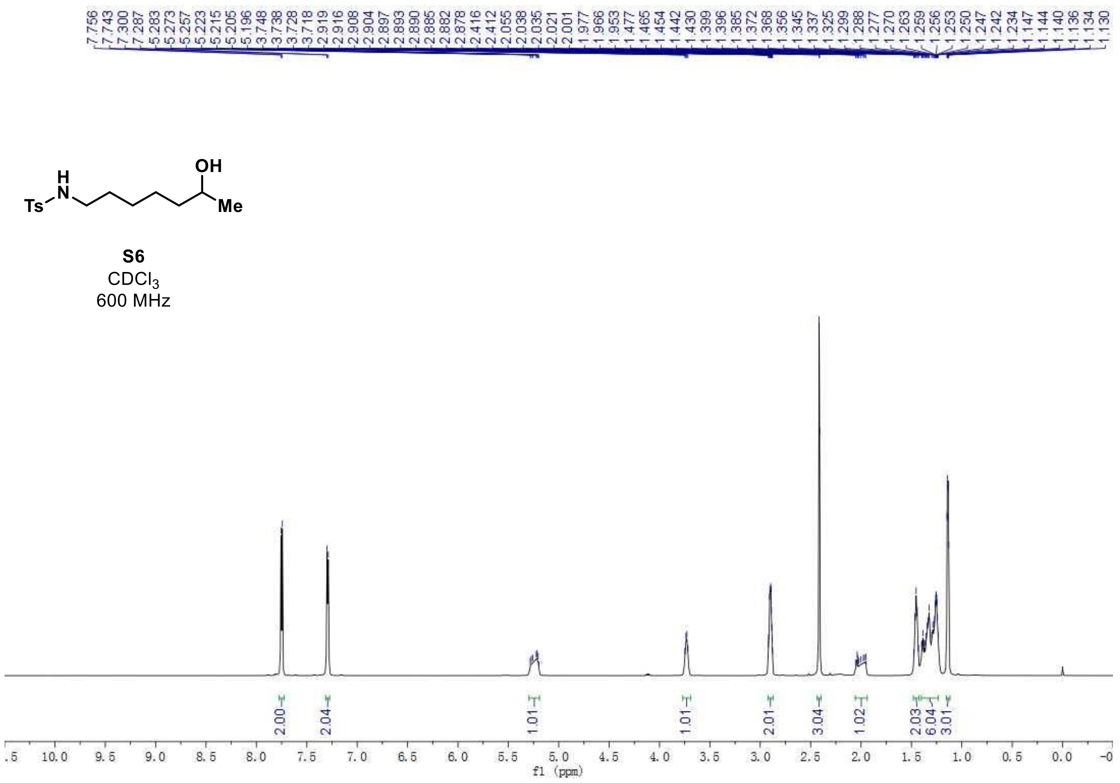

Supplementary Figure 14. ^{1}H NMR of compound S6. The sample has been recorded in 600 MHz, $CDCl_3$ at 25 °C.

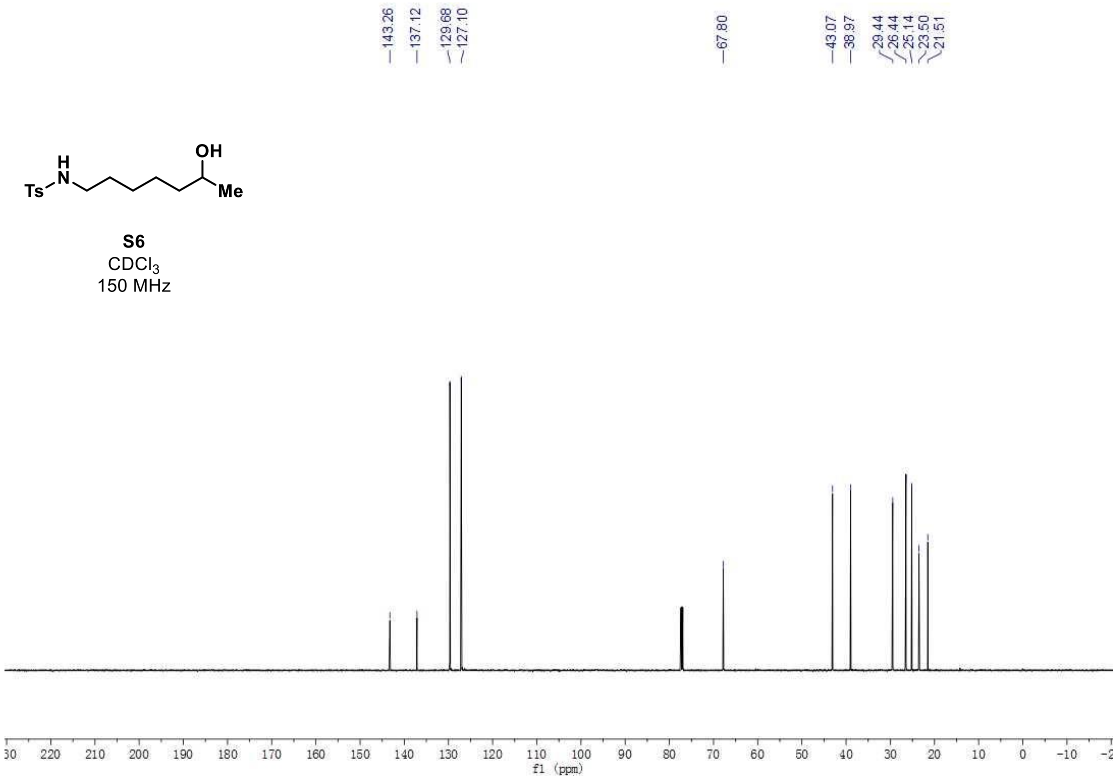

Supplementary Figure 15. ^{13}C NMR of compound S6. The sample has been recorded in 150 MHz, $CDCl_3$ at 25 °C.

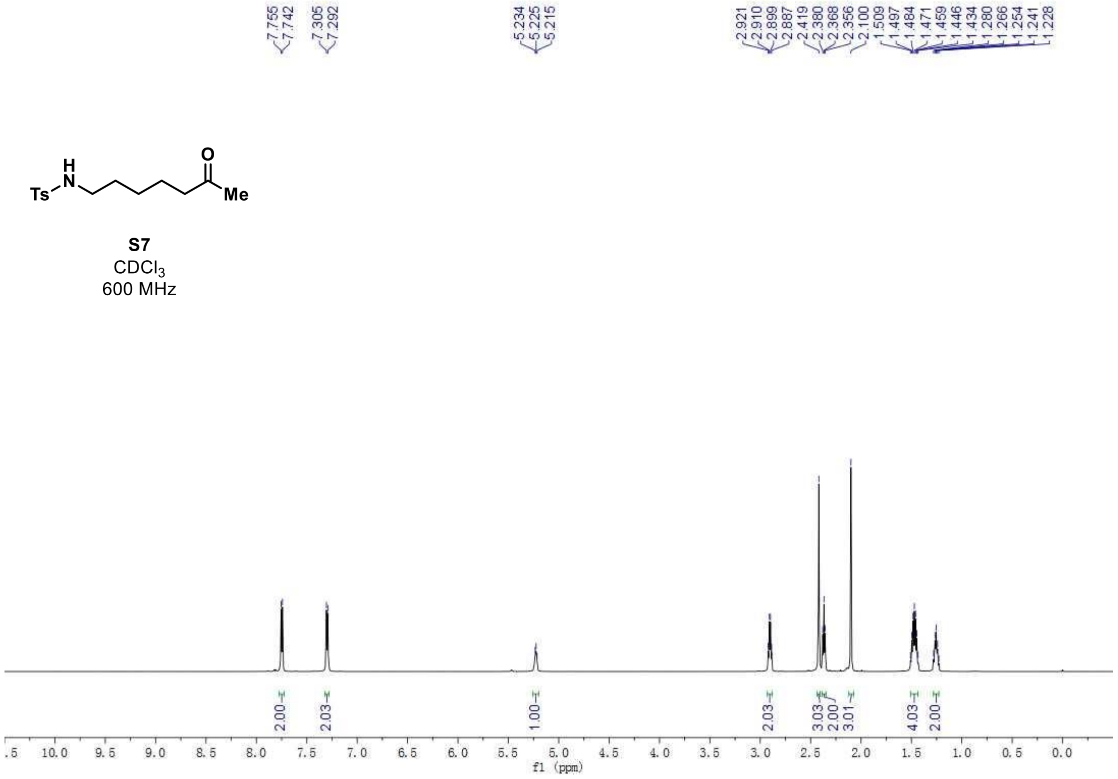

Supplementary Figure 16. ^{1}H NMR of compound S7. The sample has been recorded in 600 MHz, $CDCl_3$ at 25 °C.

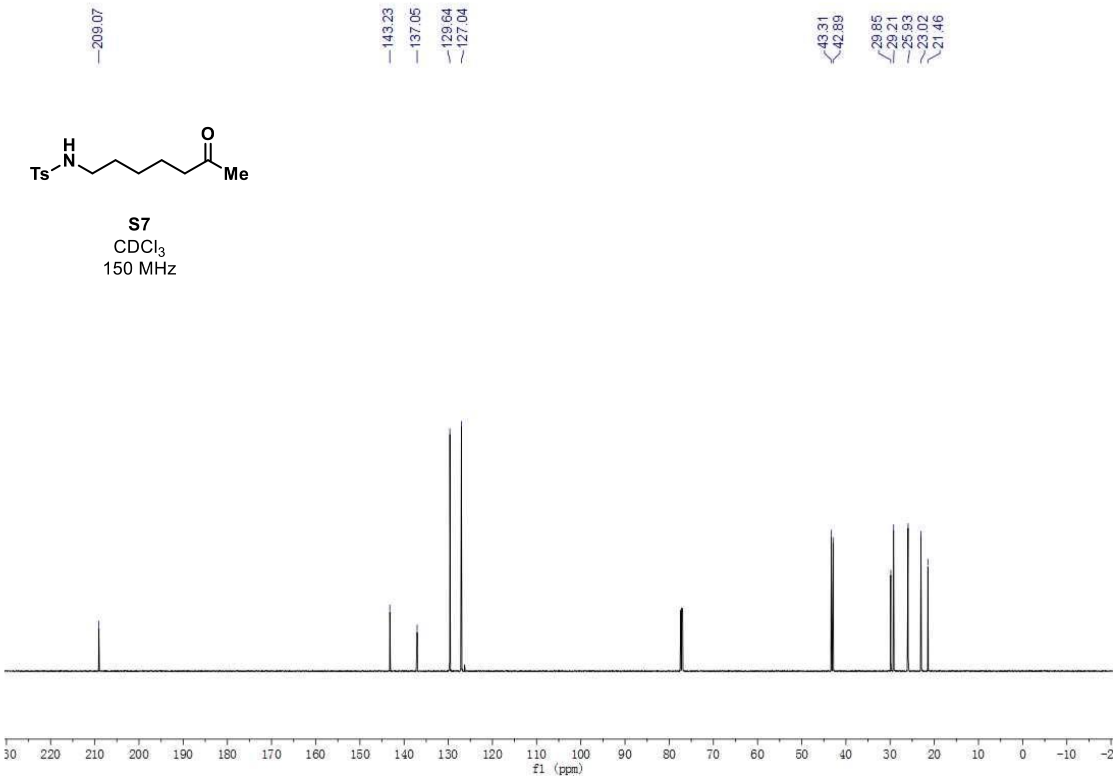

Supplementary Figure 17. ^{13}C NMR of compound S7. The sample has been recorded in 150 MHz, $CDCl_3$ at 25 °C.

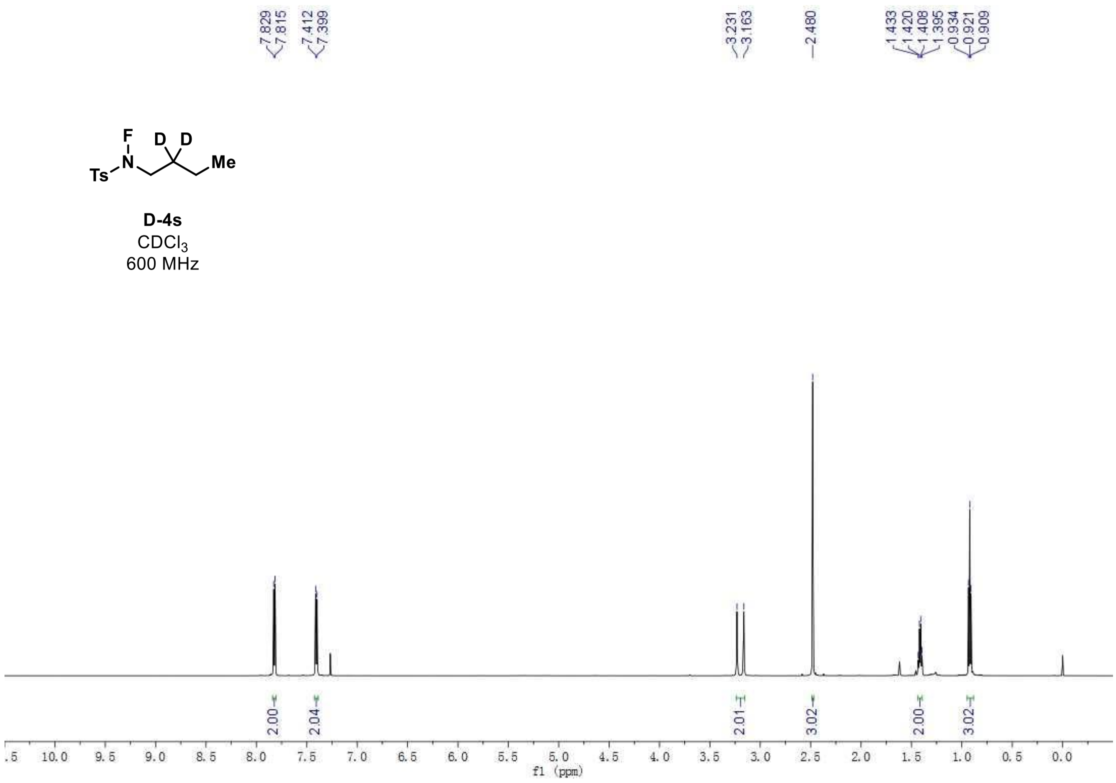

Supplementary Figure 18. ^{1}H NMR of compound D-4s. The sample has been recorded in 600 MHz, $CDCl_3$ at 25 °C.

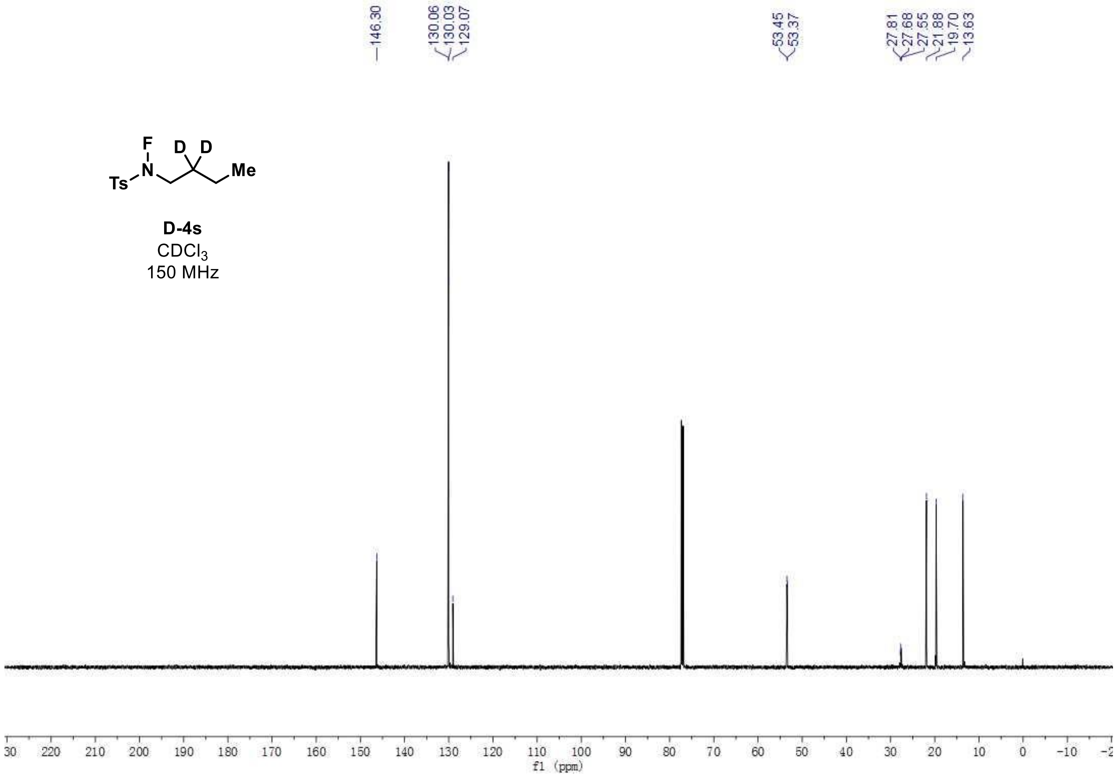

Supplementary Figure 19. ^{13}C NMR of compound D-4s. The sample has been recorded in 150 MHz, $CDCl_3$ at 25 °C.

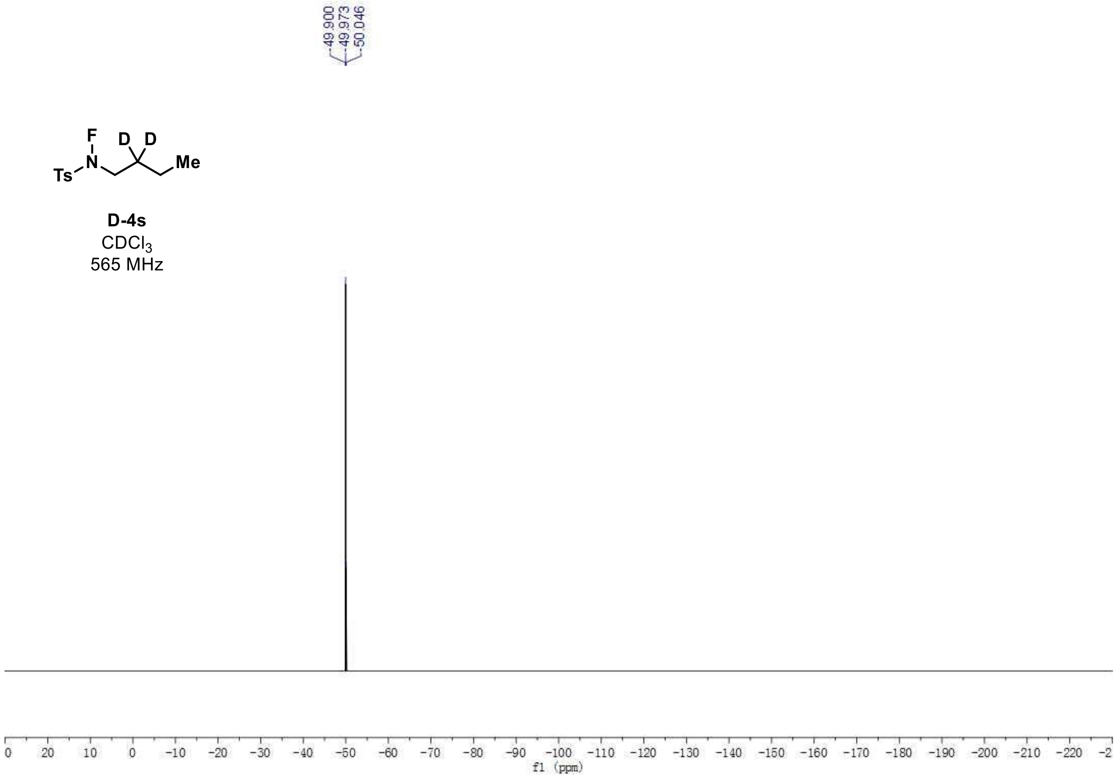

Supplementary Figure 20. ^{19}F NMR of compound D-4s. The sample has been recorded in 565 MHz, $CDCl_3$ at 25 °C.

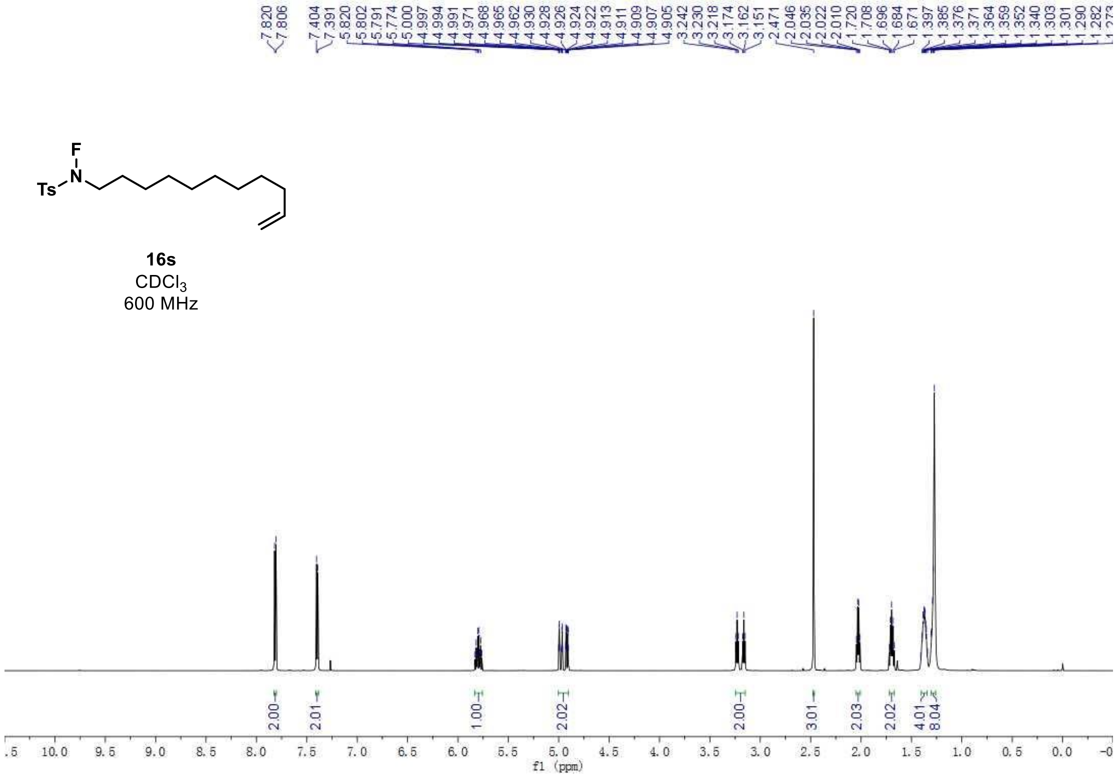

Supplementary Figure 21. ^{1}H NMR of compound 16s. The sample has been recorded in 600 MHz, $CDCl_3$ at 25 °C.

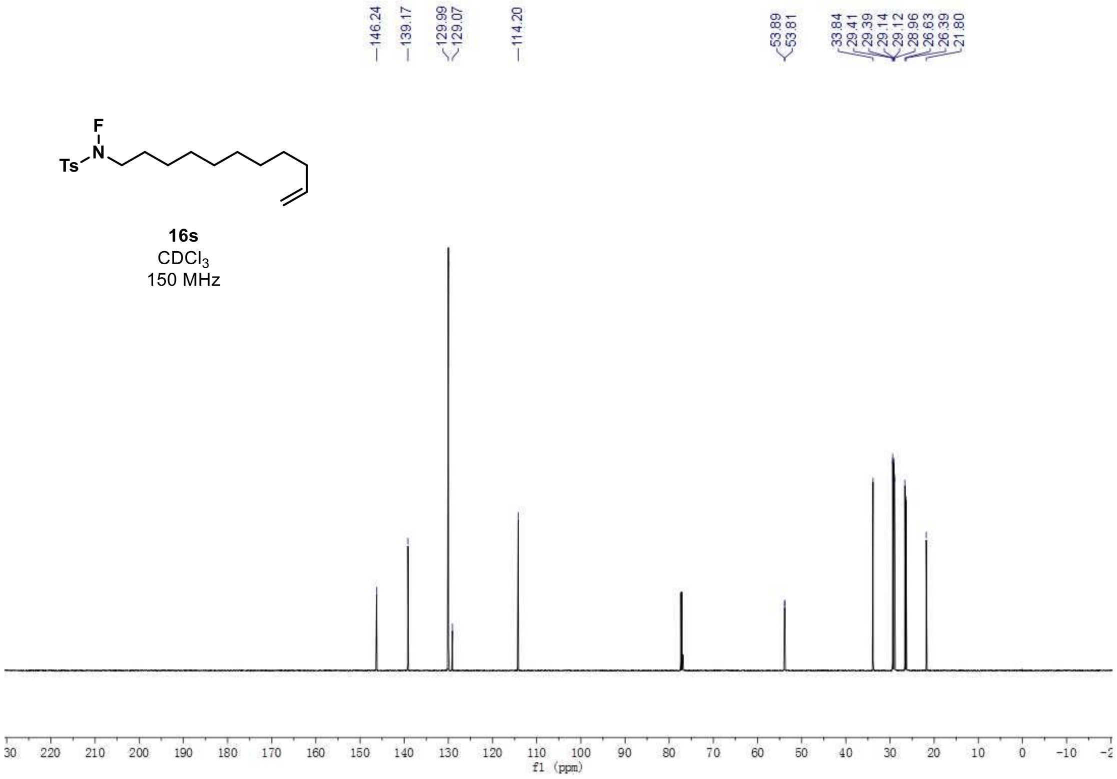

Supplementary Figure 22. ^{13}C NMR of compound 16s. The sample has been recorded in 150 MHz, $CDCl_3$ at 25 °C.

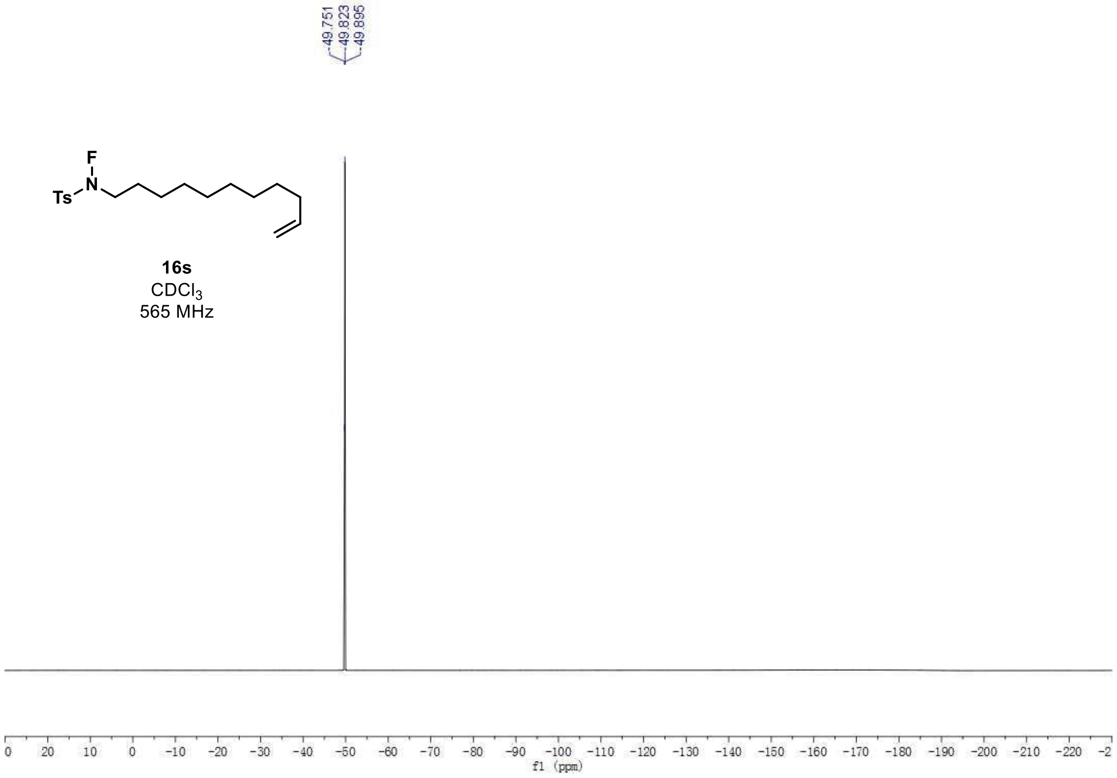

Supplementary Figure 23. ^{19}F NMR of compound 16s. The sample has been recorded in 565 MHz, $CDCl_3$ at 25 °C.

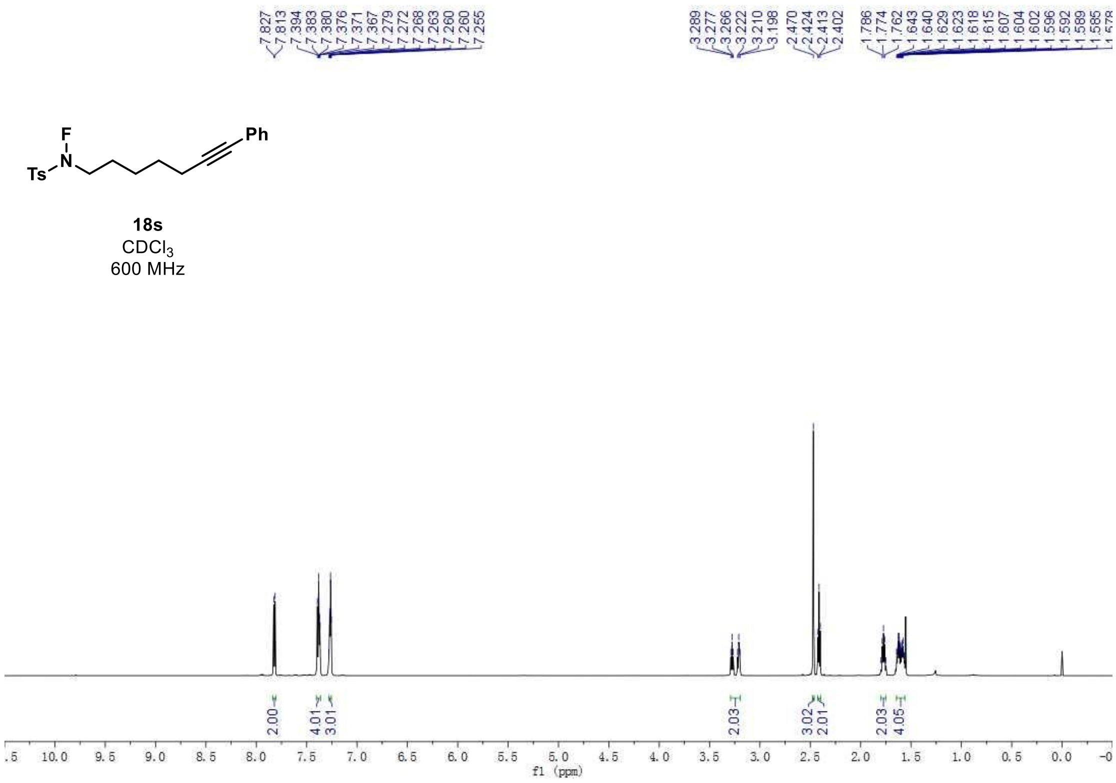

Supplementary Figure 24. ^{1}H NMR of compound 18s. The sample has been recorded in 600 MHz, $CDCl_3$ at 25 °C.

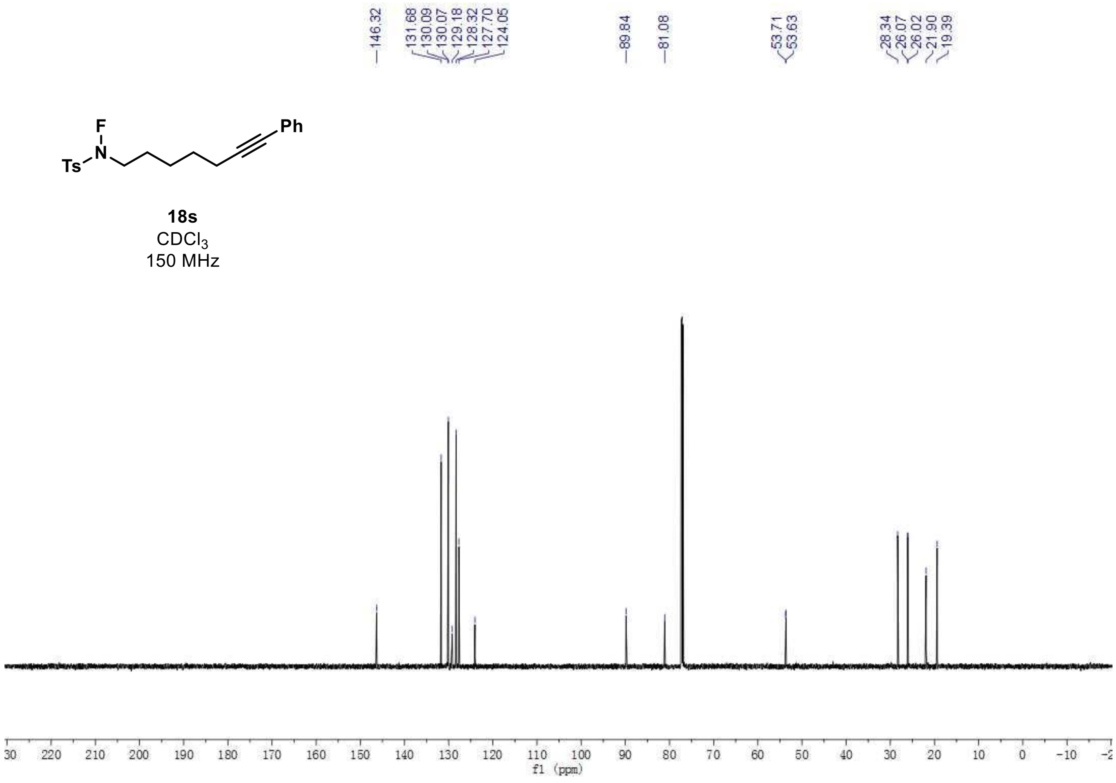

Supplementary Figure 25. ^{13}C NMR of compound 18s. The sample has been recorded in 150 MHz, $CDCl_3$ at 25 °C.

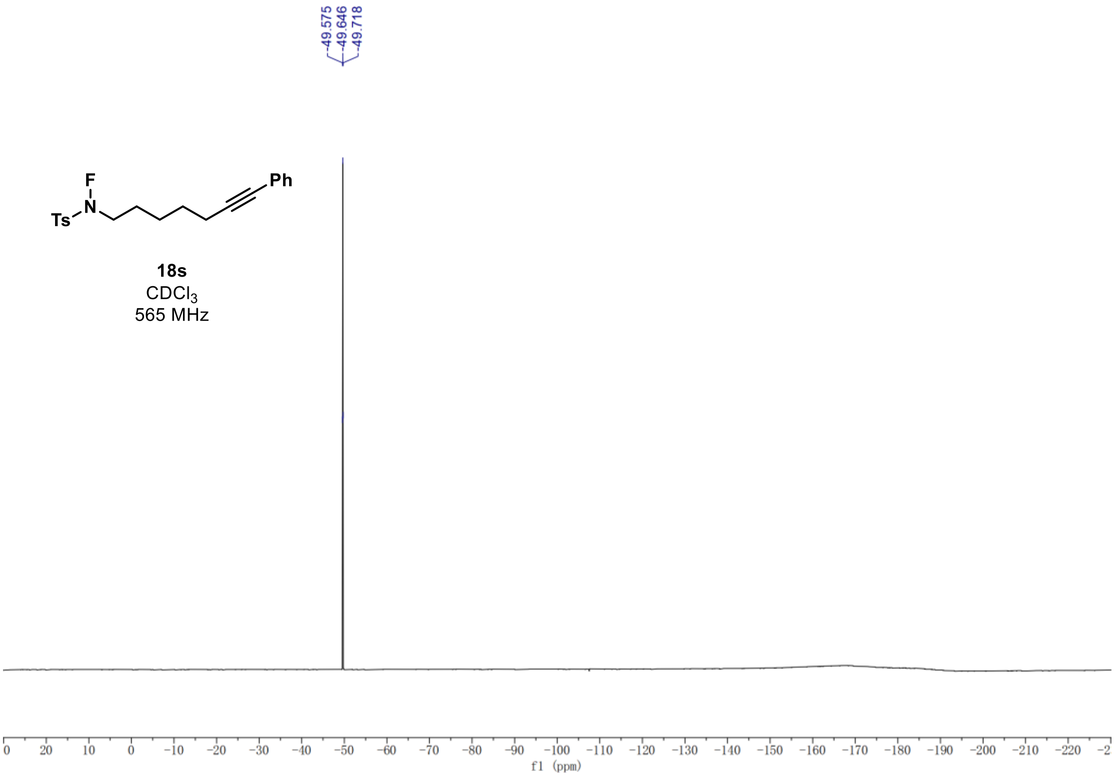

Supplementary Figure 26. ^{19}F NMR of compound 18s. The sample has been recorded in 565 MHz, $CDCl_3$ at 25 °C.

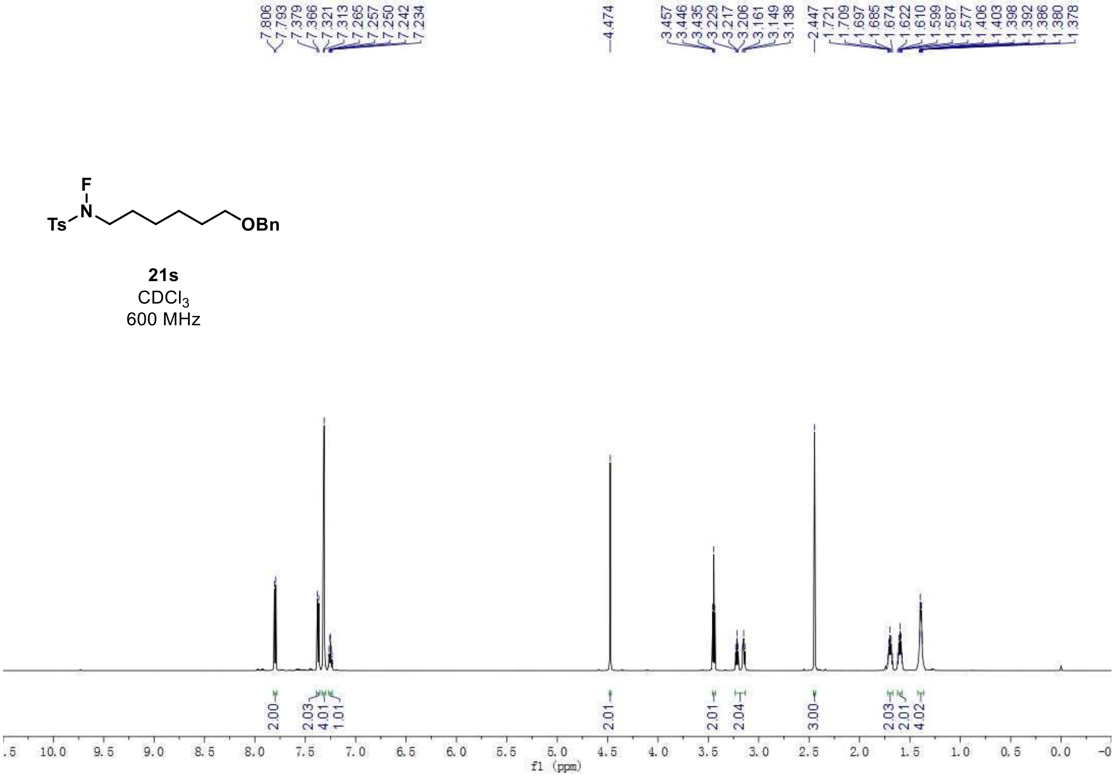

Supplementary Figure 27. ^{1}H NMR of compound 21s. The sample has been recorded in 600 MHz, $CDCl_3$ at 25 °C.

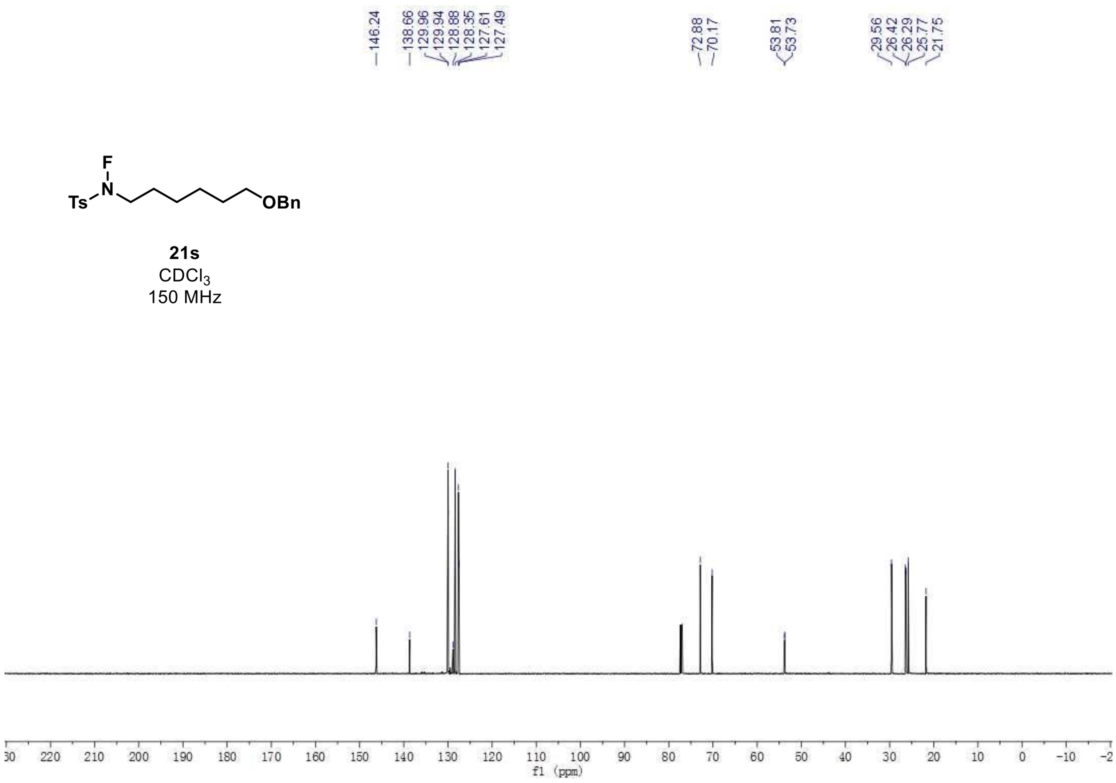

Supplementary Figure 28. ^{13}C NMR of compound 21s. The sample has been recorded in 150 MHz, $CDCl_3$ at 25 °C.

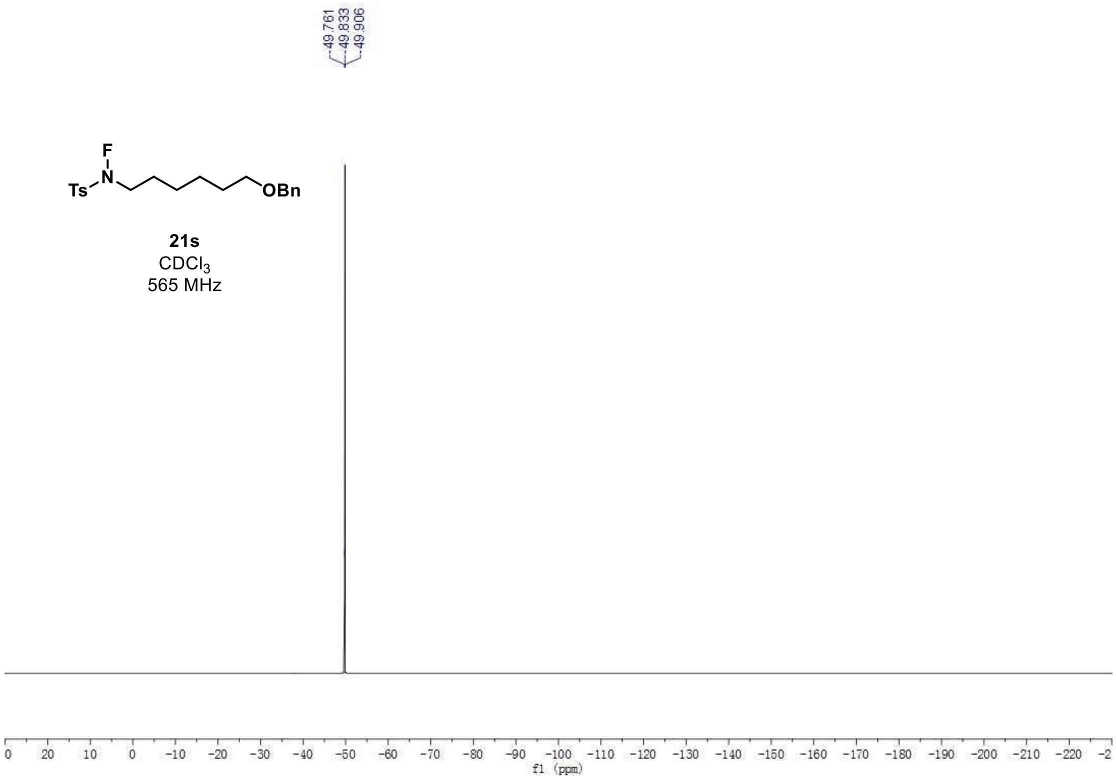

Supplementary Figure 29. ^{19}F NMR of compound 21s. The sample has been recorded in 565 MHz, $CDCl_3$ at 25 °C.

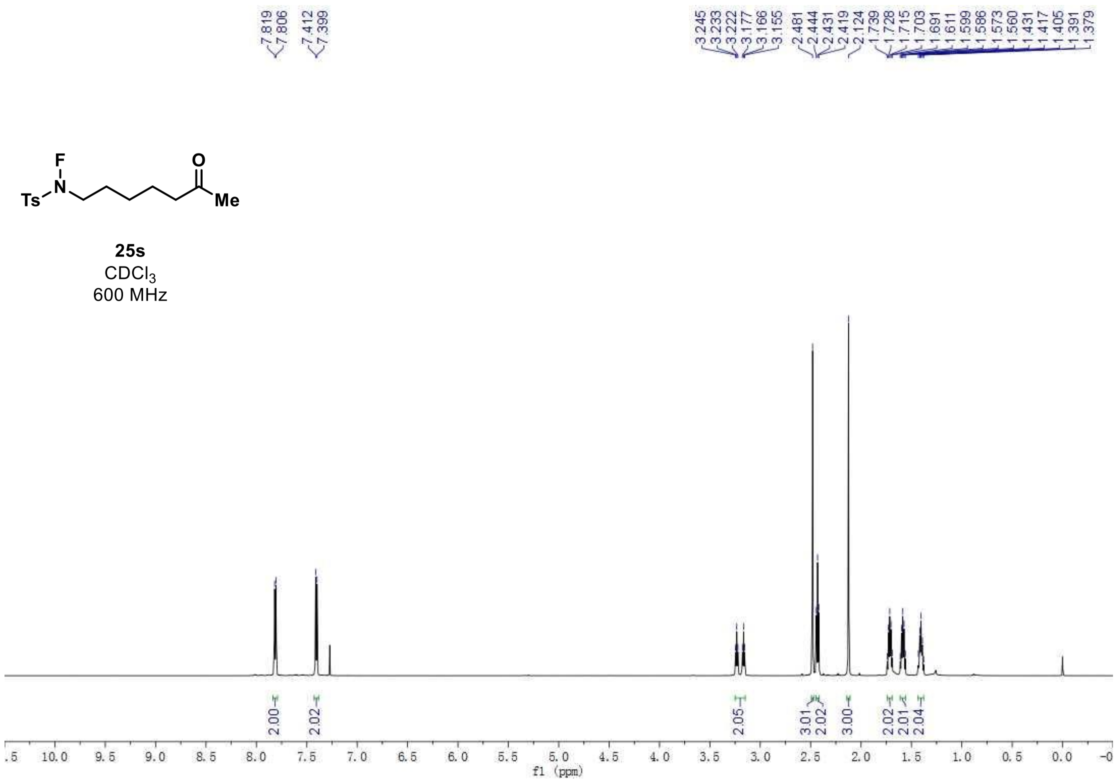

Supplementary Figure 30. ^{1}H NMR of compound 25s. The sample has been recorded in 600 MHz, $CDCl_3$ at 25 °C.

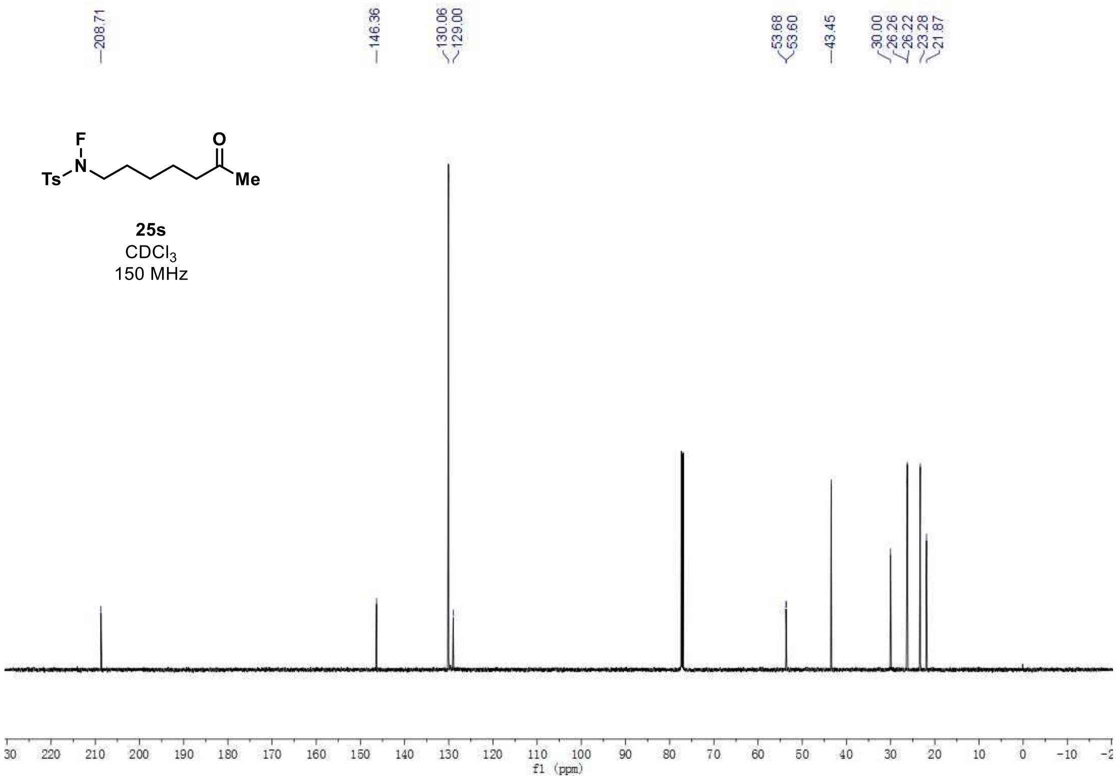

Supplementary Figure 31. ^{13}C NMR of compound 25s. The sample has been recorded in 150 MHz, $CDCl_3$ at 25 °C.

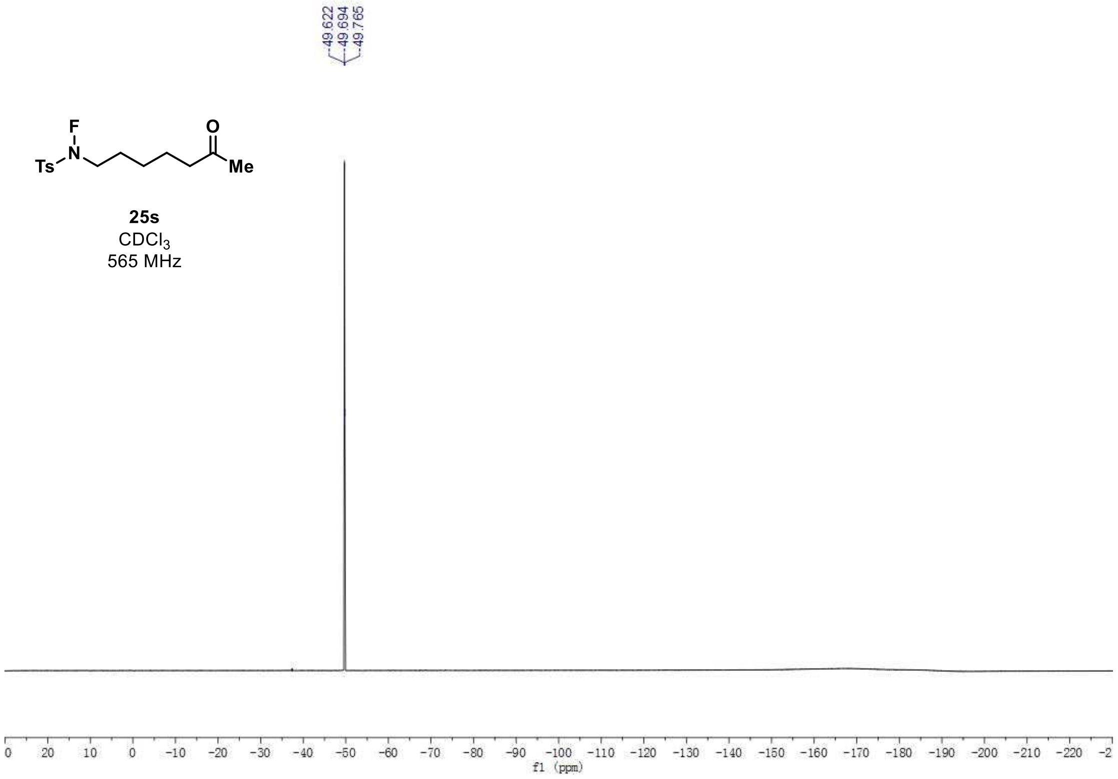

Supplementary Figure 32. ^{19}F NMR of compound 25s. The sample has been recorded in 565 MHz, $CDCl_3$ at 25 °C.

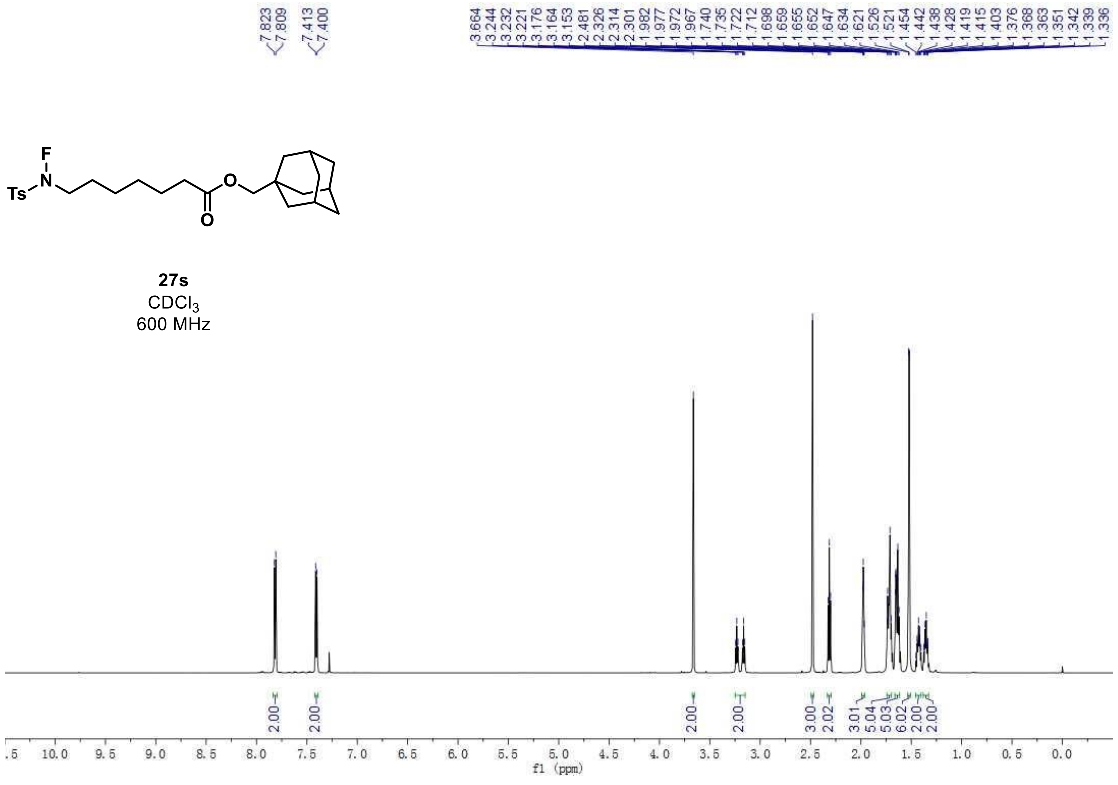

Supplementary Figure 33. ^{1}H NMR of compound 27s. The sample has been recorded in 600 MHz, $CDCl_3$ at 25 °C.

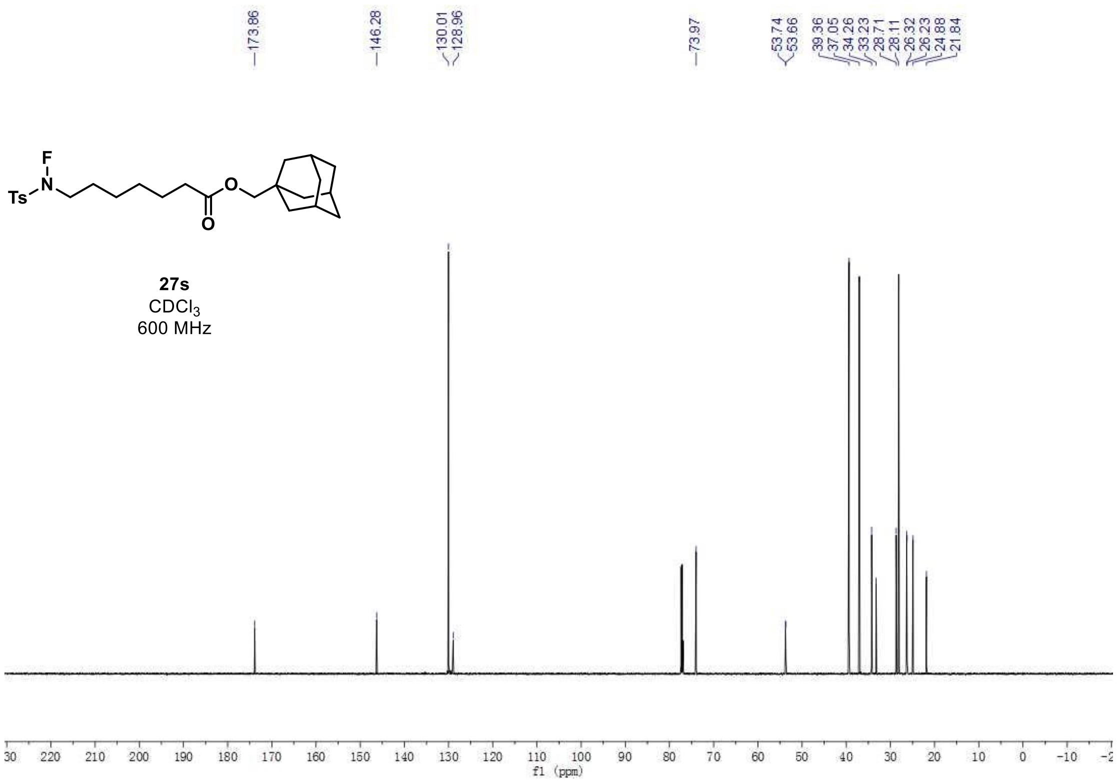

Supplementary Figure 34. ^{13}**C NMR of compound 27s.** The sample has been recorded in 150 MHz, $CDCl_3$ at 25 °C.

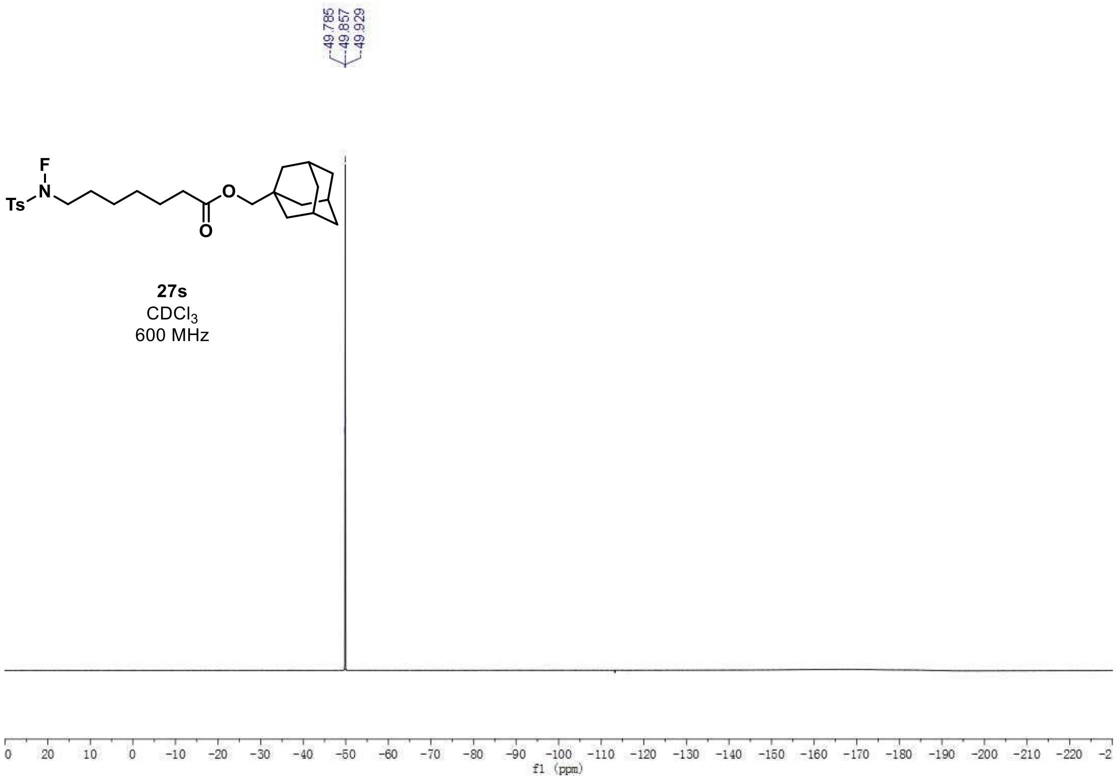

Supplementary Figure 35. ^{19}**F NMR of compound 27s.** The sample has been recorded in 565 MHz, $CDCl_3$ at 25 °C.

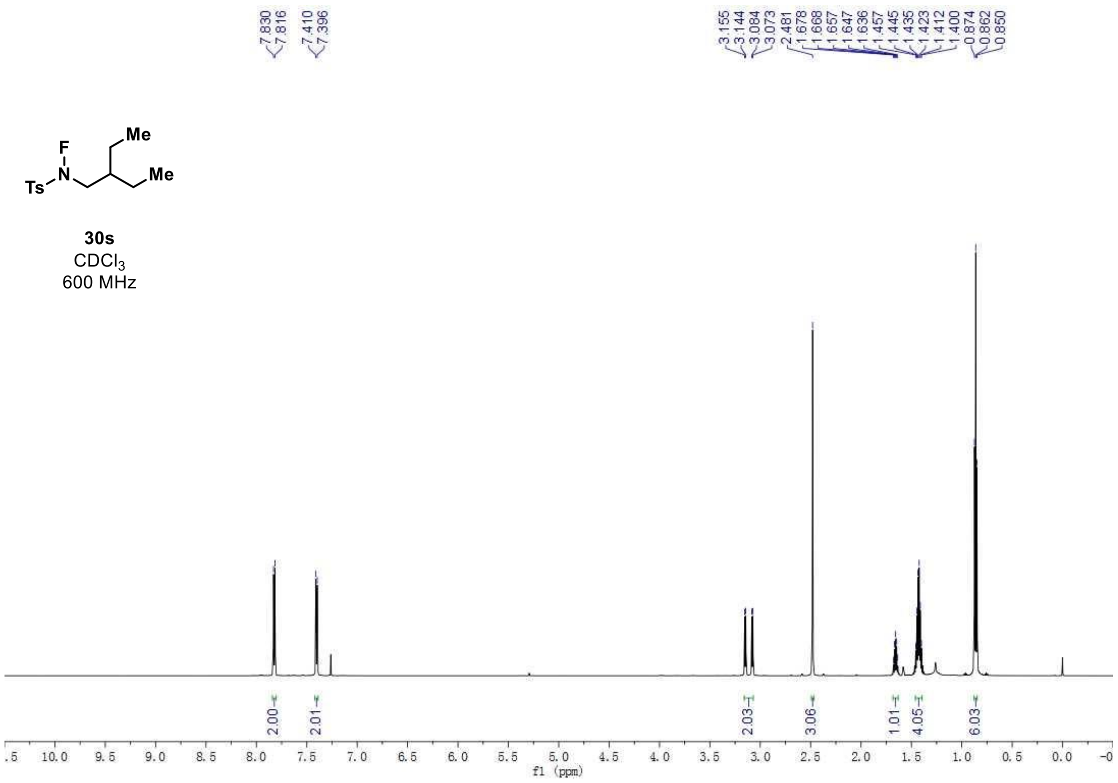

Supplementary Figure 36. ^{1}H NMR of compound 30s. The sample has been recorded in 600 MHz, $CDCl_3$ at 25 °C.

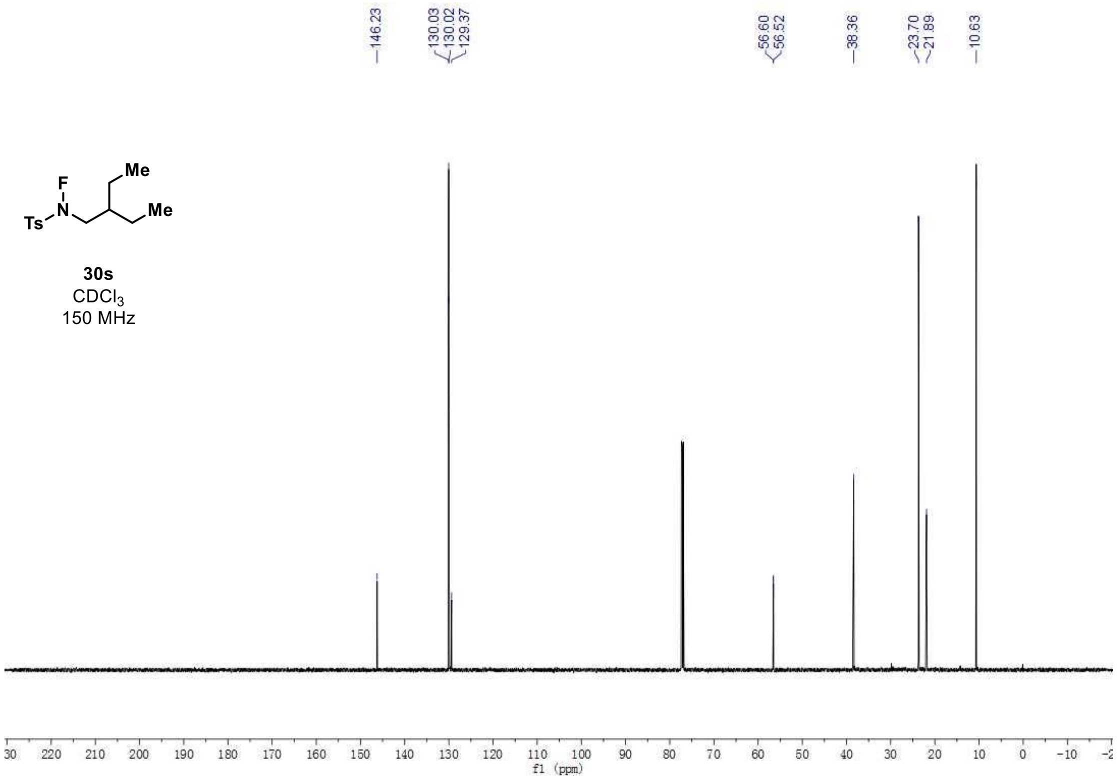

Supplementary Figure 37. ^{13}C NMR of compound 30s. The sample has been recorded in 150 MHz, $CDCl_3$ at 25 °C.

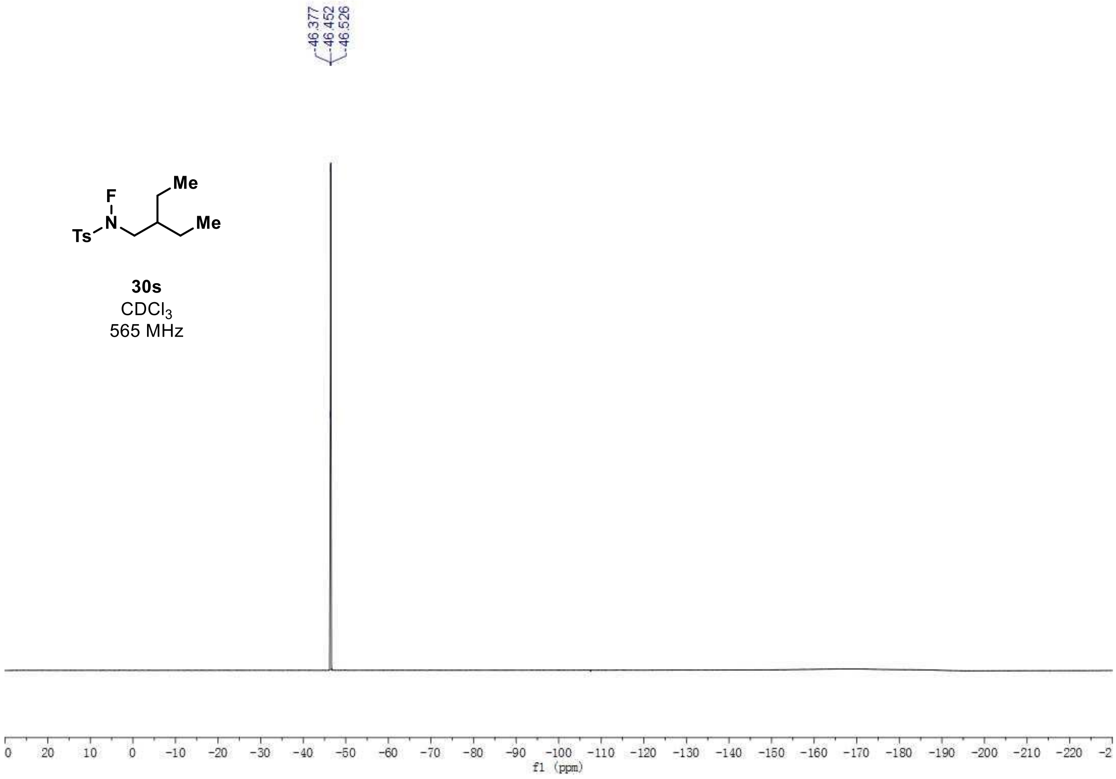

Supplementary Figure 38. ^{19}F NMR of compound 30s. The sample has been recorded in 565 MHz, $CDCl_3$ at 25 °C.

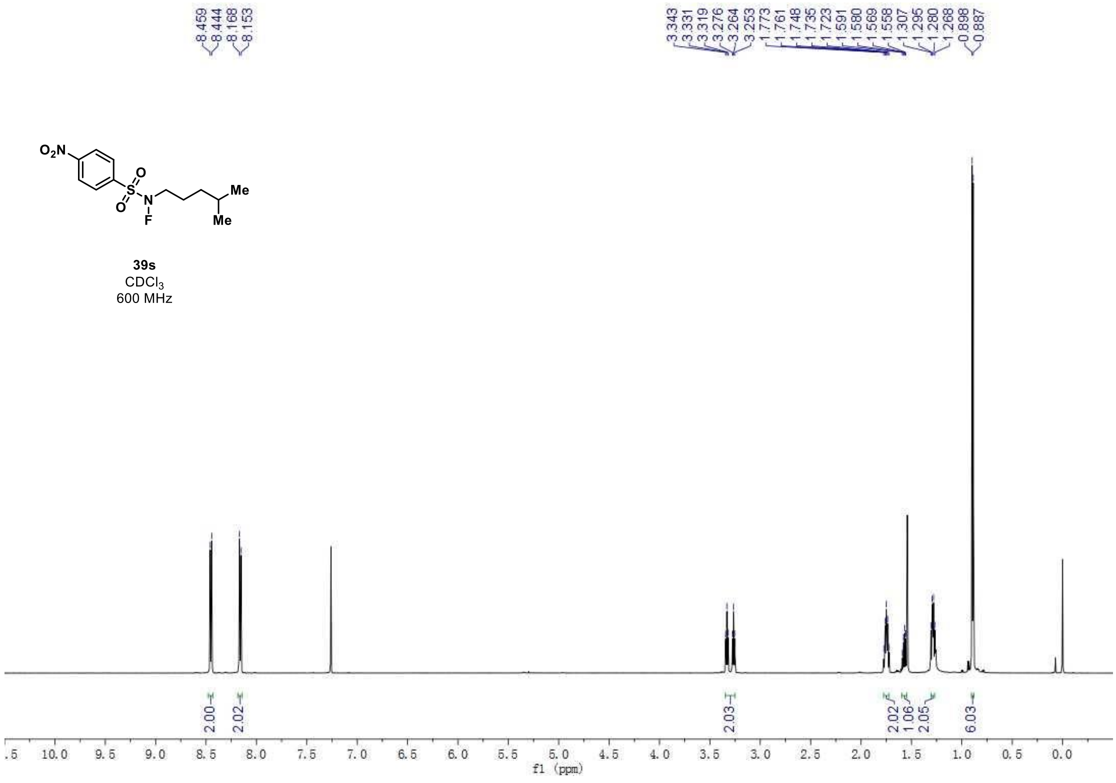

Supplementary Figure 39. ^{1}H NMR of compound 39s. The sample has been recorded in 600 MHz, $CDCl_3$ at 25 °C.

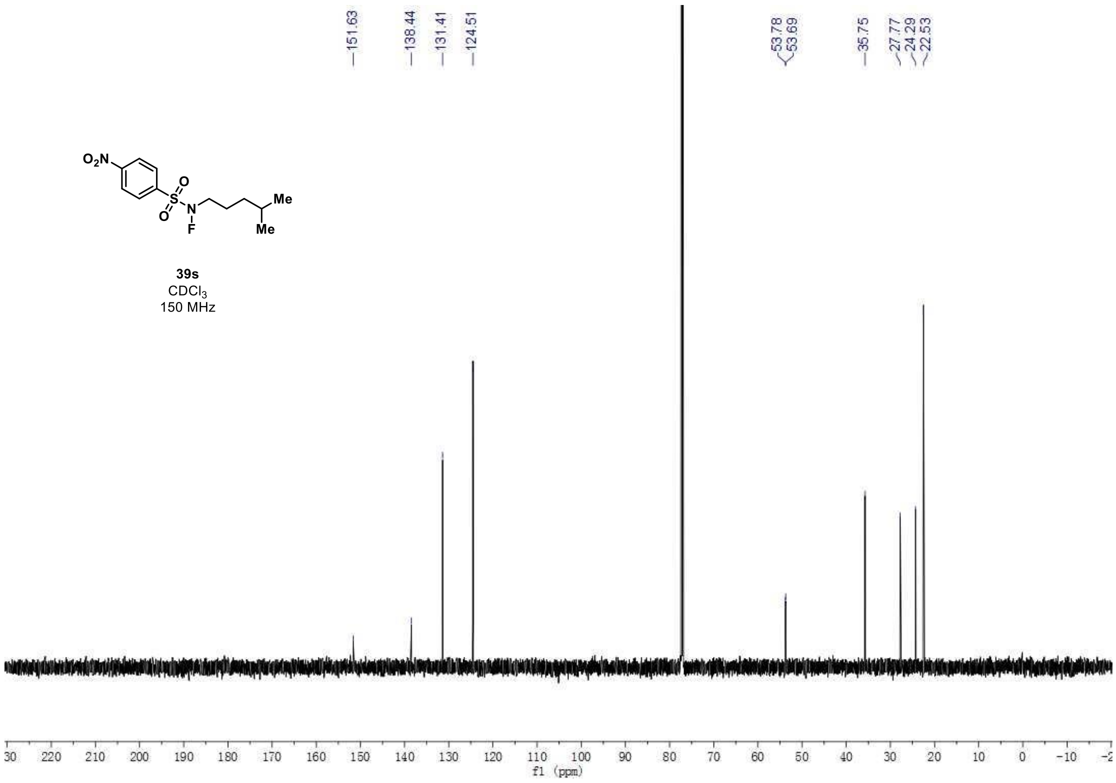

Supplementary Figure 40. ^{13}C NMR of compound 39s. The sample has been recorded in 150 MHz, $CDCl_3$ at 25 °C.

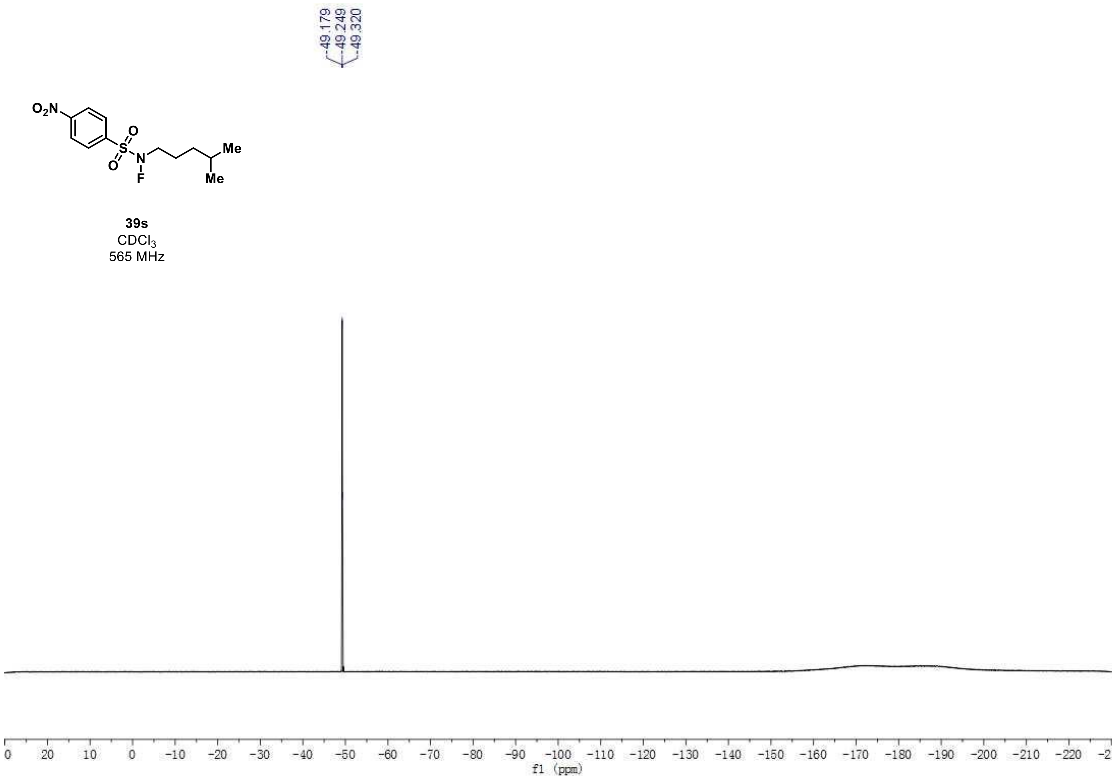

Supplementary Figure 41. ^{19}F NMR of compound 39s. The sample has been recorded in 565 MHz, $CDCl_3$ at 25 °C.

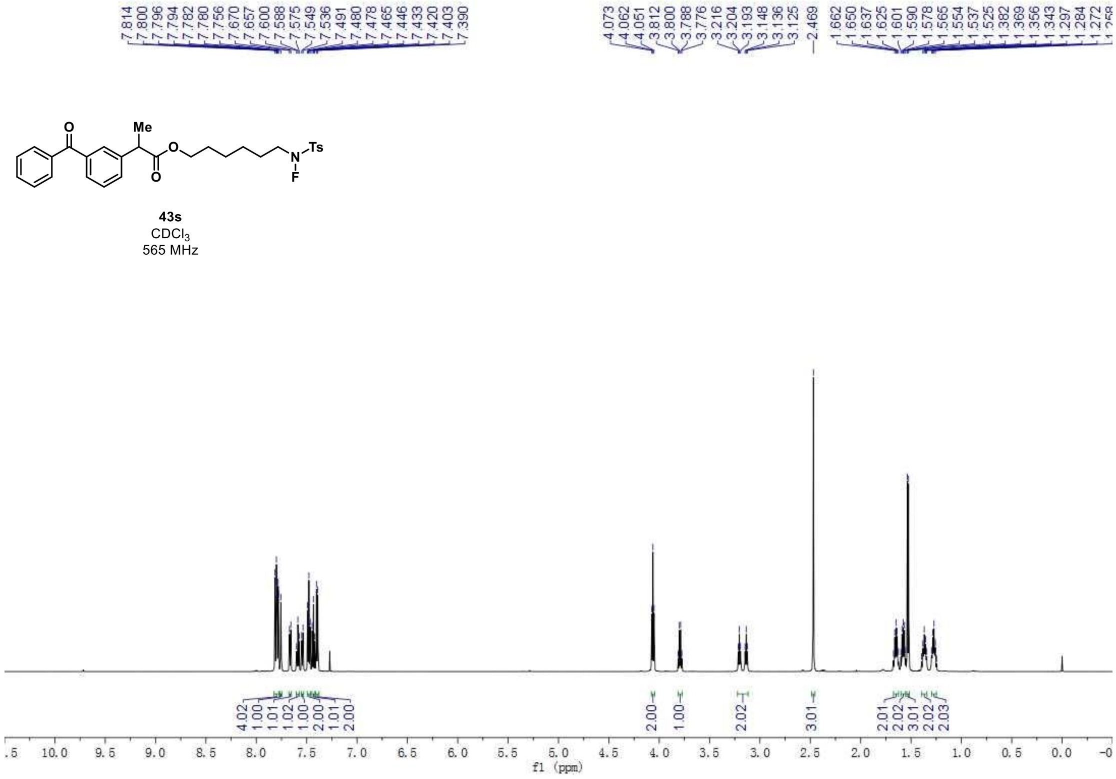

Supplementary Figure 42. ^{1}H NMR of compound 43s. The sample has been recorded in 600 MHz, $CDCl_3$ at 25 °C.

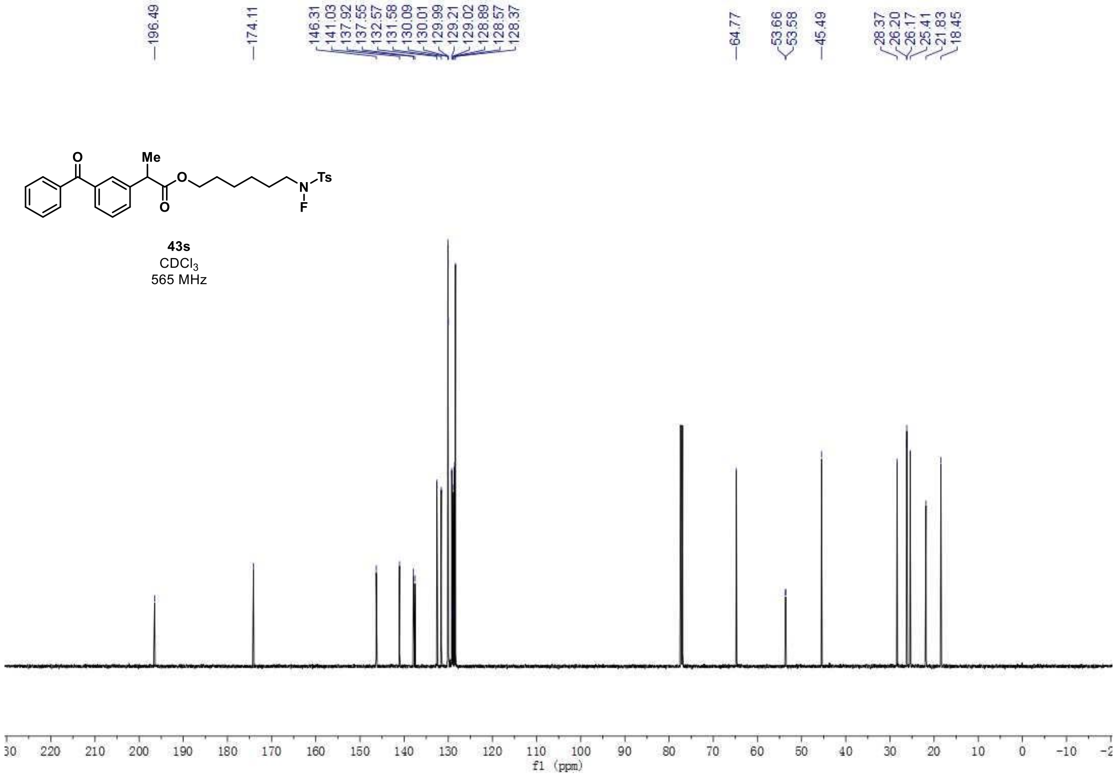

Supplementary Figure 43. ^{13}C NMR of compound 43s. The sample has been recorded in 150 MHz, $CDCl_3$ at 25 °C.

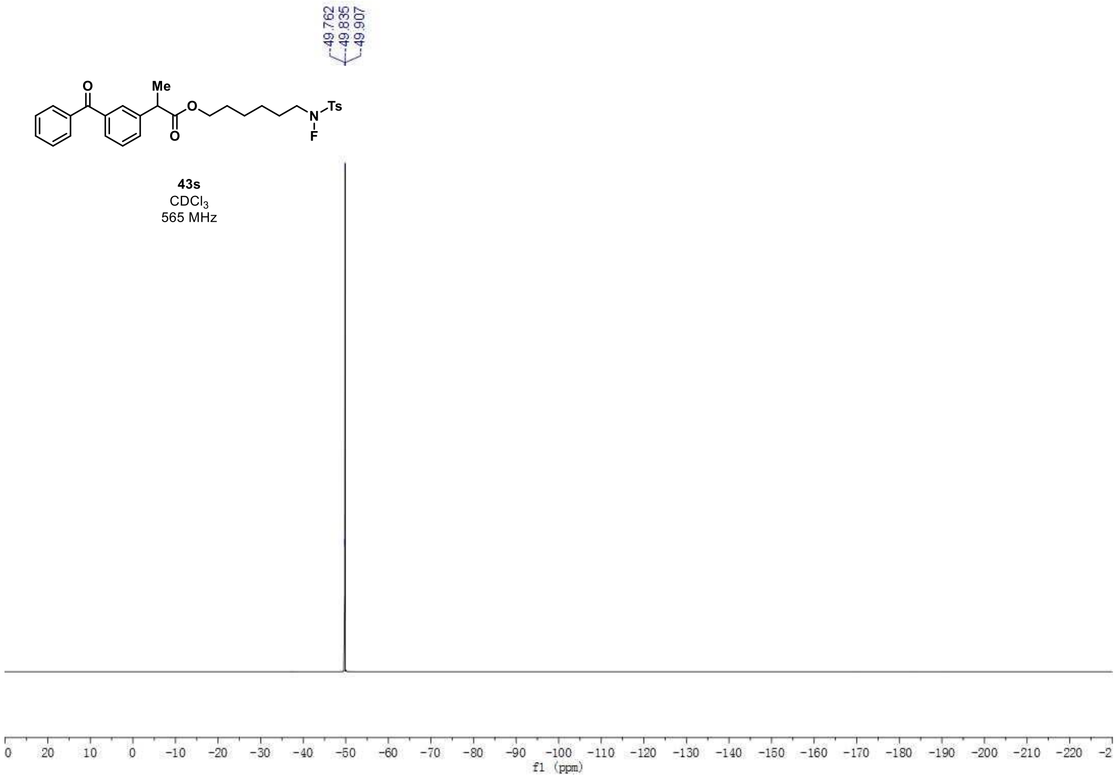

Supplementary Figure 44. ^{19}F NMR of compound 43s. The sample has been recorded in 565 MHz, $CDCl_3$ at 25 °C.

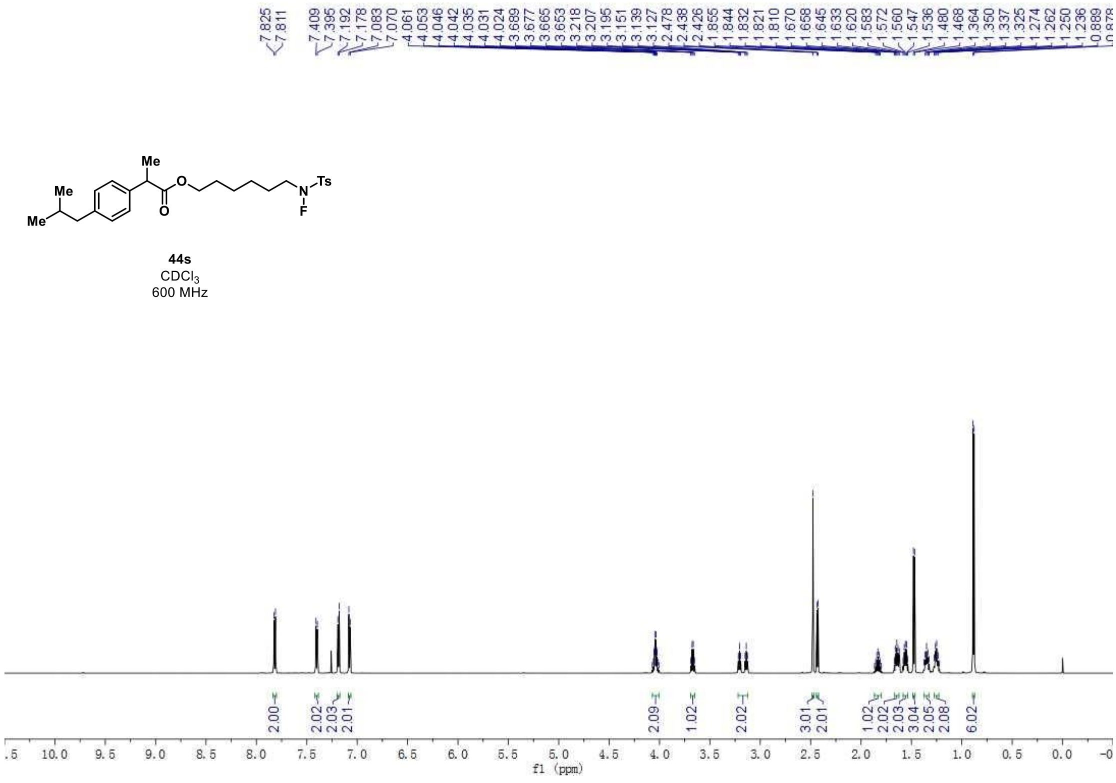

Supplementary Figure 45. ^{1}H NMR of compound 44s. The sample has been recorded in 600 MHz, $CDCl_3$ at 25 °C.

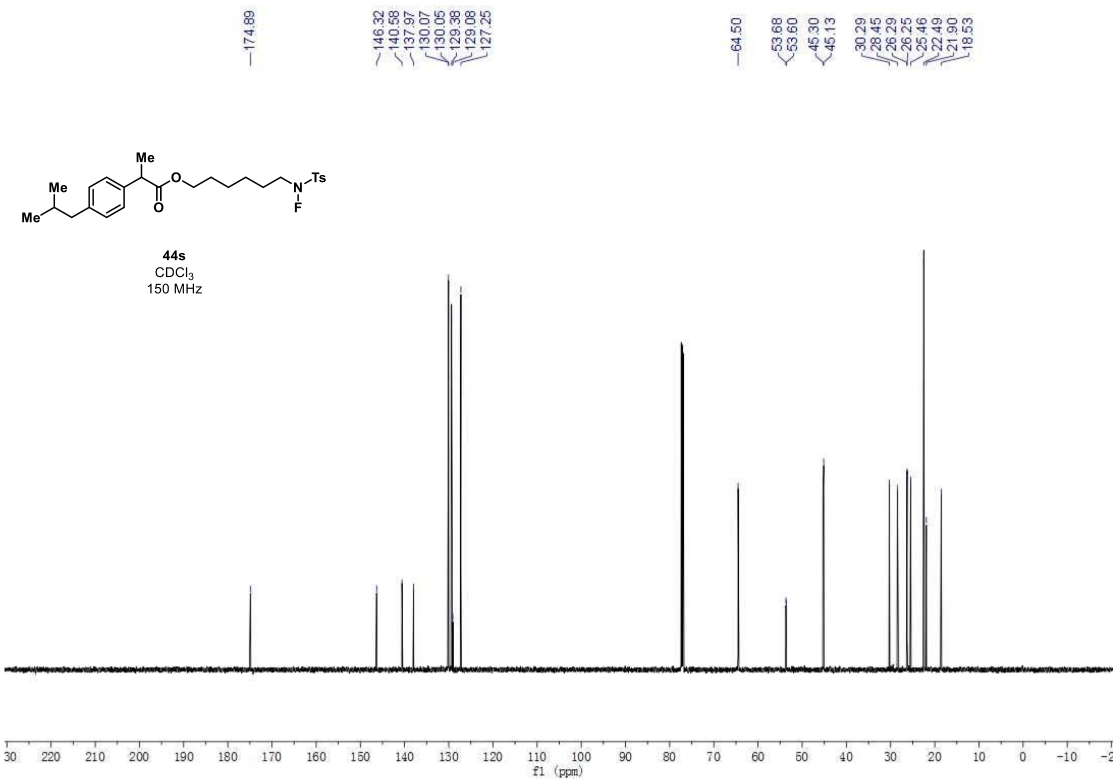

Supplementary Figure 46. ^{13}C NMR of compound 44s. The sample has been recorded in 150 MHz, $CDCl_3$ at 25 °C.

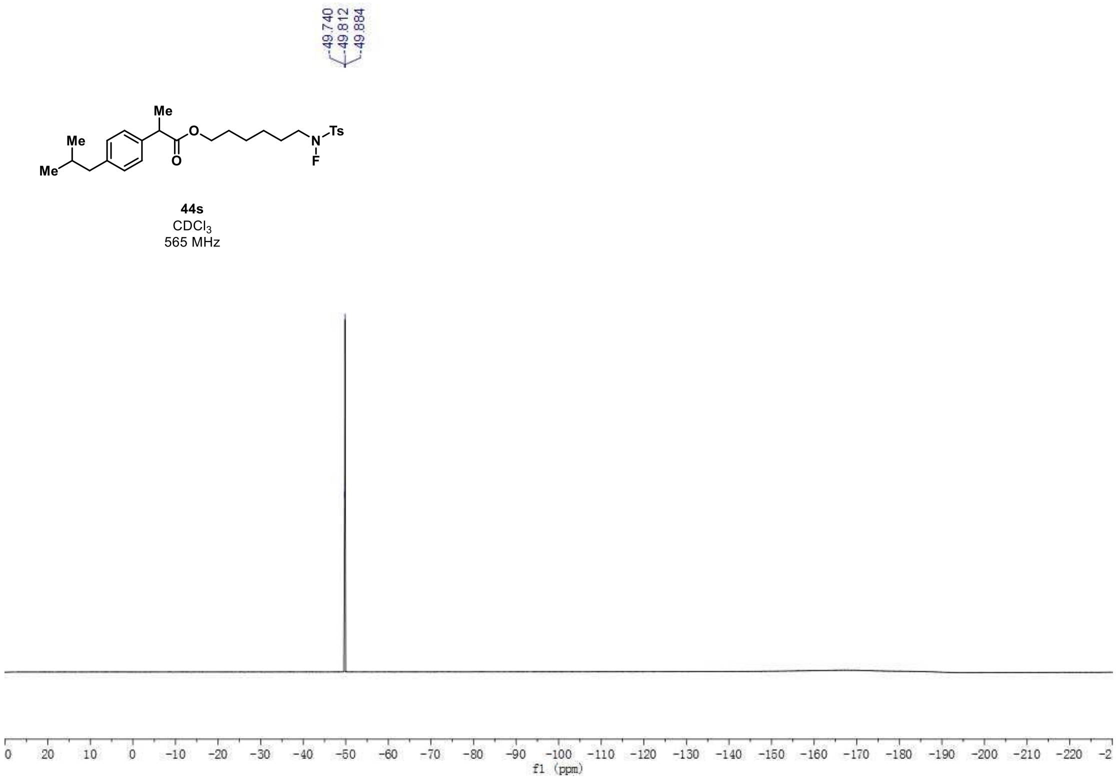

Supplementary Figure 47. ^{19}F NMR of compound 44s. The sample has been recorded in 565 MHz, $CDCl_3$ at 25 °C.

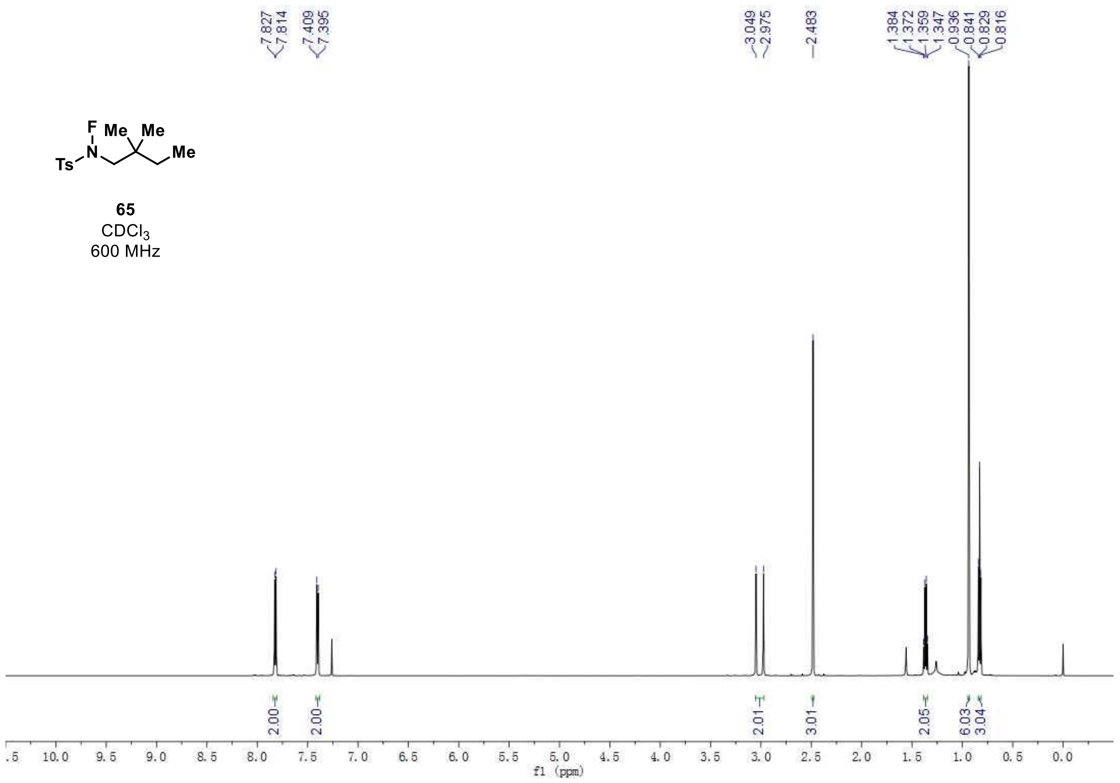

Supplementary Figure 48. ^{1}H NMR of compound 65. The sample has been recorded in 600 MHz, $CDCl_3$ at 25 °C.

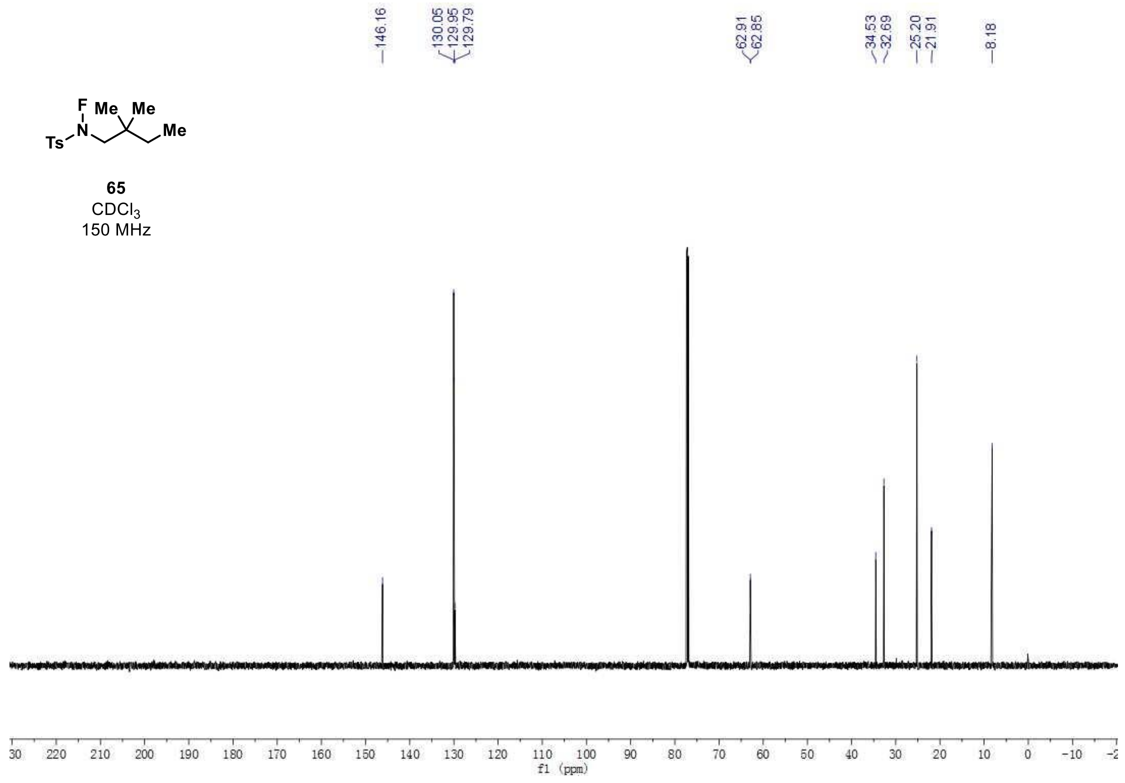

Supplementary Figure 49. ^{13}C NMR of compound 65. The sample has been recorded in 150 MHz, $CDCl_3$ at 25 °C.

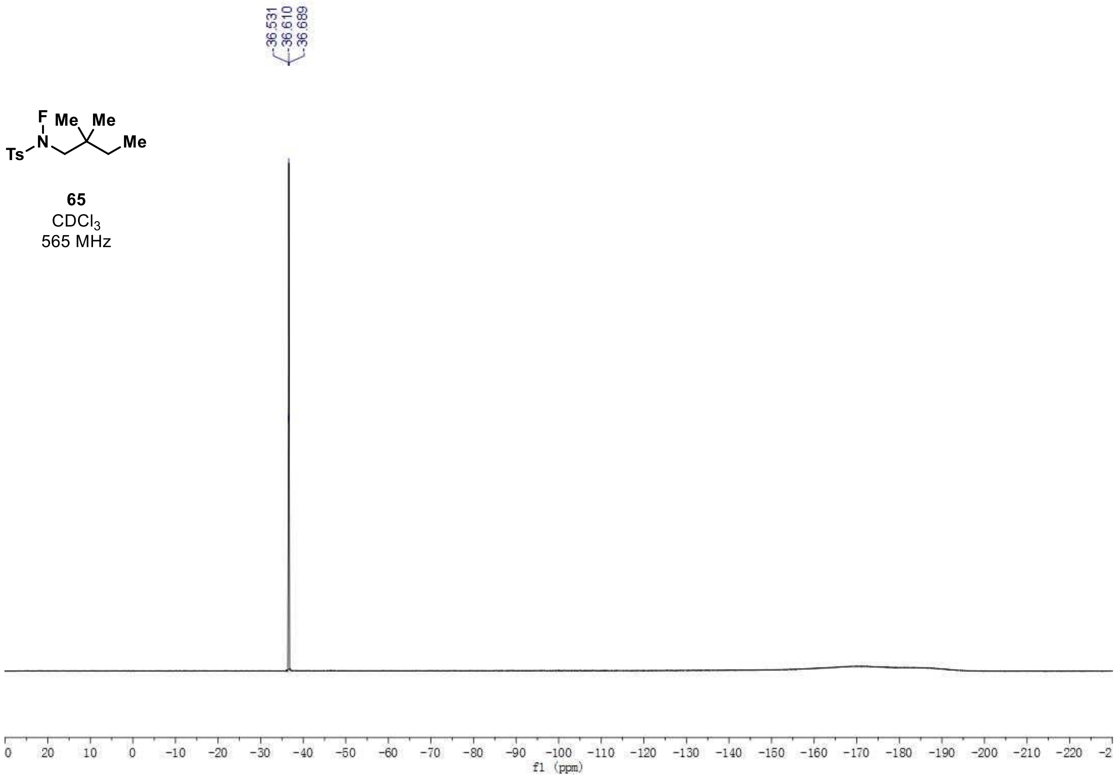

Supplementary Figure 50. ^{19}F NMR of compound 65. The sample has been recorded in 565 MHz, $CDCl_3$ at 25 °C.

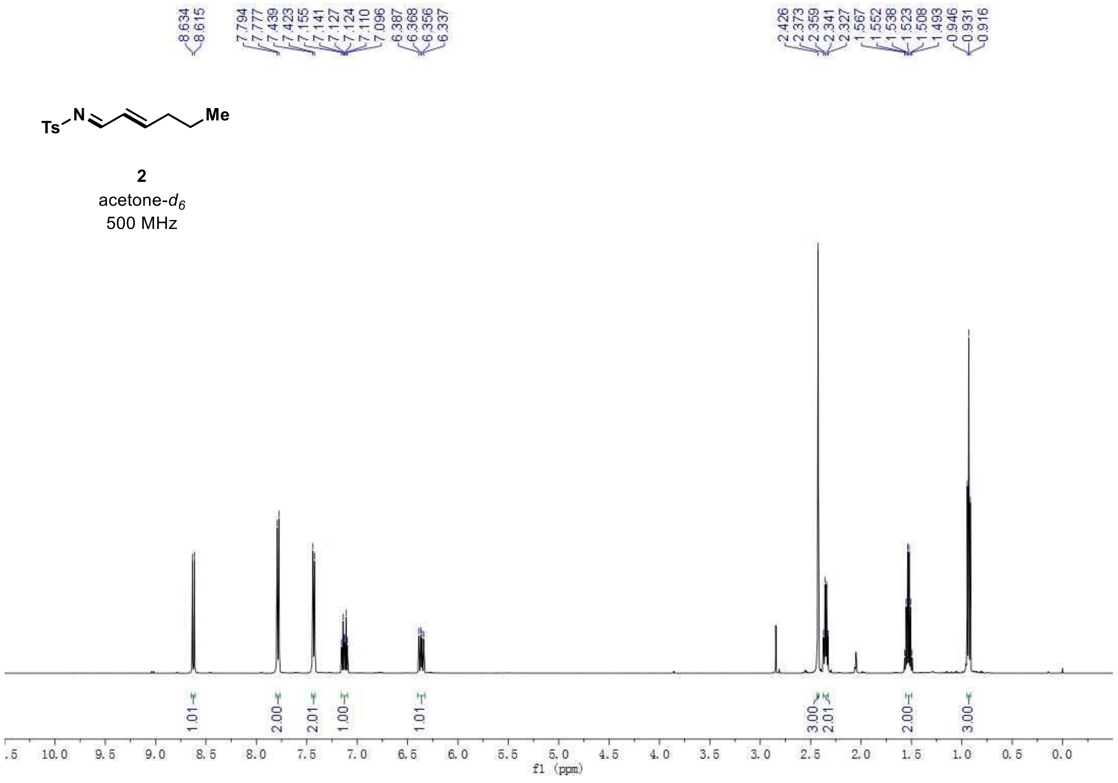

Supplementary Figure 51. ^{1}H NMR of compound 2. The sample has been recorded in 500 MHz, acetone-d_6 at 25 °C.

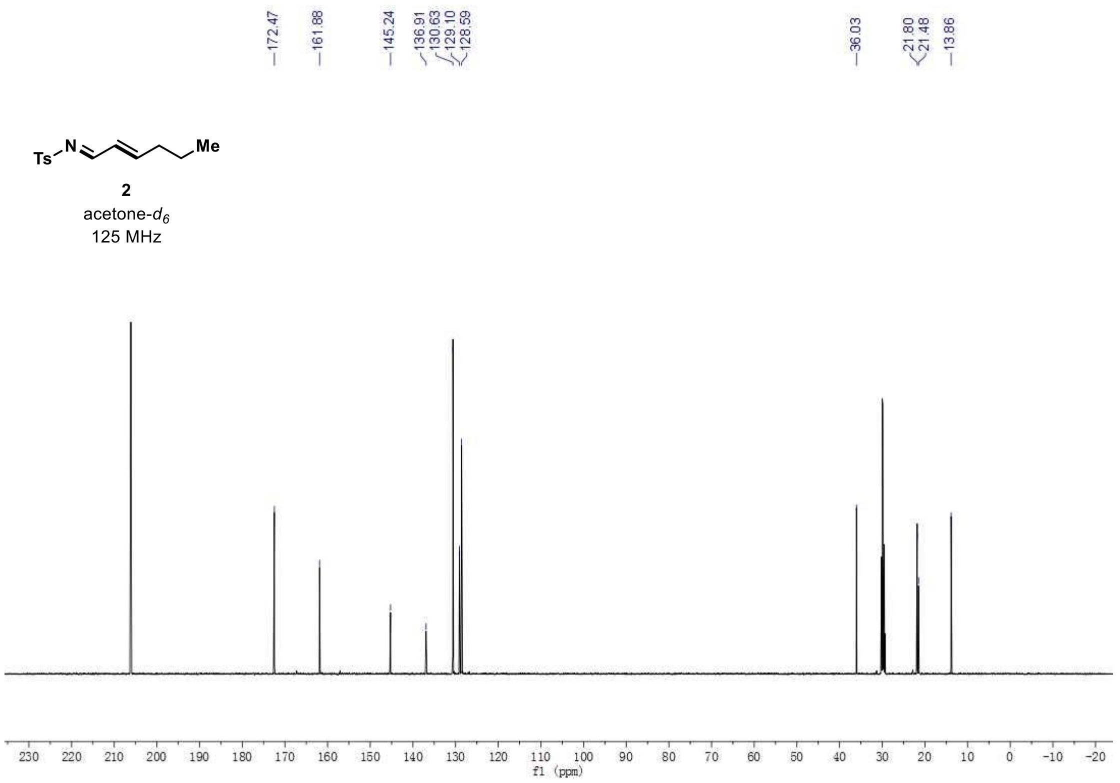

Supplementary Figure 52. ^{13}C NMR of compound 2. The sample has been recorded in 125 MHz, acetone-d_6 at 25 °C.

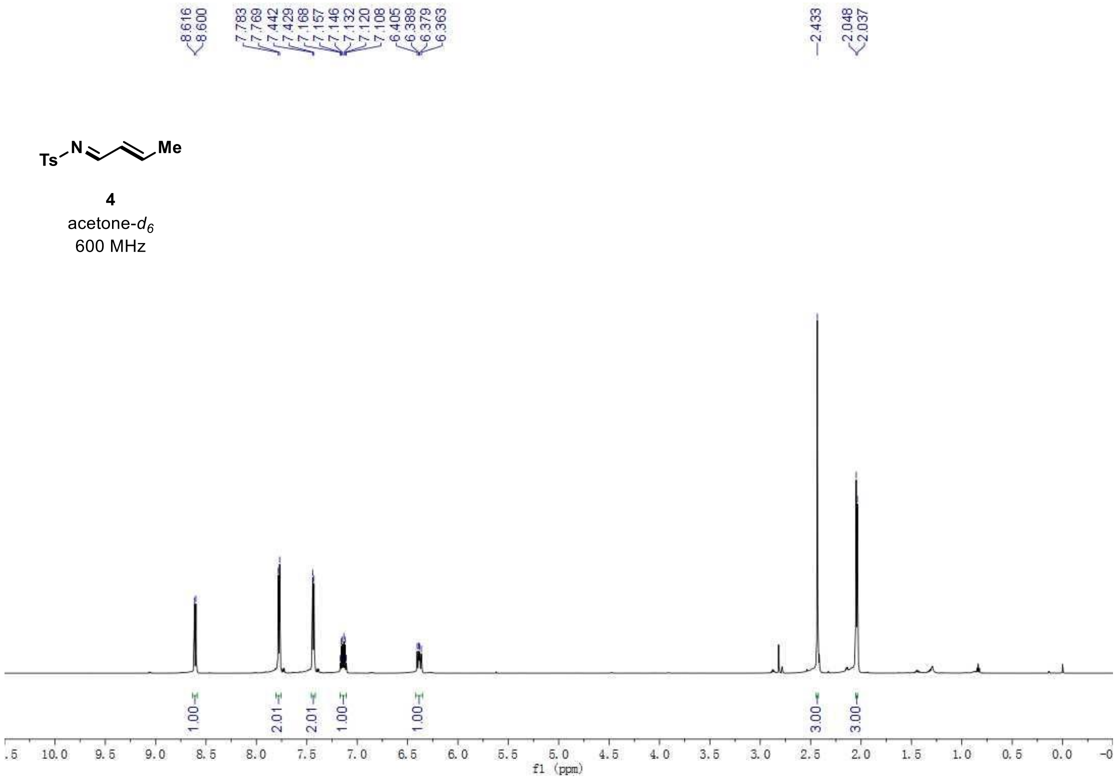

Supplementary Figure 53. ^{1}H NMR of compound 4. The sample has been recorded in 600 MHz, acetone-d_6 at 25 °C.

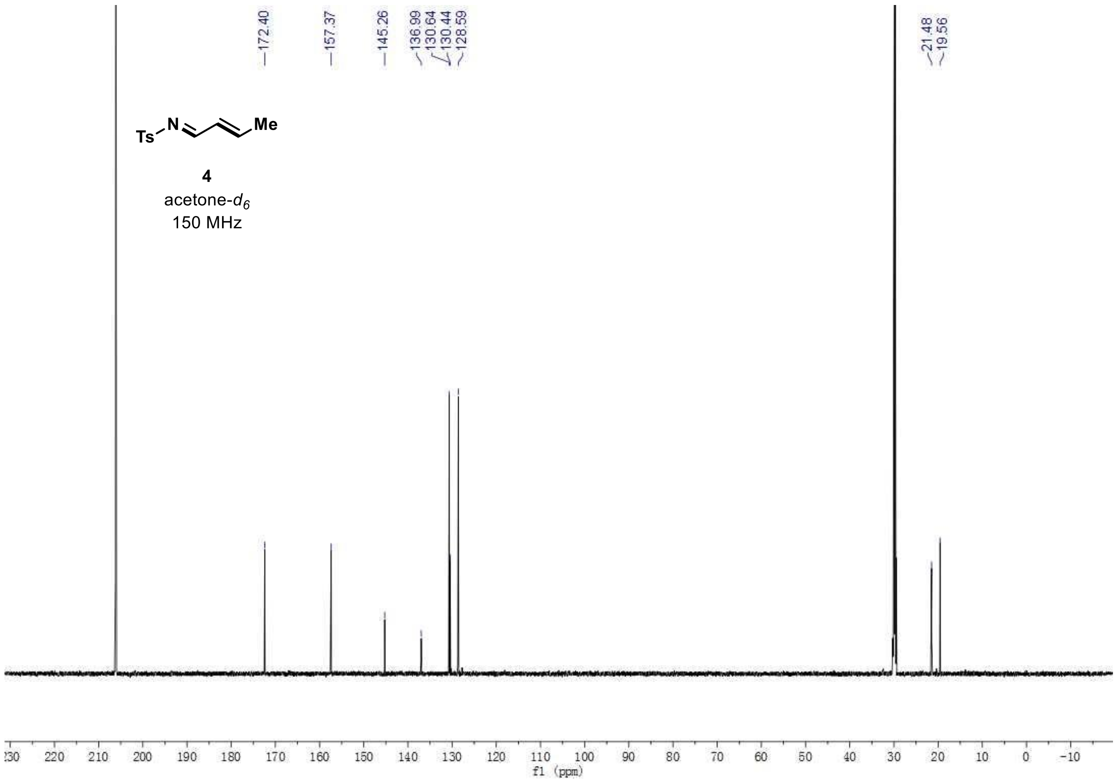

Supplementary Figure 54. ^{13}C NMR of compound 4. The sample has been recorded in 150 MHz, acetone-d_6 at 25 °C.

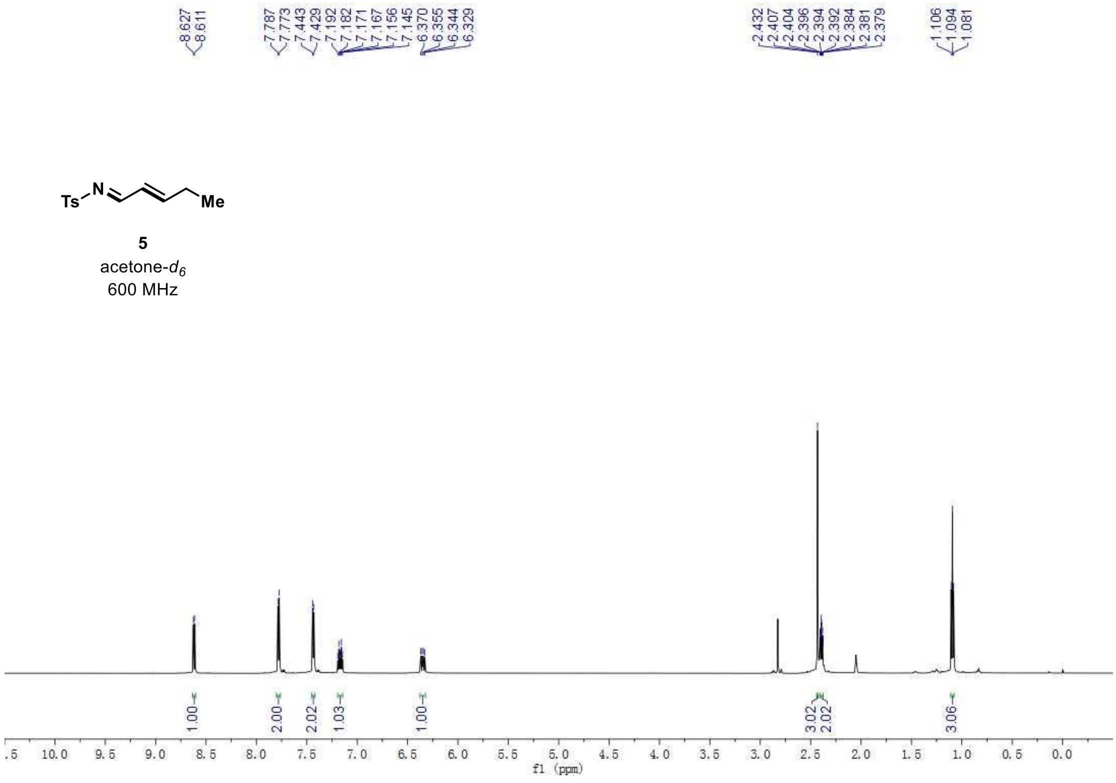

Supplementary Figure 55. ^{1}H NMR of compound 5. The sample has been recorded in 600 MHz, acetone-d_6 at 25 °C.

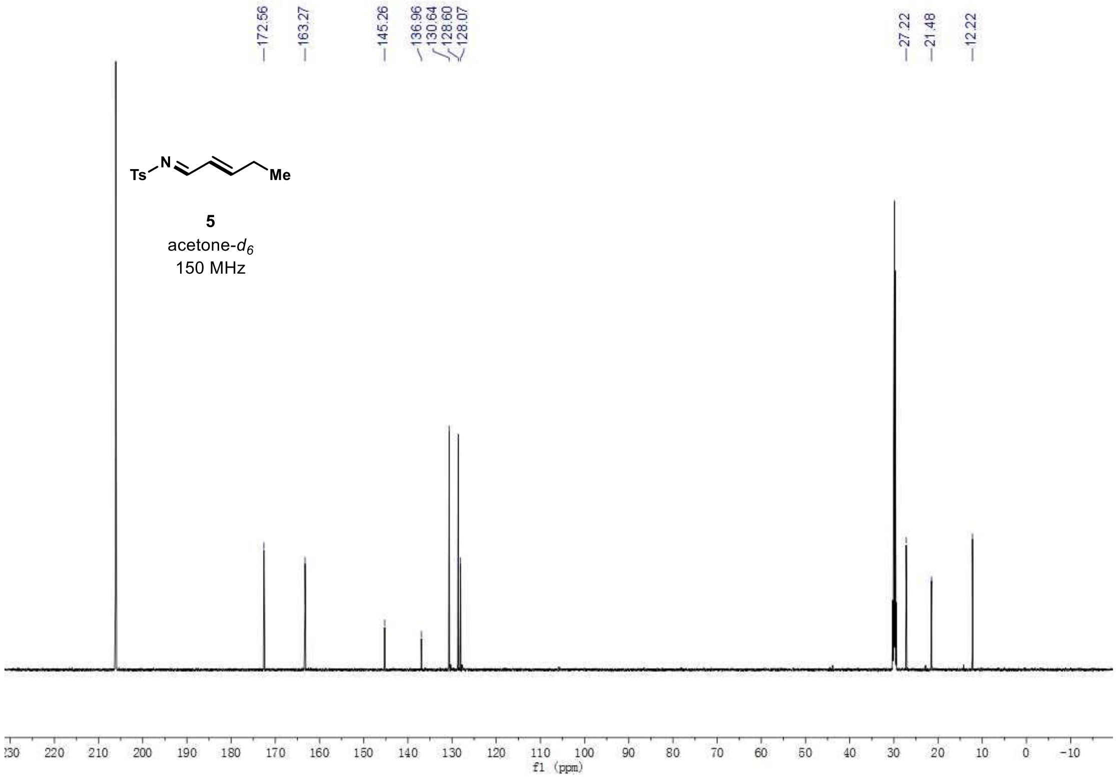

Supplementary Figure 56. ^{13}C NMR of compound 5. The sample has been recorded in 150 MHz, acetone-d_6 at 25 °C.

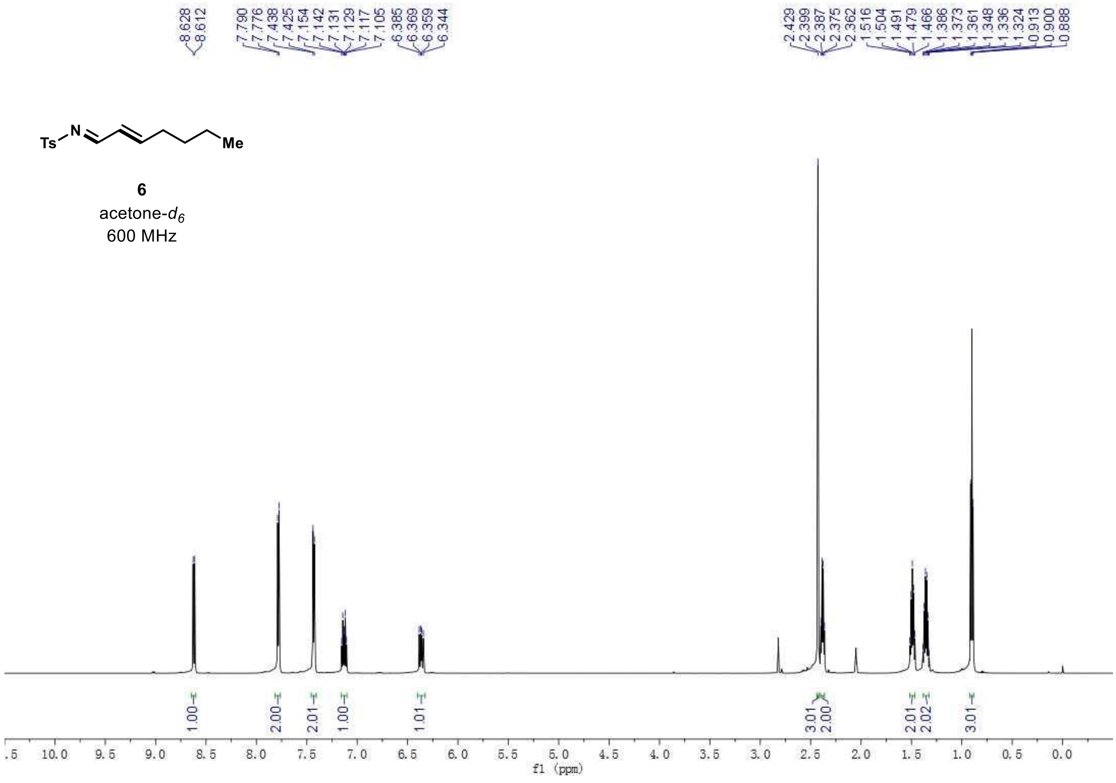

Supplementary Figure 57. ^{1}H NMR of compound 6. The sample has been recorded in 600 MHz, acetone-d_6 at 25 °C.

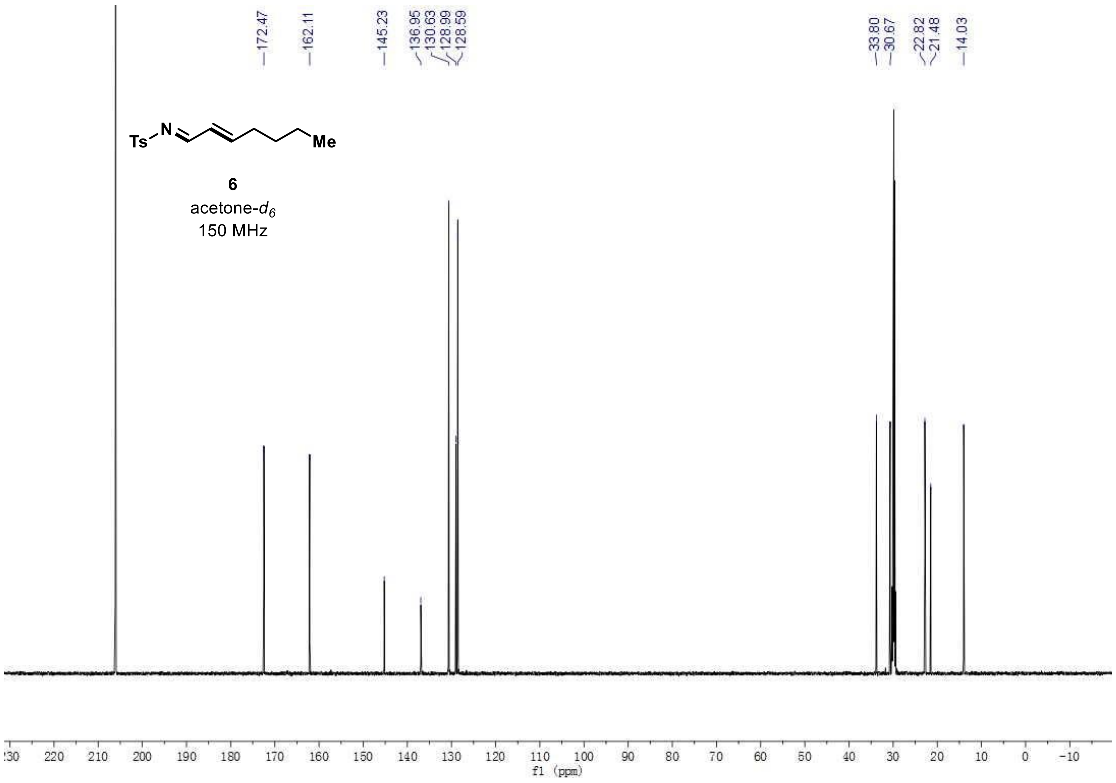

Supplementary Figure 58. ^{13}C NMR of compound 6. The sample has been recorded in 150 MHz, acetone-d_6 at 25 °C.

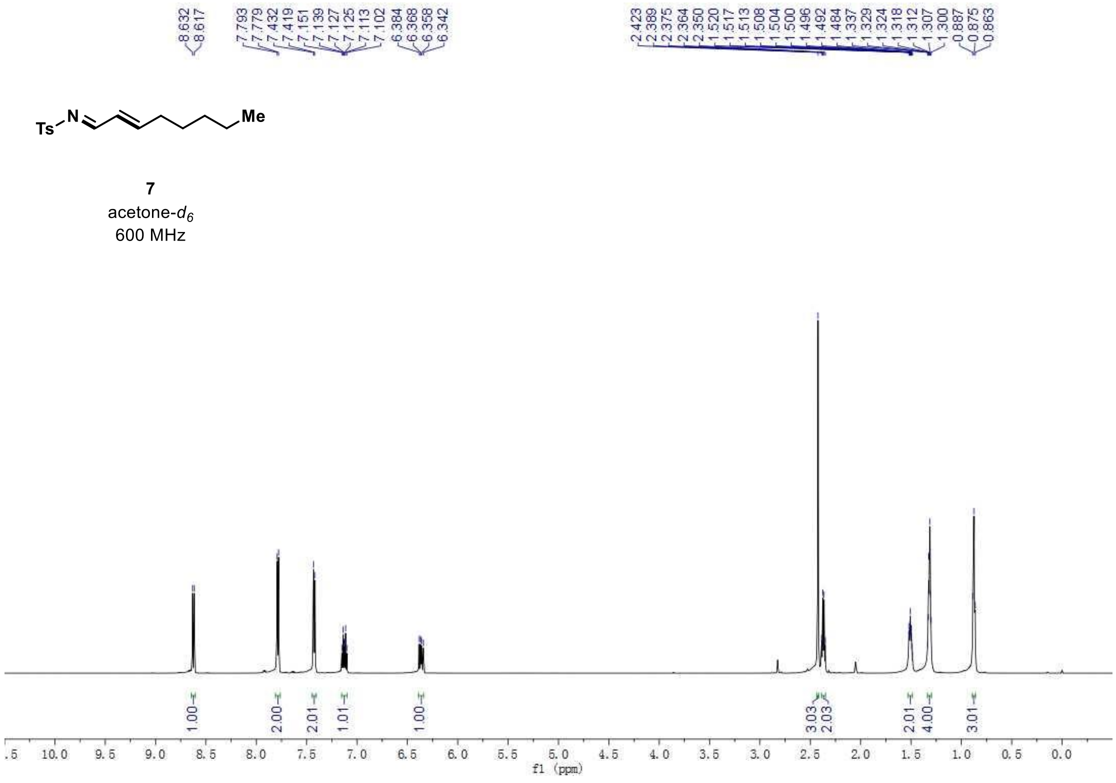

Supplementary Figure 59. ^{1}H NMR of compound 7. The sample has been recorded in 600 MHz, acetone-d_6 at 25 °C.

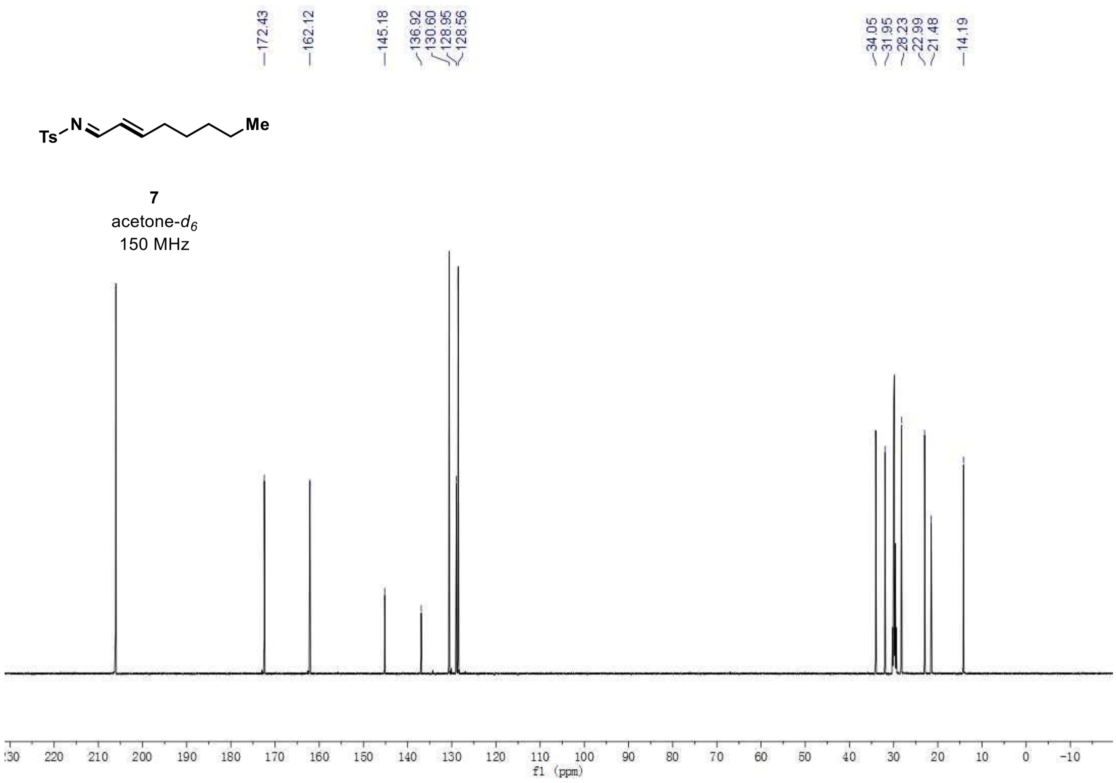

Supplementary Figure 60. ^{13}C NMR of compound 7. The sample has been recorded in 150 MHz, acetone-d_6 at 25 °C.

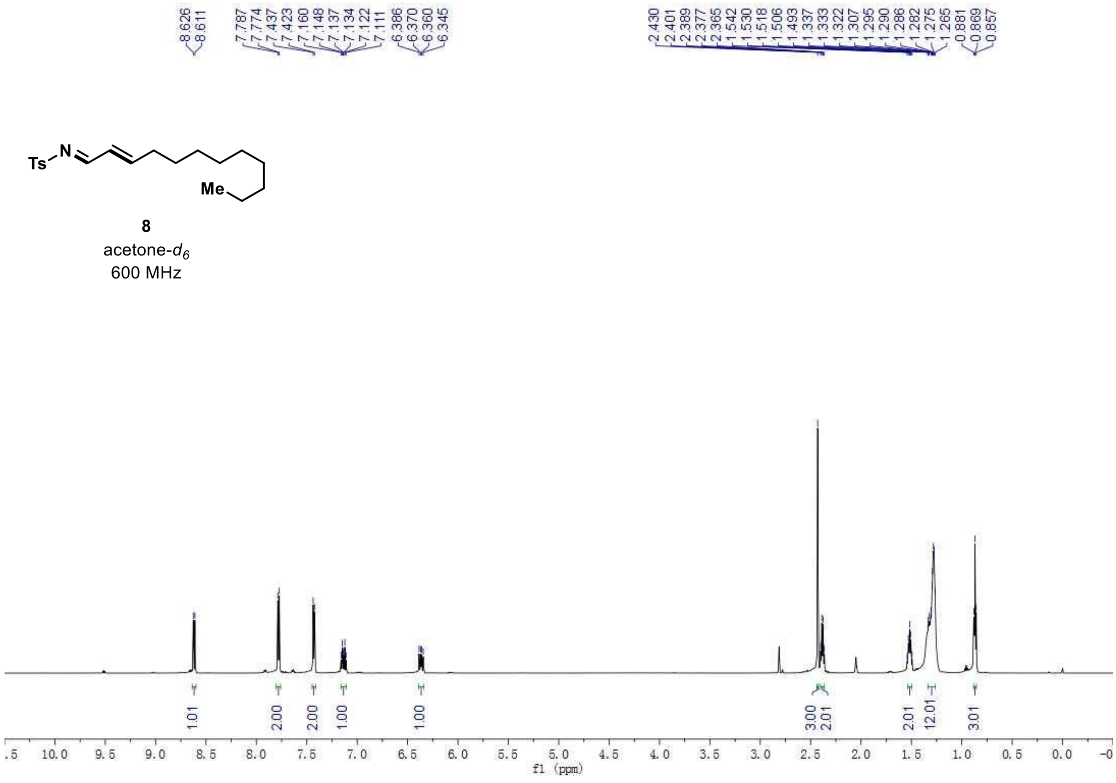

Supplementary Figure 61. ^{1}H NMR of compound 8. The sample has been recorded in 600 MHz, acetone-d_6 at 25 °C.

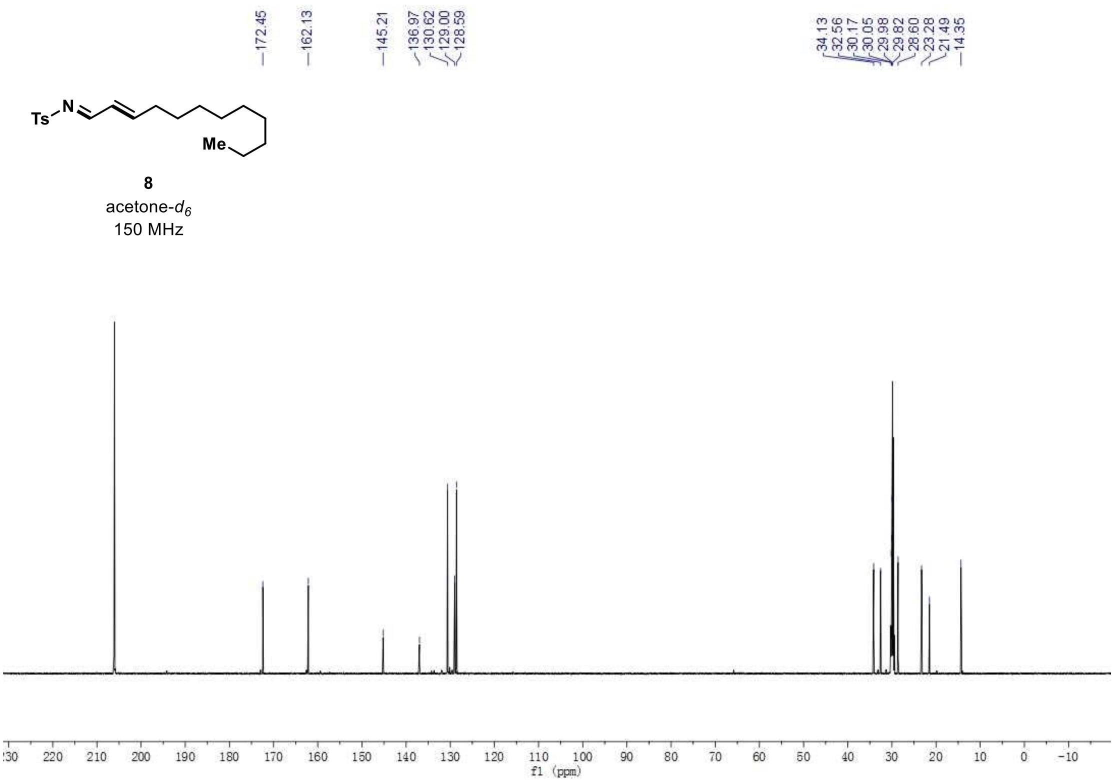

Supplementary Figure 62. ^{13}C NMR of compound 8. The sample has been recorded in 150 MHz, acetone-d_6 at 25 °C.

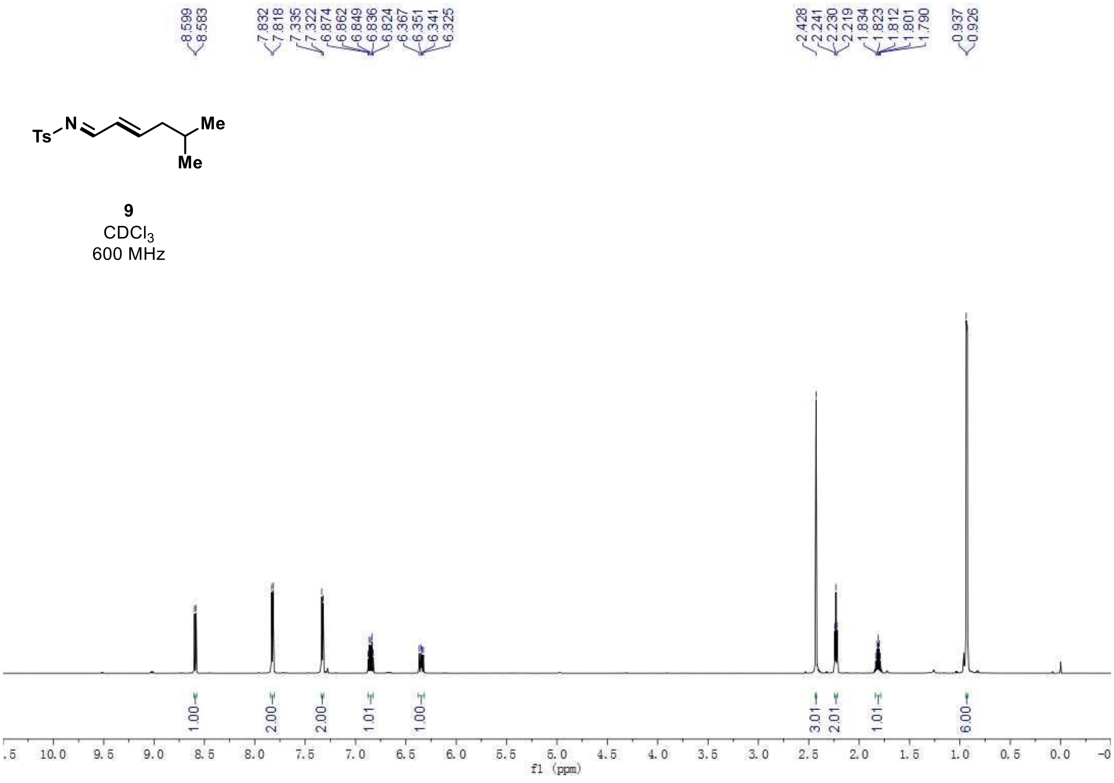

Supplementary Figure 63. ^{1}H NMR of compound 9. The sample has been recorded in 600 MHz, $CDCl_3$ at 25 °C.

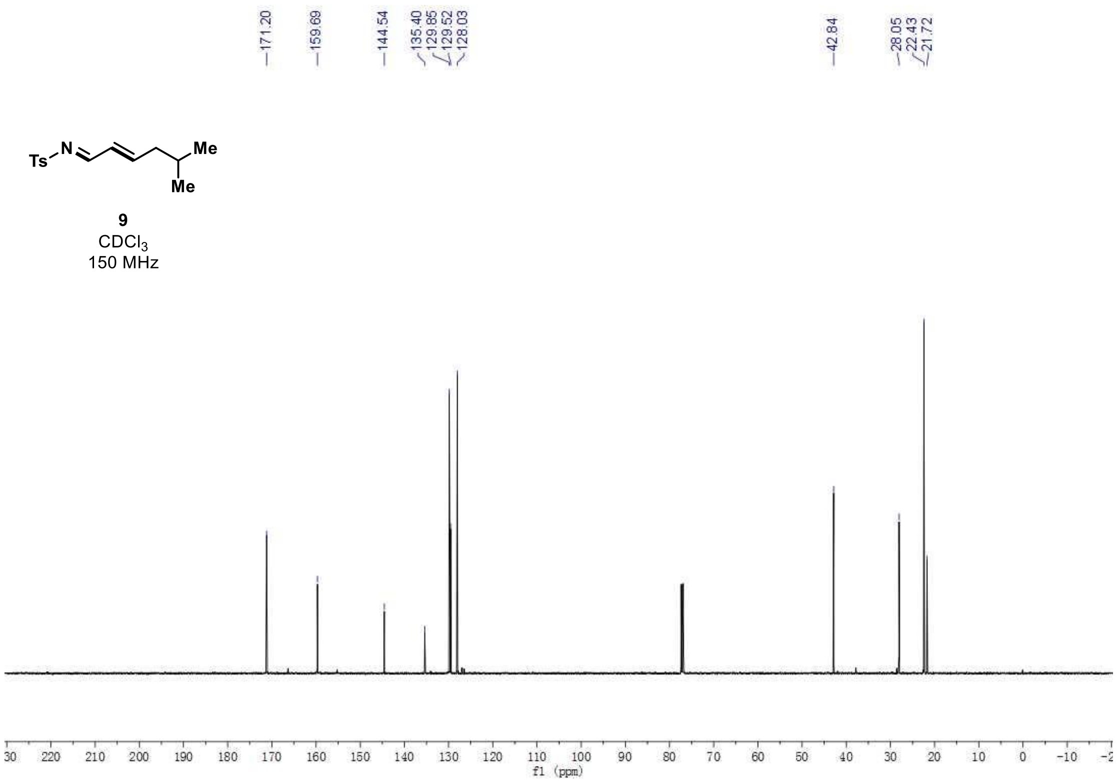

Supplementary Figure 64. ^{13}C NMR of compound 9. The sample has been recorded in 150 MHz, $CDCl_3$ at 25 °C.

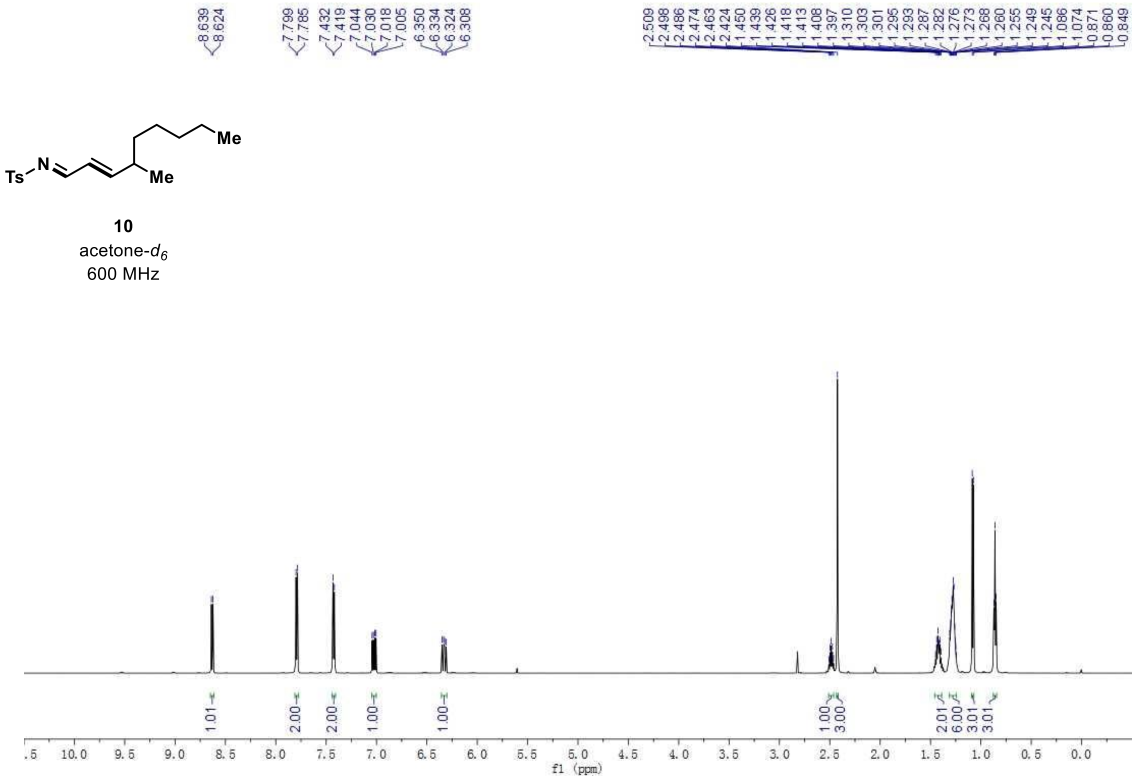

Supplementary Figure 65. ^{1}H NMR of compound 10. The sample has been recorded in 600 MHz, acetone-d_6 at 25 °C.

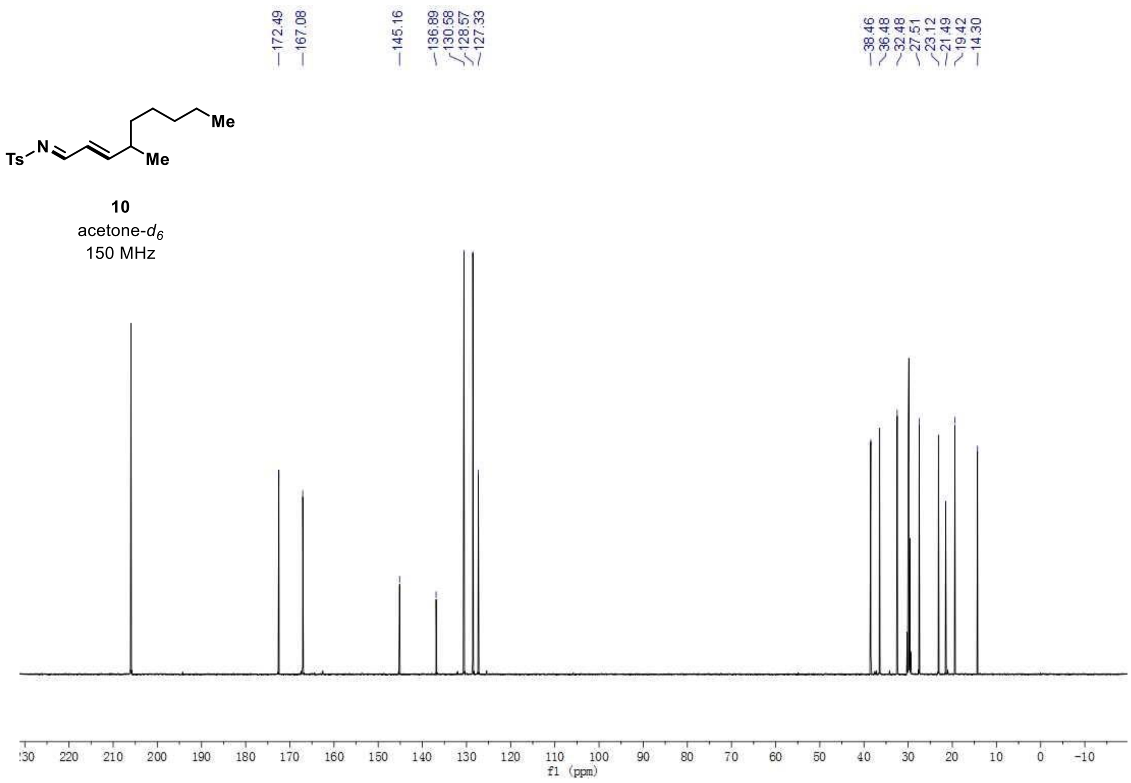

Supplementary Figure 66. ^{13}C NMR of compound 10. The sample has been recorded in 150 MHz, acetone-d_6 at 25 °C.

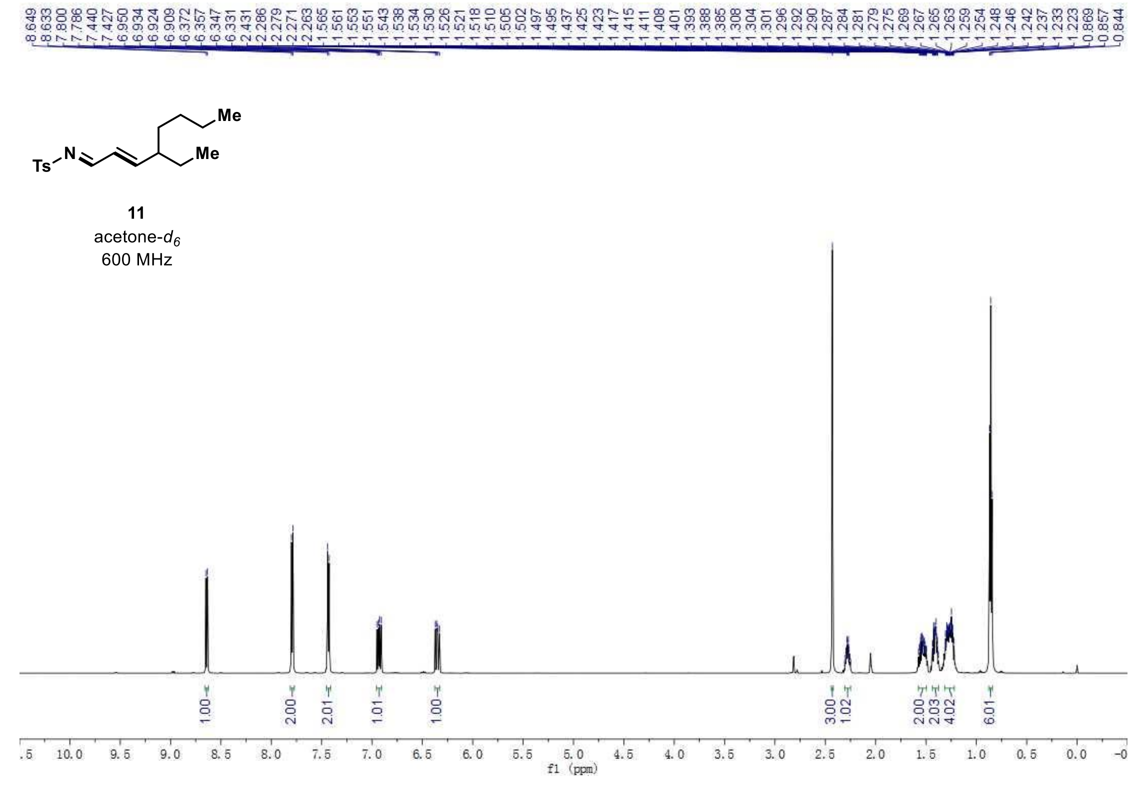

Supplementary Figure 67. ^{1}H NMR of compound 11. The sample has been recorded in 600 MHz, acetone-d_6 at 25 °C.

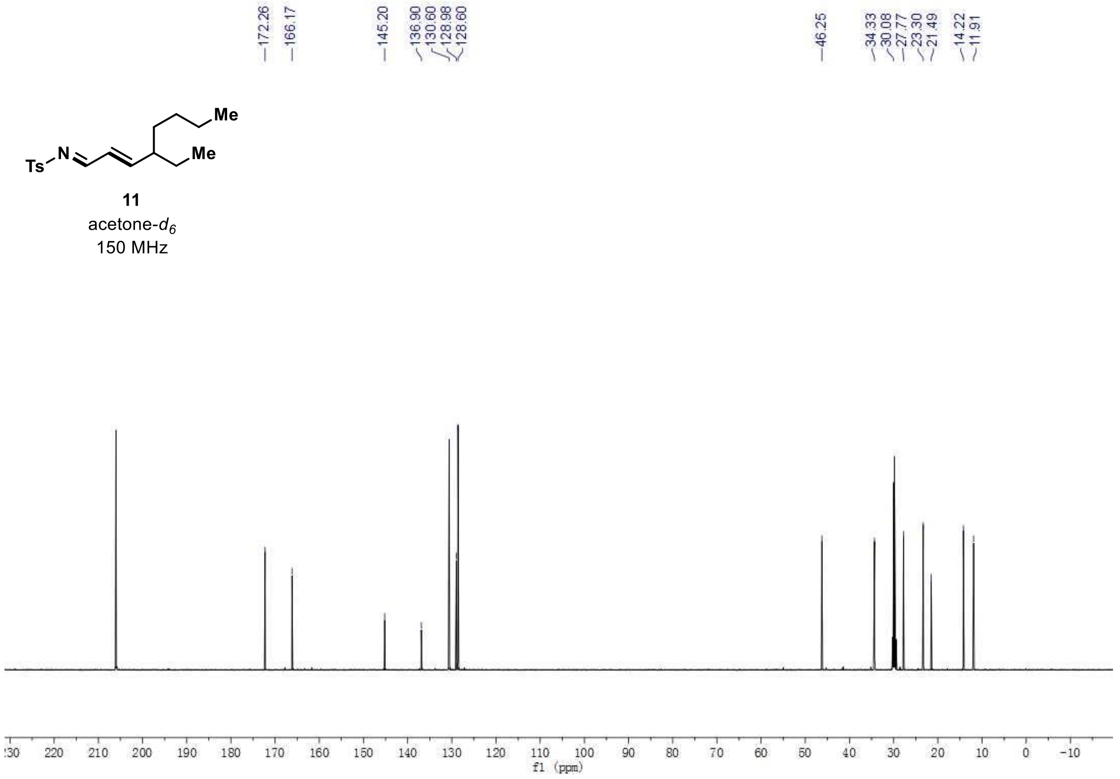

Supplementary Figure 68. ^{13}C NMR of compound 11. The sample has been recorded in 150 MHz, acetone-*d*$_6$ at 25 °C.

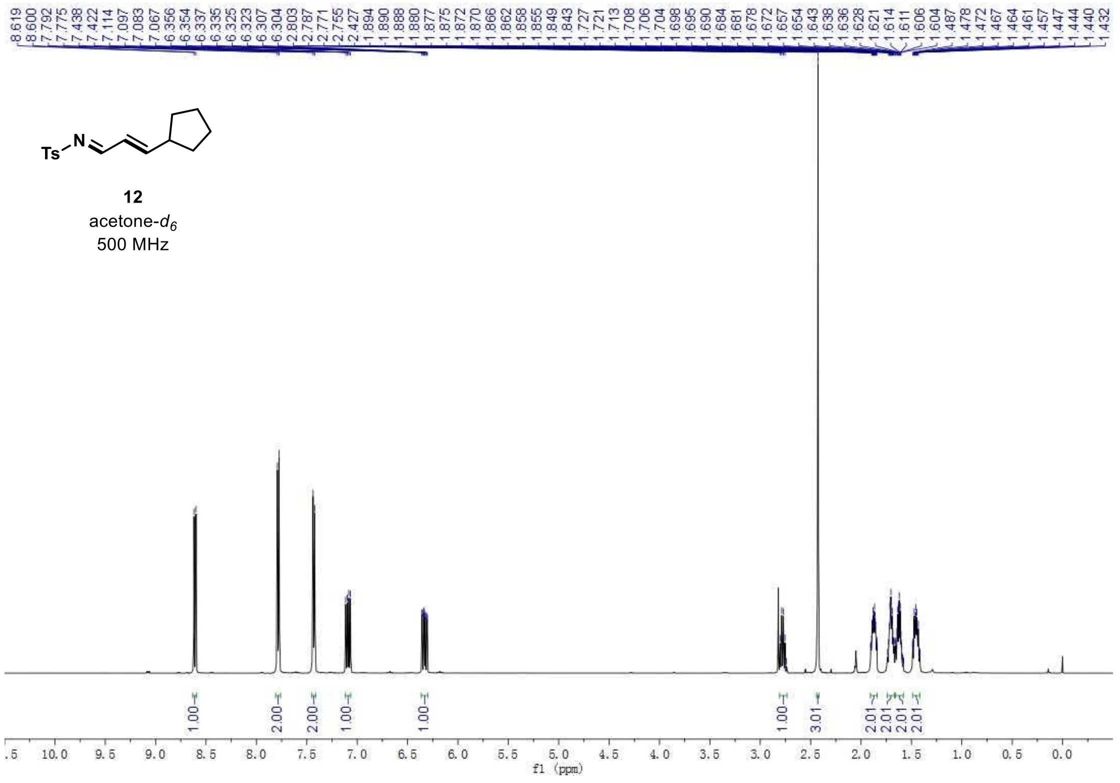

Supplementary Figure 69. ^{1}H NMR of compound 12. The sample has been recorded in 500 MHz, acetone-*d*$_6$ at 25 °C.

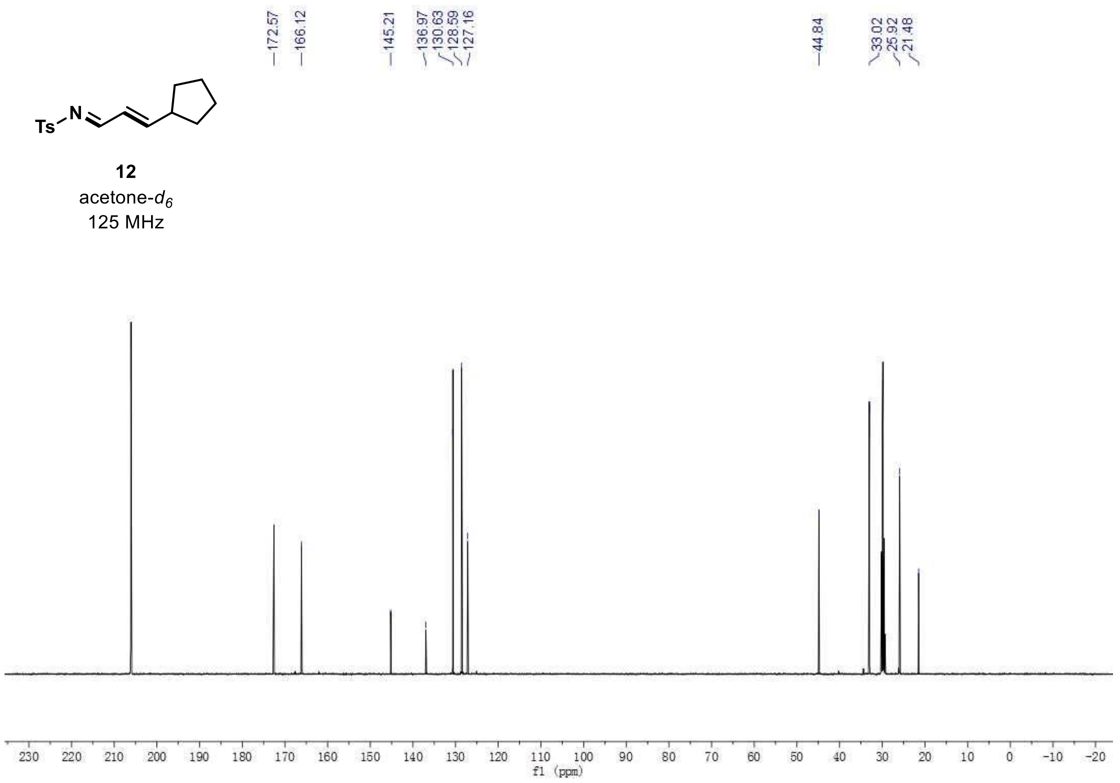

Supplementary Figure 70. ^{13}C NMR of compound 12. The sample has been recorded in 125 MHz, acetone-d_6 at 25 °C.

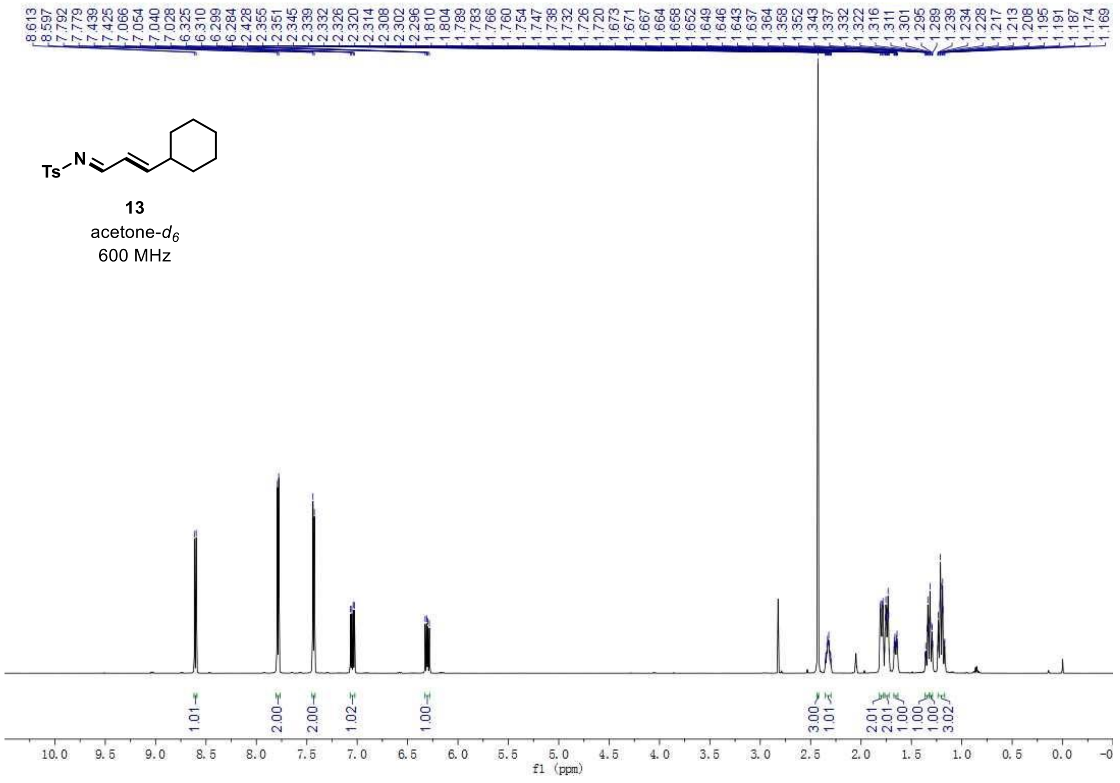

Supplementary Figure 71. ^{1}H NMR of compound 13. The sample has been recorded in 600 MHz, acetone-d_6 at 25 °C.

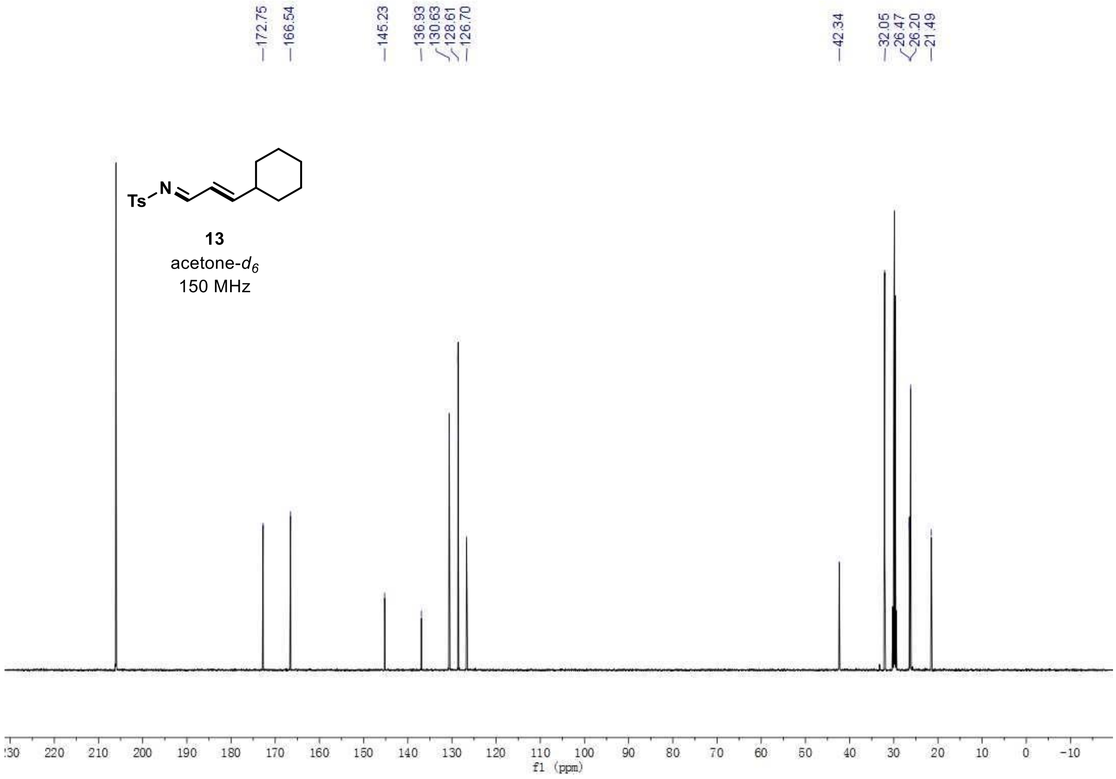

Supplementary Figure 72. ^{13}C NMR of compound 13. The sample has been recorded in 150 MHz, acetone-d_6 at 25 °C.

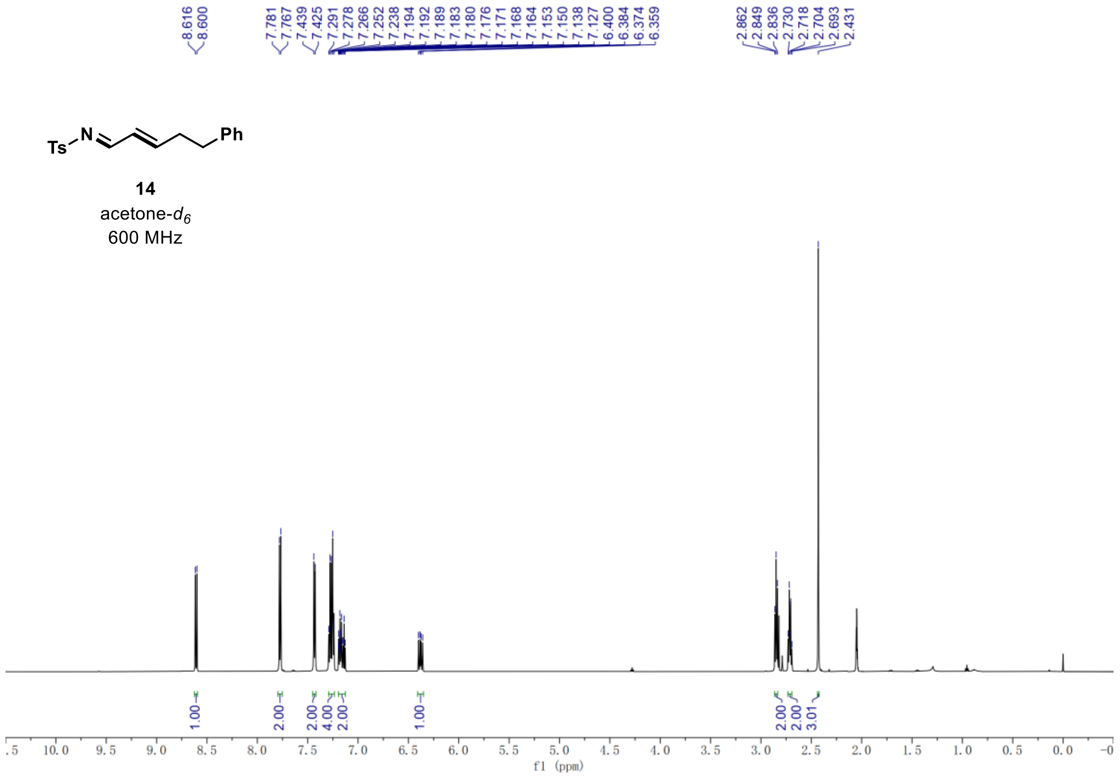

Supplementary Figure 73. ^{1}H NMR of compound 14. The sample has been recorded in 600 MHz, acetone-d_6 at 25 °C.

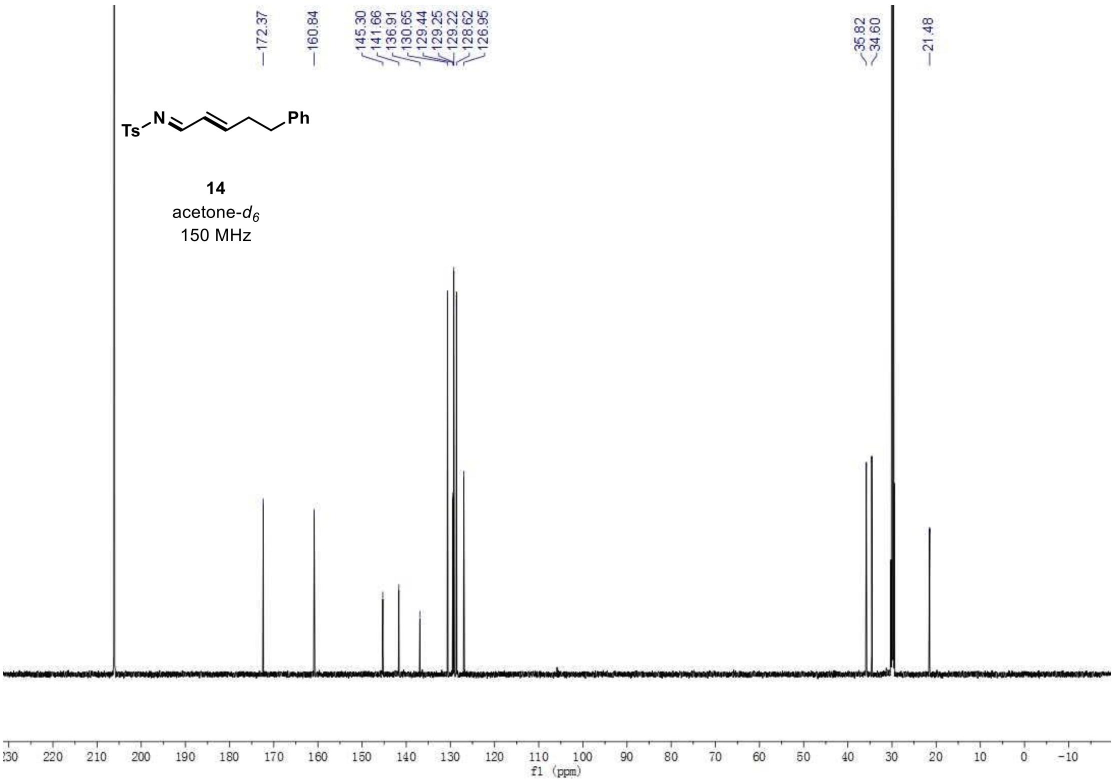

Supplementary Figure 74. ^{13}C NMR of compound 14. The sample has been recorded in 150 MHz, acetone-d_6 at 25 °C.

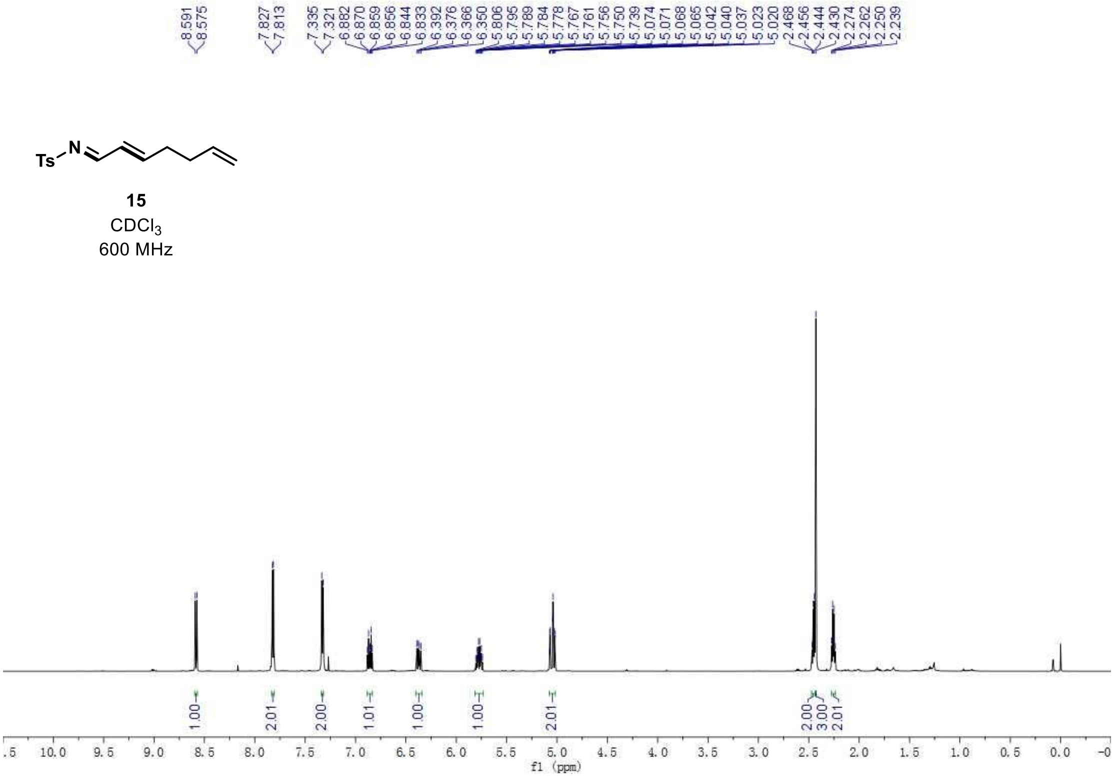

Supplementary Figure 75. ^{1}H NMR of compound 15. The sample has been recorded in 600 MHz, $CDCl_3$ at 25 °C.

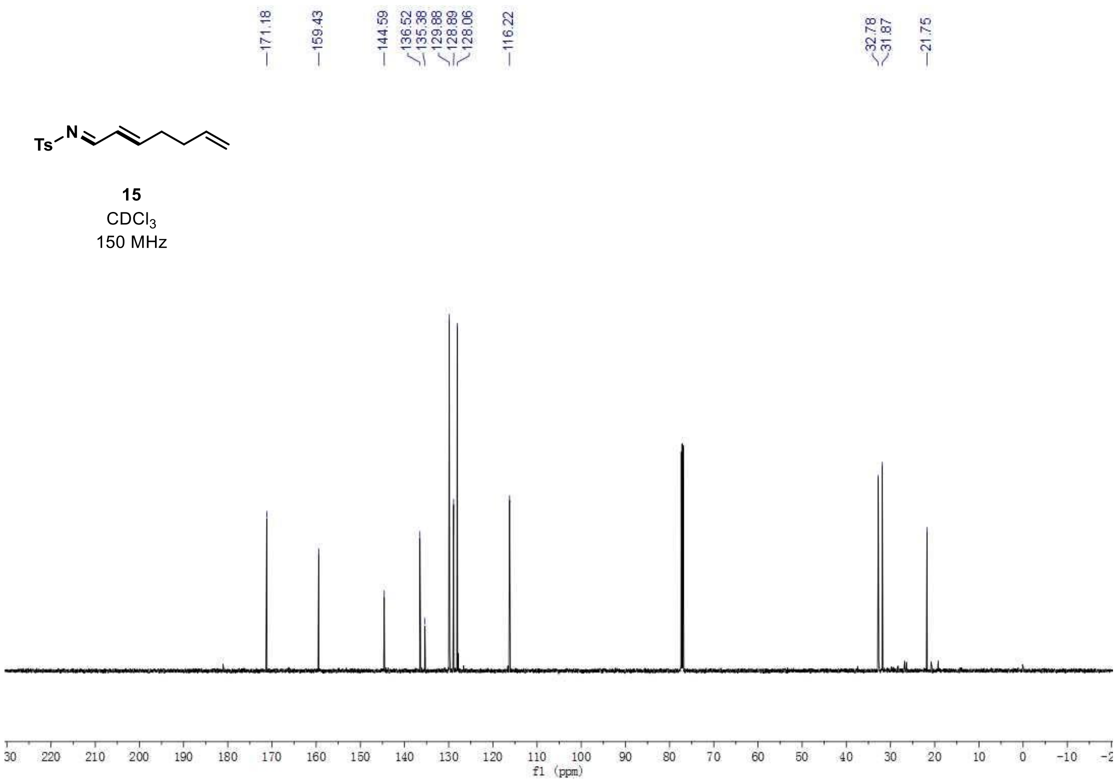

Supplementary Figure 76. ^{13}C NMR of compound 15. The sample has been recorded in 150 MHz, $CDCl_3$ at 25 °C.

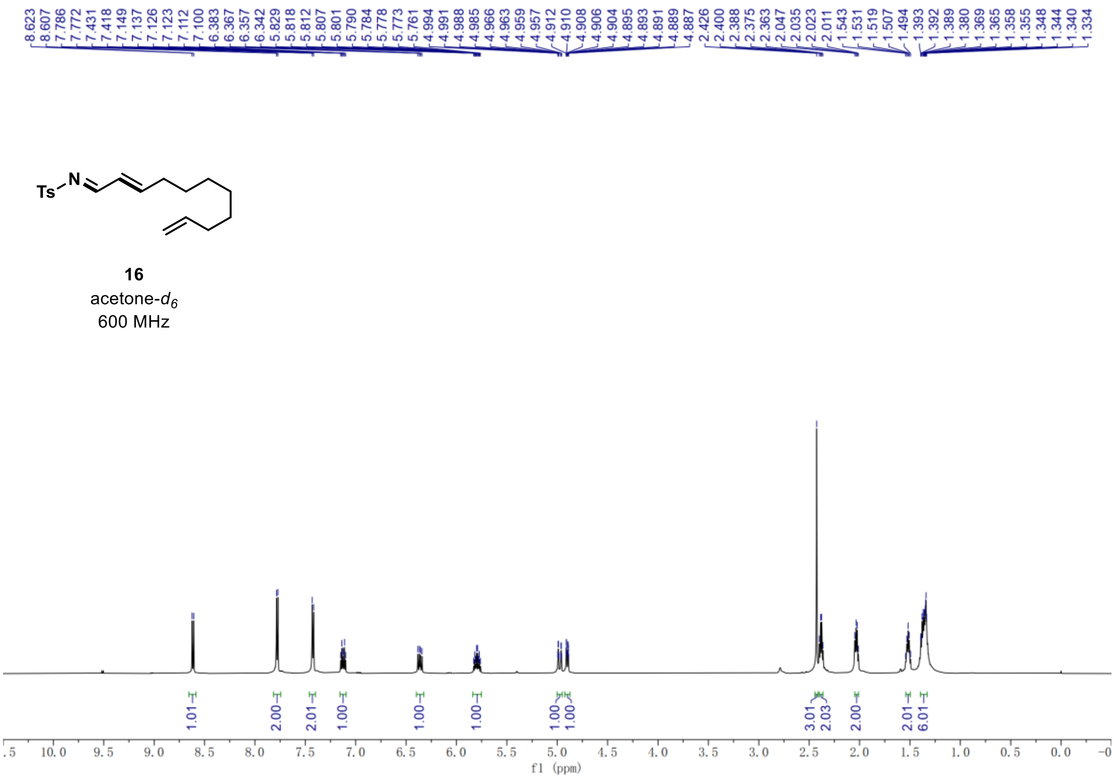

Supplementary Figure 77. ^{1}H NMR of compound 16. The sample has been recorded in 600 MHz, acetone-d_6 at 25 °C.

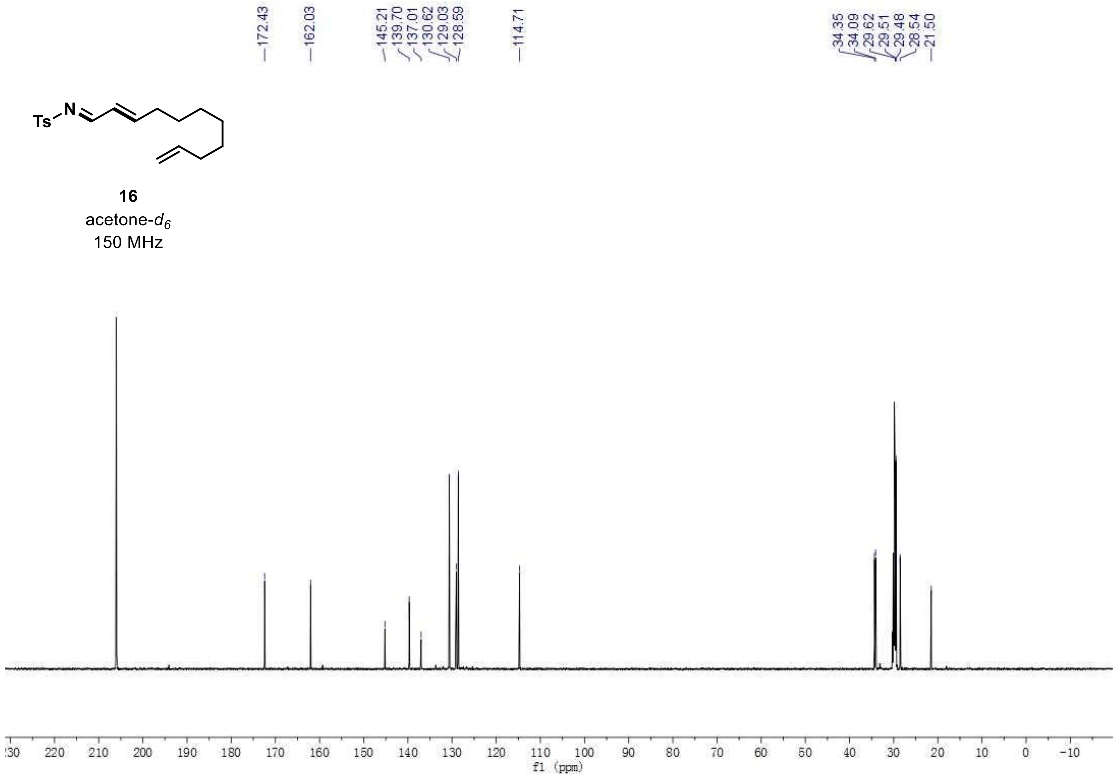

Supplementary Figure 78. ^{13}C NMR of compound 16. The sample has been recorded in 150 MHz, acetone-d_6 at 25 °C.

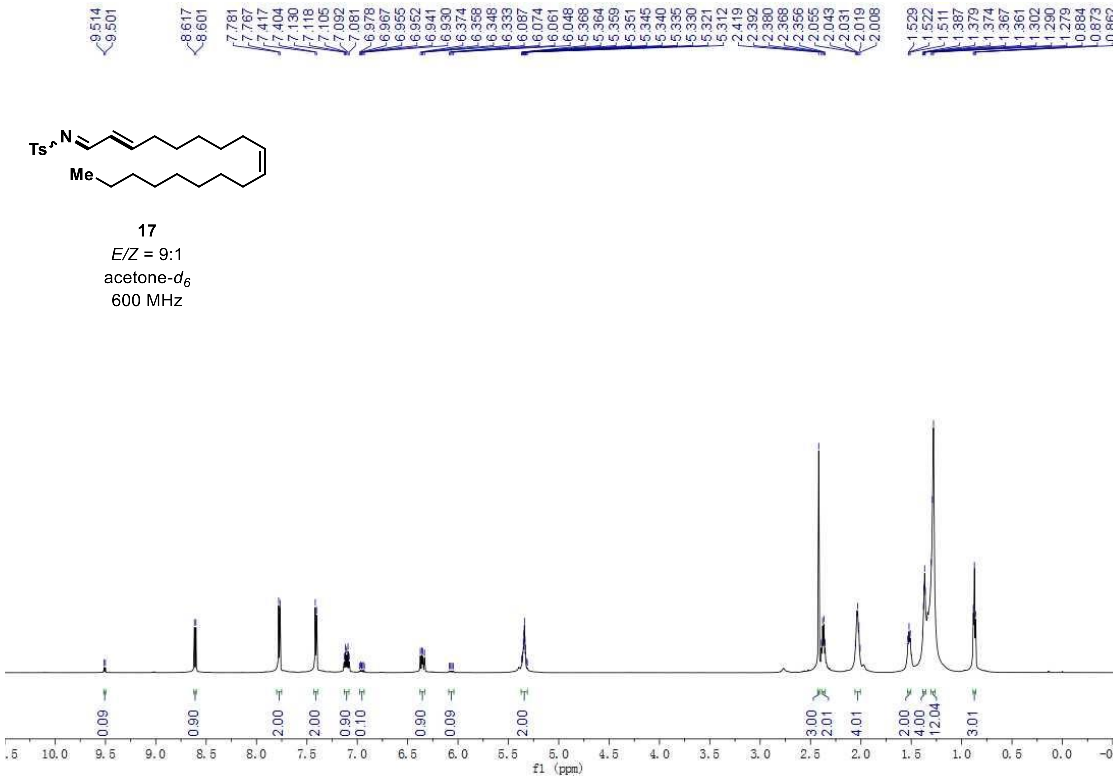

Supplementary Figure 79. ^{1}H NMR of compound 17. The sample has been recorded in 600 MHz, acetone-d_6 at 25 °C.

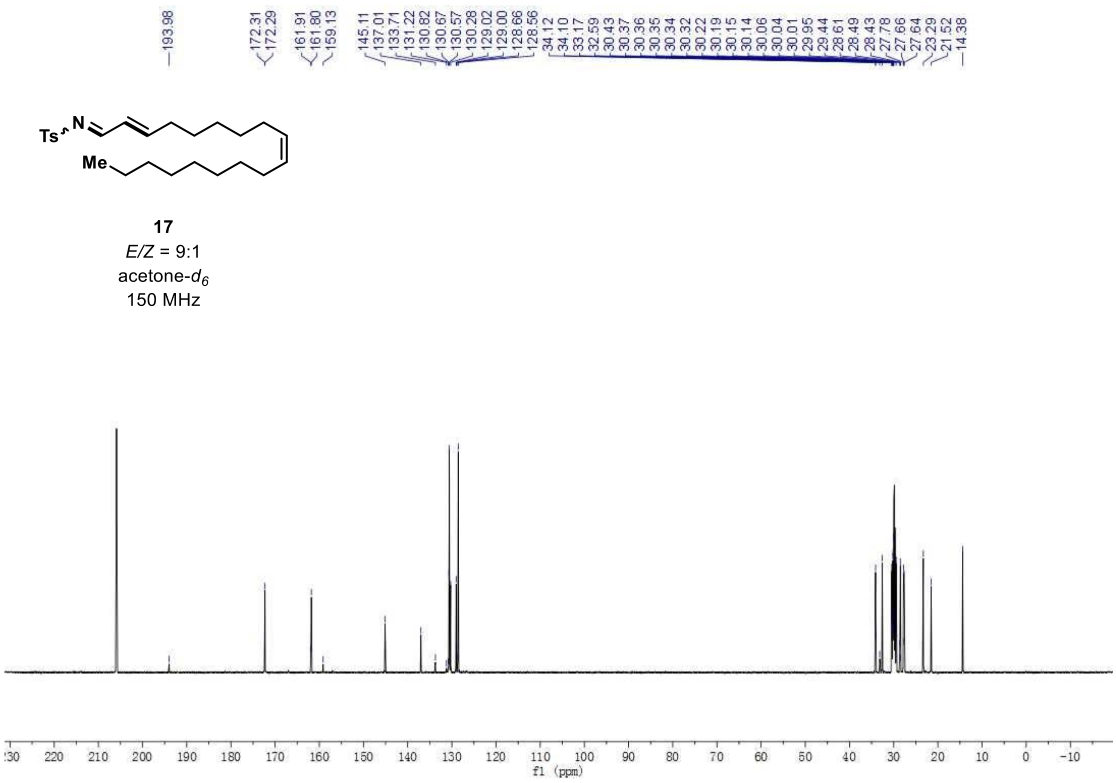

Supplementary Figure 80. ^{13}C NMR of compound 17. The sample has been recorded in 150 MHz, acetone-d_6 at 25 °C.

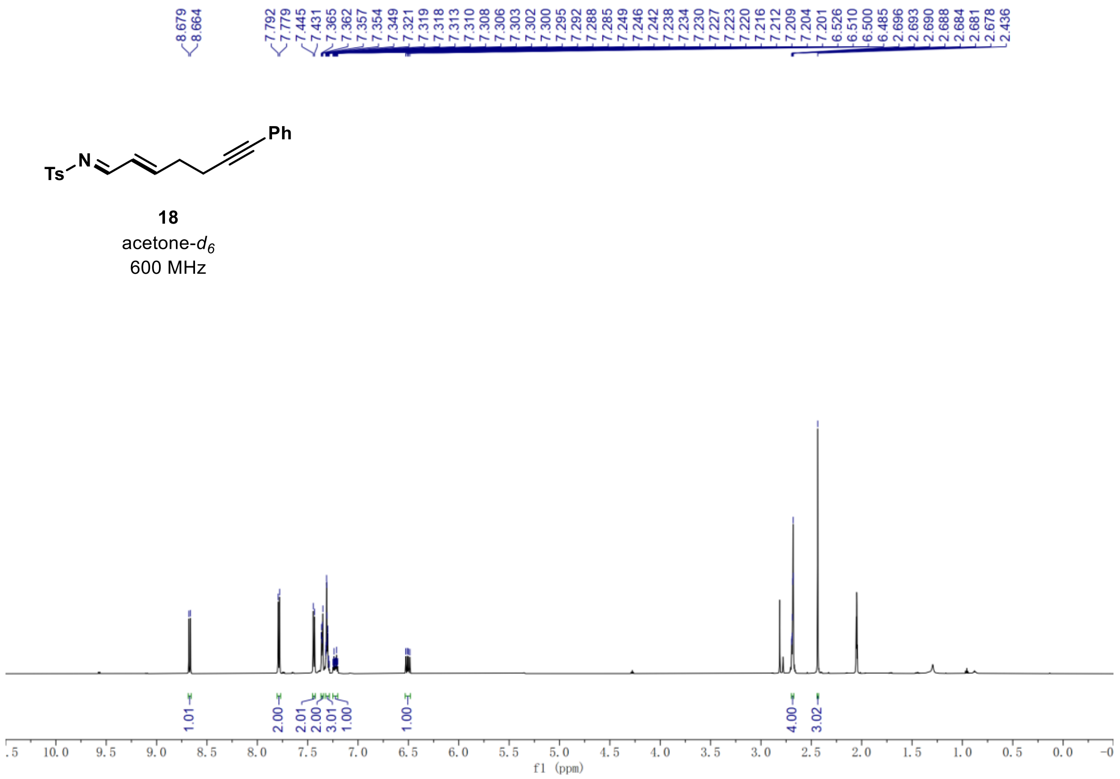

Supplementary Figure 81. ^{1}H NMR of compound 18. The sample has been recorded in 600 MHz, acetone-d_6 at 25 °C.

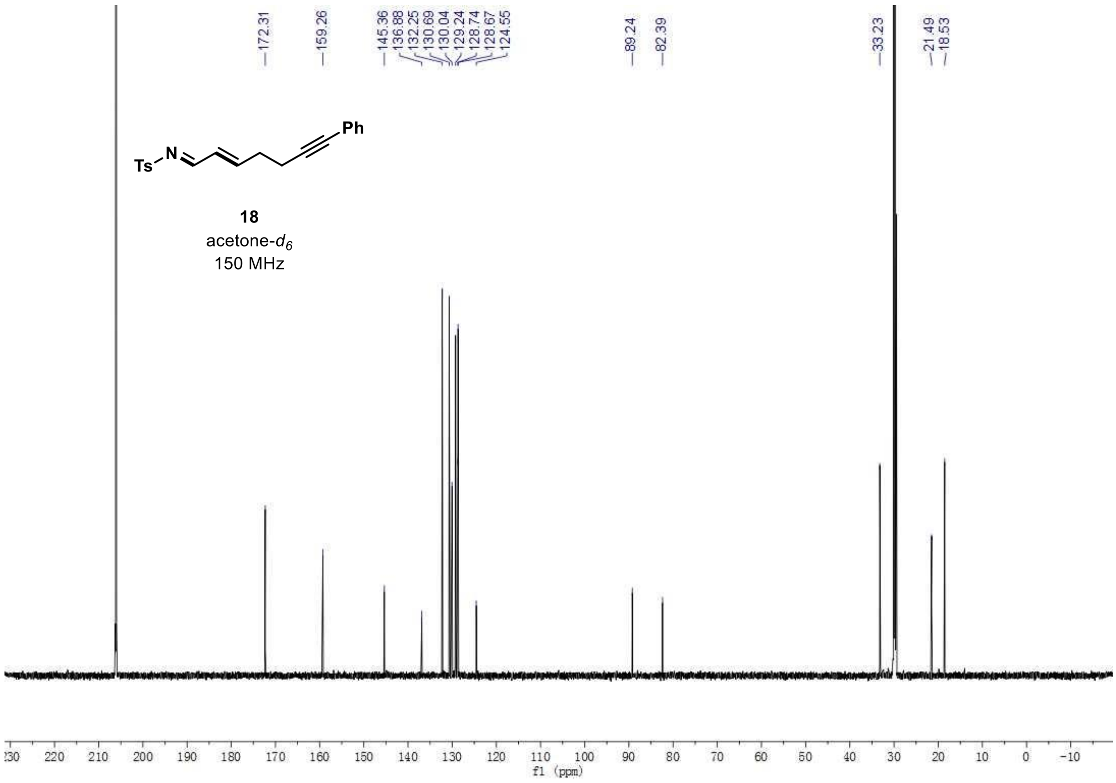

Supplementary Figure 82. ^{13}C NMR of compound 18. The sample has been recorded in 150 MHz, acetone-d_6 at 25 °C.

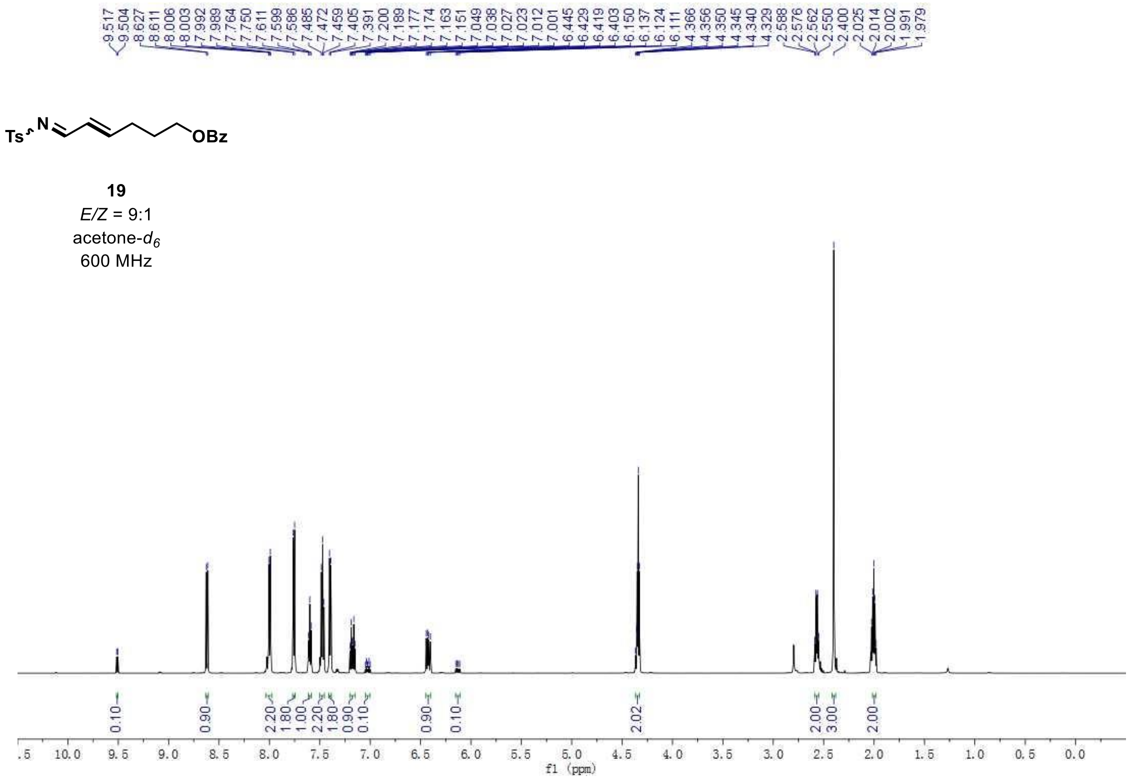

Supplementary Figure 83. ^{1}H NMR of compound 19. The sample has been recorded in 600 MHz, acetone-d_6 at 25 °C.

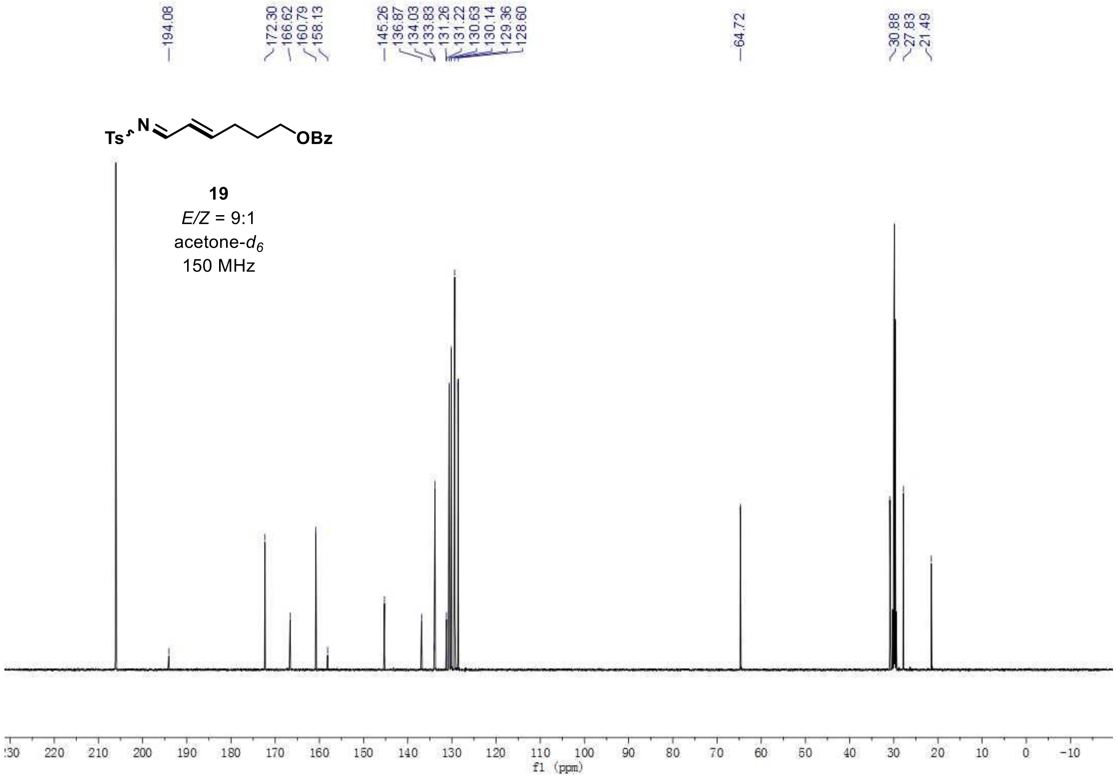

Supplementary Figure 84. ^{13}C NMR of compound 19. The sample has been recorded in 150 MHz, acetone-d_6 at 25 °C.

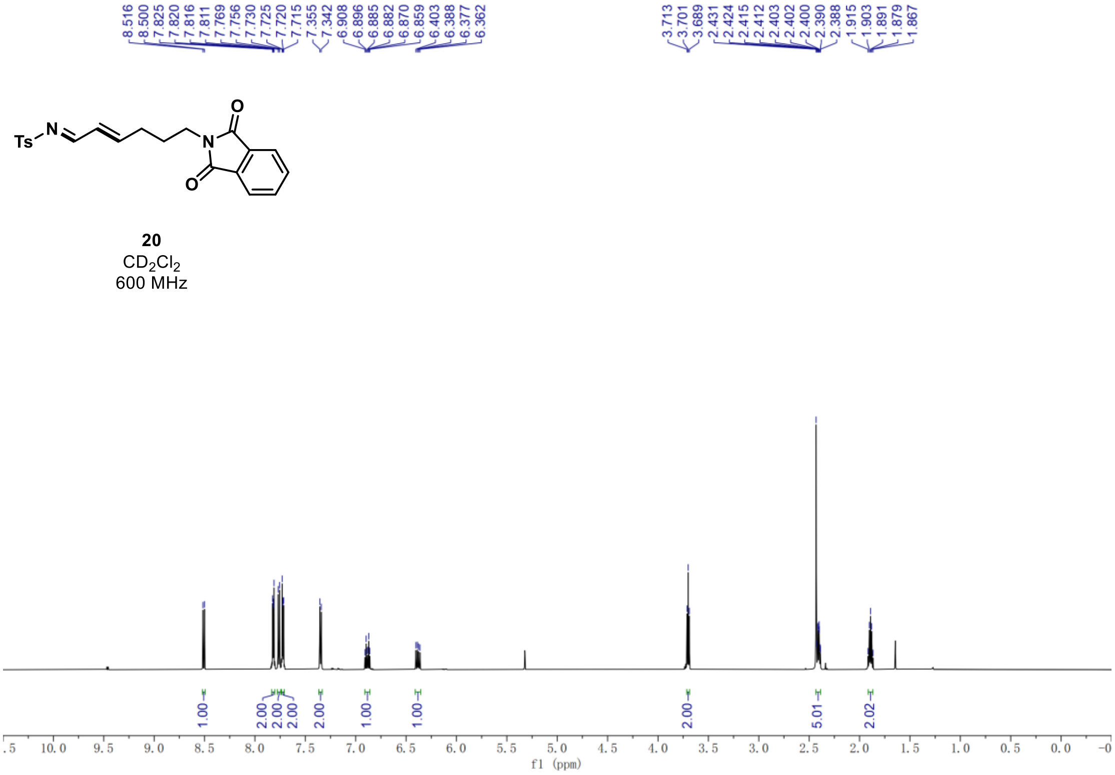

Supplementary Figure 85. ^{1}H NMR of compound 20. The sample has been recorded in 600 MHz, CD_2Cl_2 at 25 °C.

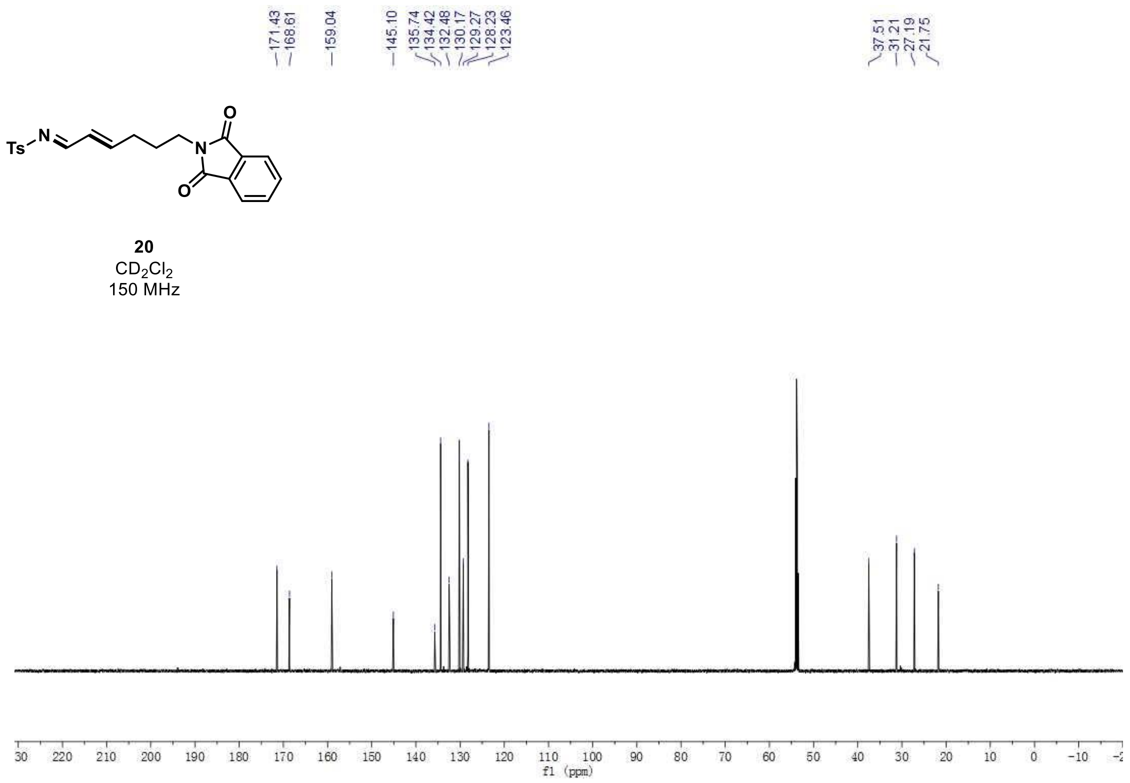

Supplementary Figure 86. ^{13}C NMR of compound 20. The sample has been recorded in 150 MHz, CD_2Cl_2 at 25 °C.

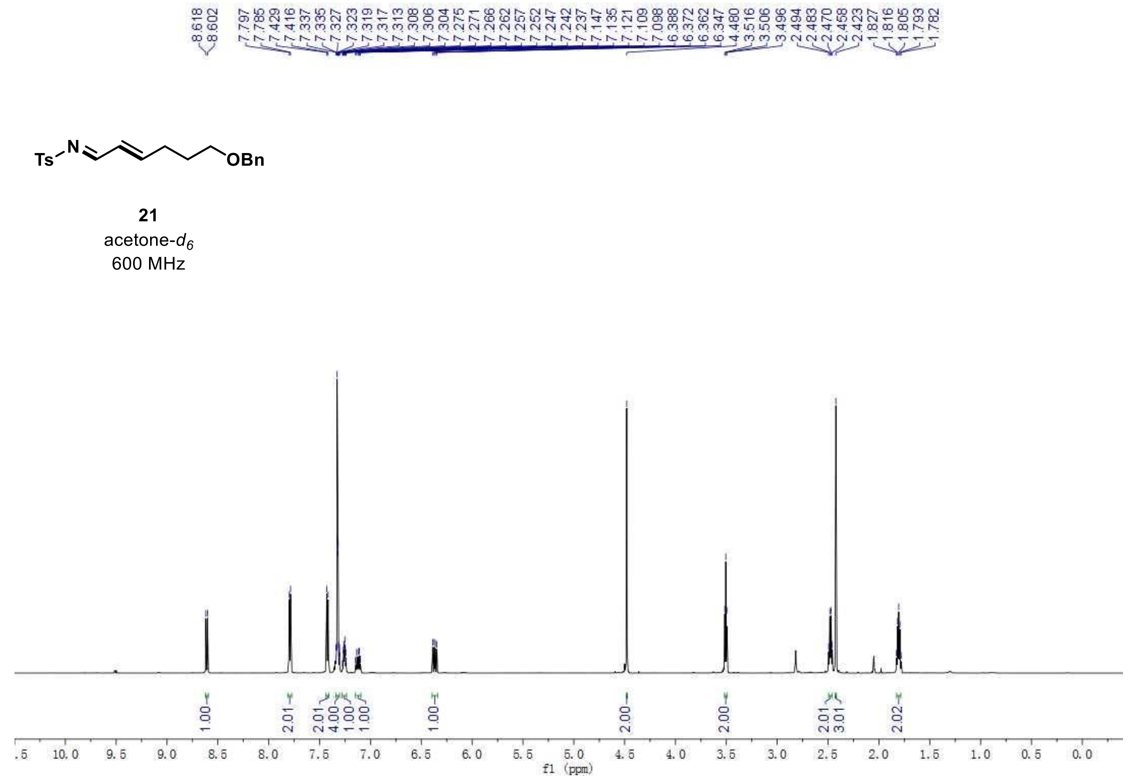

Supplementary Figure 87. ^{1}H NMR of compound 21. The sample has been recorded in 600 MHz, acetone-d_6 at 25 °C.

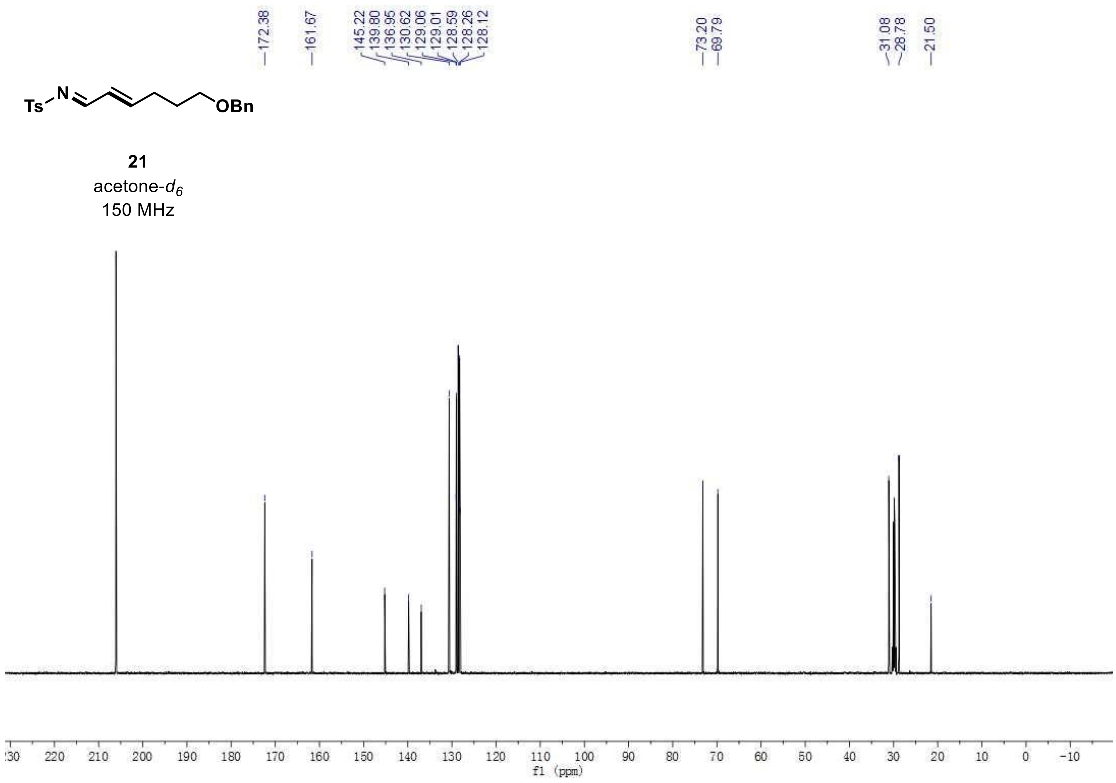

Supplementary Figure 88. ^{13}C NMR of compound 21. The sample has been recorded in 150 MHz, acetone-d_6 at 25 °C.

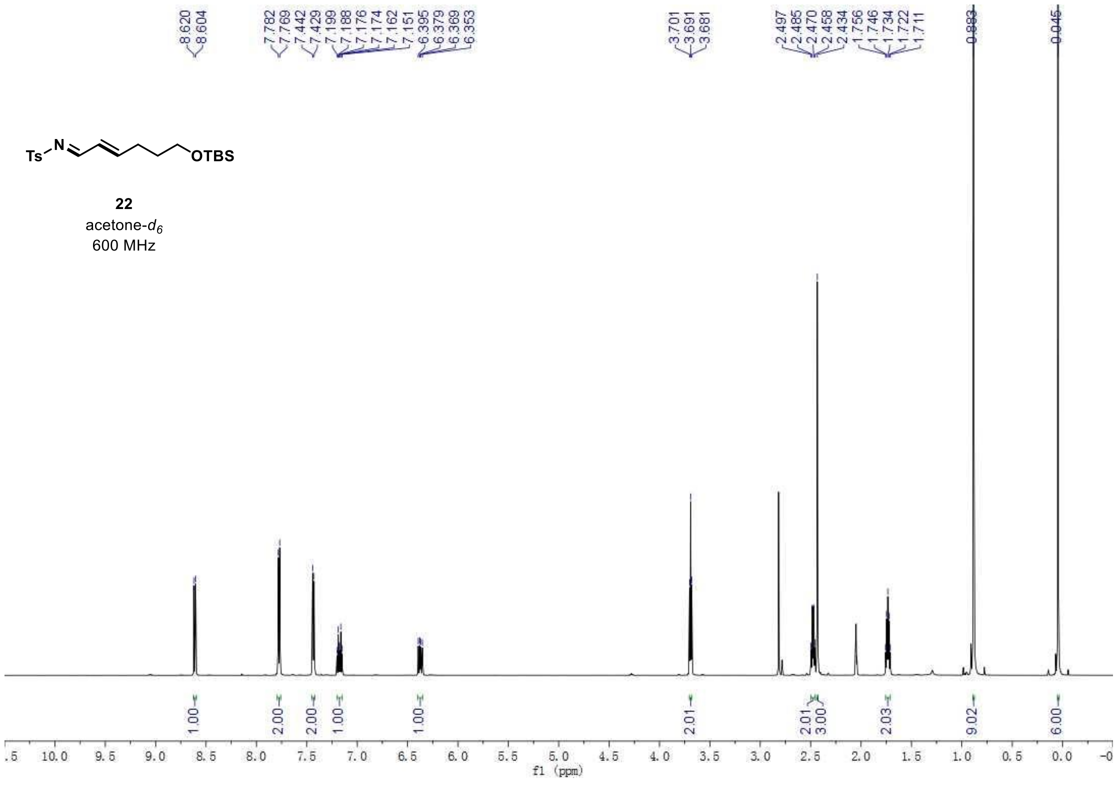

Supplementary Figure 89. ^{1}H NMR of compound 22. The sample has been recorded in 600 MHz, acetone-d_6 at 25 °C.

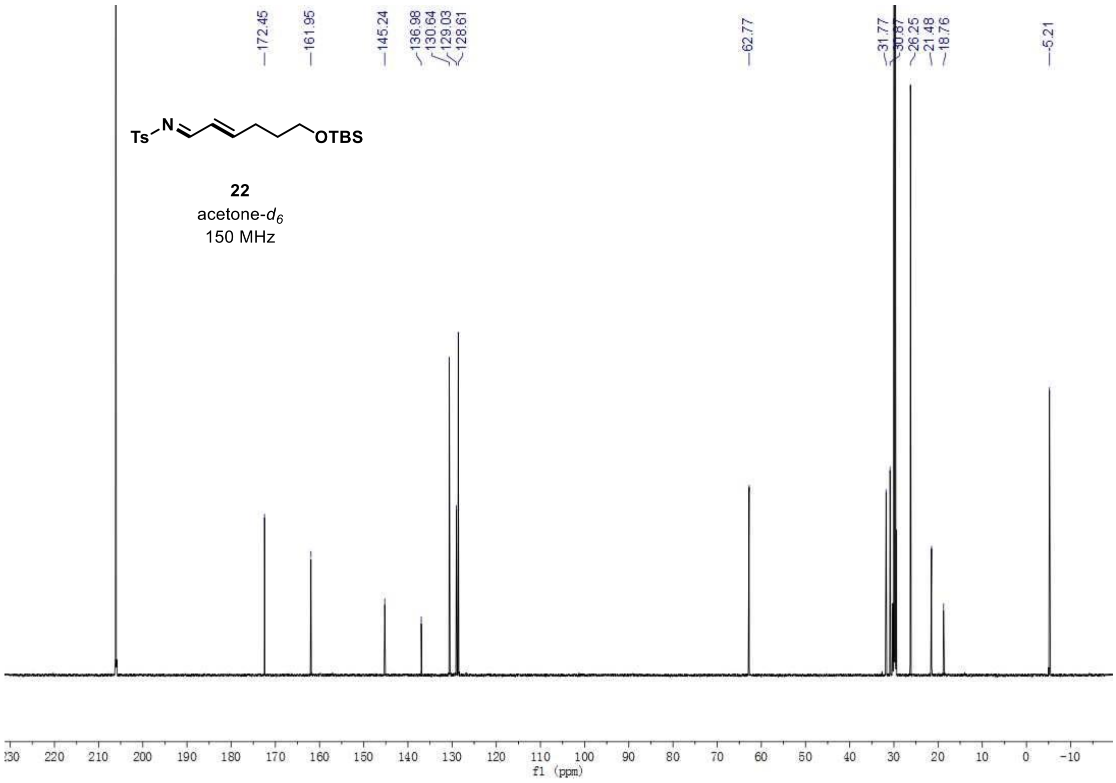

Supplementary Figure 90. ^{13}C NMR of compound 22. The sample has been recorded in 150 MHz, acetone-d_6 at 25 °C.

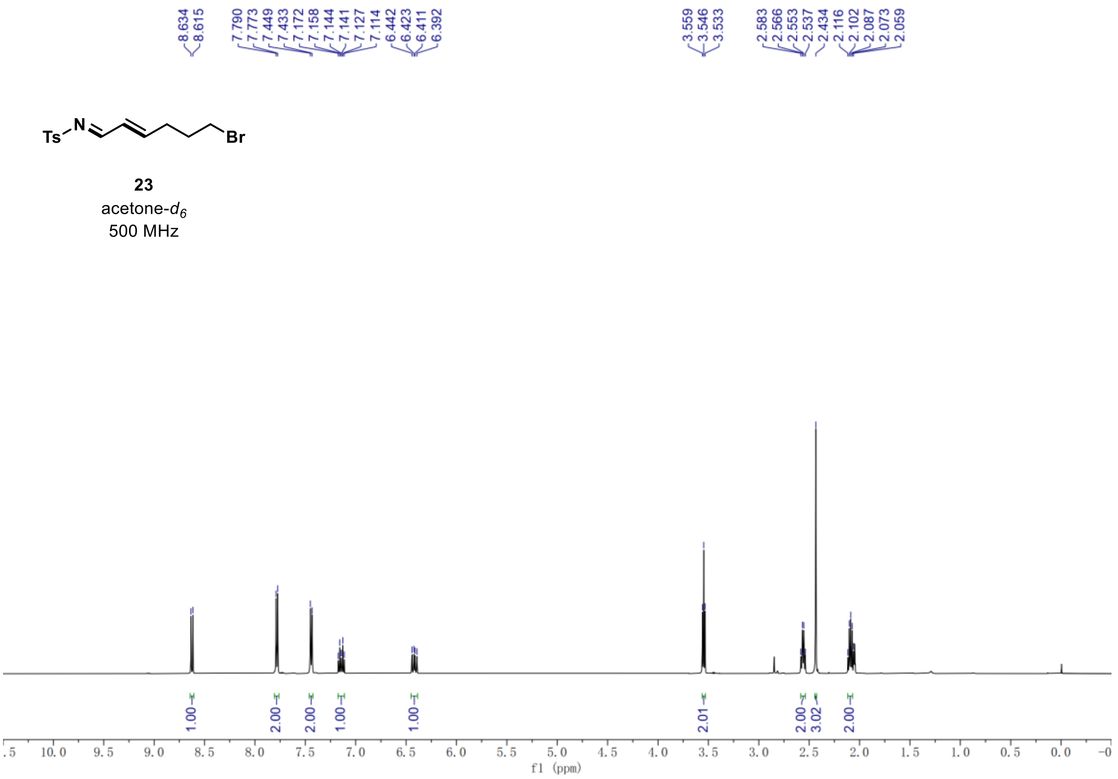

Supplementary Figure 91. ^{1}H NMR of compound 23. The sample has been recorded in 500 MHz, acetone-d_6 at 25 °C.

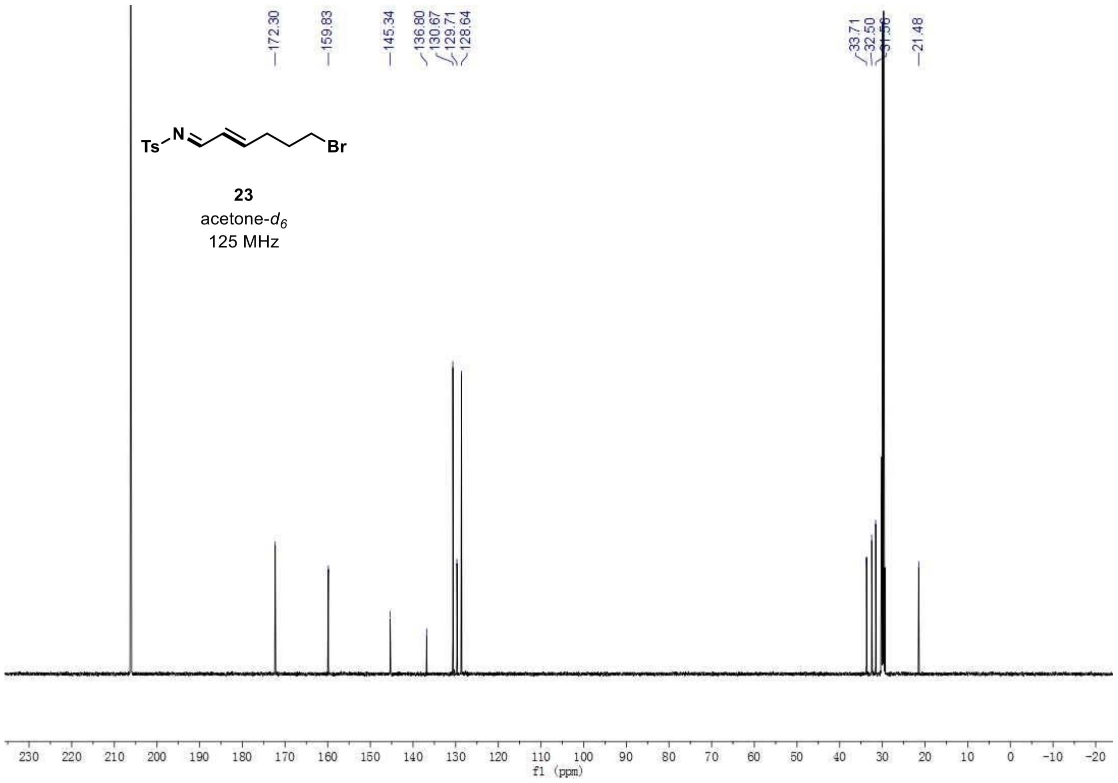

Supplementary Figure 92. ^{13}C NMR of compound 23. The sample has been recorded in 125 MHz, acetone-d_6 at 25 °C.

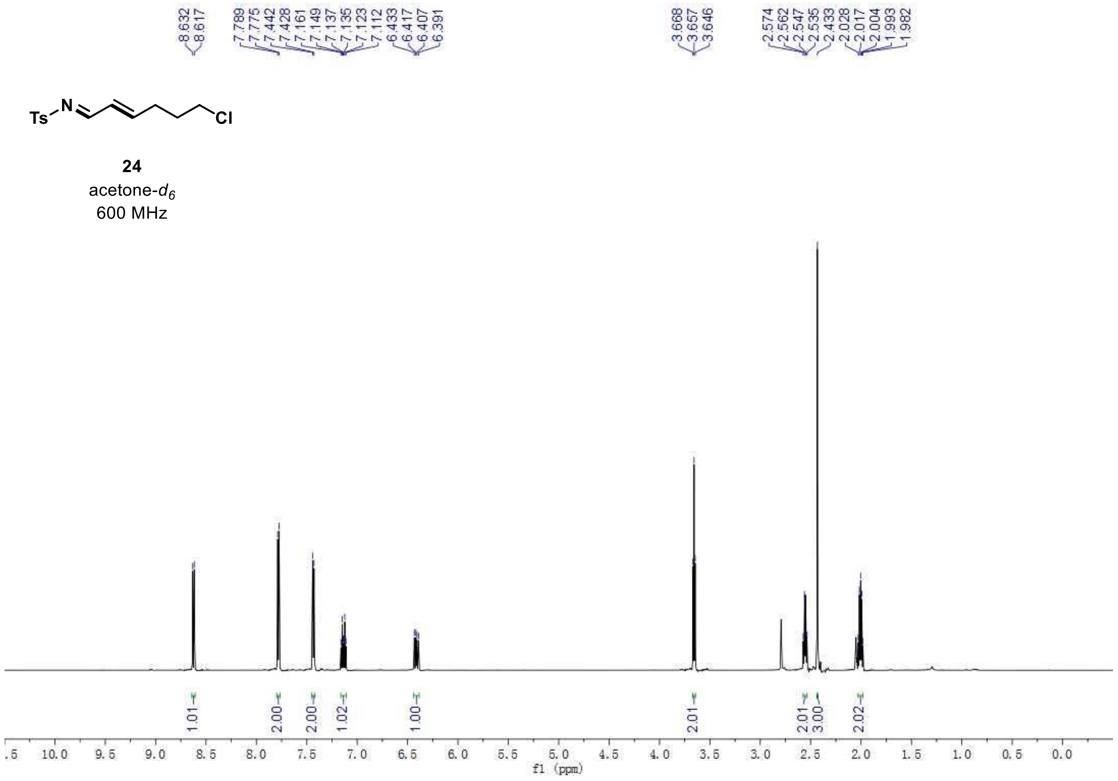

Supplementary Figure 93. ^{1}H NMR of compound 24. The sample has been recorded in 600 MHz, acetone-d_6 at 25 °C.

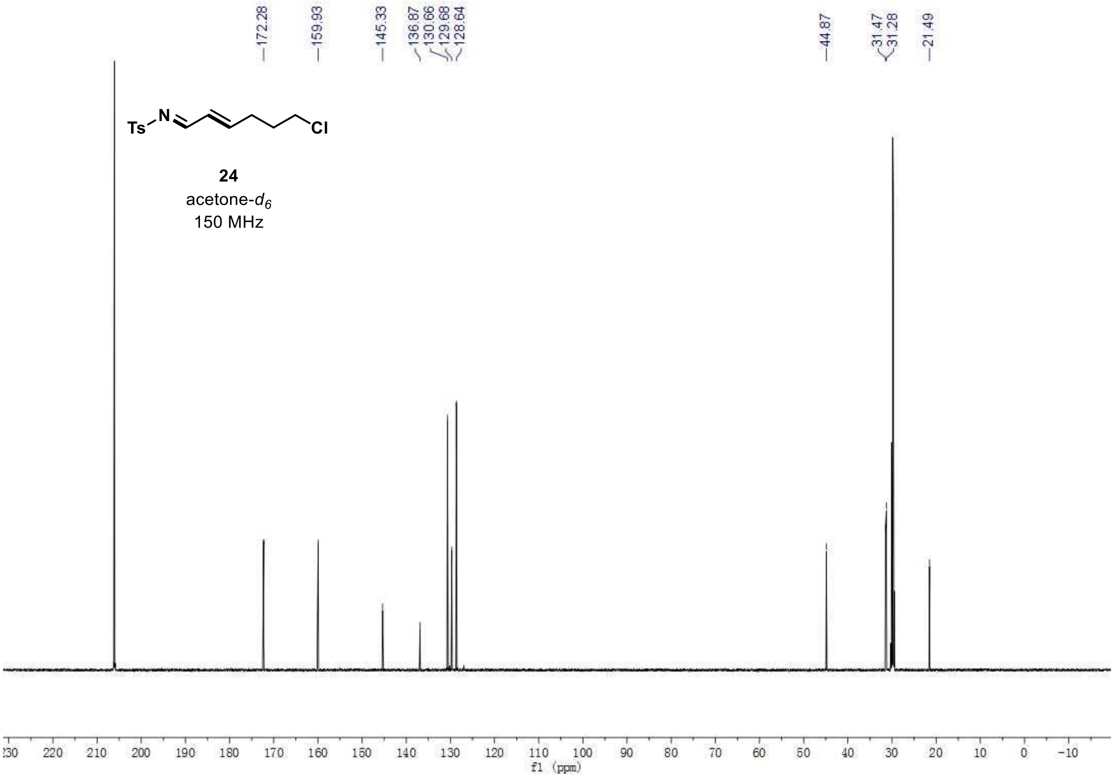

Supplementary Figure 94. ^{13}C NMR of compound 24. The sample has been recorded in 150 MHz, acetone-d_6 at 25 °C.

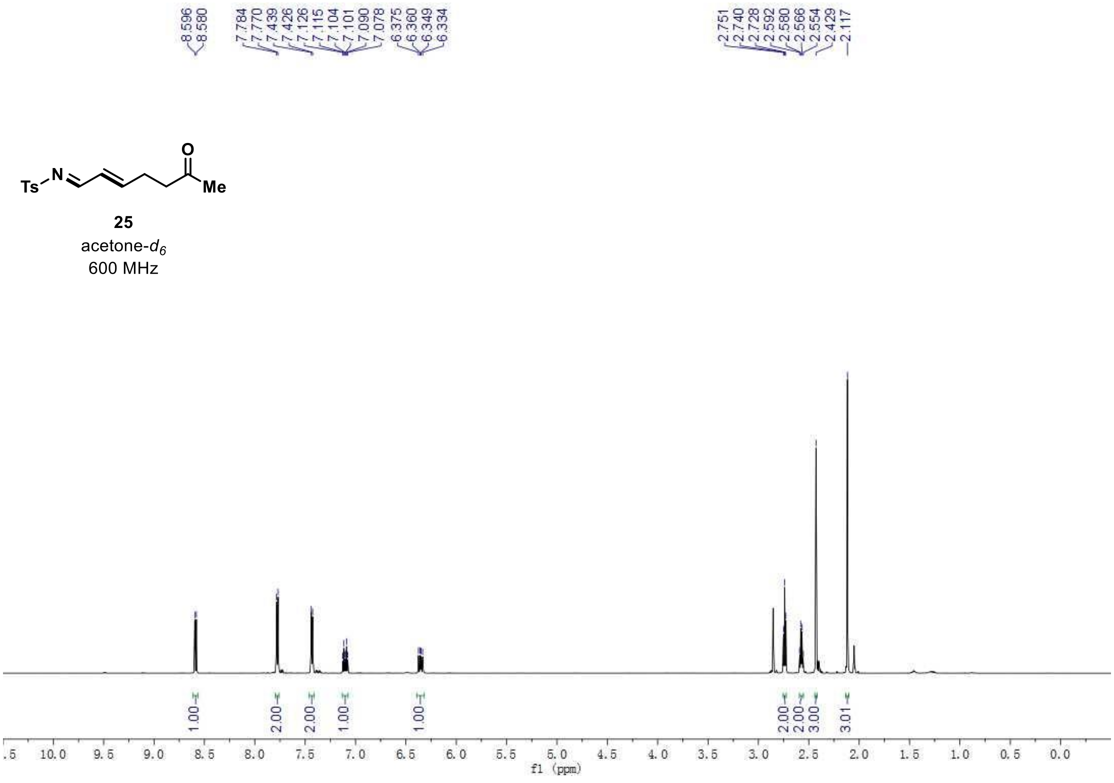

Supplementary Figure 95. ^{1}H NMR of compound 25. The sample has been recorded in 600 MHz, acetone-d_6 at 25 °C.

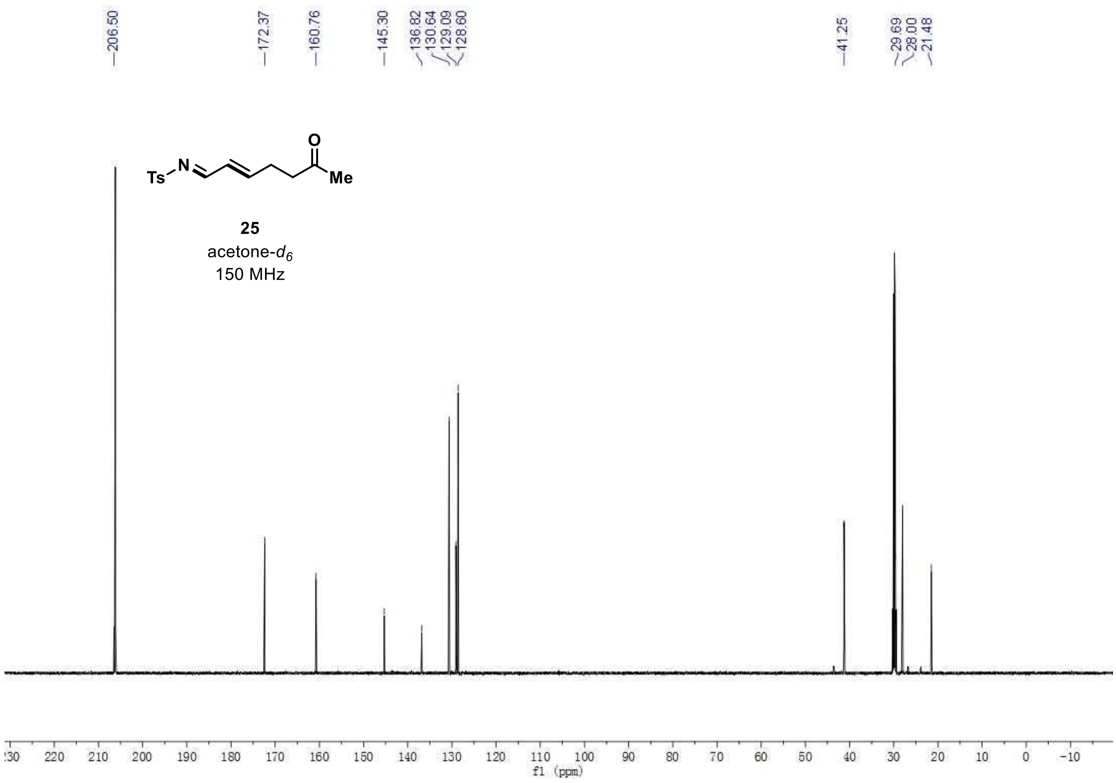

Supplementary Figure 96. ^{13}C NMR of compound 25. The sample has been recorded in 150 MHz, acetone-d_6 at 25 °C.

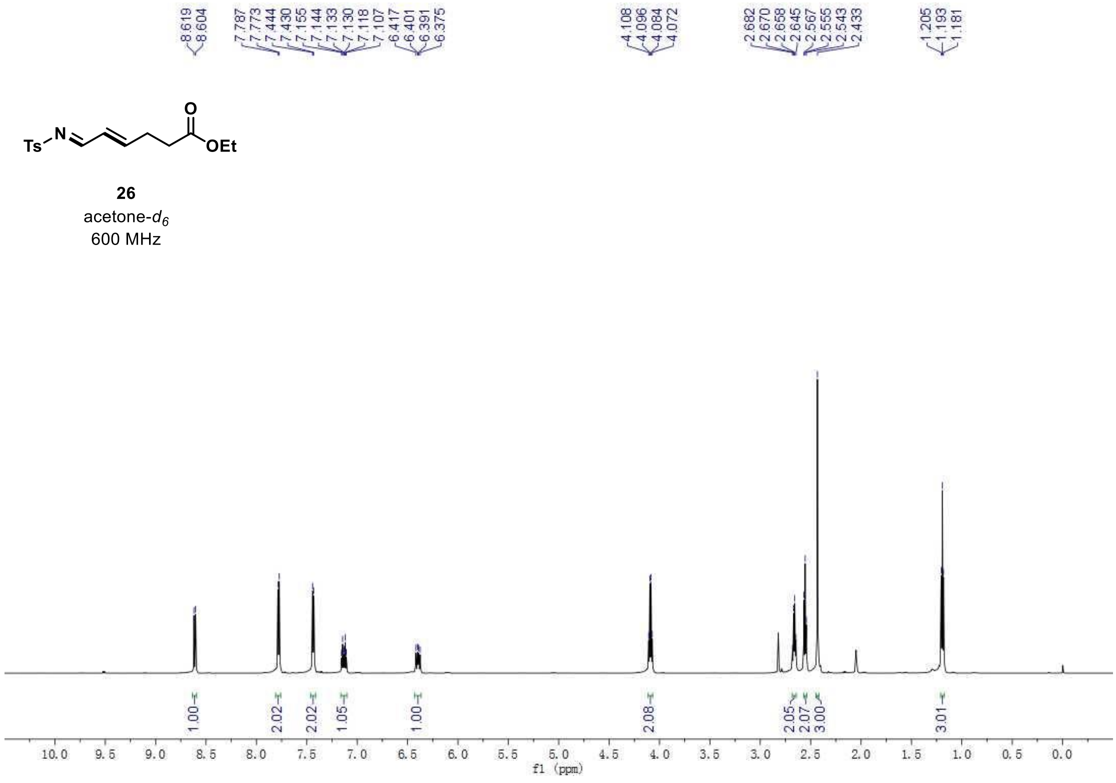

Supplementary Figure 97. ^{1}H NMR of compound 26. The sample has been recorded in 600 MHz, acetone-d_6 at 25 °C.

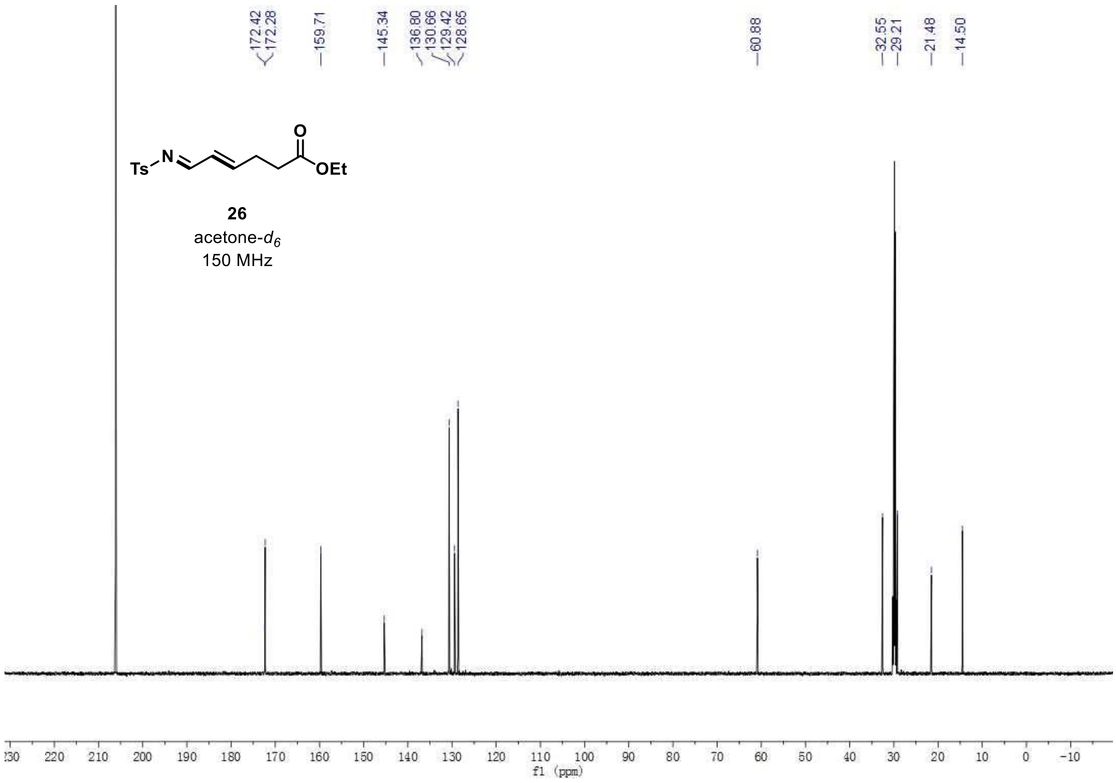

Supplementary Figure 98. ^{13}C NMR of compound 26. The sample has been recorded in 150 MHz, acetone-d_6 at 25 °C.

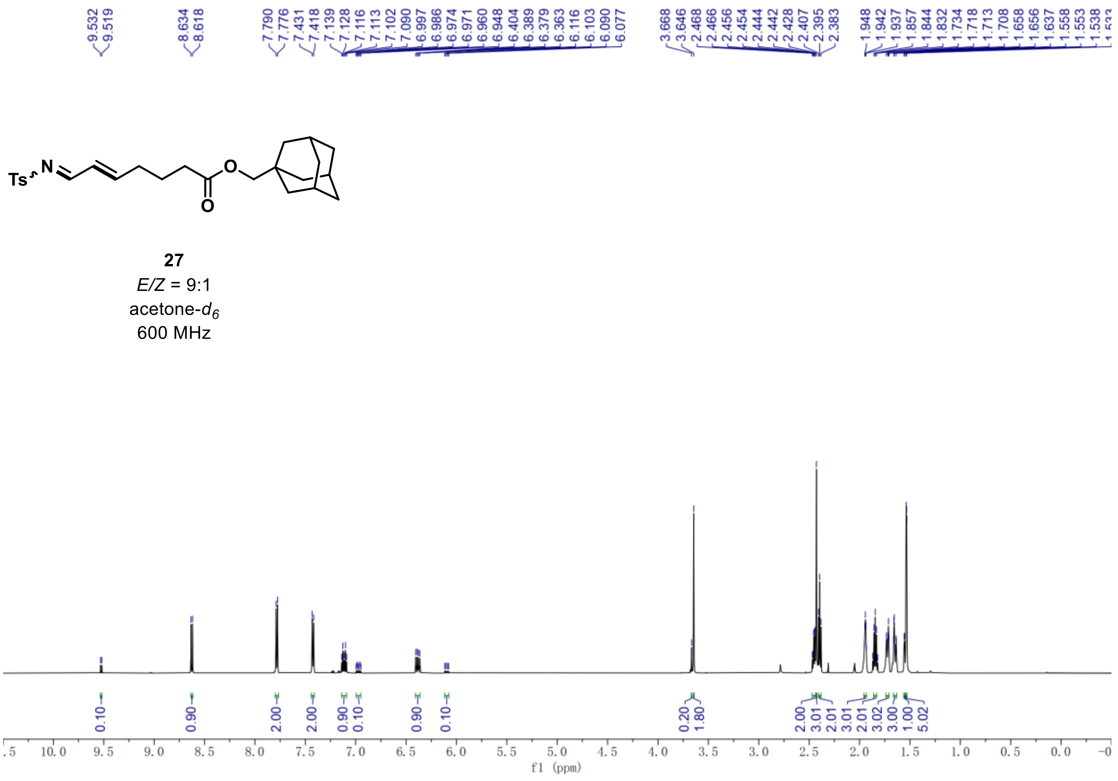

Supplementary Figure 99. ^{1}H NMR of compound 27. The sample has been recorded in 600 MHz, acetone-d_6 at 25 °C.

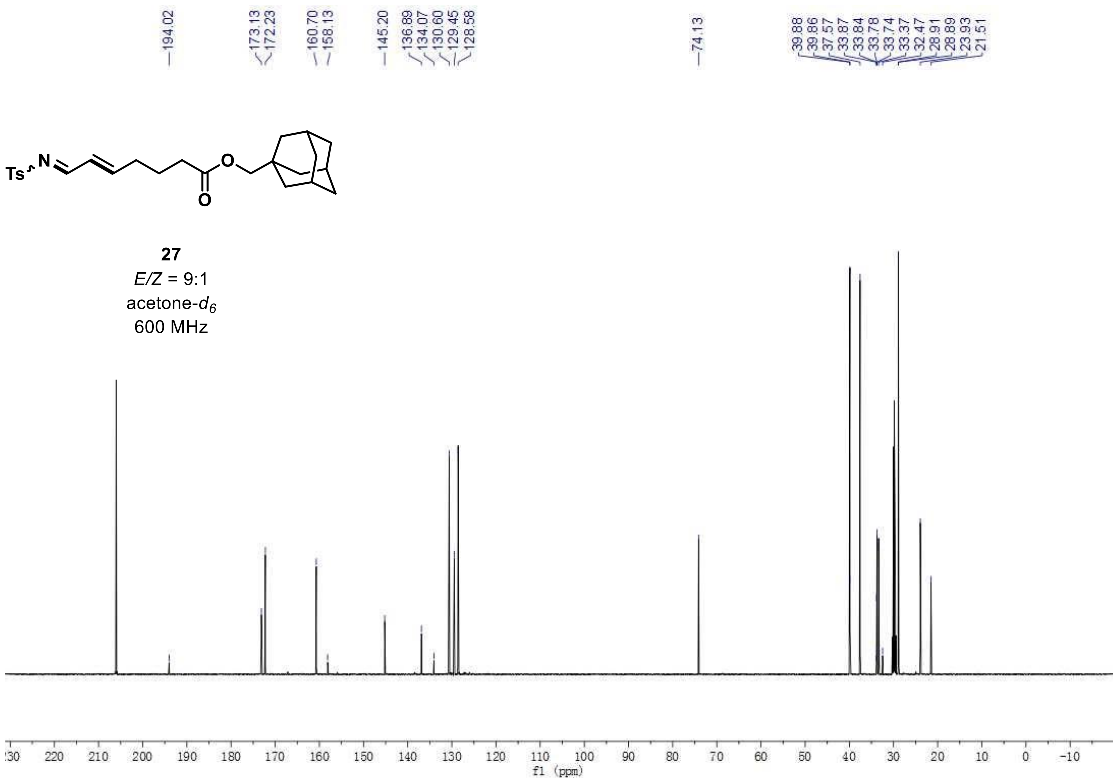

Supplementary Figure 100. ^{13}**C NMR of compound 27.** The sample has been recorded in 150 MHz, acetone-d_6 at 25 °C.

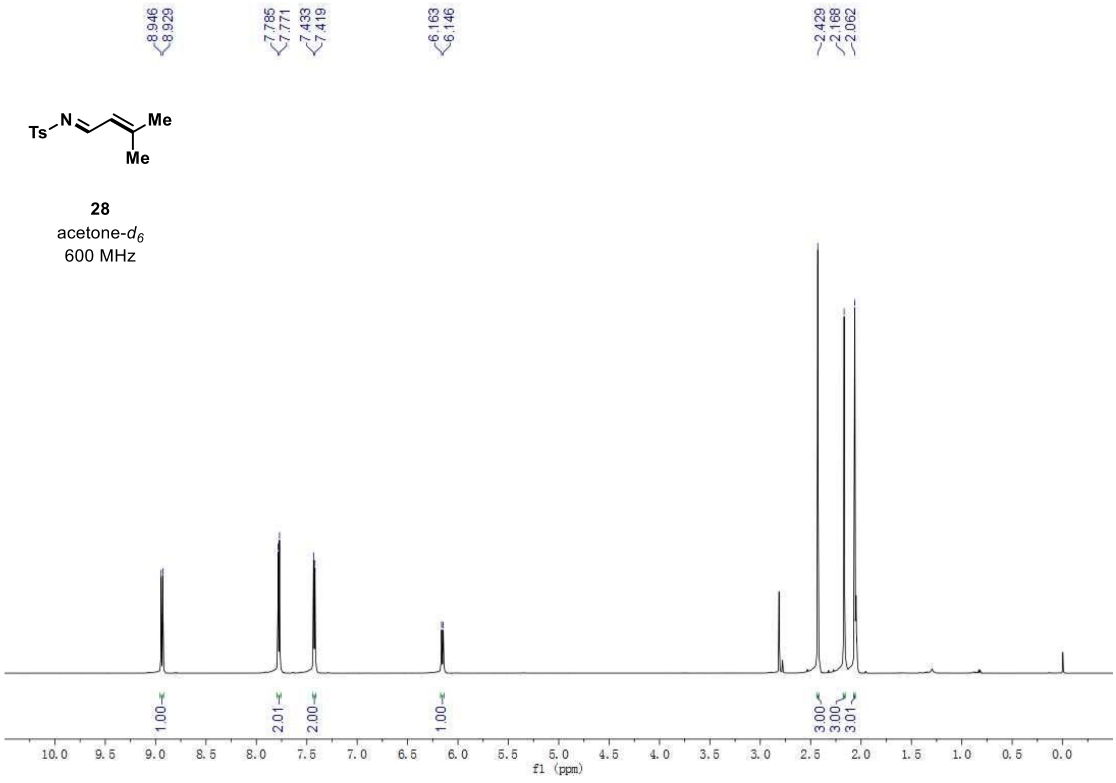

Supplementary Figure 101. ^{1}**H NMR of compound 28.** The sample has been recorded in 600 MHz, acetone-d_6 at 25 °C.

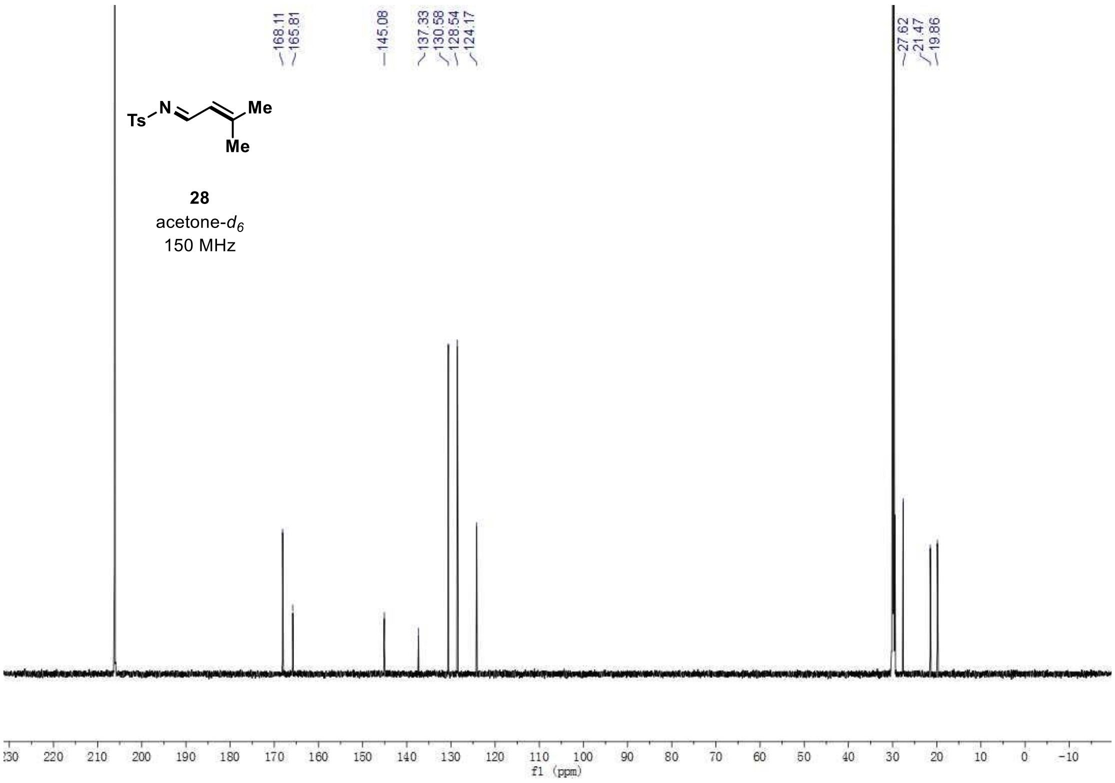

Supplementary Figure 102. ^{13}C NMR of compound 28. The sample has been recorded in 150 MHz, acetone-d_6 at 25 °C.

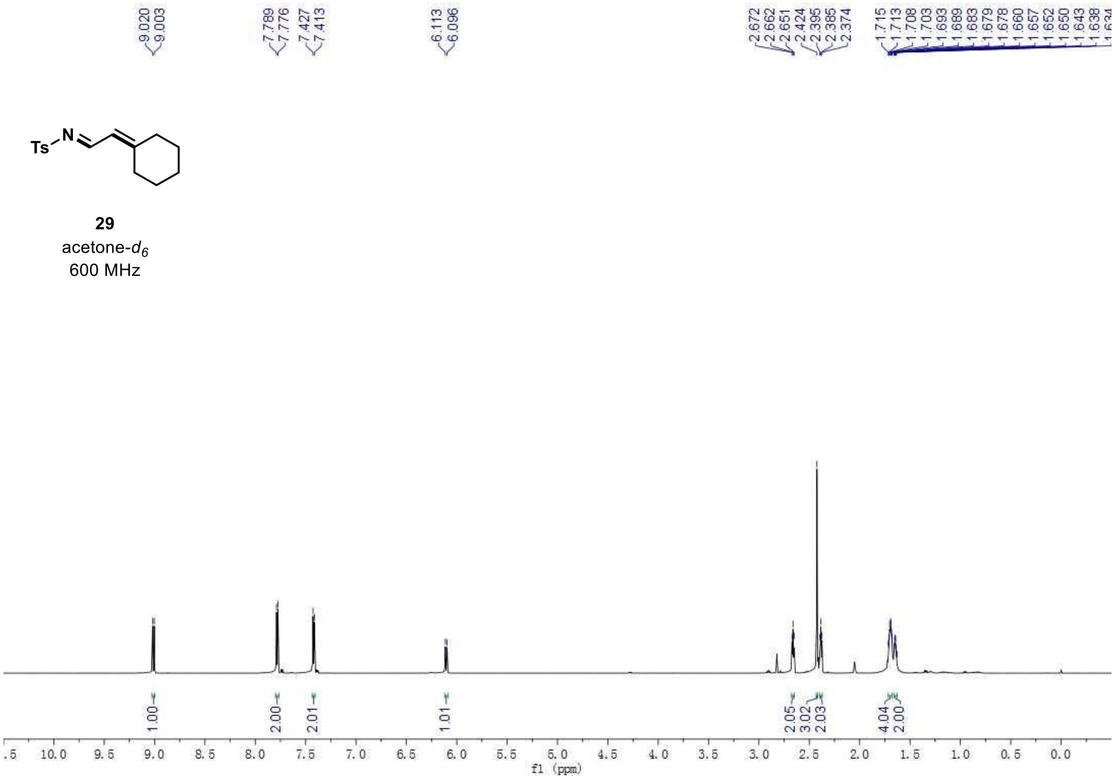

Supplementary Figure 103. ^{1}H NMR of compound 29. The sample has been recorded in 600 MHz, acetone-d_6 at 25 °C.

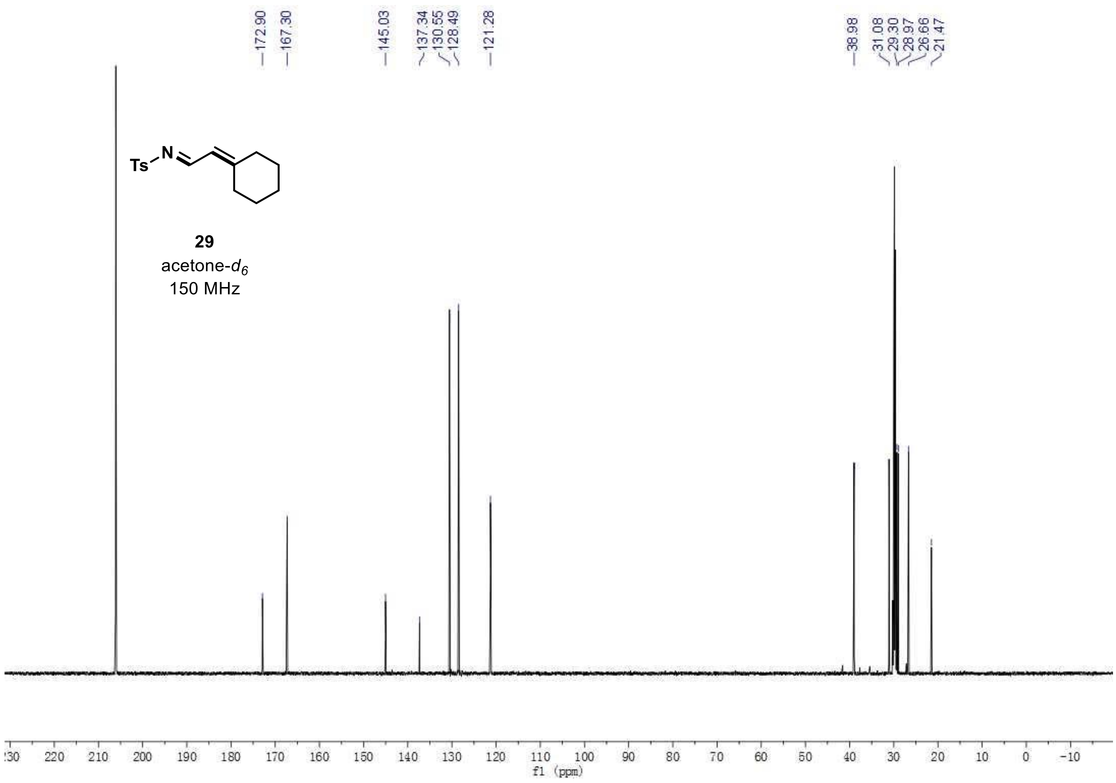

Supplementary Figure 104. ^{13}C NMR of compound 29. The sample has been recorded in 150 MHz, acetone-d_6 at 25 °C.

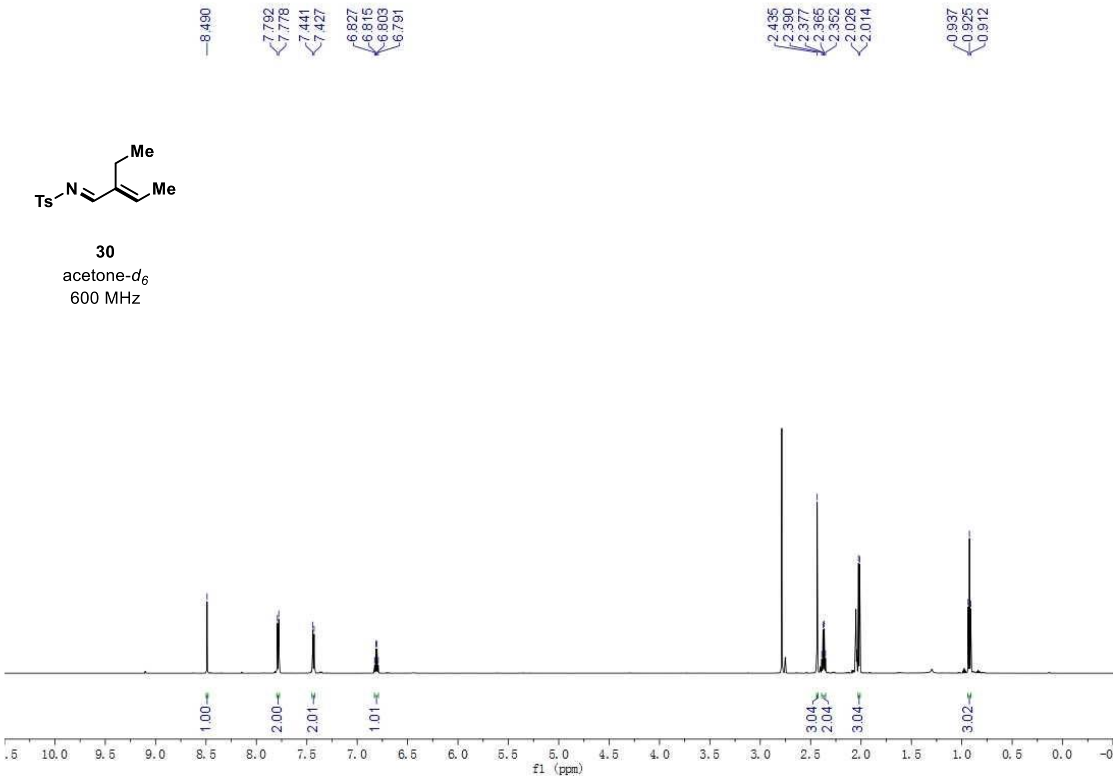

Supplementary Figure 105. ^{1}H NMR of compound 30. The sample has been recorded in 600 MHz, acetone-d_6 at 25 °C.

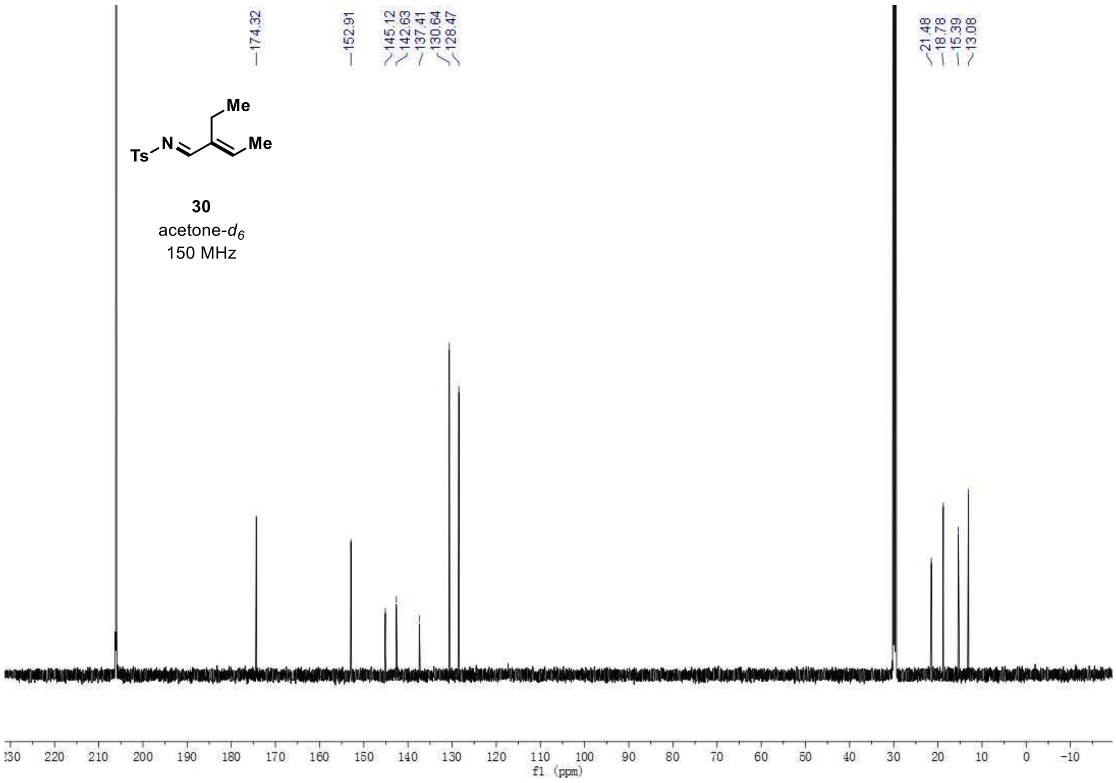

Supplementary Figure 106. ^{13}C NMR of compound 30. The sample has been recorded in 150 MHz, acetone-d_6 at 25 °C.

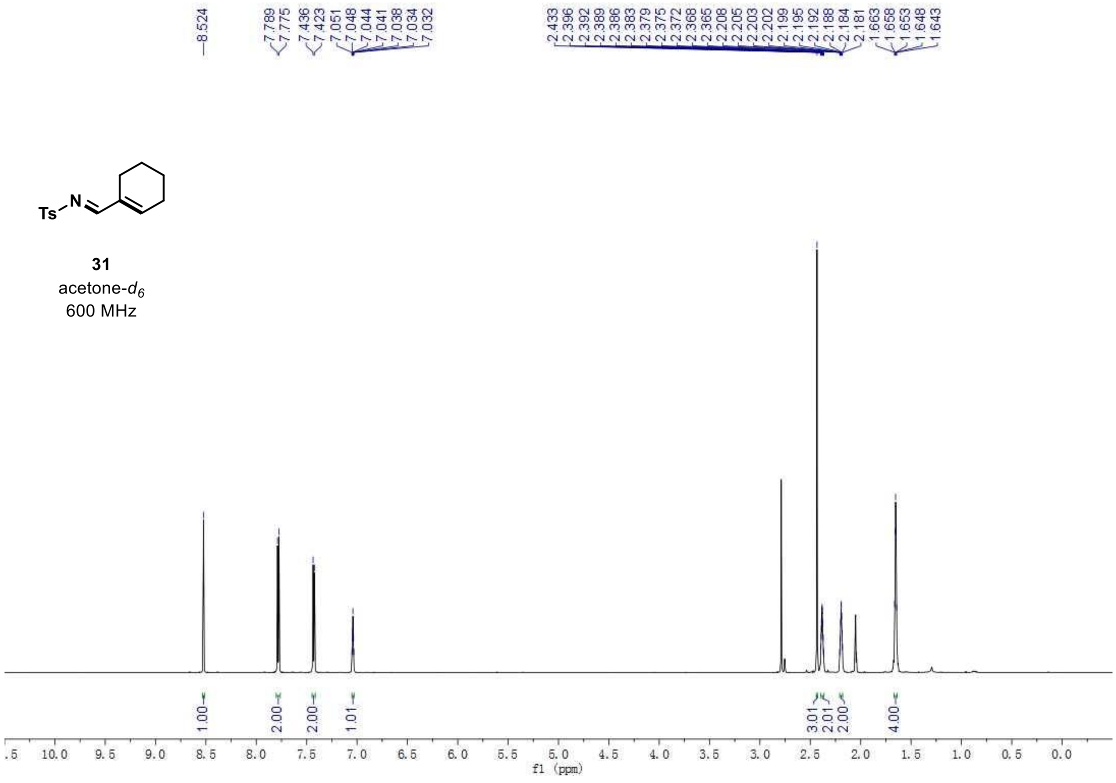

Supplementary Figure 107. ^{1}H NMR of compound 31. The sample has been recorded in 600 MHz, acetone-d_6 at 25 °C.

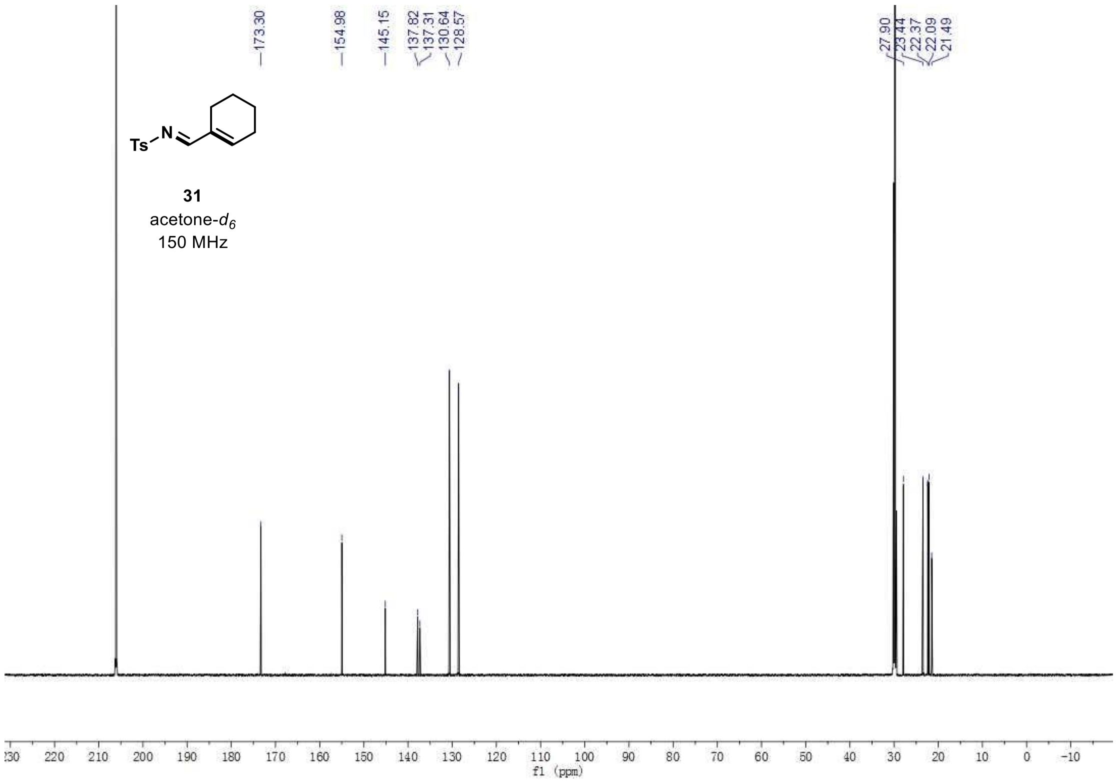

Supplementary Figure 108. ^{13}C NMR of compound 31. The sample has been recorded in 150 MHz, acetone-d_6 at 25 °C.

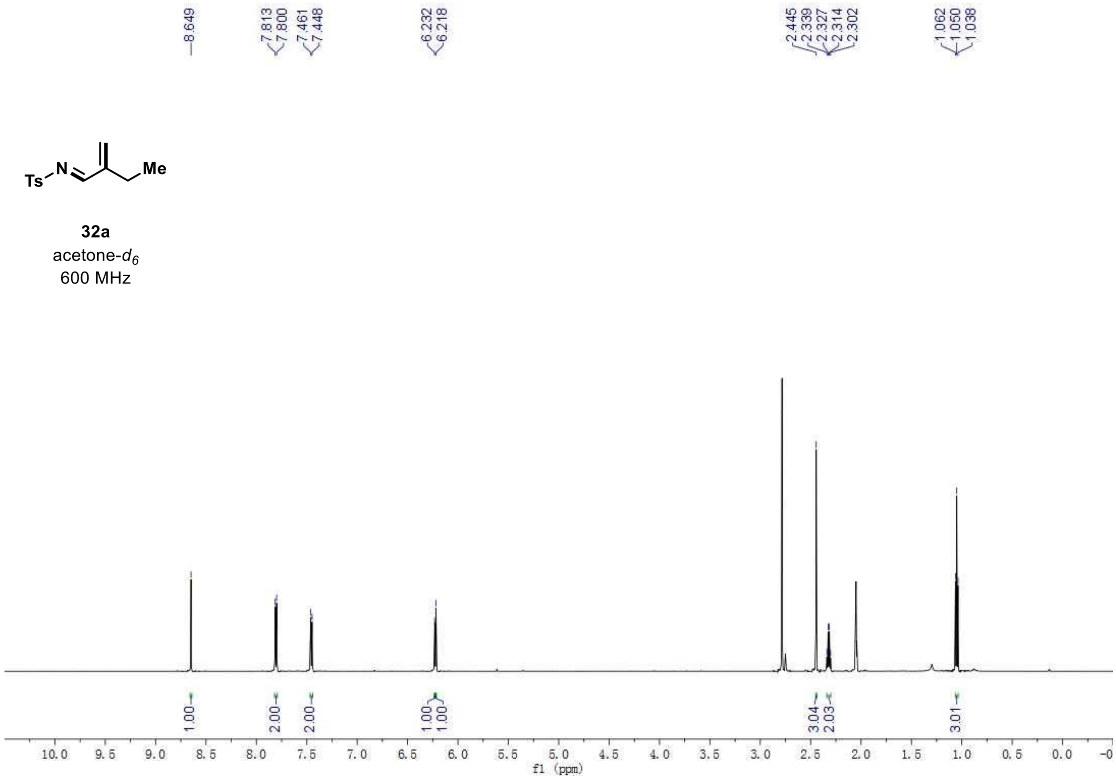

Supplementary Figure 109. ^{1}H NMR of compound 32a. The sample has been recorded in 600 MHz, acetone-d_6 at 25 °C.

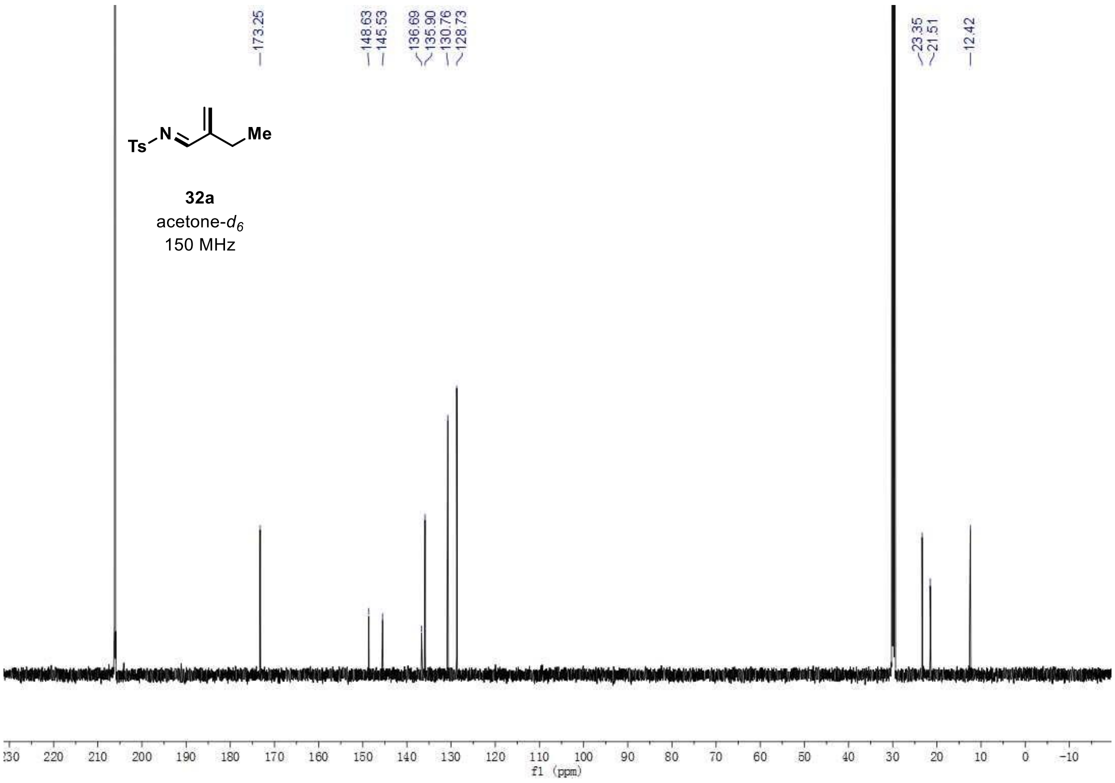

Supplementary Figure 110. ^{13}C NMR of compound 32a. The sample has been recorded in 150 MHz, acetone-d_6 at 25 °C.

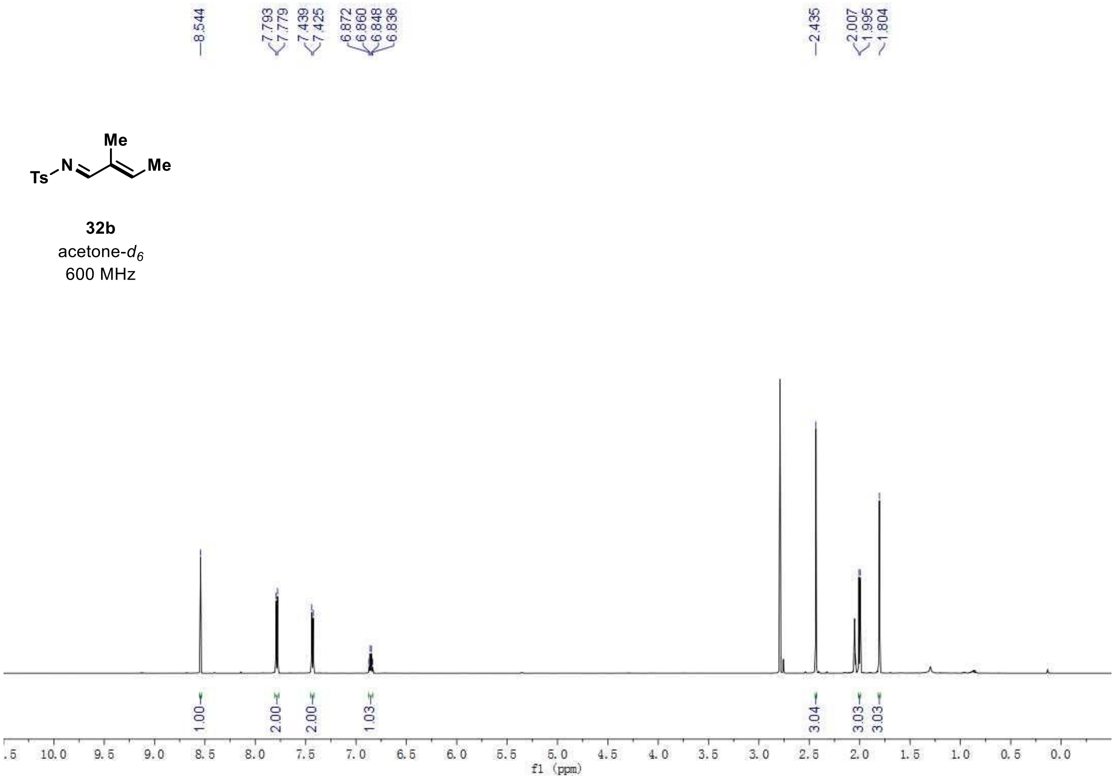

Supplementary Figure 111. ^{1}H NMR of compound 32b. The sample has been recorded in 600 MHz, acetone-d_6 at 25 °C.

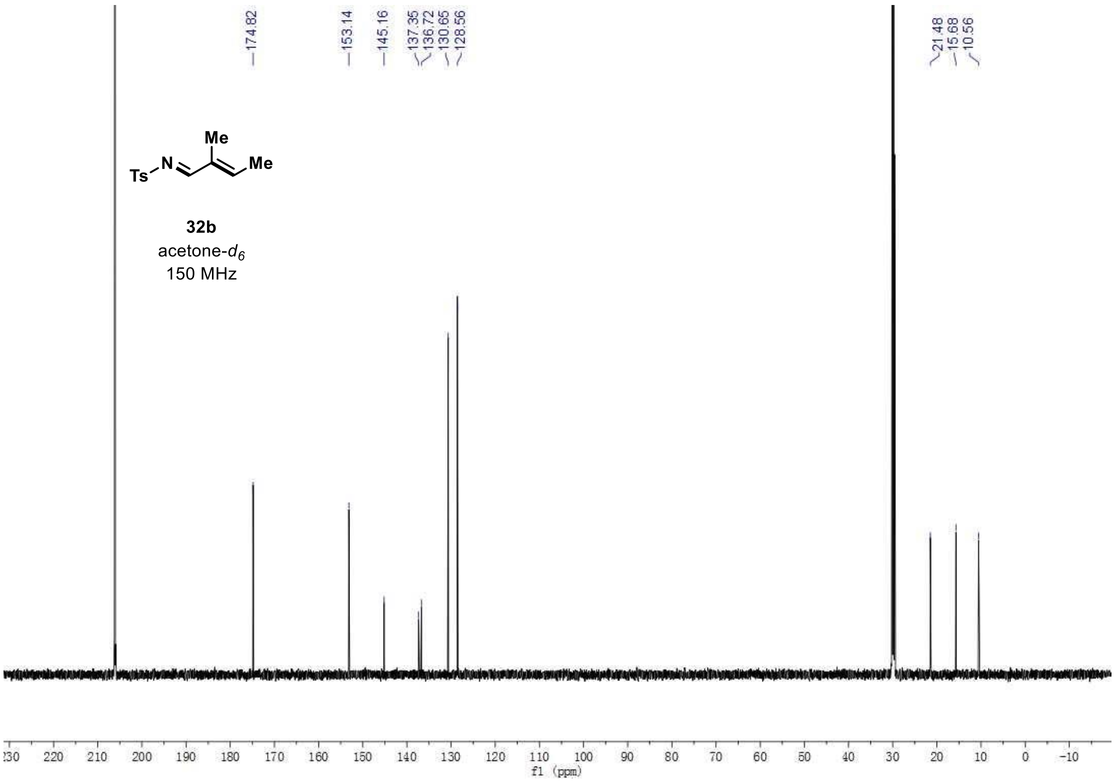

Supplementary Figure 112. ^{13}C NMR of compound 32b. The sample has been recorded in 150 MHz, acetone-d_6 at 25 °C.

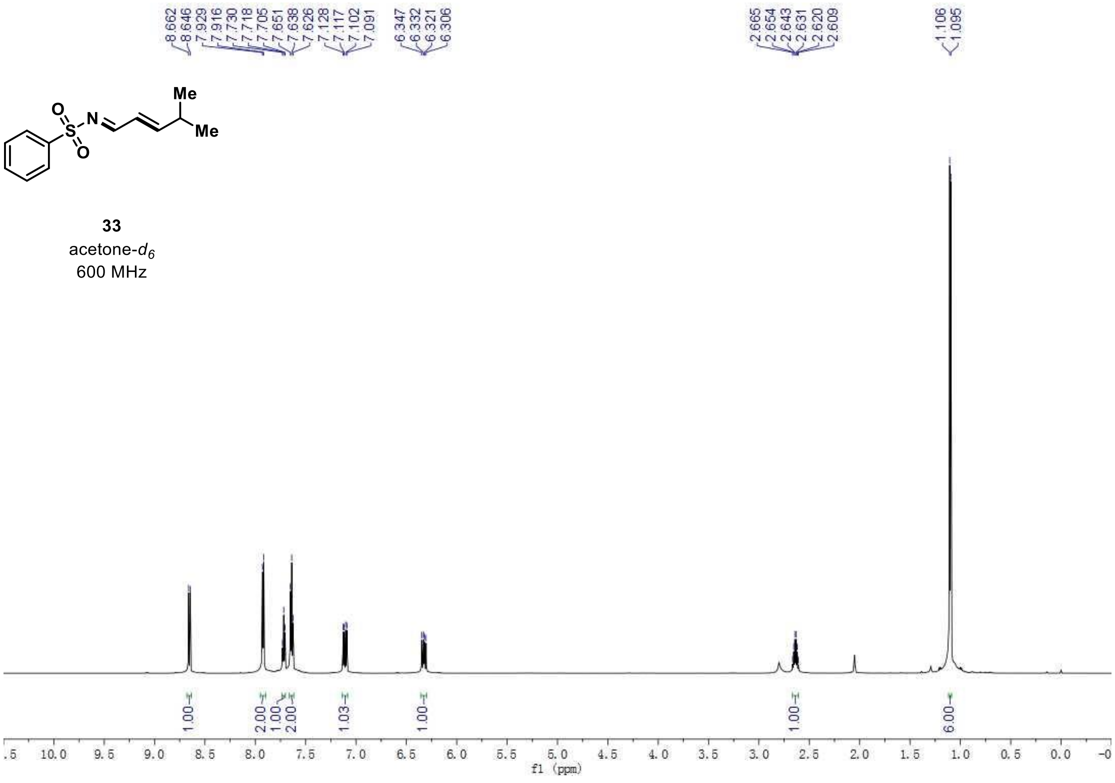

Supplementary Figure 113. ^{1}H NMR of compound 33. The sample has been recorded in 600 MHz, acetone-d_6 at 25 °C.

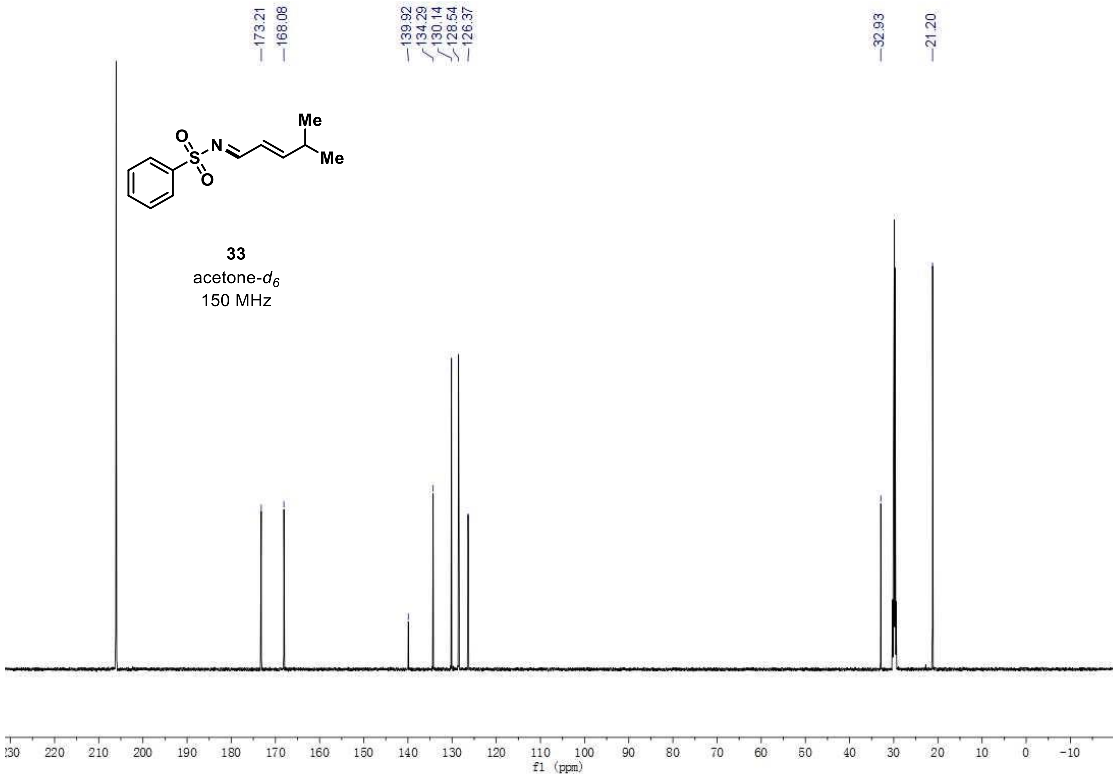

Supplementary Figure 114. ^{13}C NMR of compound 33. The sample has been recorded in 150 MHz, acetone-d_6 at 25 °C.

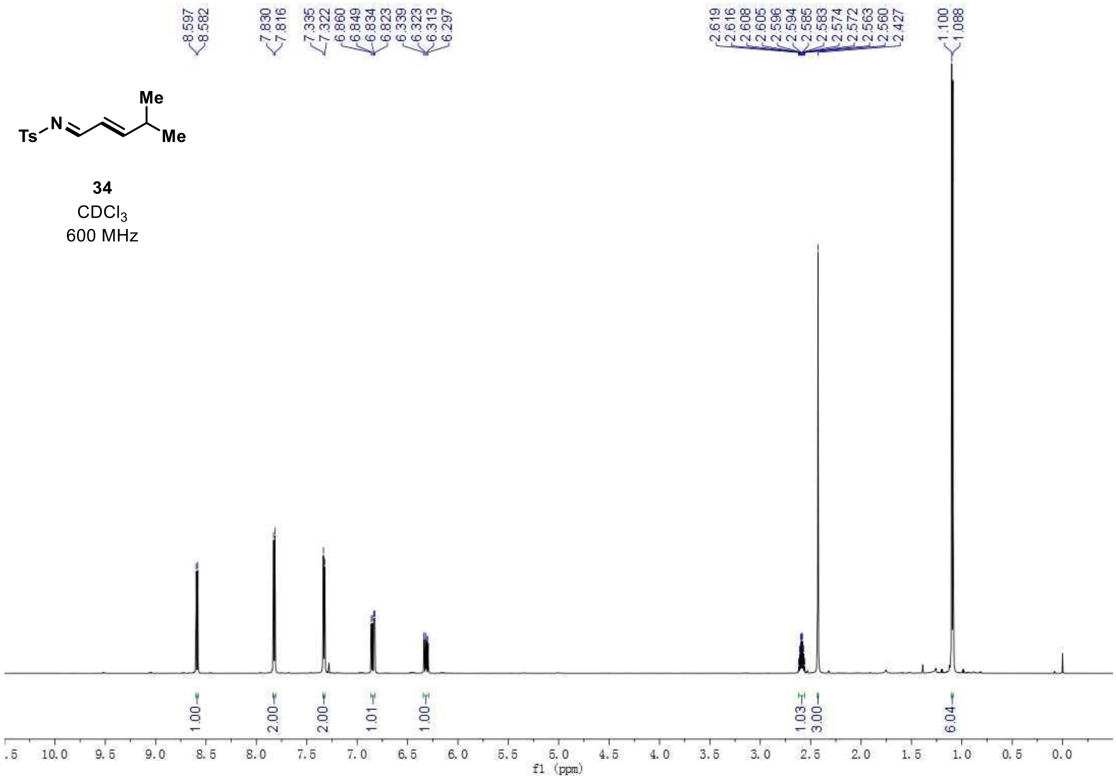

Supplementary Figure 115. ^{1}H NMR of compound 34. The sample has been recorded in 600 MHz, $CDCl_3$ at 25 °C.

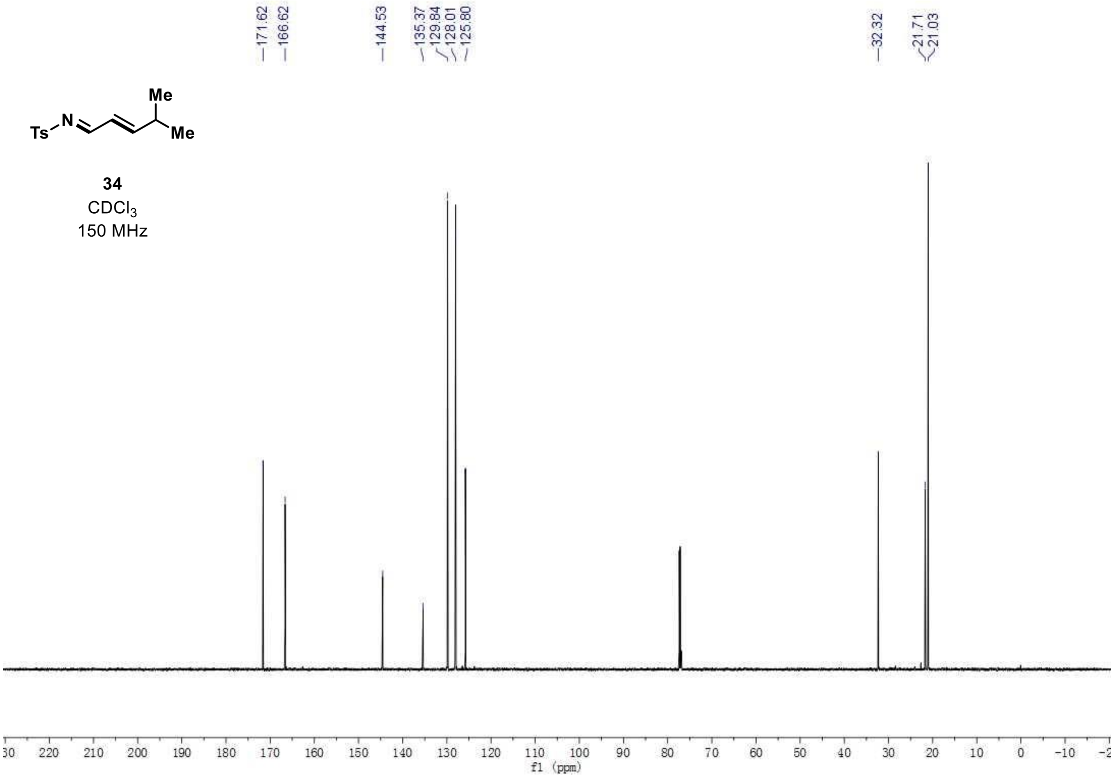

Supplementary Figure 116. ^{13}C NMR of compound 34. The sample has been recorded in 150 MHz, $CDCl_3$ at 25 °C.

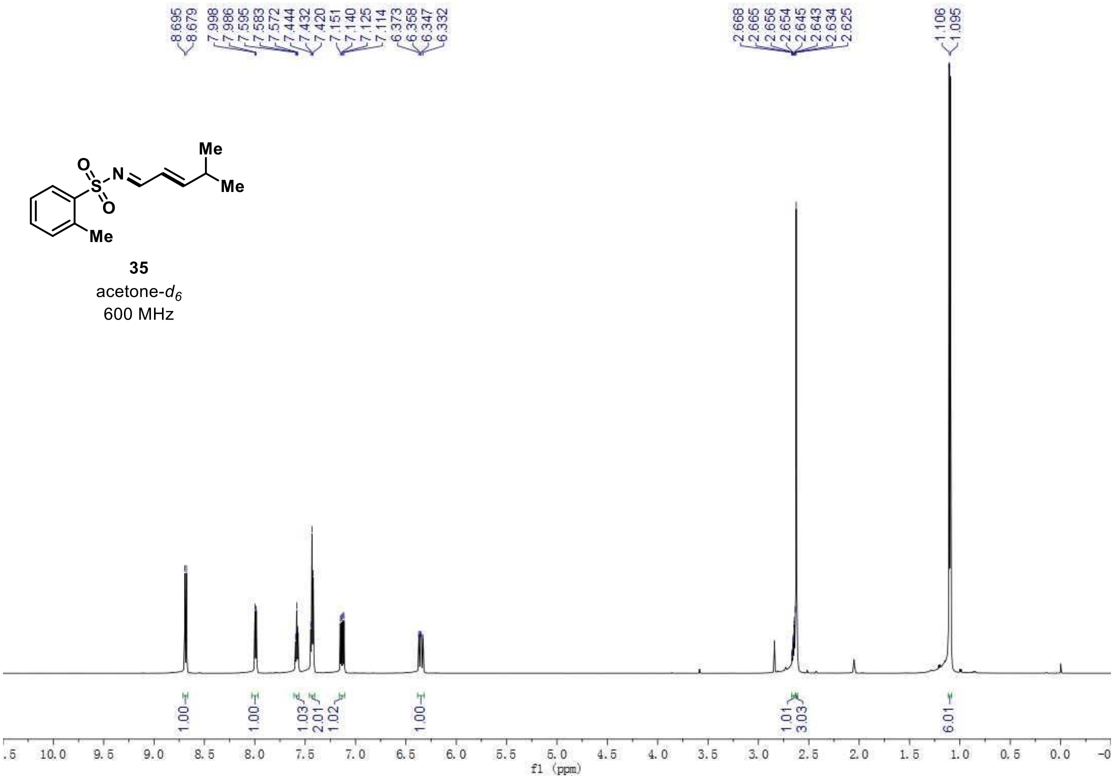

Supplementary Figure 117. ^{1}H NMR of compound 35. The sample has been recorded in 600 MHz, acetone-d_6 at 25 °C.

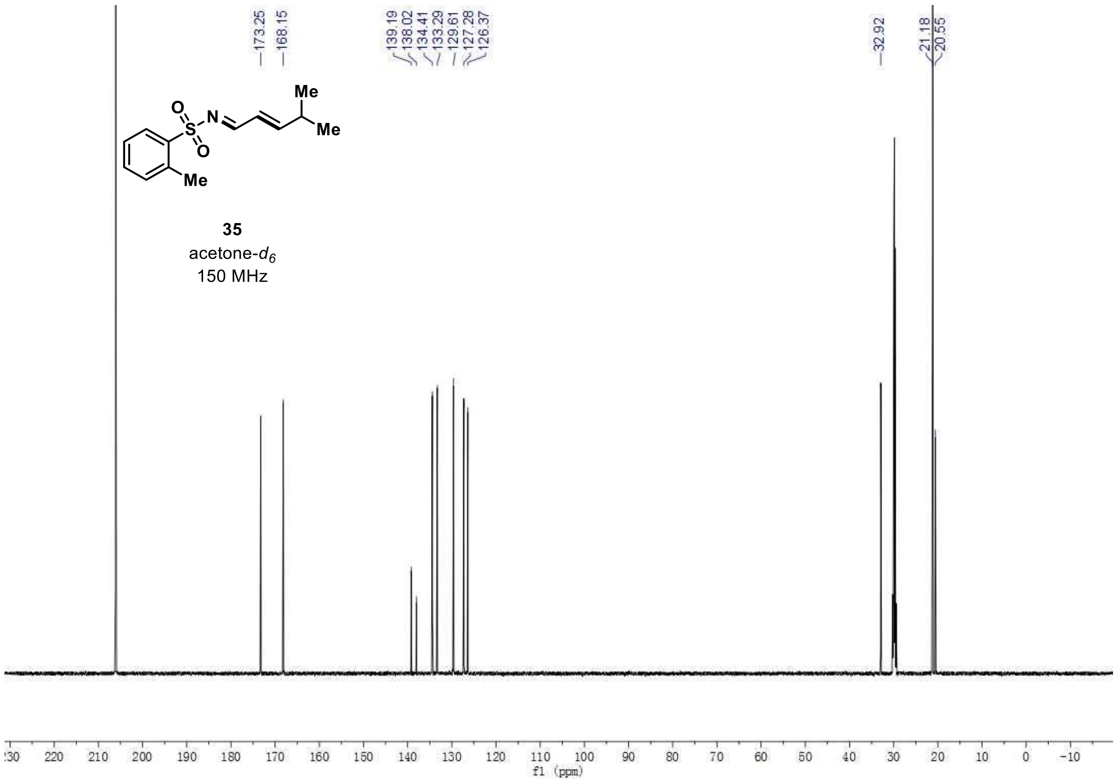

Supplementary Figure 118. ^{13}C NMR of compound 35. The sample has been recorded in 150 MHz, acetone-d_6 at 25 °C.

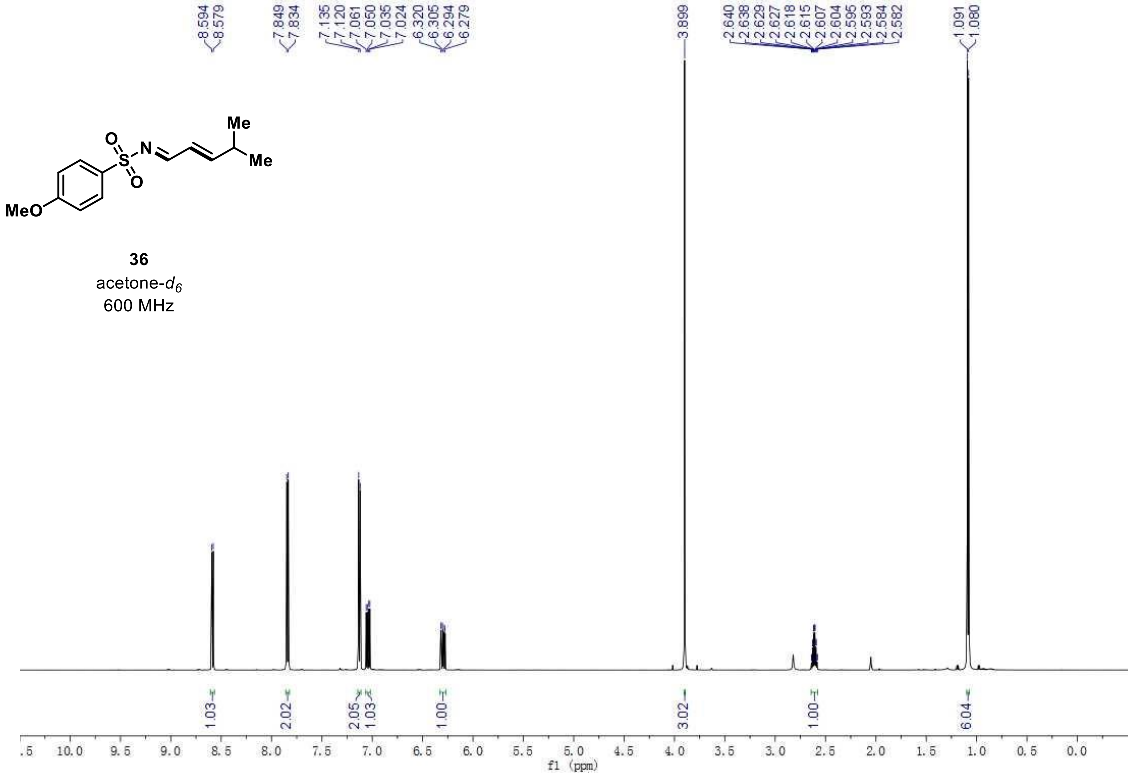

Supplementary Figure 119. ^{1}H NMR of compound 36. The sample has been recorded in 600 MHz, acetone-d_6 at 25 °C.

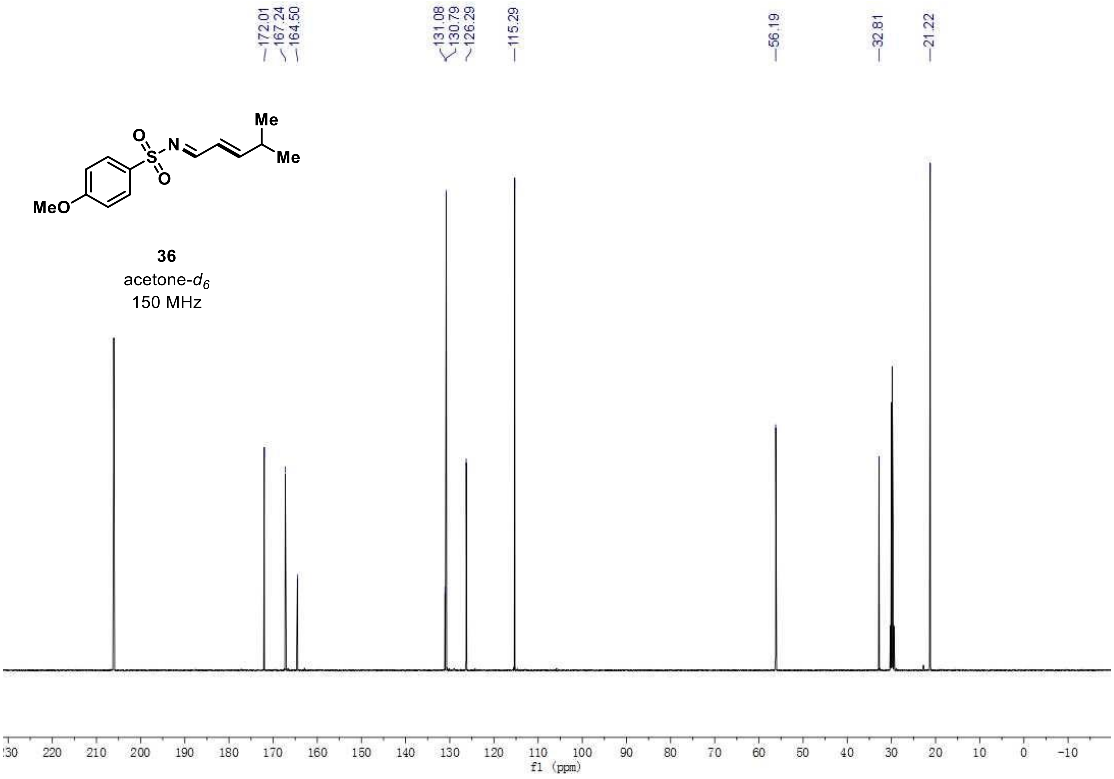

Supplementary Figure 120. ^{13}C NMR of compound 36. The sample has been recorded in 150 MHz, acetone-d_6 at 25 °C.

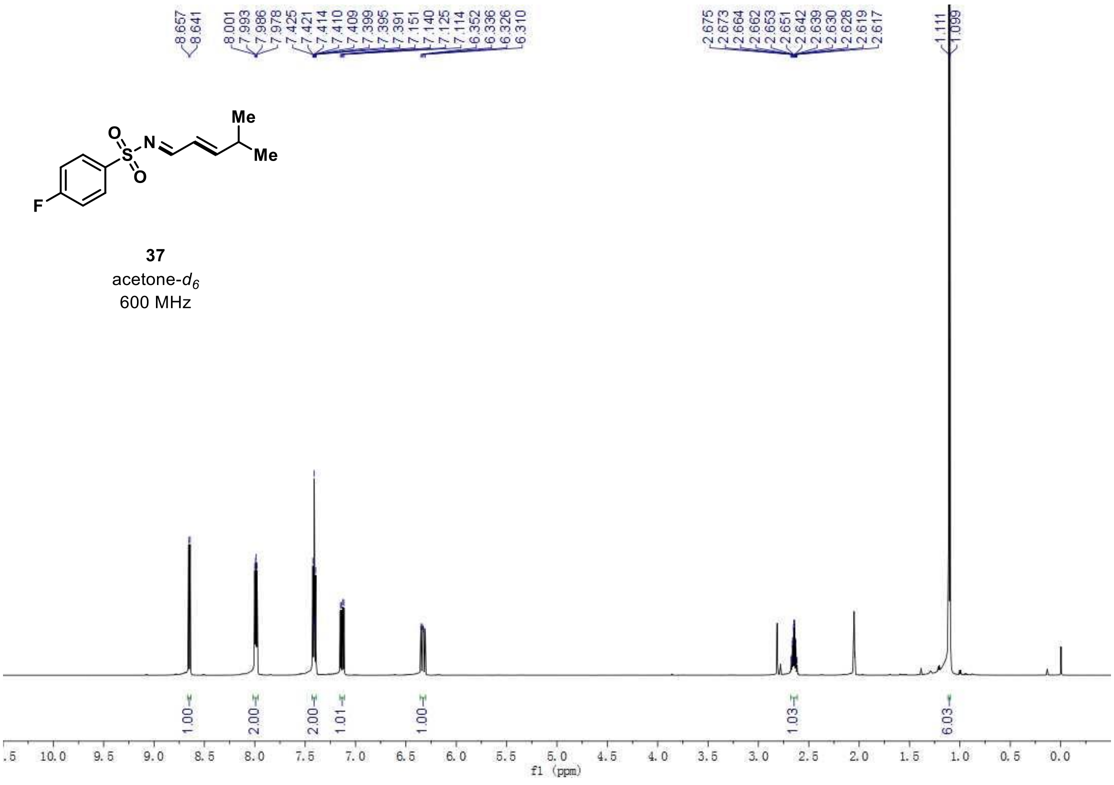

Supplementary Figure 121. ^{1}H NMR of compound 37. The sample has been recorded in 600 MHz, acetone-d_6 at 25 °C.

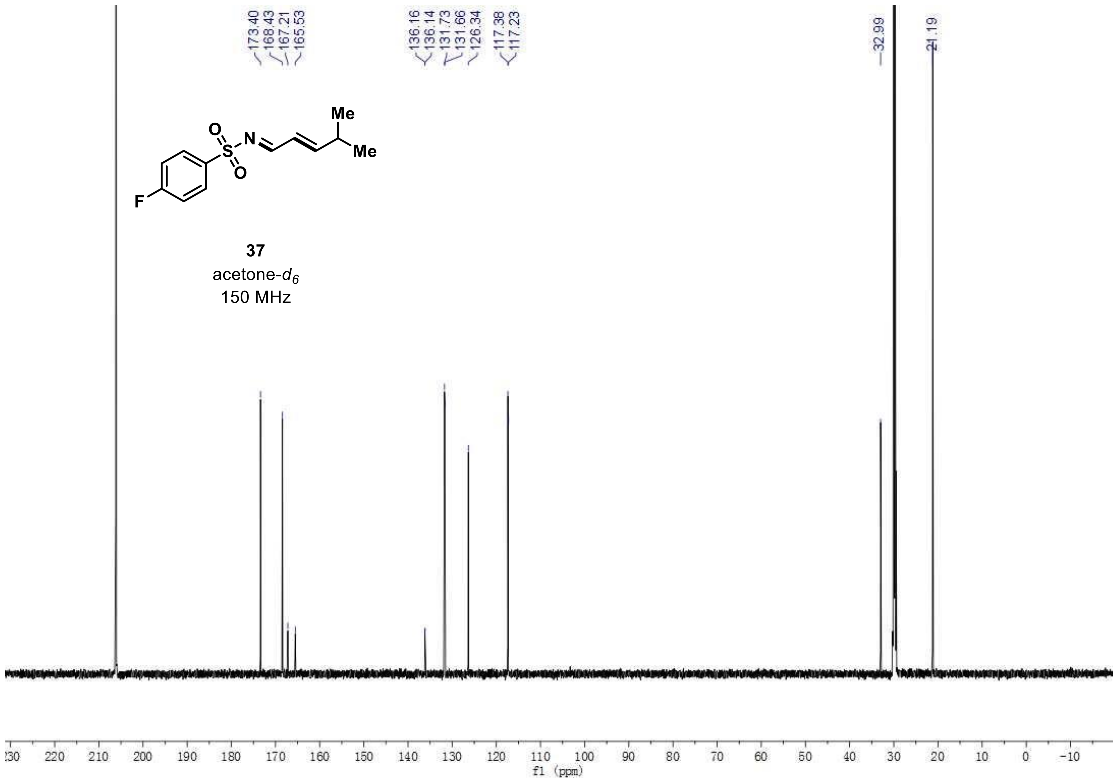

Supplementary Figure 122. ^{13}C NMR of compound 37. The sample has been recorded in 150 MHz, acetone-d_6 at 25 °C.

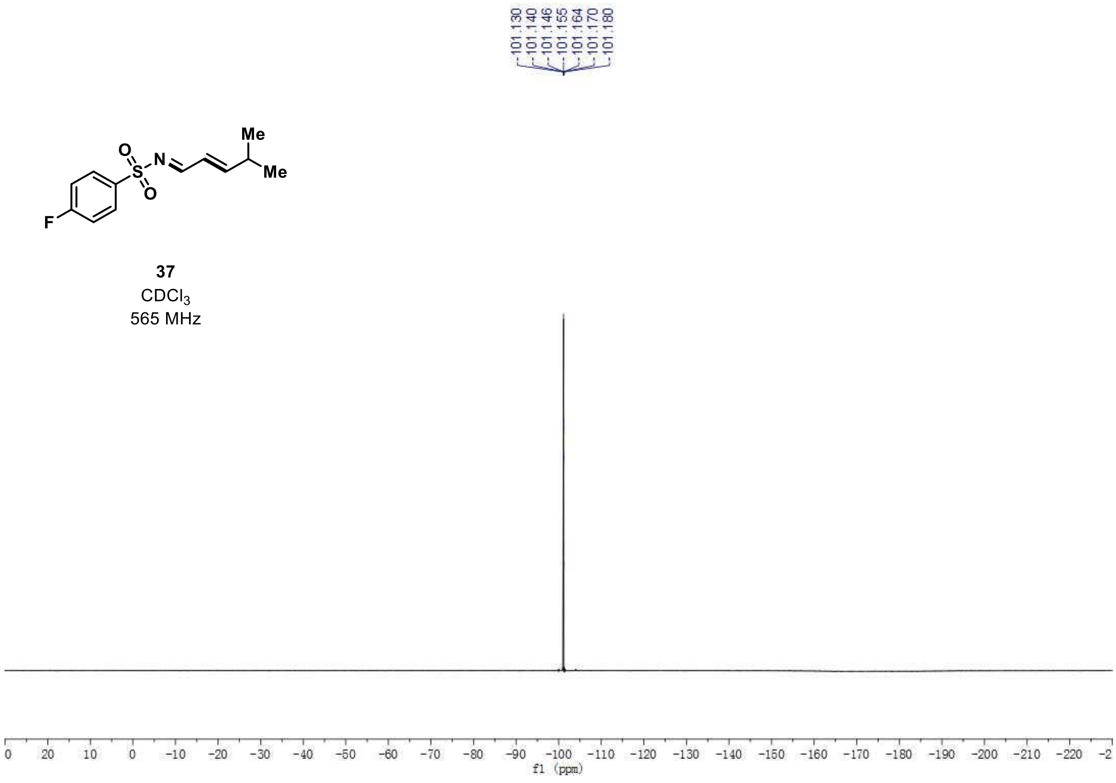

Supplementary Figure 123. ^{19}F NMR of compound 37. The sample has been recorded in 565 MHz, $CDCl_3$ at 25 °C.

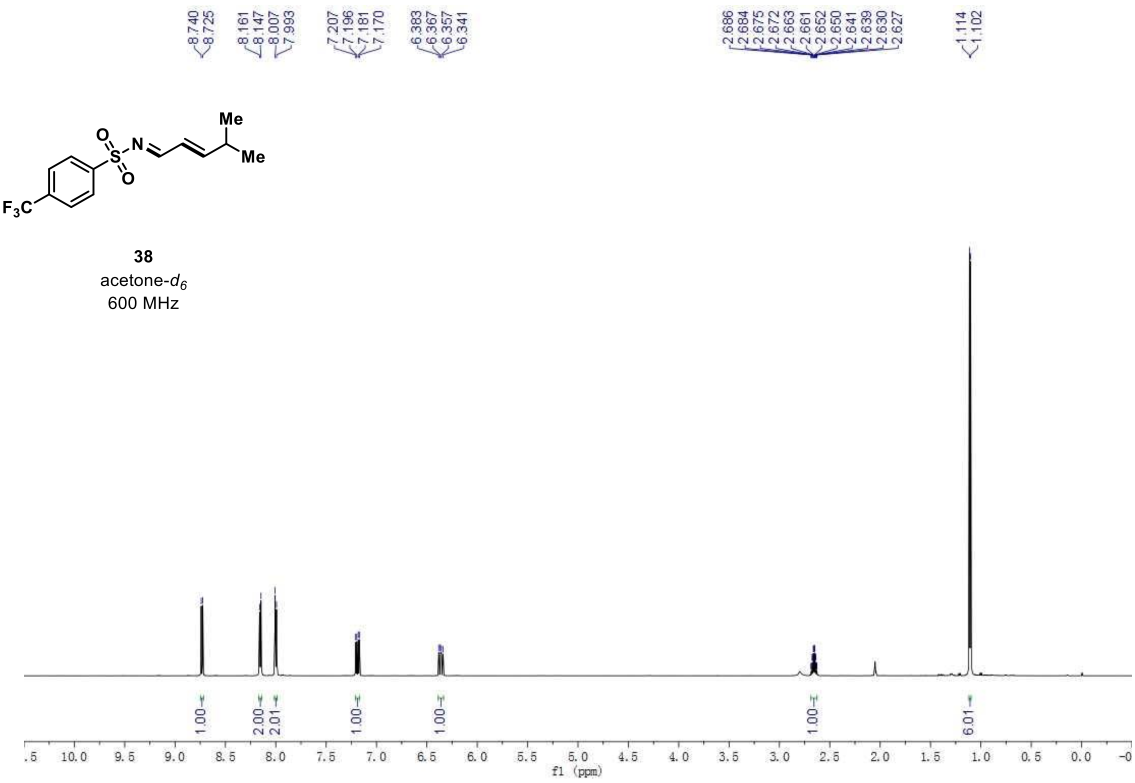

Supplementary Figure 124. ^{1}H NMR of compound 38. The sample has been recorded in 600 MHz, acetone-d_6 at 25 °C.

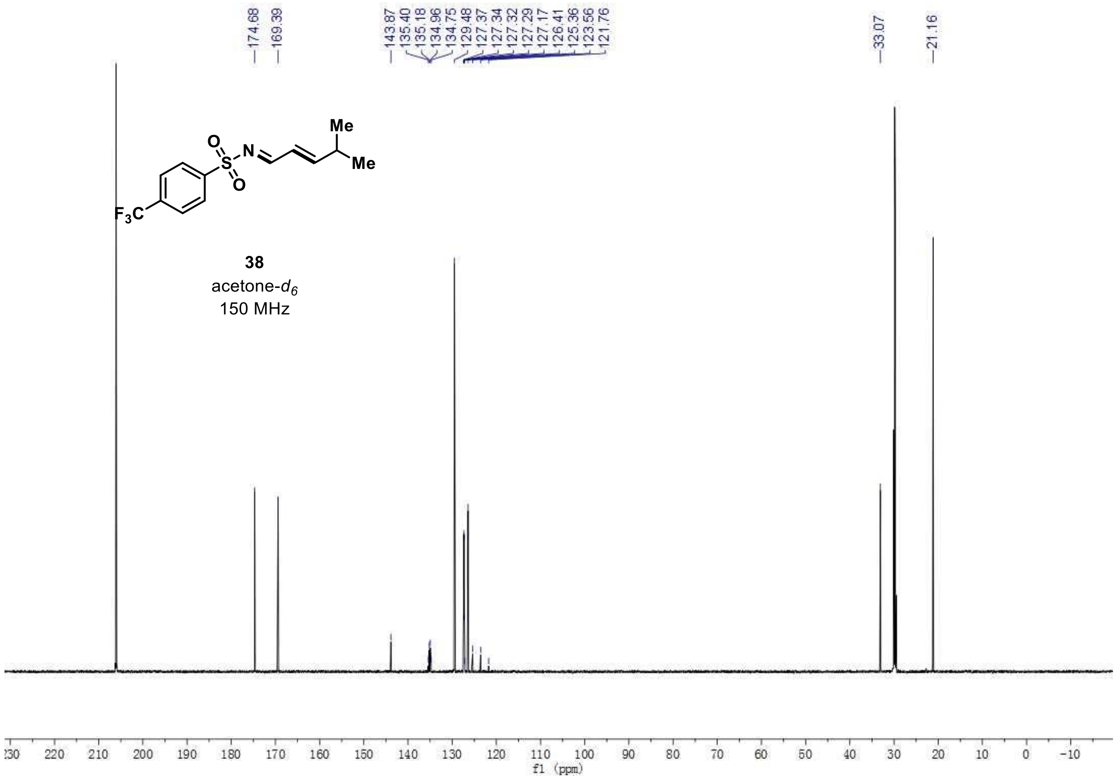

Supplementary Figure 125. ^{13}C NMR of compound 38. The sample has been recorded in 150 MHz, acetone-d_6 at 25 °C.

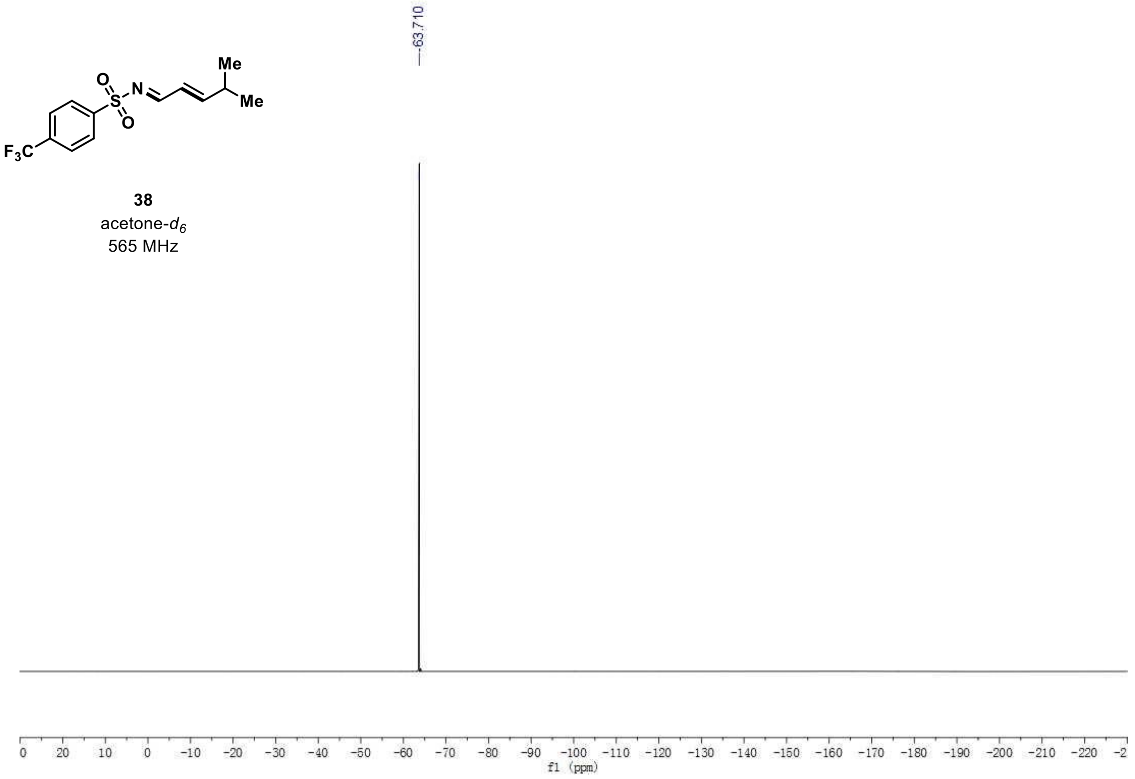

Supplementary Figure 126. ^{19}F NMR of compound 38. The sample has been recorded in 565 MHz, acetone-d_6 at 25 °C.

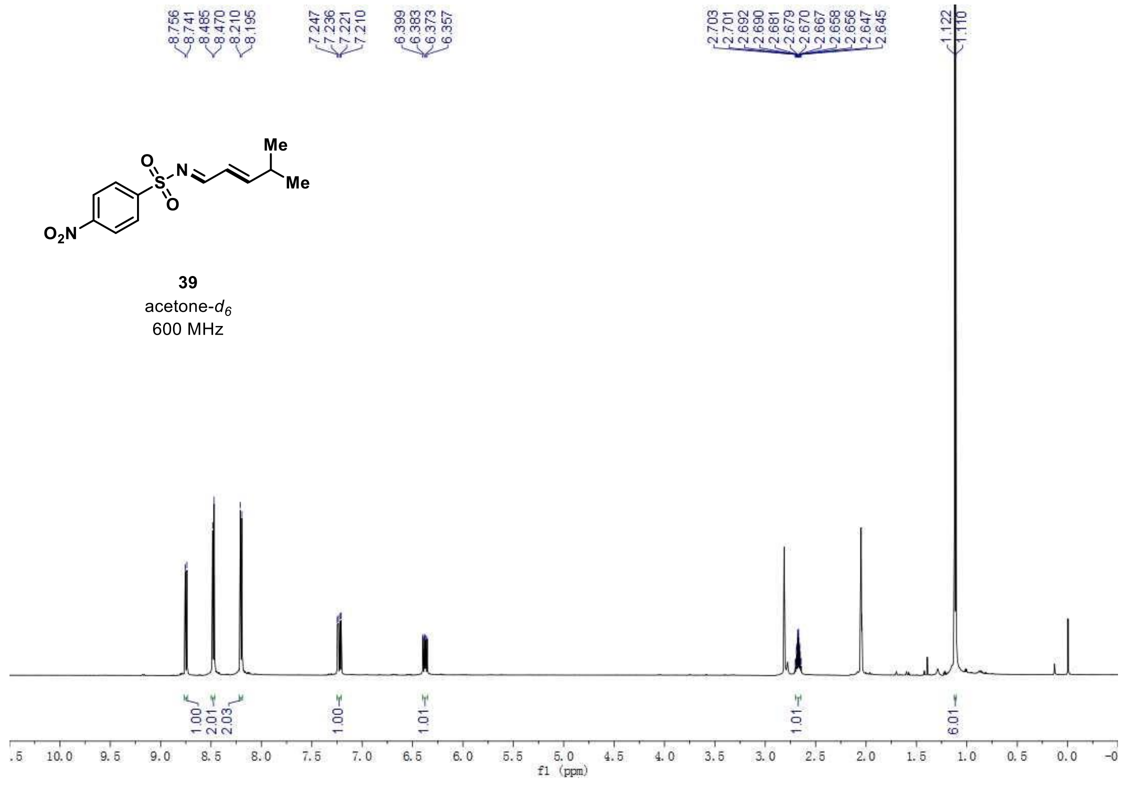

Supplementary Figure 127. ^{1}H NMR of compound 39. The sample has been recorded in 600 MHz, acetone-d_6 at 25 °C.

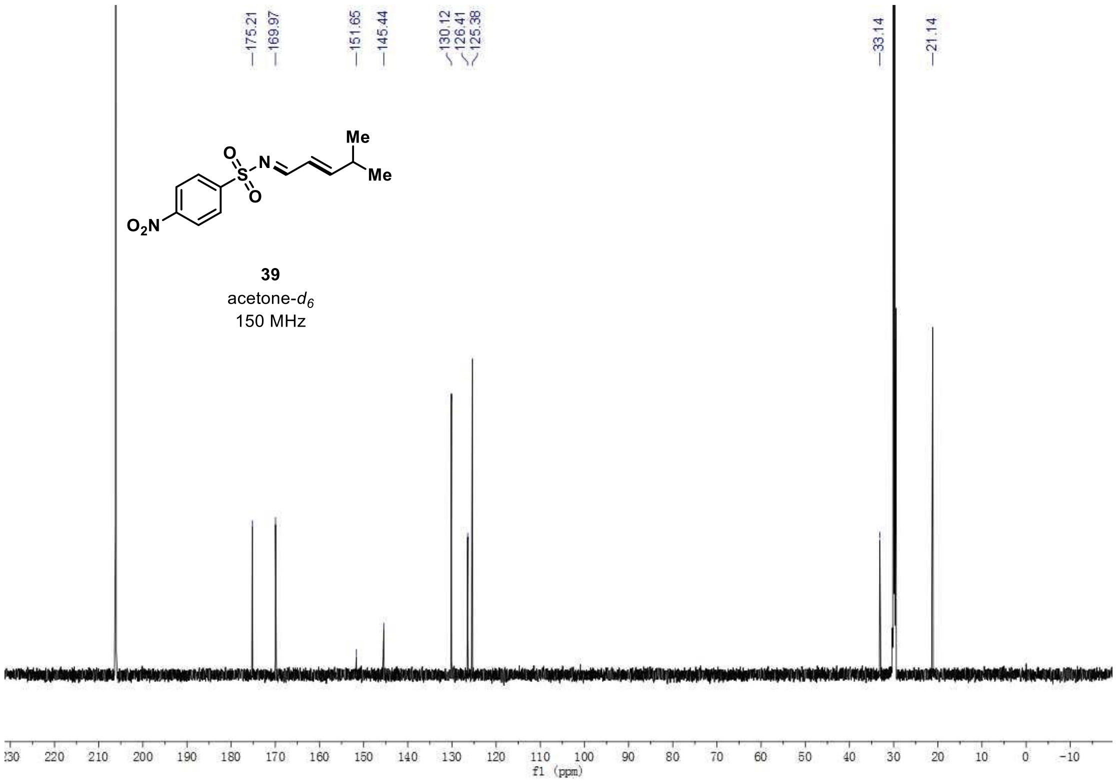

Supplementary Figure 128. ^{13}C NMR of compound 39. The sample has been recorded in 150 MHz, acetone-d_6 at 25 °C.

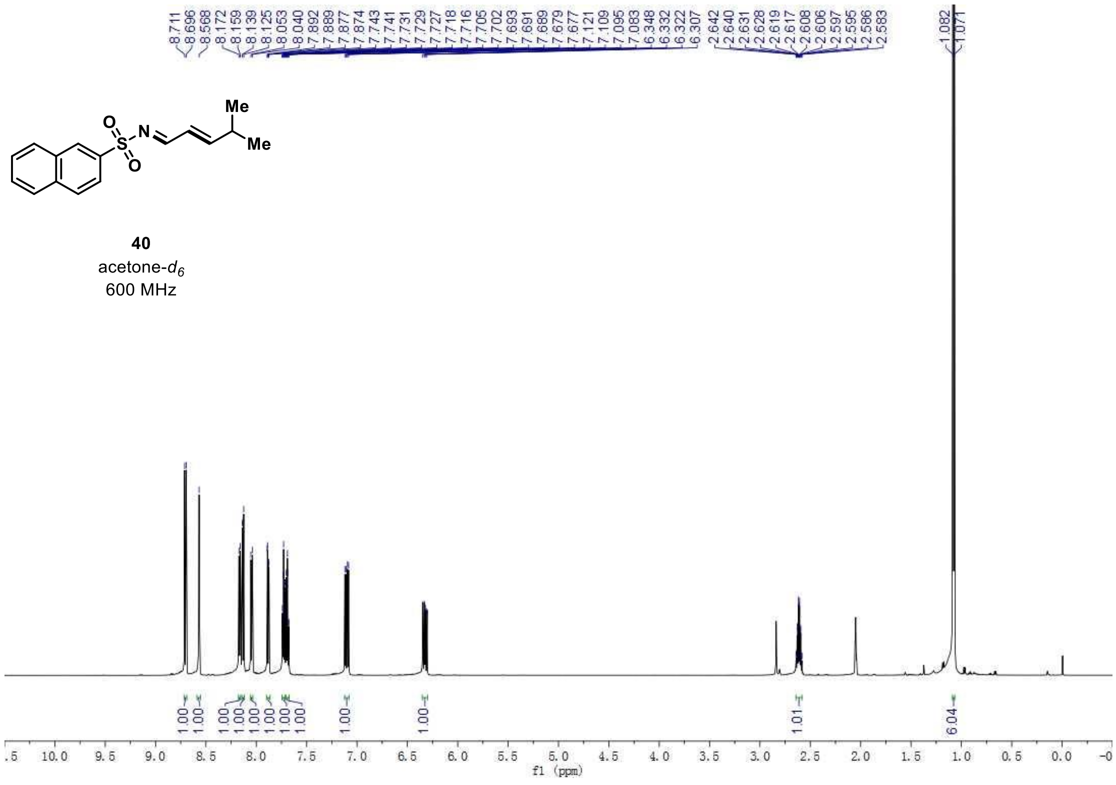

Supplementary Figure 129. ^{1}H NMR of compound 40. The sample has been recorded in 600 MHz, acetone-d_6 at 25 °C.

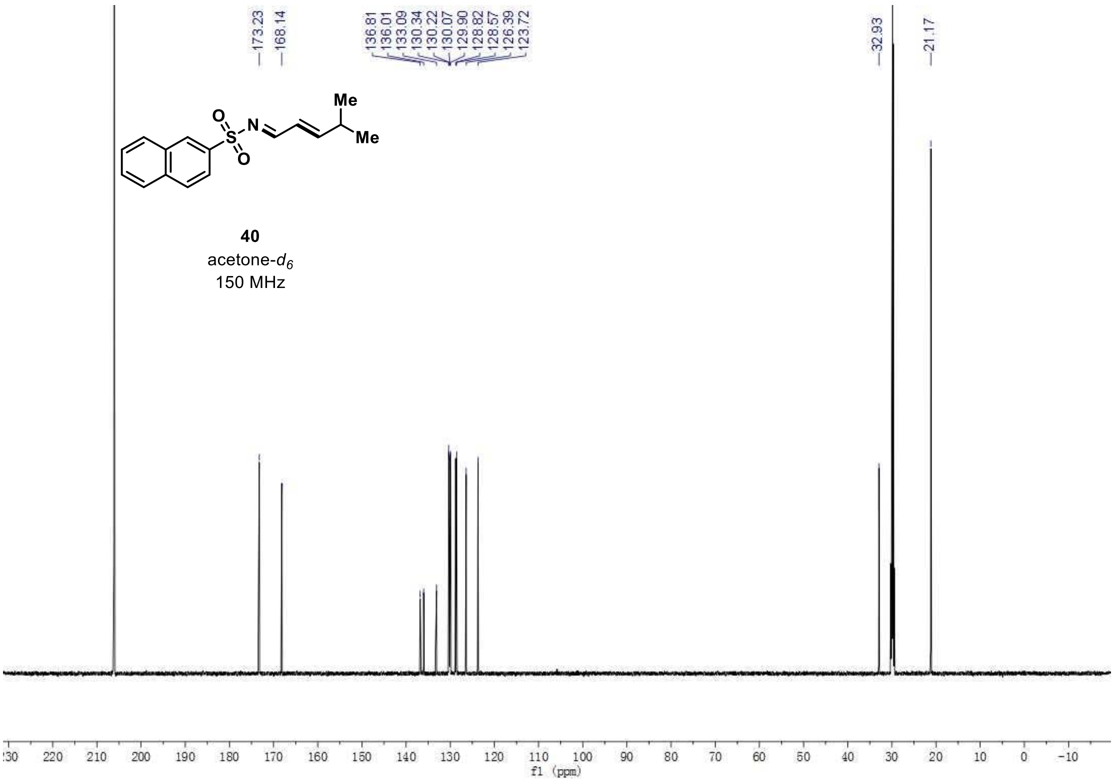

Supplementary Figure 130. ^{13}C NMR of compound 40. The sample has been recorded in 150 MHz, acetone-d_6 at 25 °C.

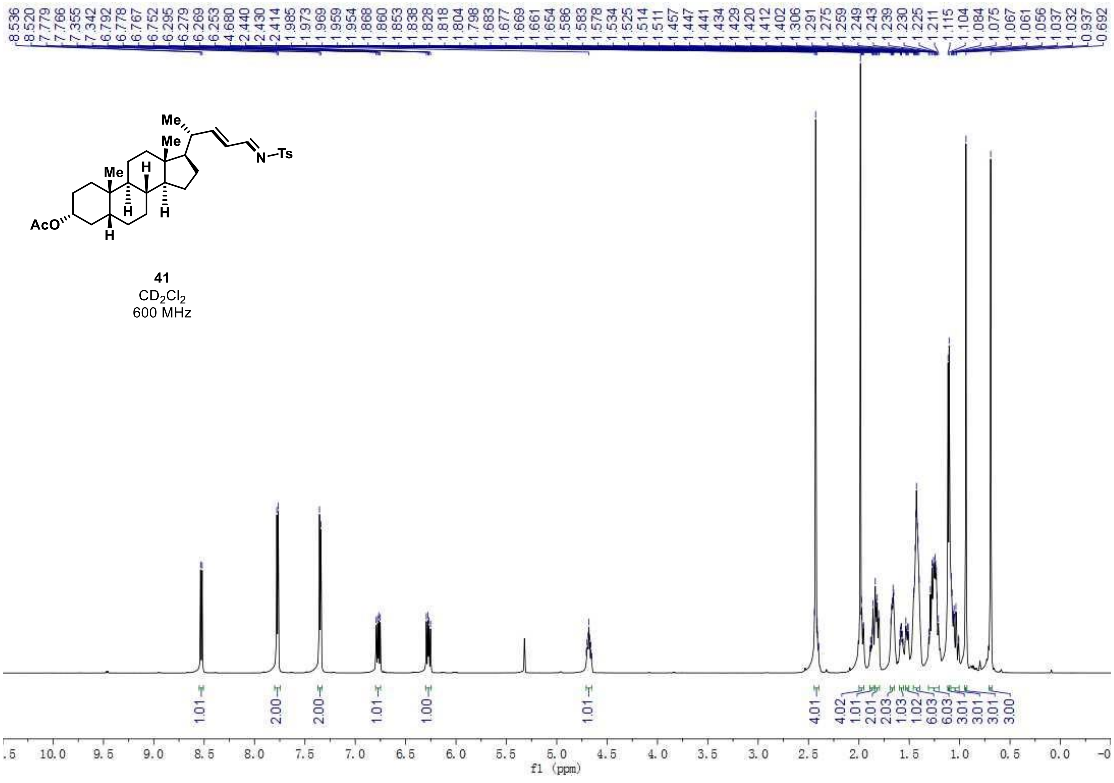

Supplementary Figure 131. ^{1}H NMR of compound 41. The sample has been recorded in 600 MHz, CD_2Cl_2 at 25 °C.

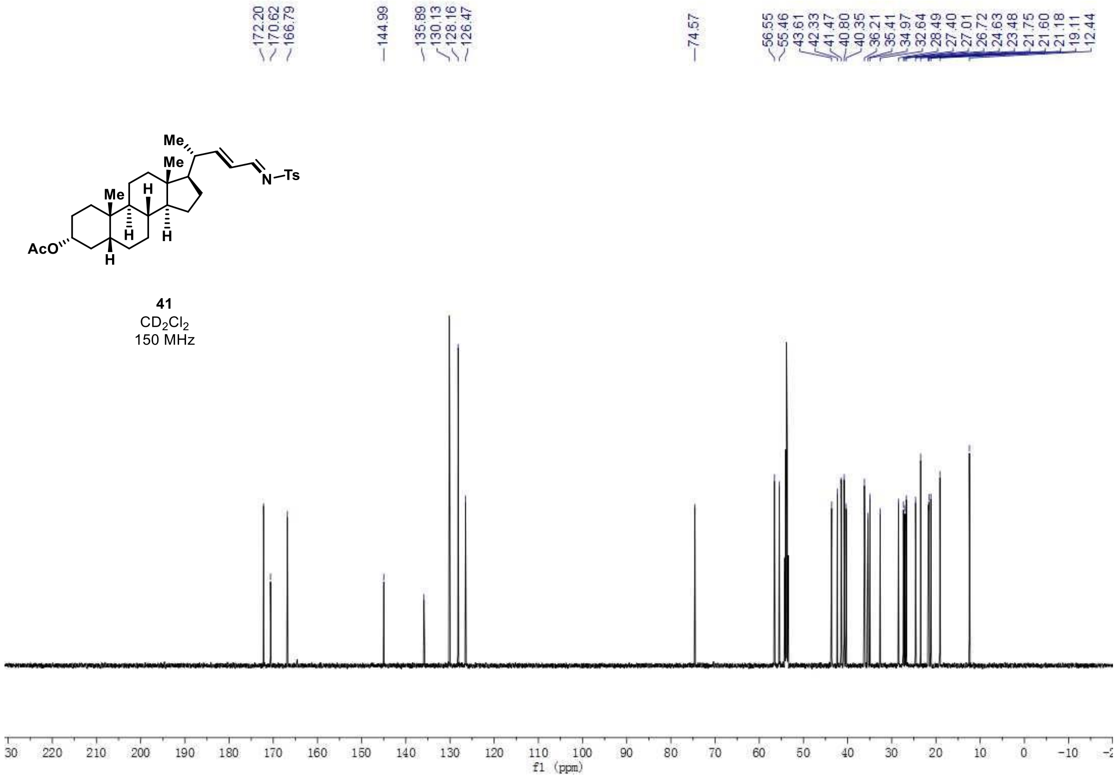

Supplementary Figure 132. ^{13}C NMR of compound 41. The sample has been recorded in 150 MHz, CD_2Cl_2 at 25 °C.

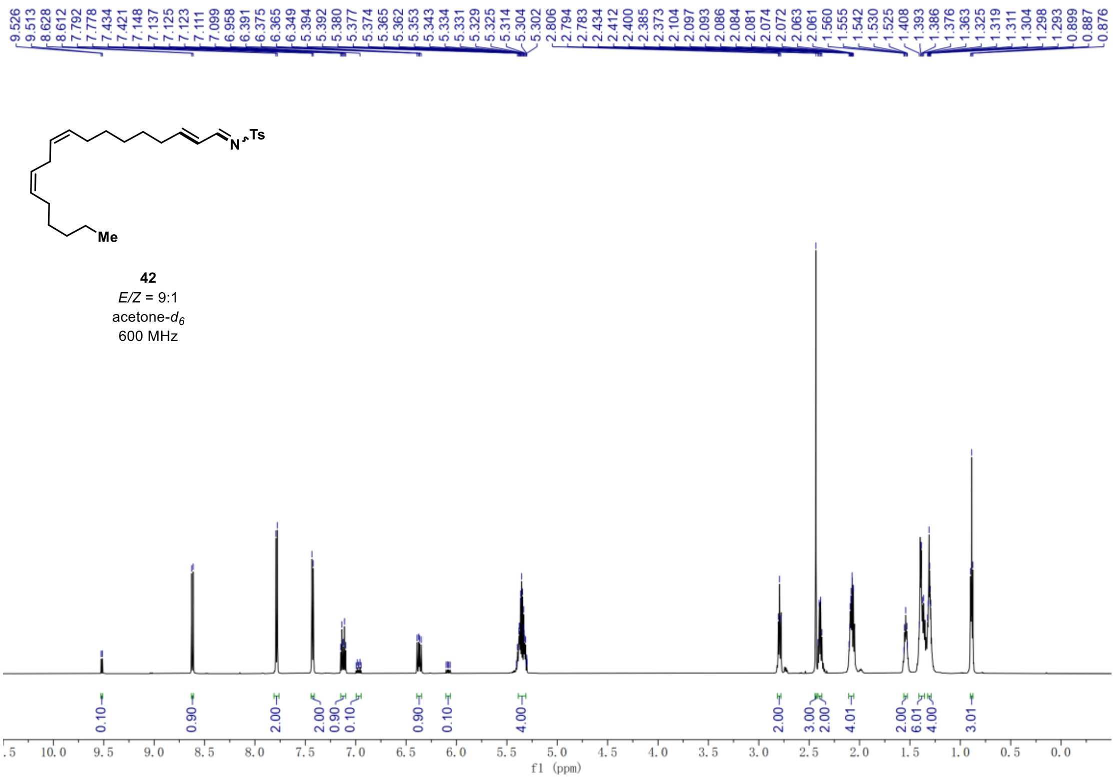

Supplementary Figure 133. ^{1}H NMR of compound 42. The sample has been recorded in 600 MHz, acetone-d_6 at 25 °C.

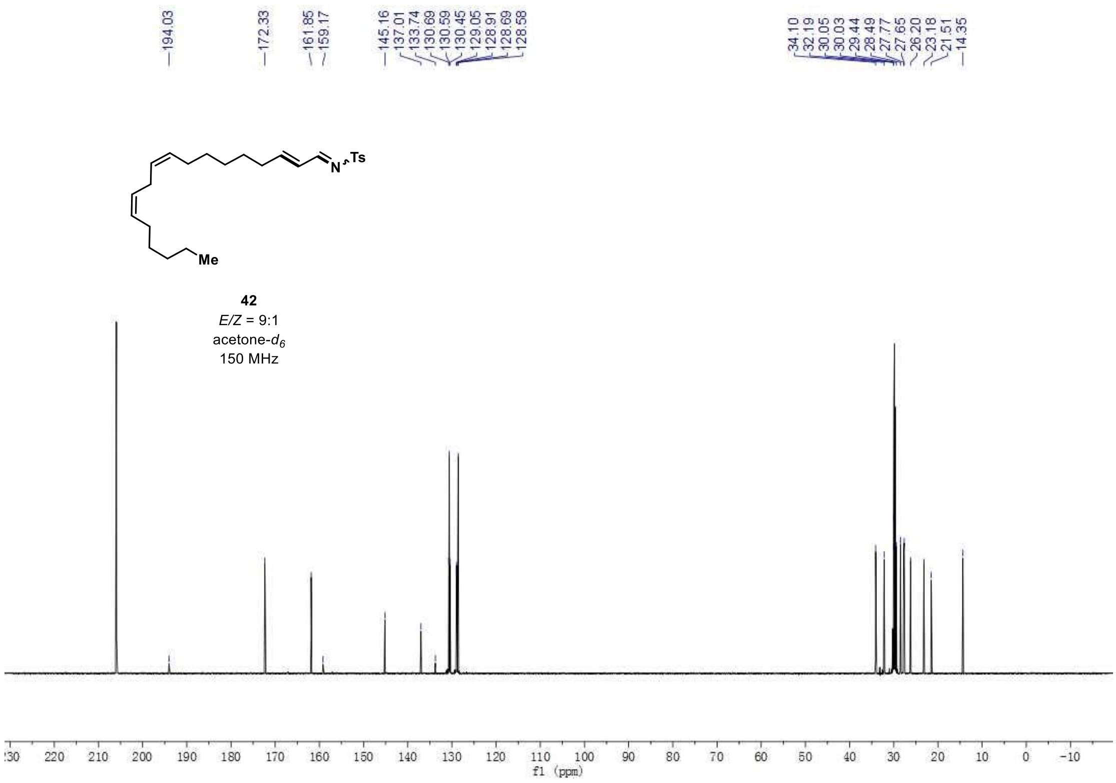

Supplementary Figure 134. ^{13}C NMR of compound 42. The sample has been recorded in 150 MHz, acetone-d_6 at 25 °C.

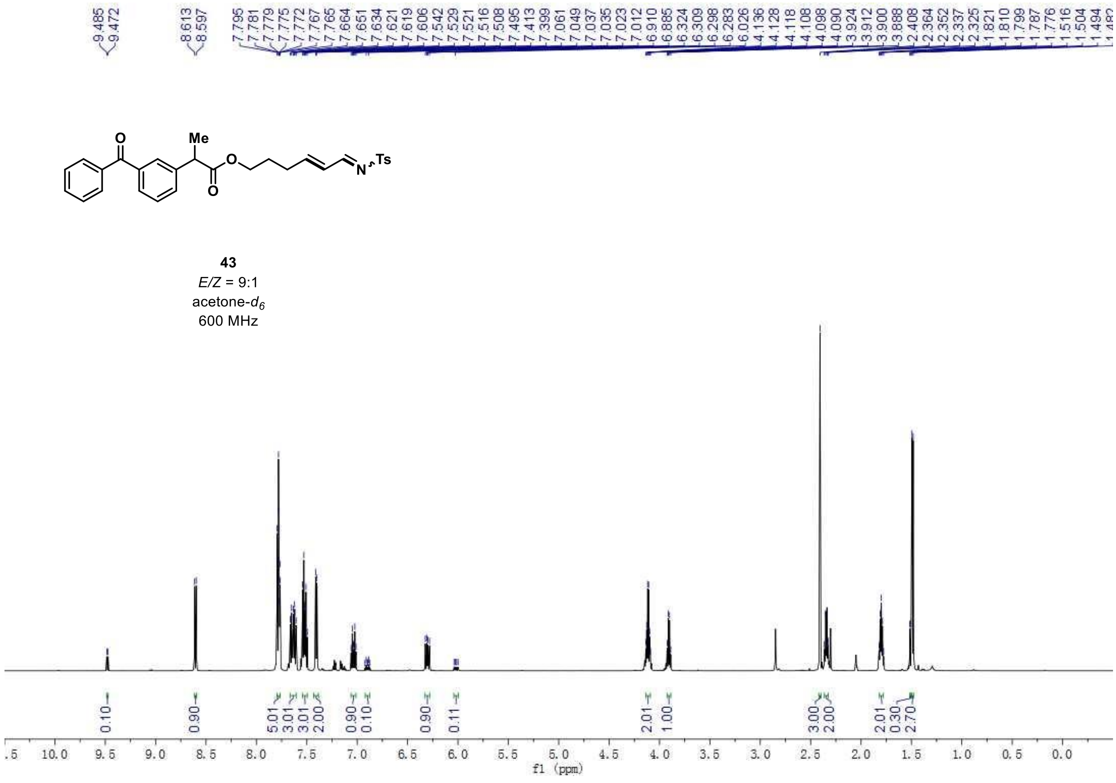

Supplementary Figure 135. ^{1}H NMR of compound 43. The sample has been recorded in 600 MHz, acetone-d_6 at 25 °C.

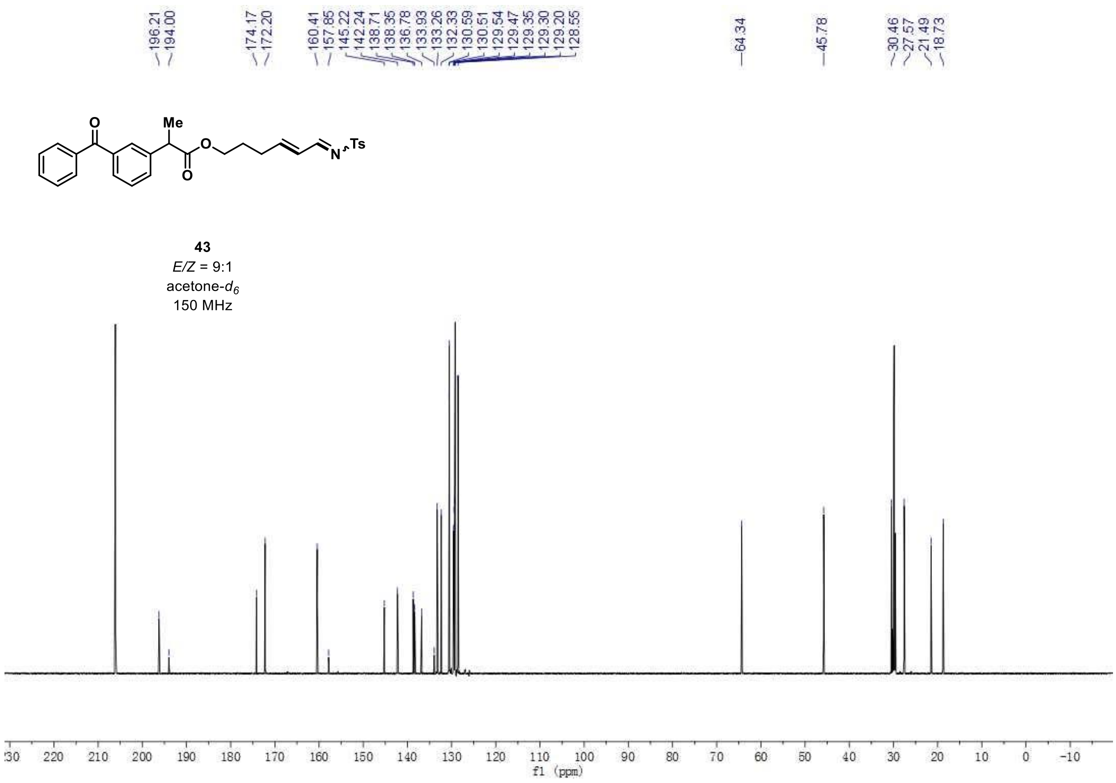

Supplementary Figure 136. ^{13}C NMR of compound 43. The sample has been recorded in 150 MHz, acetone-d_6 at 25 °C.

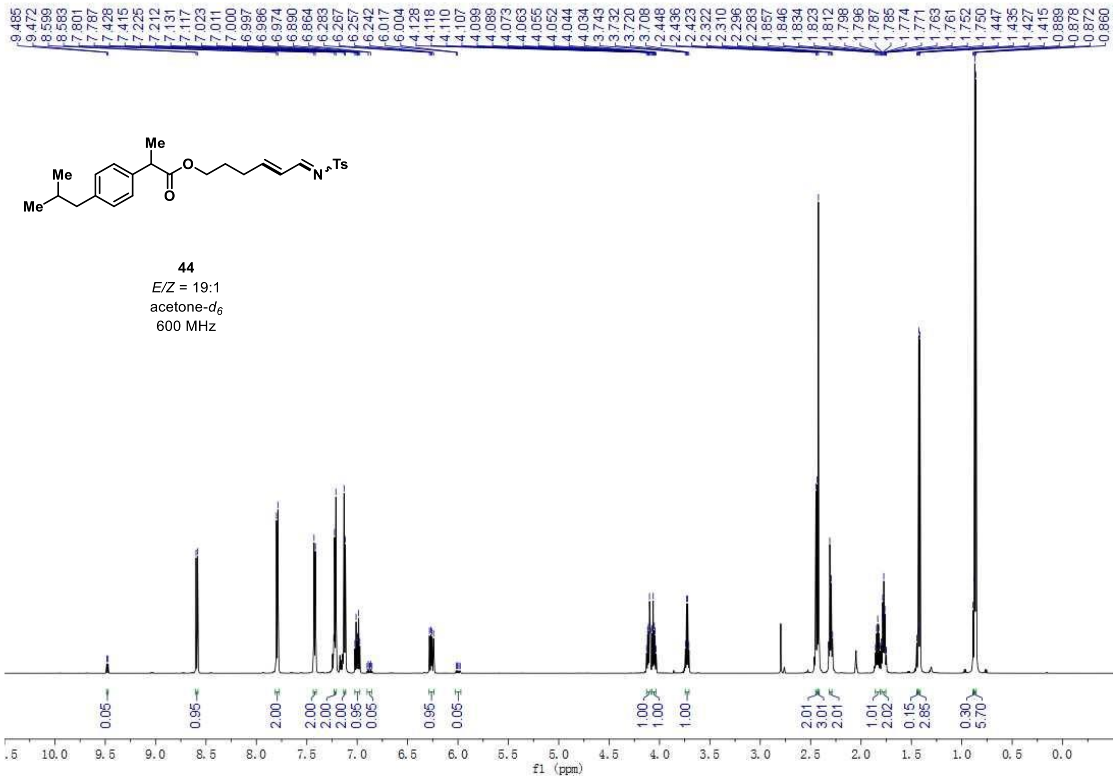

Supplementary Figure 137. ^{1}H NMR of compound 44. The sample has been recorded in 600 MHz, acetone-d_6 at 25 °C.

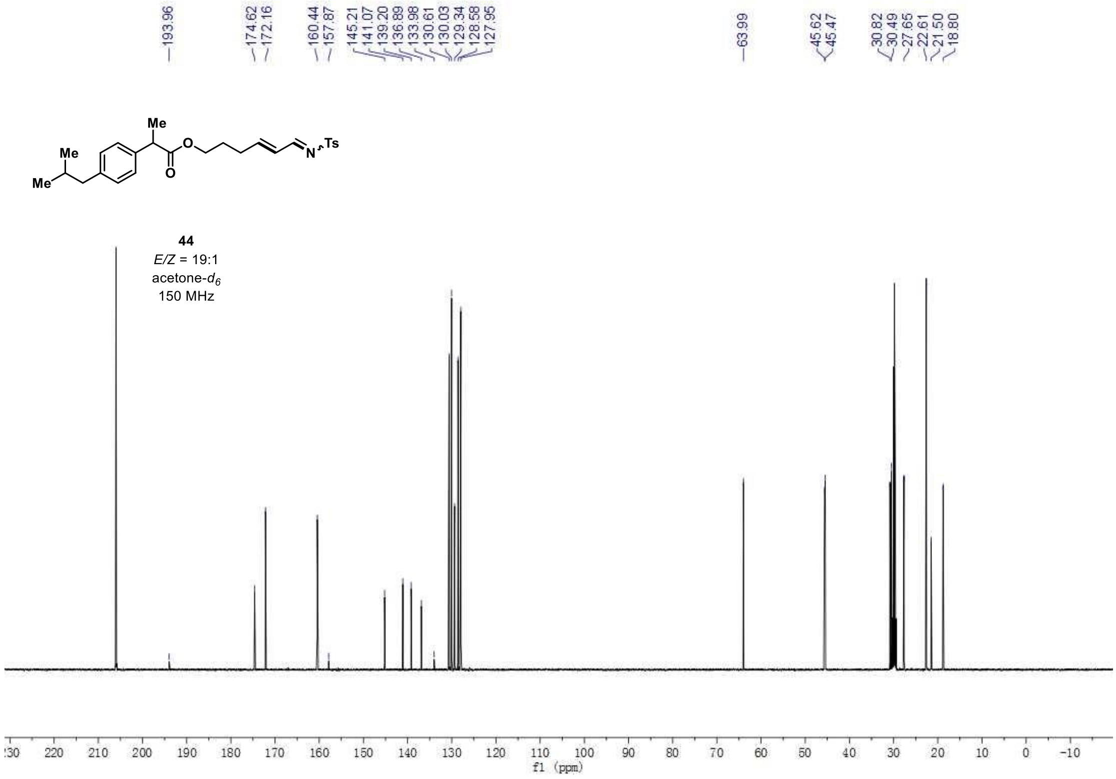

Supplementary Figure 138. ^{13}C NMR of compound 44. The sample has been recorded in 150 MHz, acetone-d_6 at 25 °C.

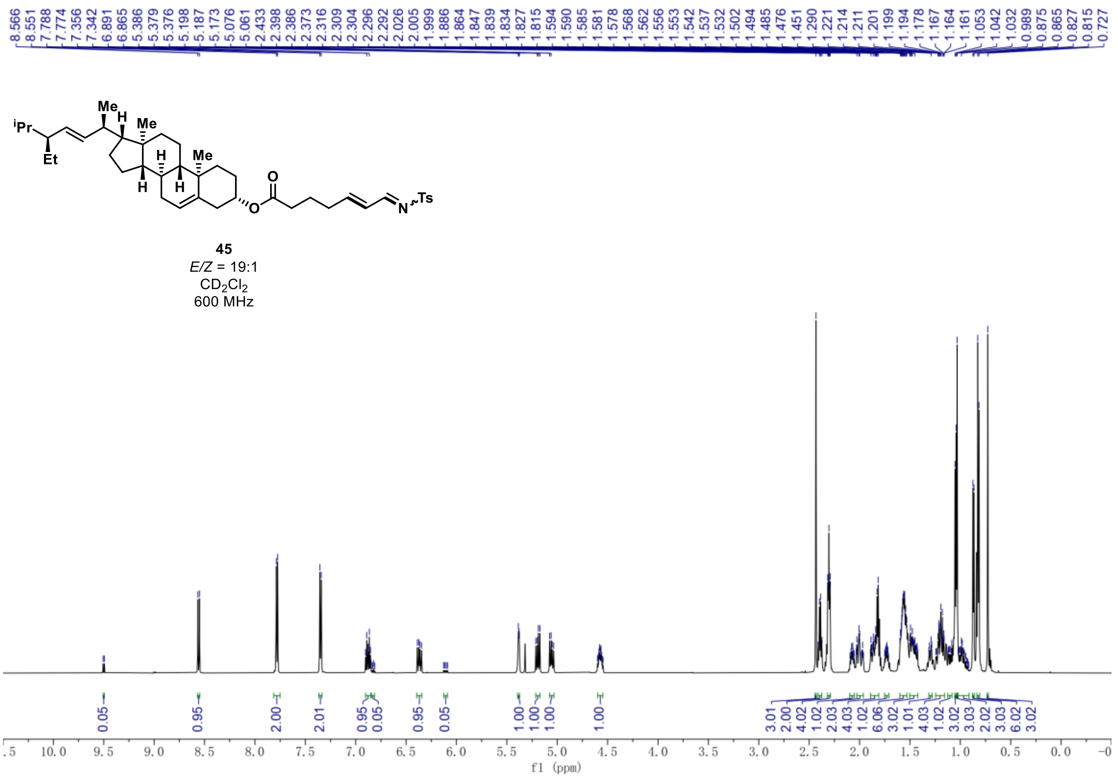

Supplementary Figure 139. ^{1}H NMR of compound 45. The sample has been recorded in 600 MHz, CD_2Cl_2 at 25 °C.

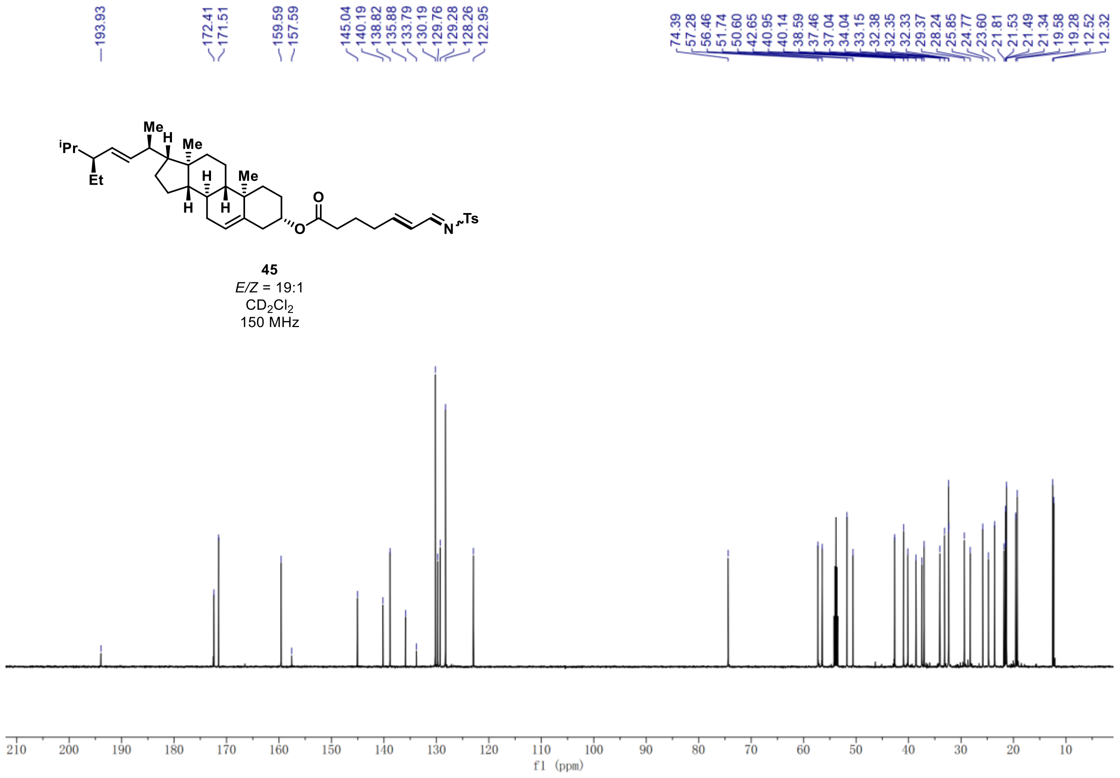

Supplementary Figure 140. ^{13}C NMR of compound 45. The sample has been recorded in 150 MHz, CD_2Cl_2 at 25 °C.

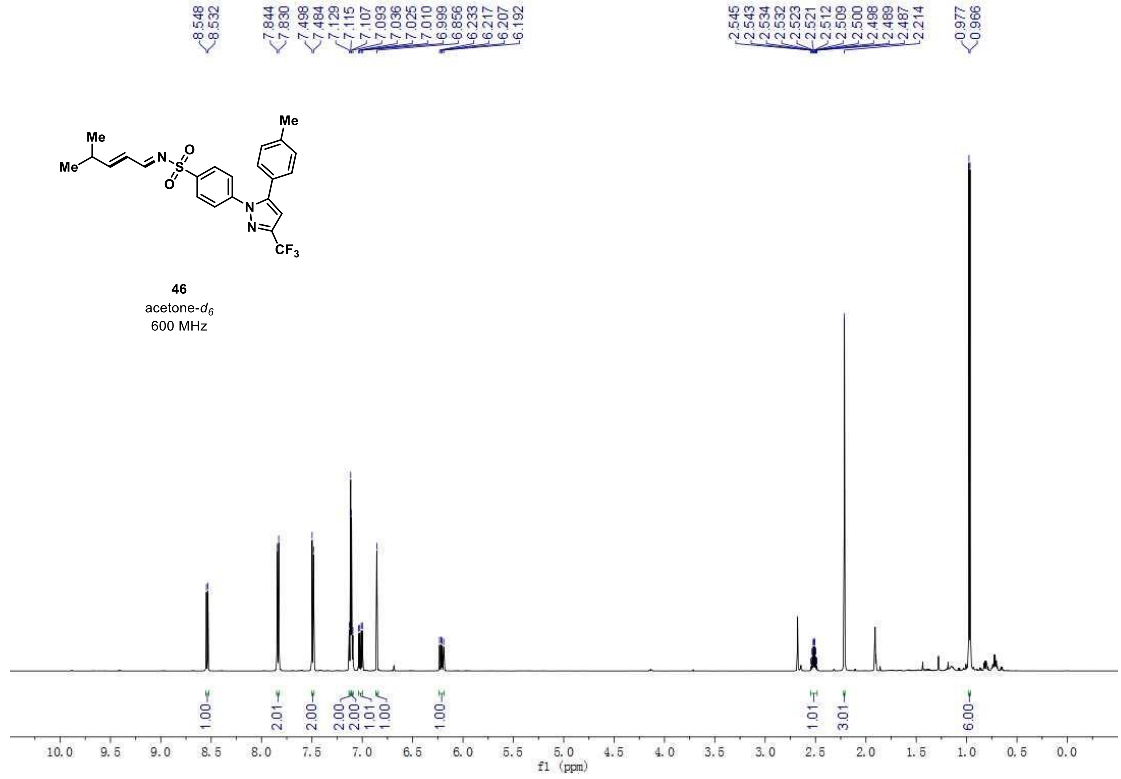

Supplementary Figure 141. ^{1}H NMR of compound 46. The sample has been recorded in 600 MHz, acetone-d_6 at 25 °C.

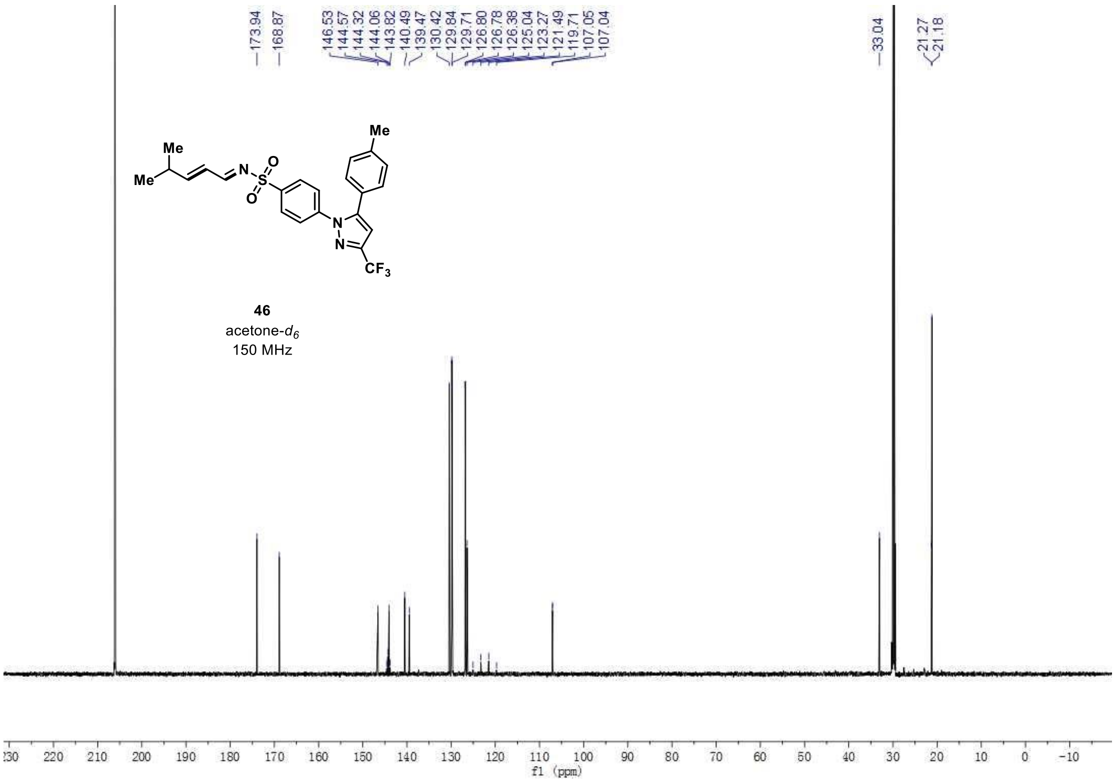

Supplementary Figure 142. ^{13}C NMR of compound 46. The sample has been recorded in 150 MHz, acetone-*d*$_6$ at 25 °C.

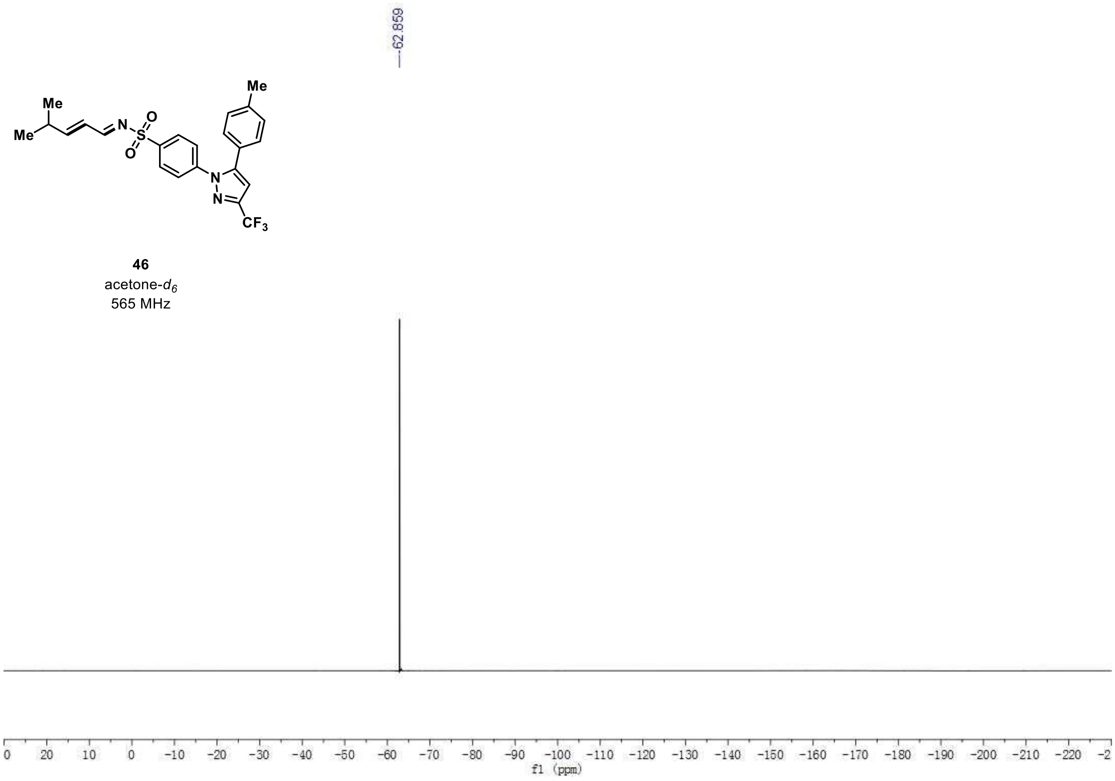

Supplementary Figure 143. ^{19}F NMR of compound 46. The sample has been recorded in 565 MHz, acetone-*d*$_6$ at 25 °C.

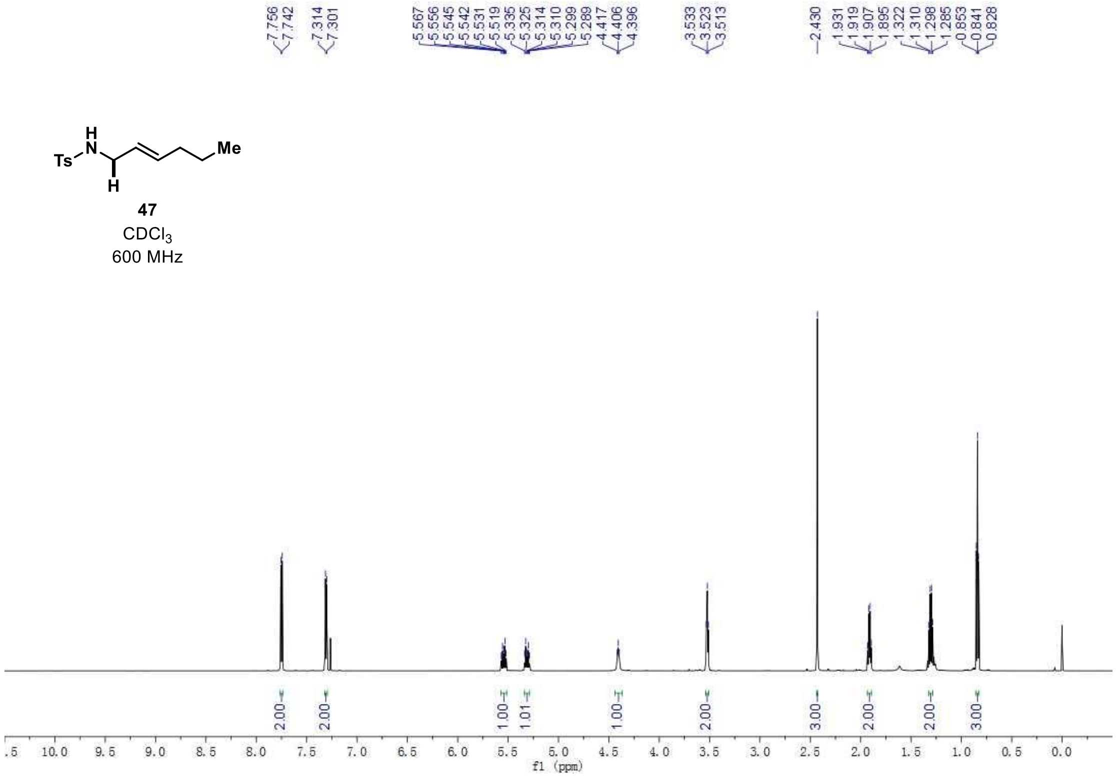

Supplementary Figure 144. ^{1}H NMR of compound 47. The sample has been recorded in 600 MHz, $CDCl_3$ at 25 °C.

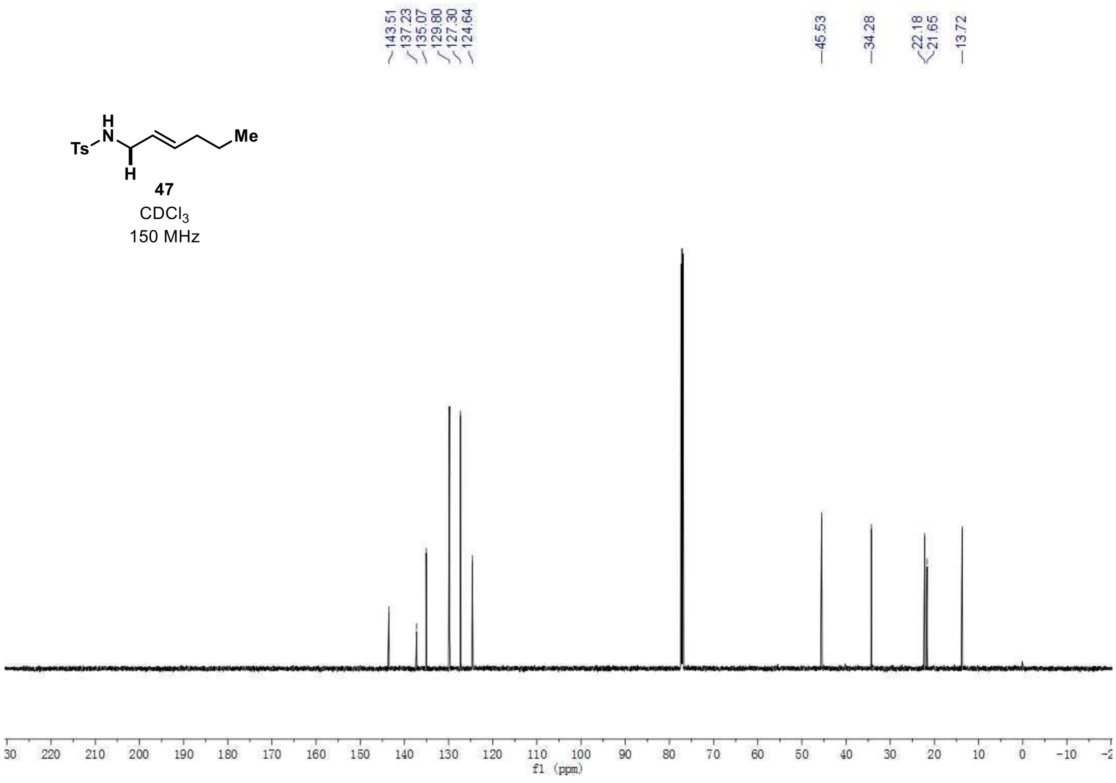

Supplementary Figure 145. ^{13}C NMR of compound 47. The sample has been recorded in 150 MHz, $CDCl_3$ at 25 °C.

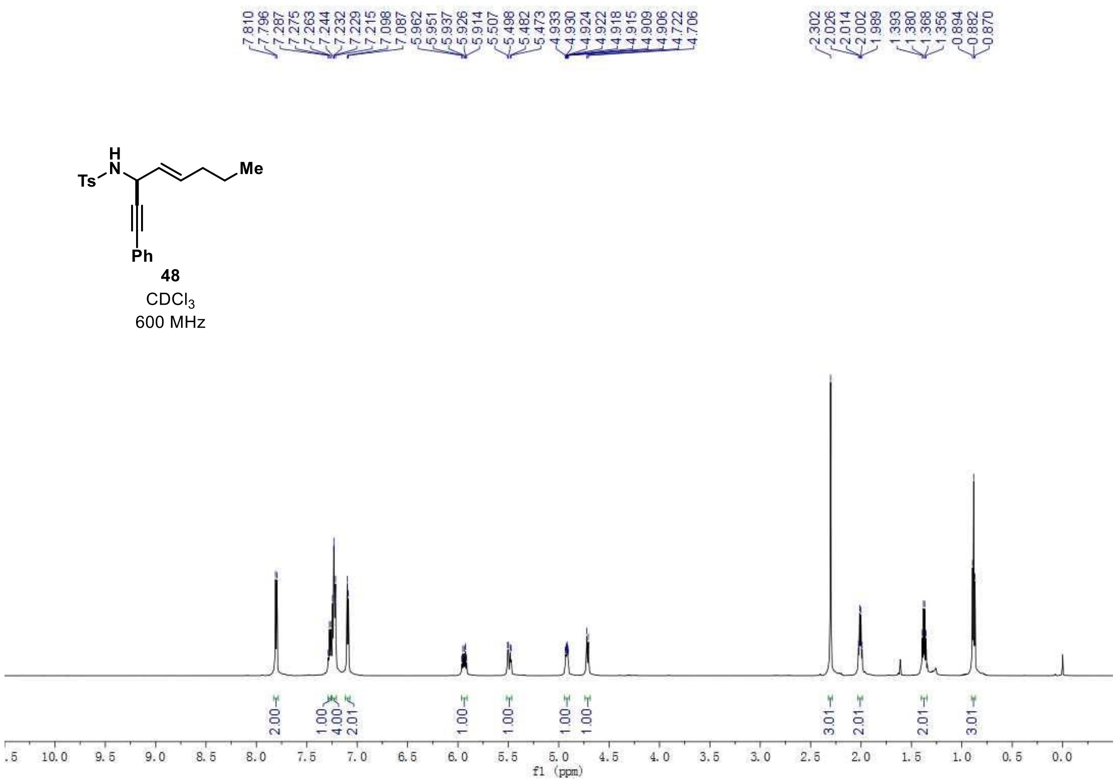

Supplementary Figure 146. ^{1}H NMR of compound 48. The sample has been recorded in 600 MHz, $CDCl_3$ at 25 °C.

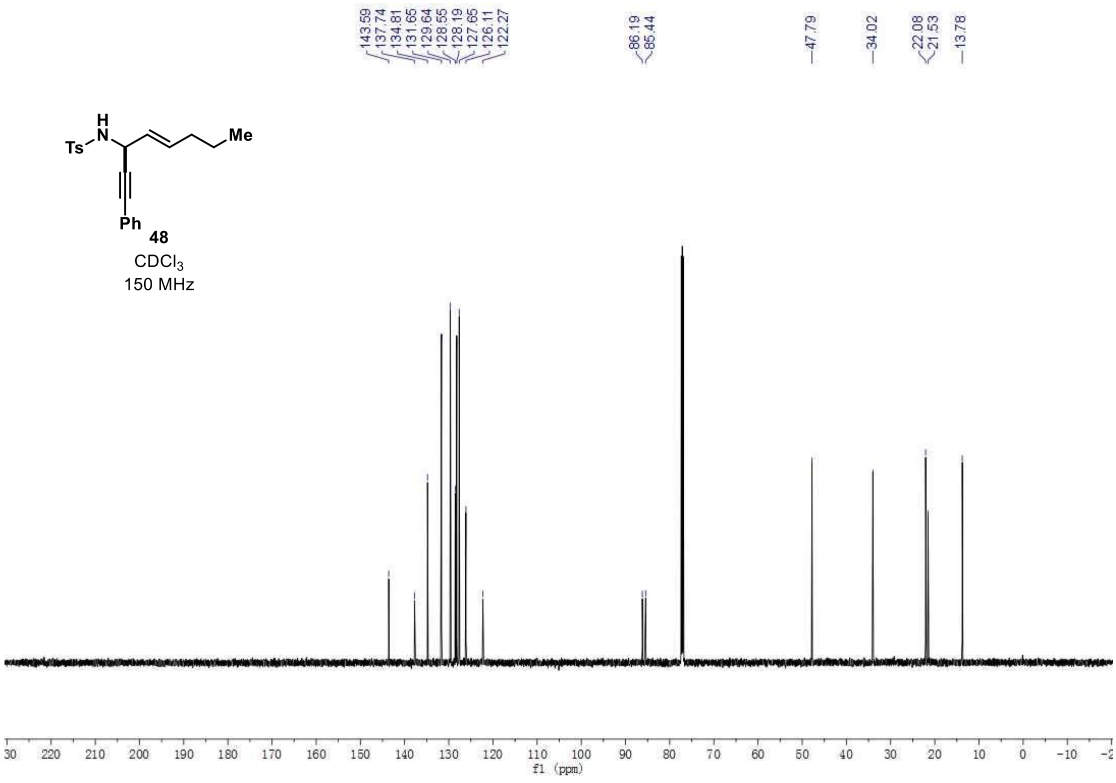

Supplementary Figure 147. ^{13}C NMR of compound 48. The sample has been recorded in 150 MHz, $CDCl_3$ at 25 °C.

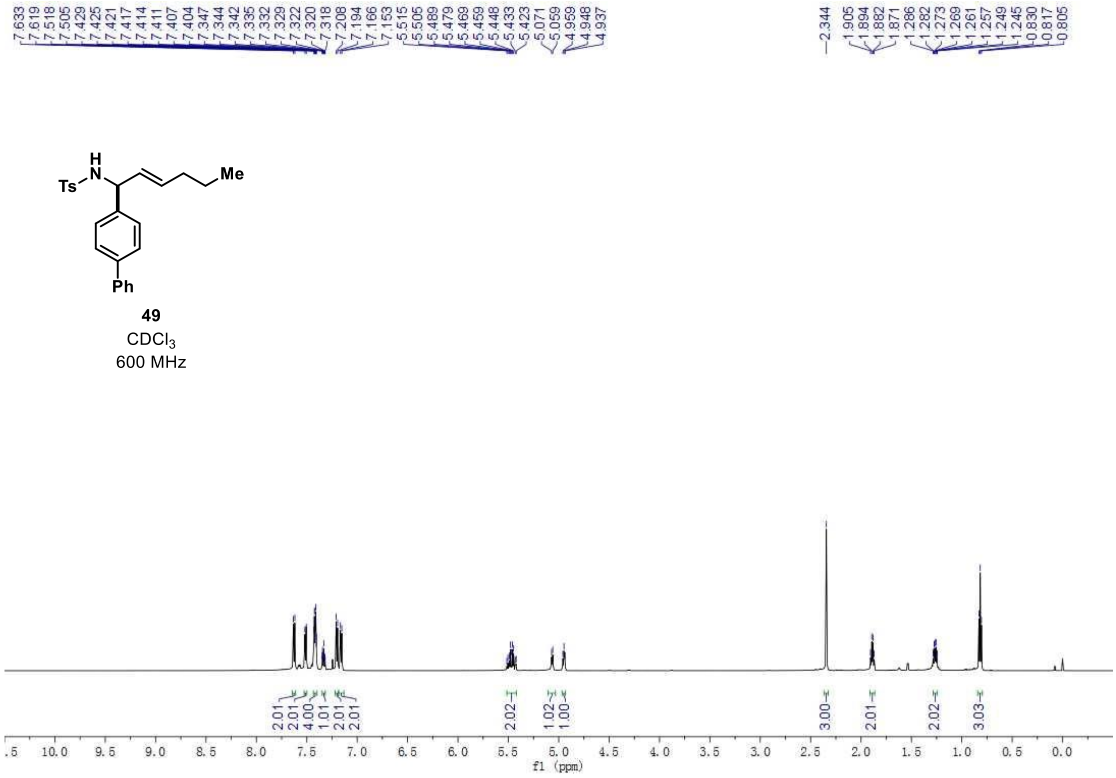

Supplementary Figure 148. ^{1}H NMR of compound 49. The sample has been recorded in 600 MHz, $CDCl_3$ at 25 °C.

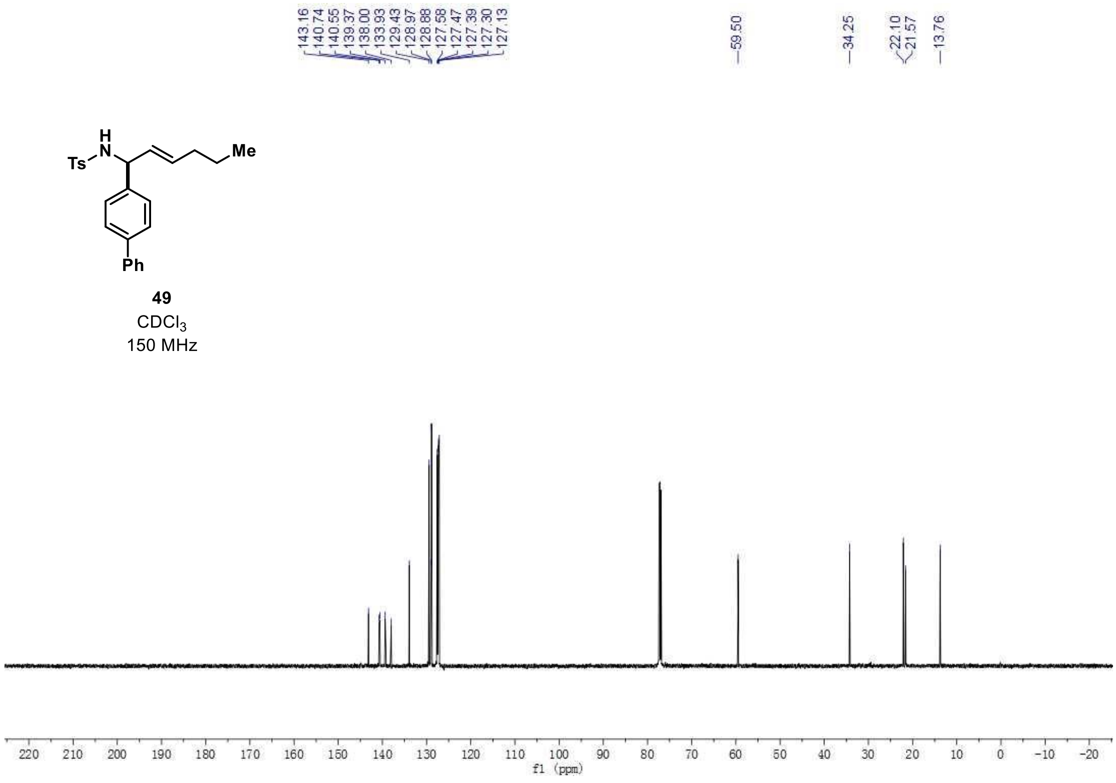

Supplementary Figure 149. ^{13}C NMR of compound 49. The sample has been recorded in 150 MHz, $CDCl_3$ at 25 °C.

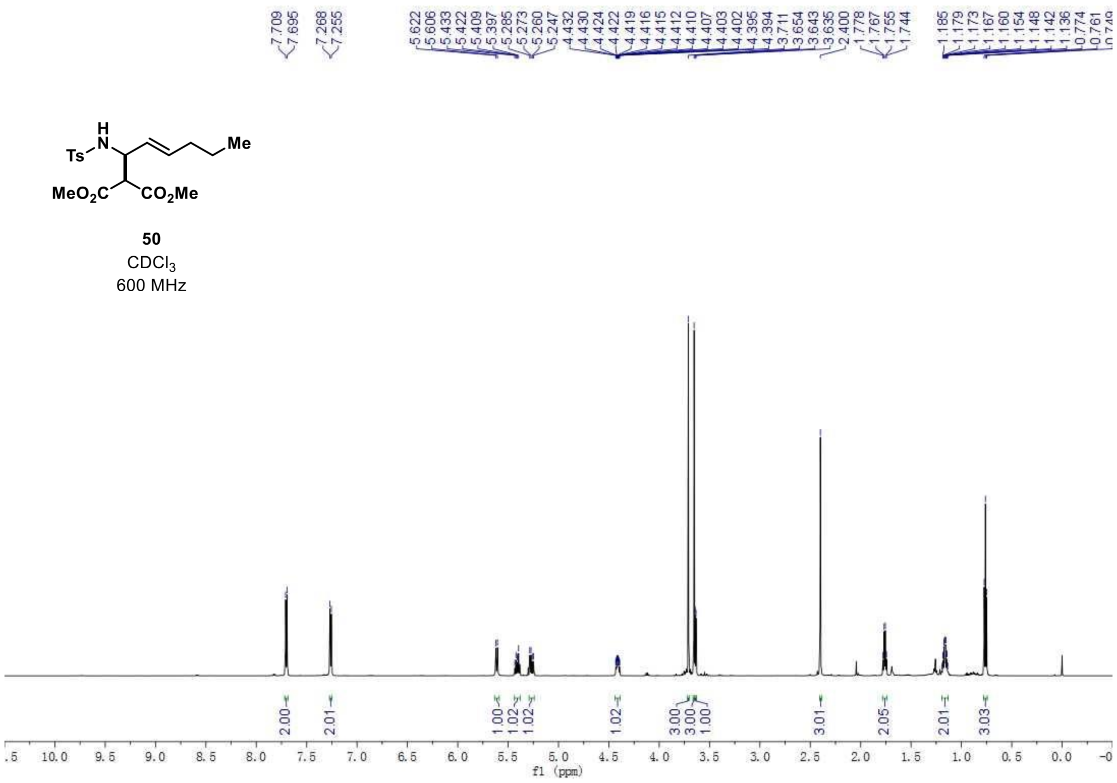

Supplementary Figure 150. ^{1}H NMR of compound 50. The sample has been recorded in 600 MHz, $CDCl_3$ at 25 °C.

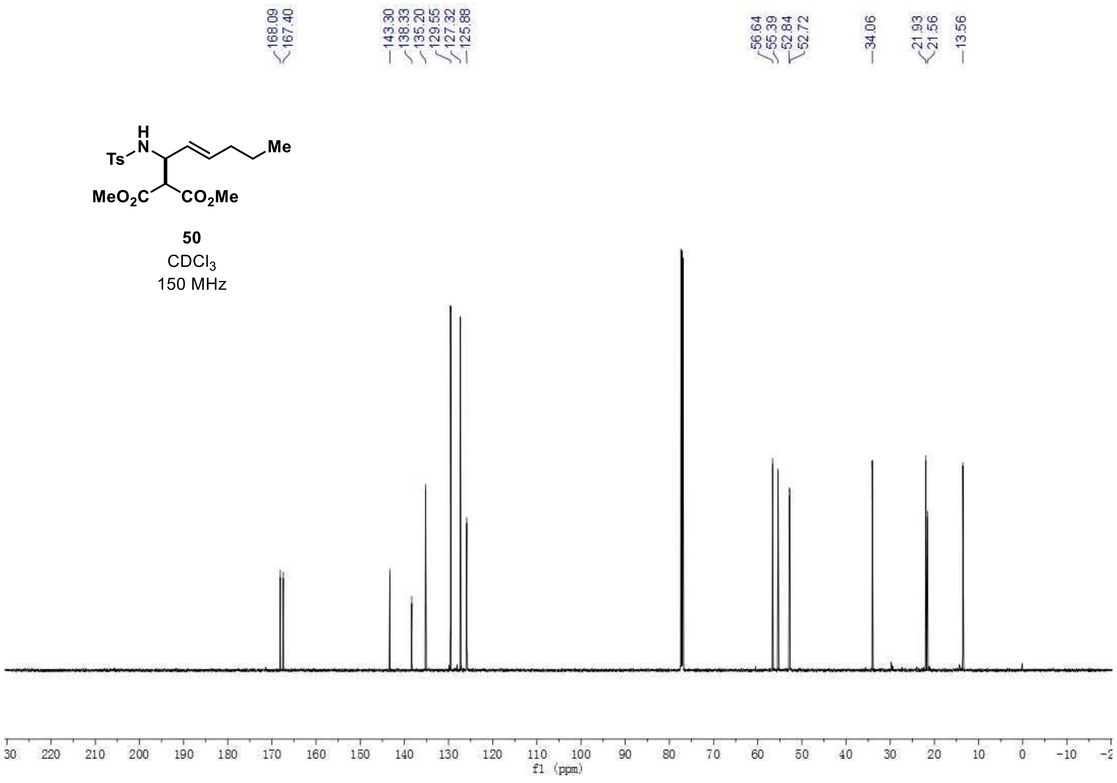

Supplementary Figure 151. ^{13}C NMR of compound 50. The sample has been recorded in 150 MHz, $CDCl_3$ at 25 °C.

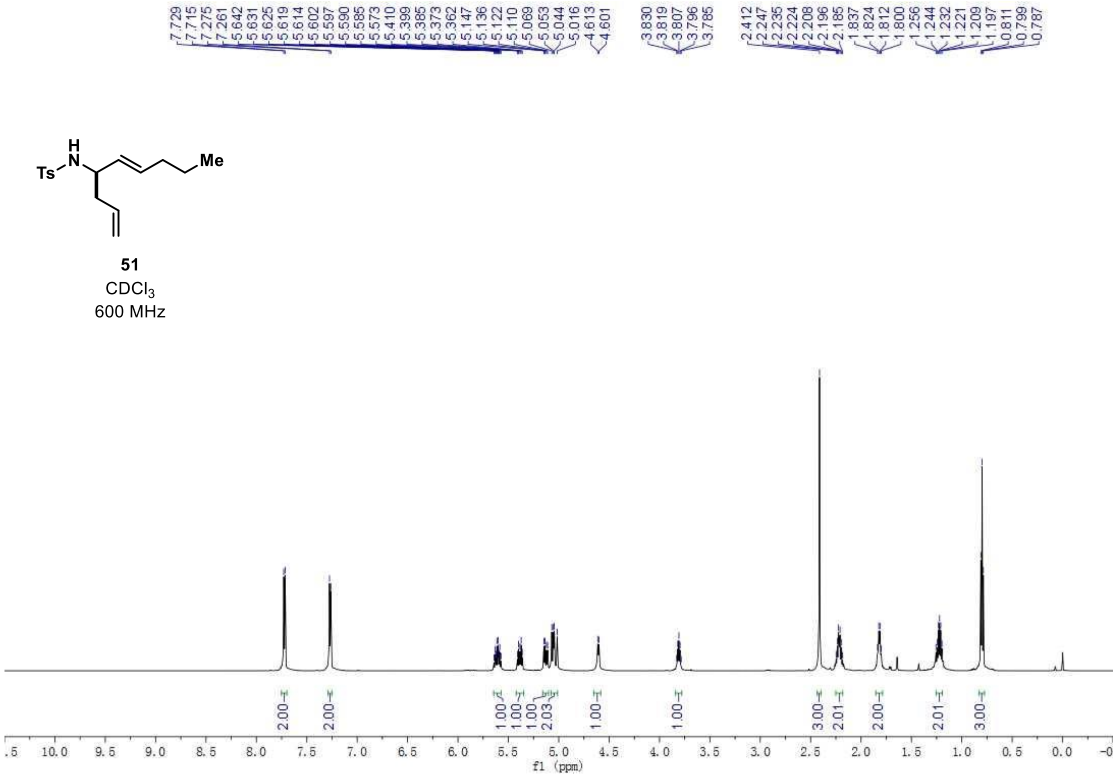

Supplementary Figure 152. ^1H NMR of compound 51. The sample has been recorded in 600 MHz, $CDCl_3$ at 25 °C.

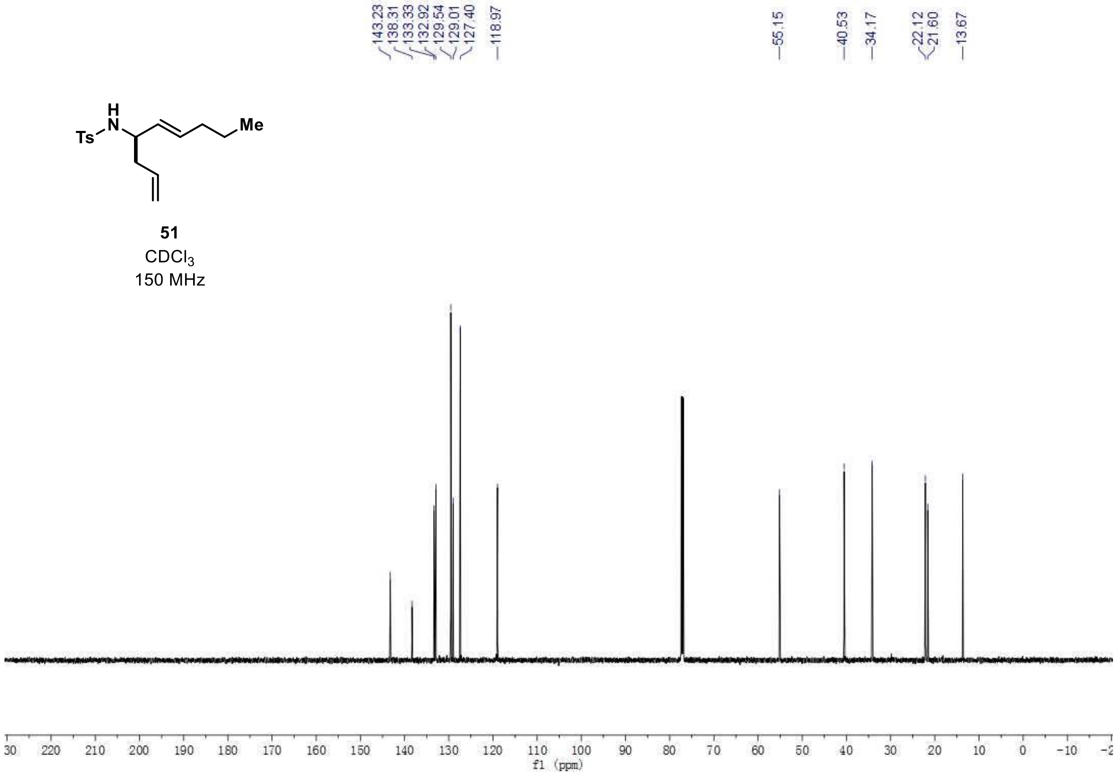

Supplementary Figure 153. ^{13}C NMR of compound 51. The sample has been recorded in 150 MHz, $CDCl_3$ at 25 °C.

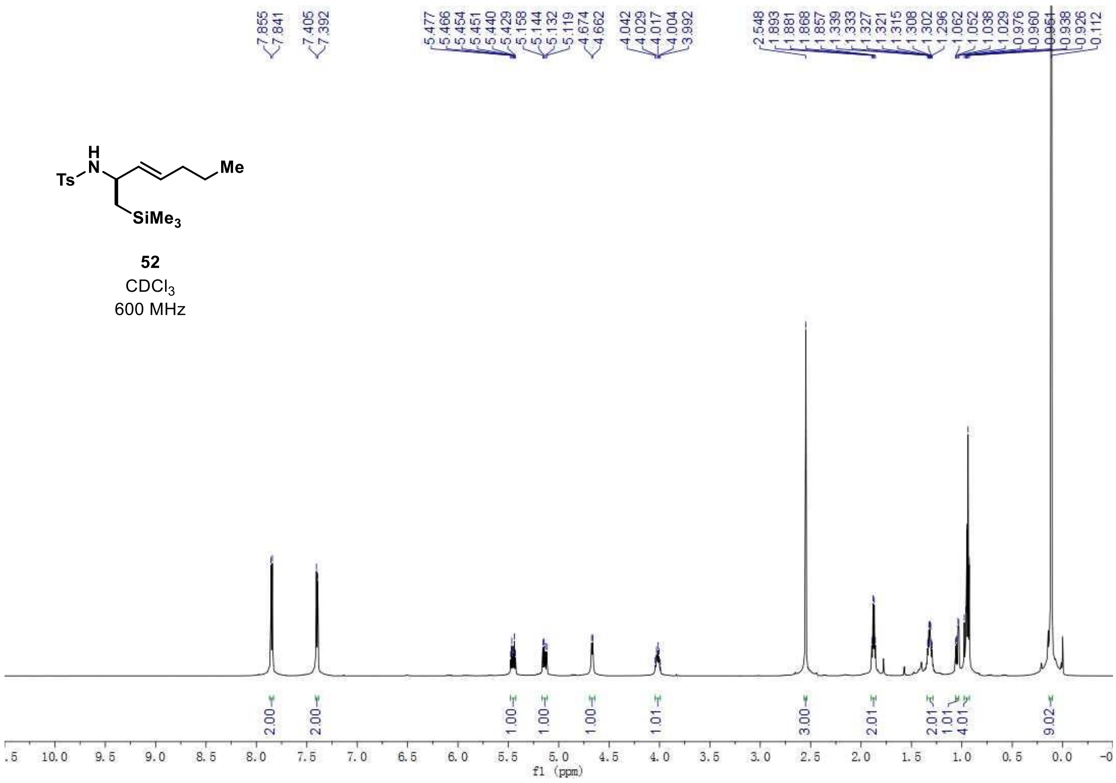

Supplementary Figure 154. ^{1}H NMR of compound 52. The sample has been recorded in 600 MHz, $CDCl_3$ at 25 °C.

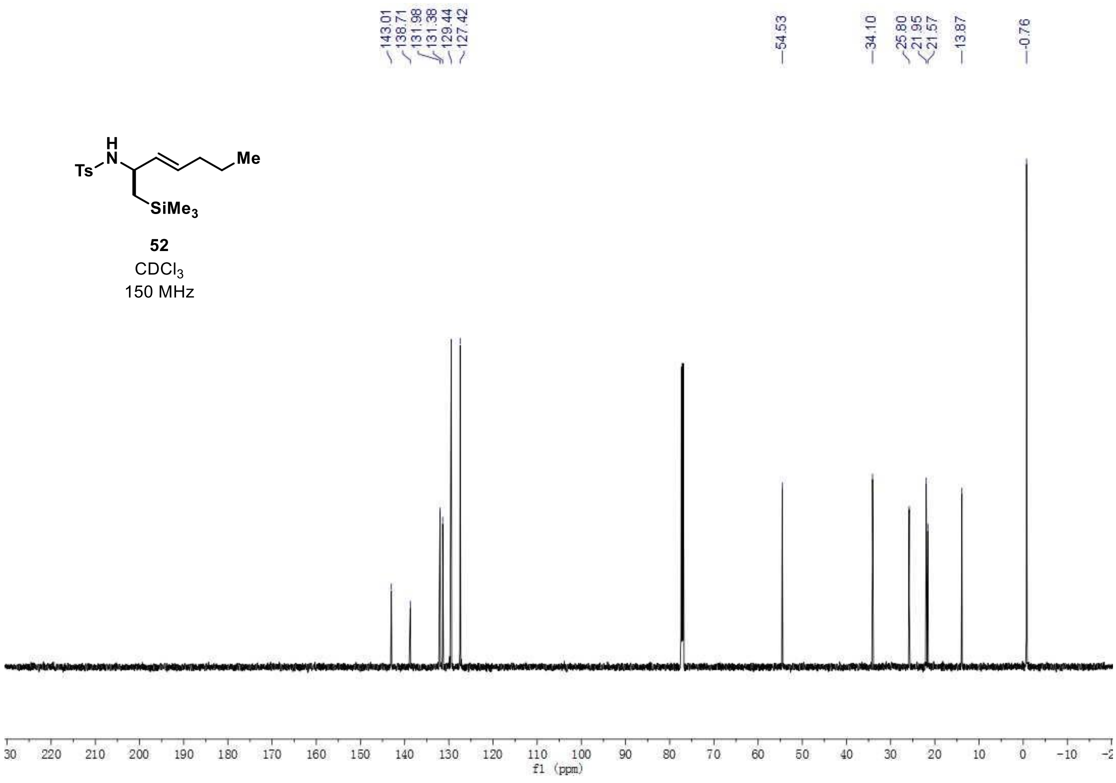

Supplementary Figure 155. ^{13}C NMR of compound 52. The sample has been recorded in 150 MHz, $CDCl_3$ at 25 °C.

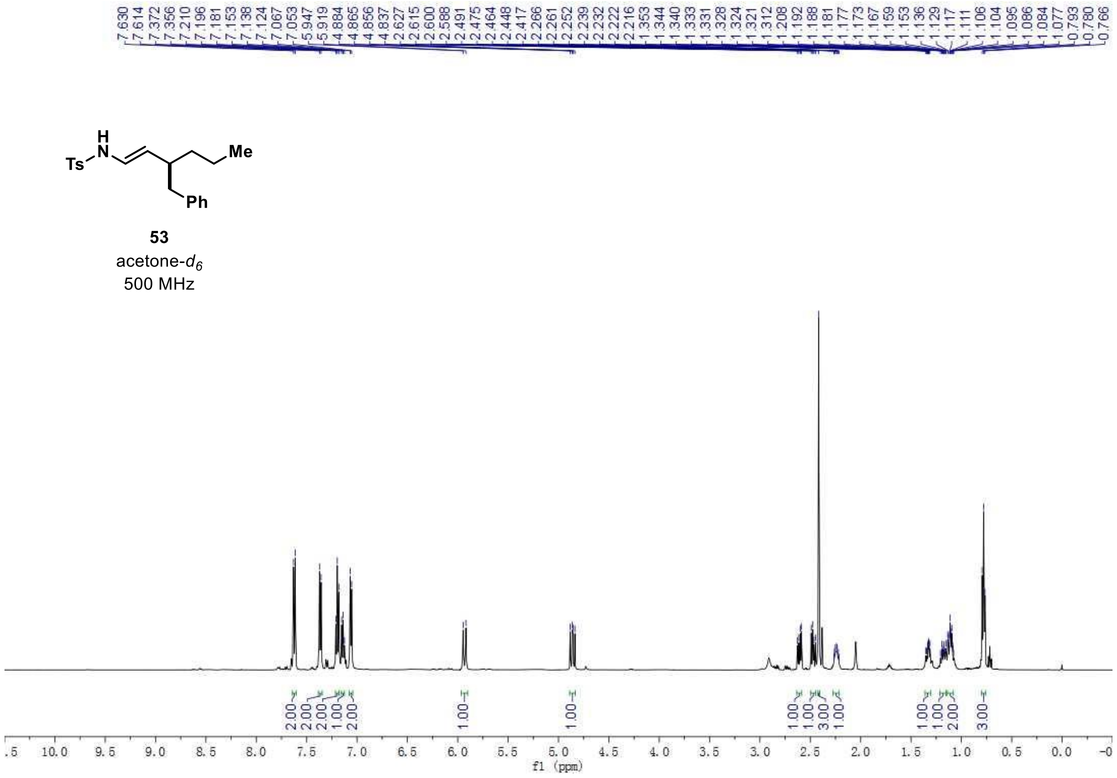

Supplementary Figure 156. ^{1}H NMR of compound 53. The sample has been recorded in 500 MHz, acetone-d_6 at 25 °C.

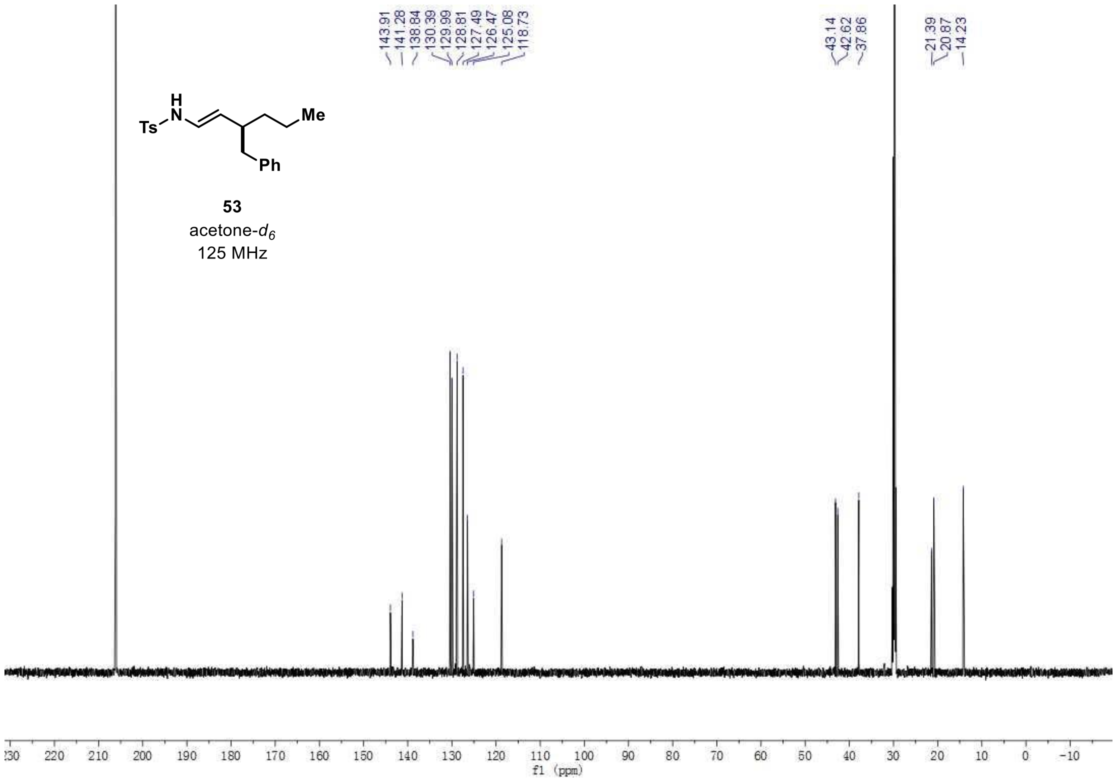

Supplementary Figure 157. ^{13}C NMR of compound 53. The sample has been recorded in 125 MHz, acetone-d_6 at 25 °C.

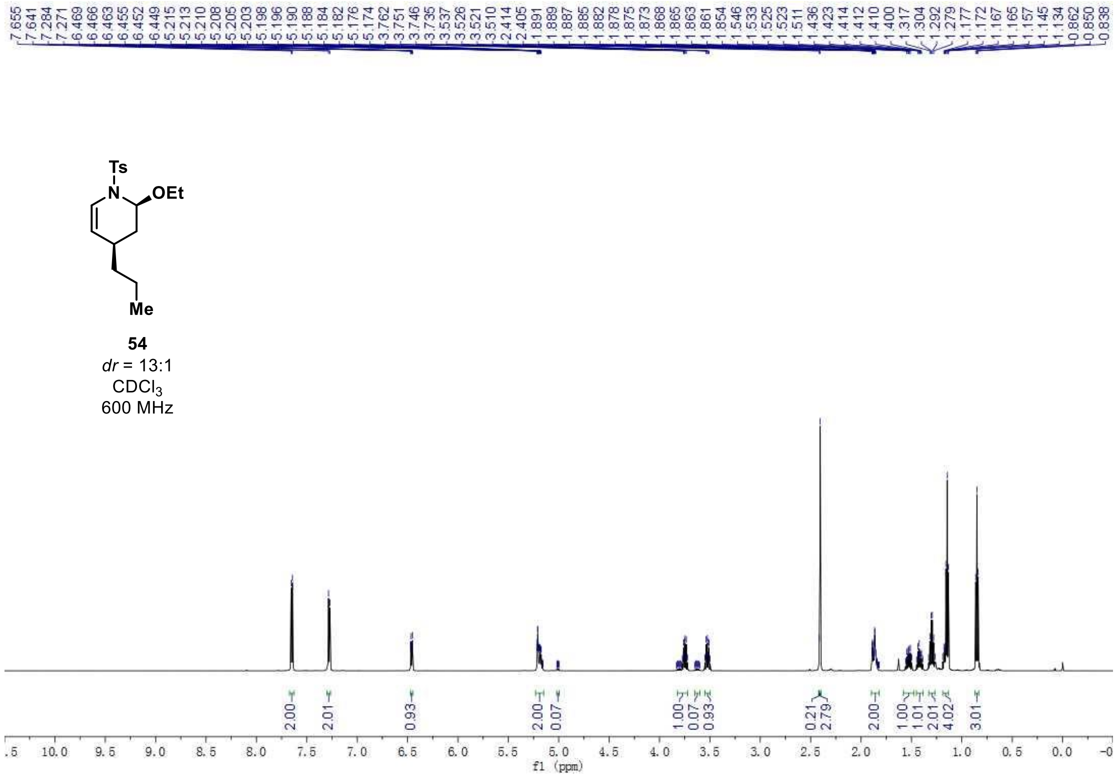

Supplementary Figure 158. ^{1}H NMR of compound 54. The sample has been recorded in 600 MHz, $CDCl_3$ at 25 °C.

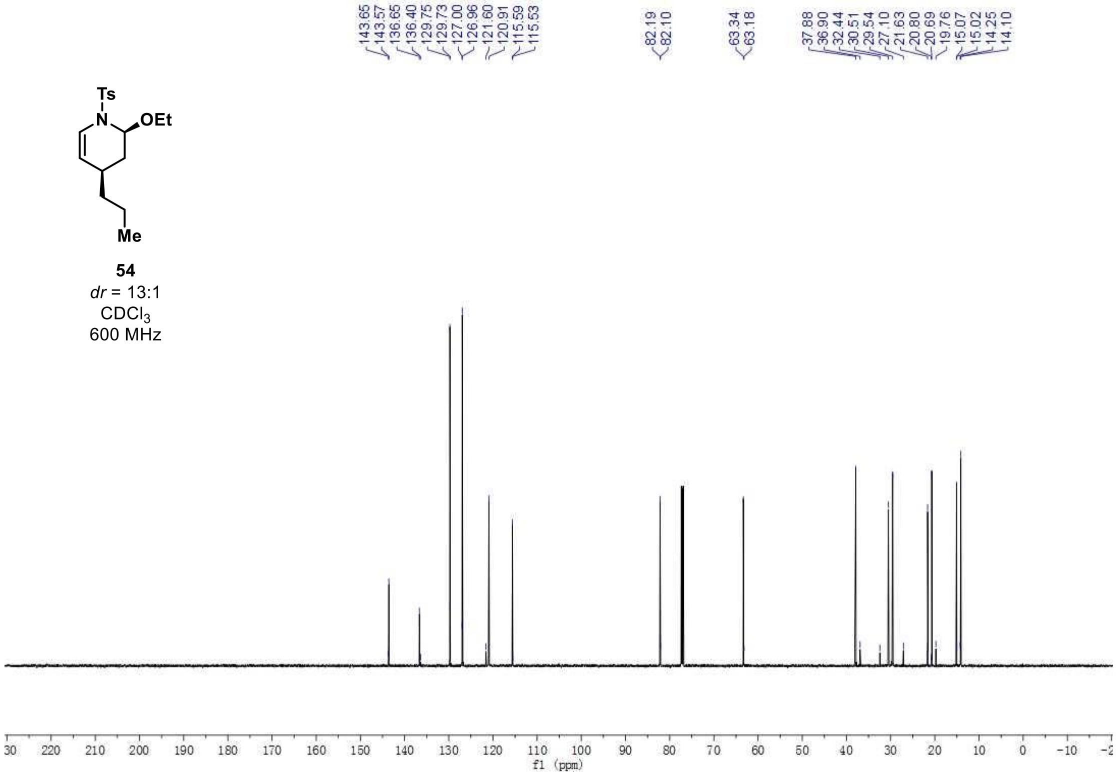

Supplementary Figure 159. ^{13}C NMR of compound 54. The sample has been recorded in 150 MHz, $CDCl_3$ at 25 °C.

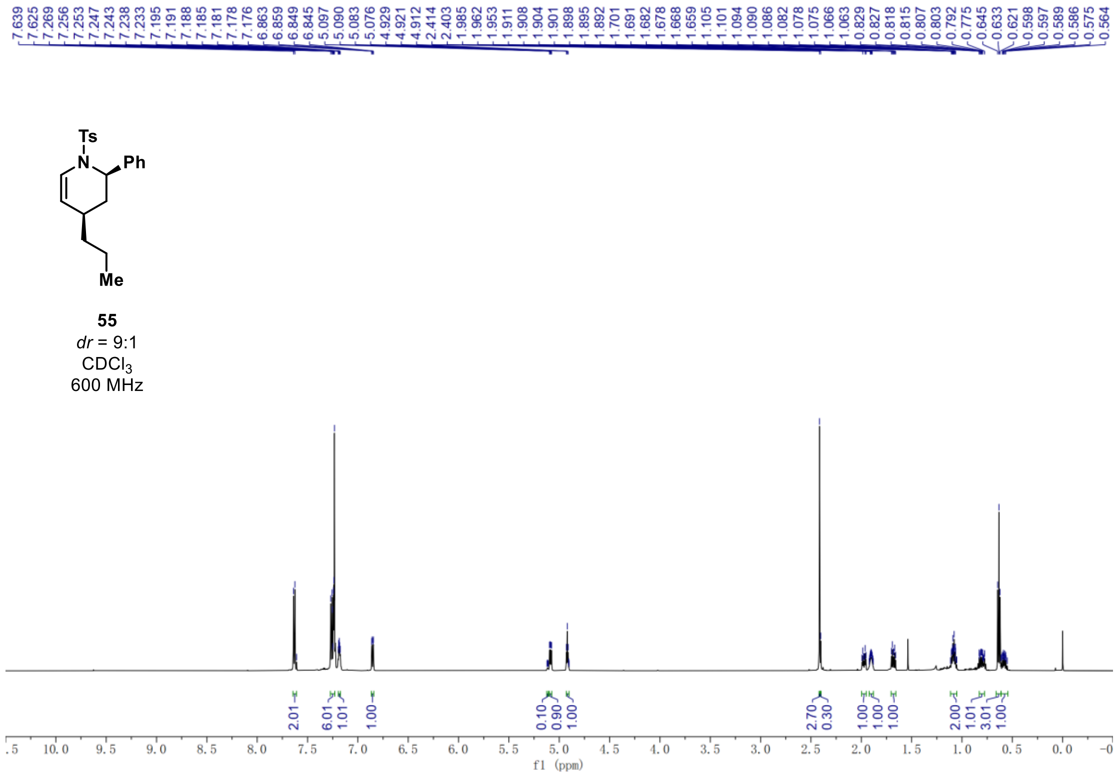

Supplementary Figure 160. ^{1}H NMR of compound 55. The sample has been recorded in 600 MHz, $CDCl_3$ at 25 °C.

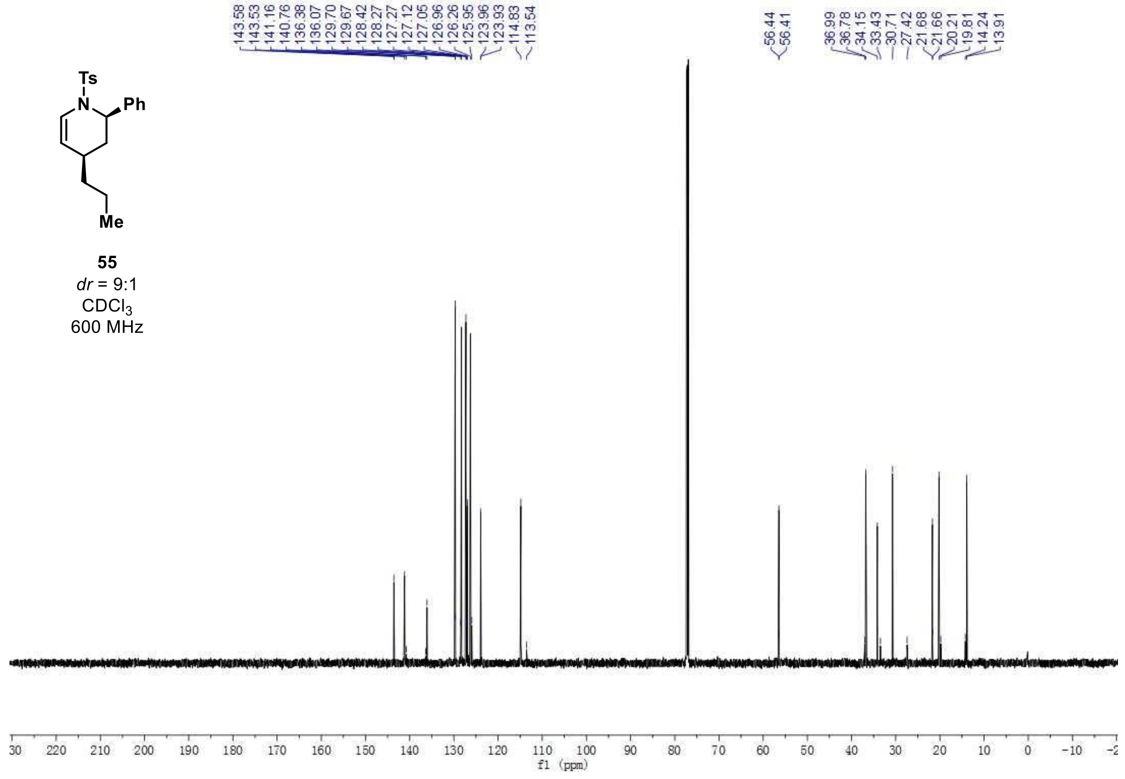

Supplementary Figure 161. ^{13}C NMR of compound 55. The sample has been recorded in 150 MHz, $CDCl_3$ at 25 °C.

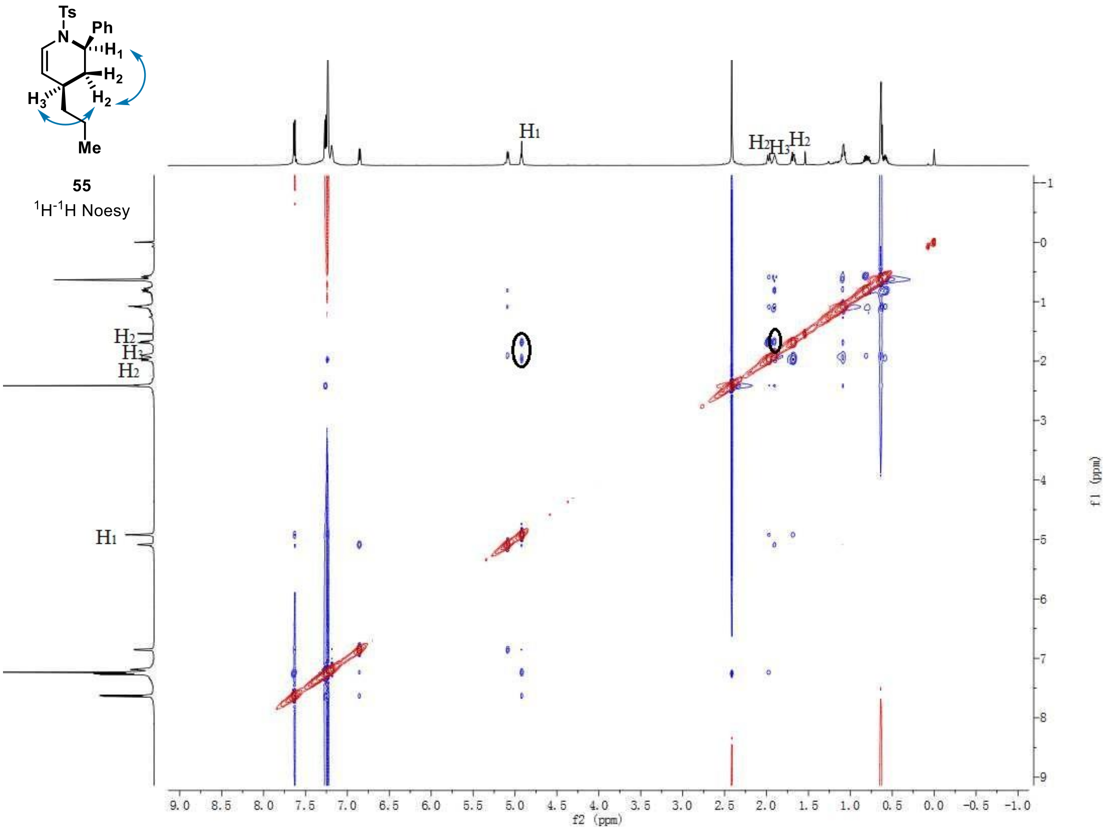

Supplementary Figure 162. ^{1}H-^{1}H Noesy of compound 55. The sample has been recorded in 600 MHz, $CDCl_3$ at 25 °C.

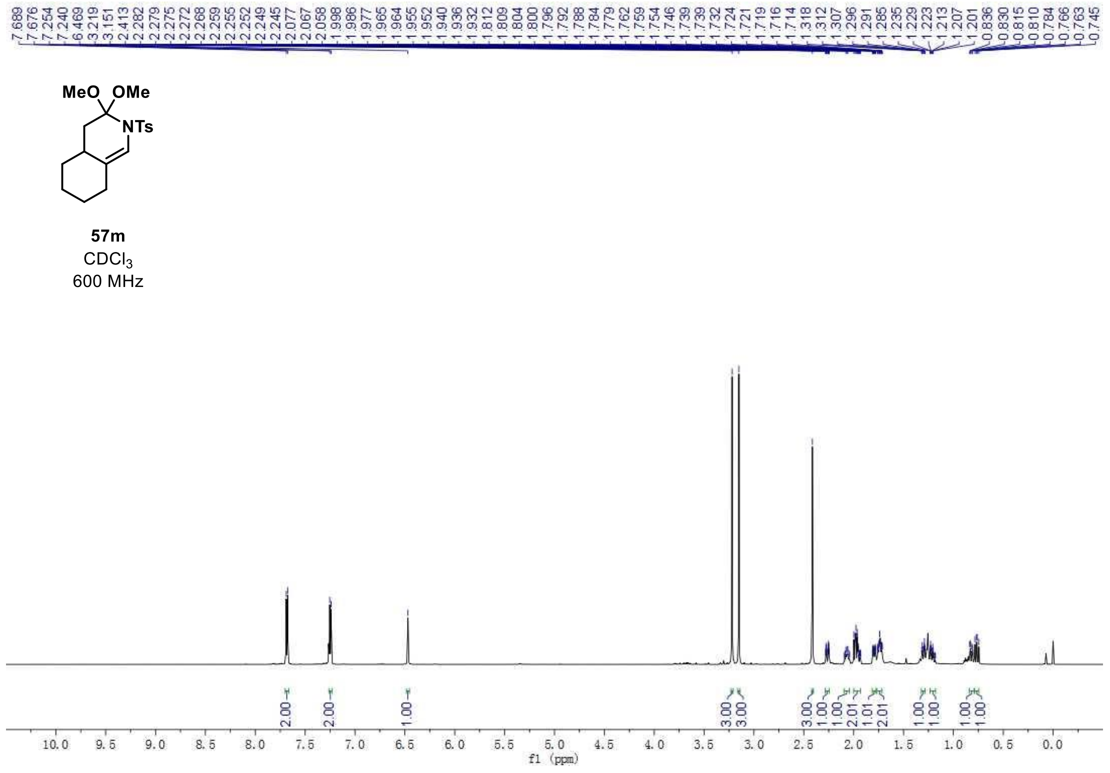

Supplementary Figure 163. ^{1}H NMR of compound 57m. The sample has been recorded in 600 MHz, $CDCl_3$ at 25 °C.

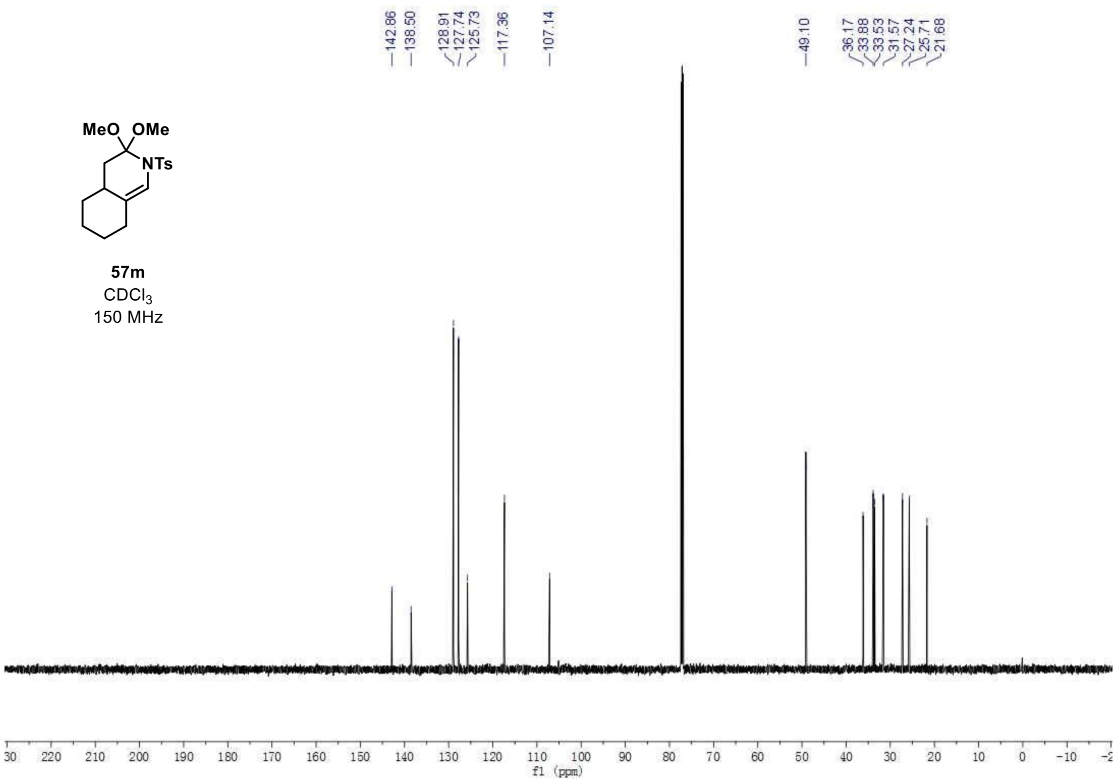

Supplementary Figure 164. ^{13}C NMR of compound 57m. The sample has been recorded in 150 MHz, $CDCl_3$ at 25 °C.

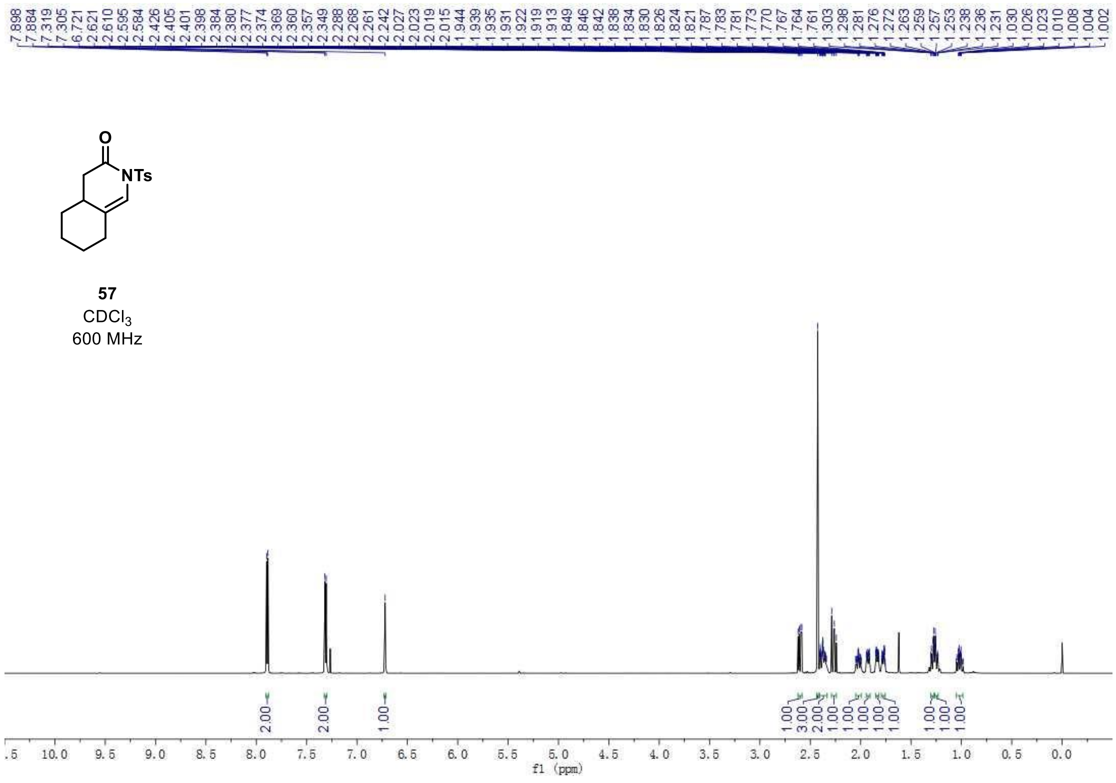

Supplementary Figure 165. ^{1}H NMR of compound 57. The sample has been recorded in 600 MHz, $CDCl_3$ at 25 °C.

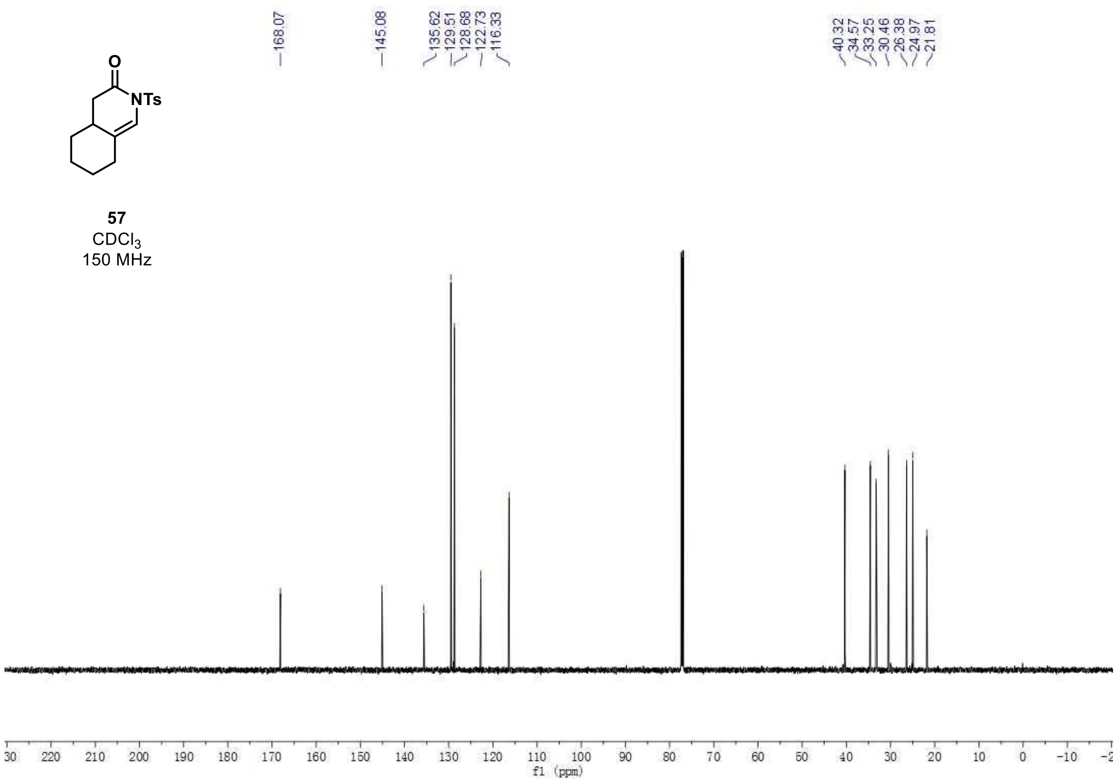

Supplementary Figure 166. ^{13}C NMR of compound 57. The sample has been recorded in 150 MHz, $CDCl_3$ at 25 °C.

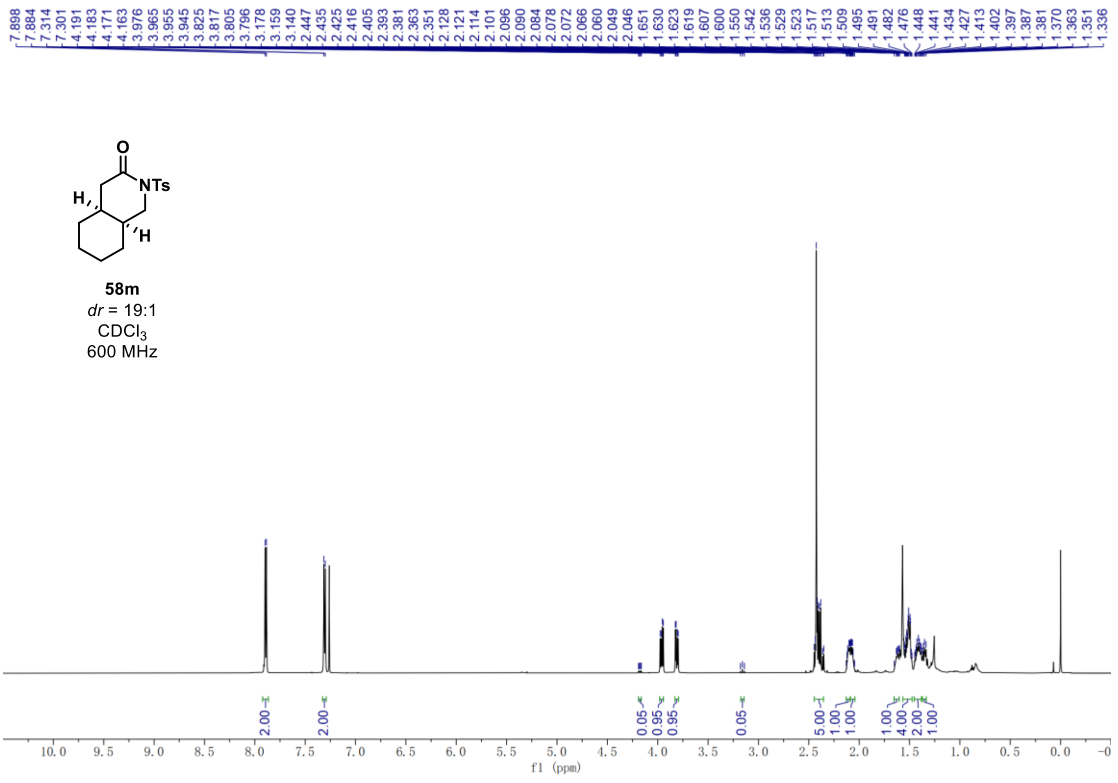

Supplementary Figure 167. ^{1}H NMR of compound 58m. The sample has been recorded in 600 MHz, $CDCl_3$ at 25 °C.

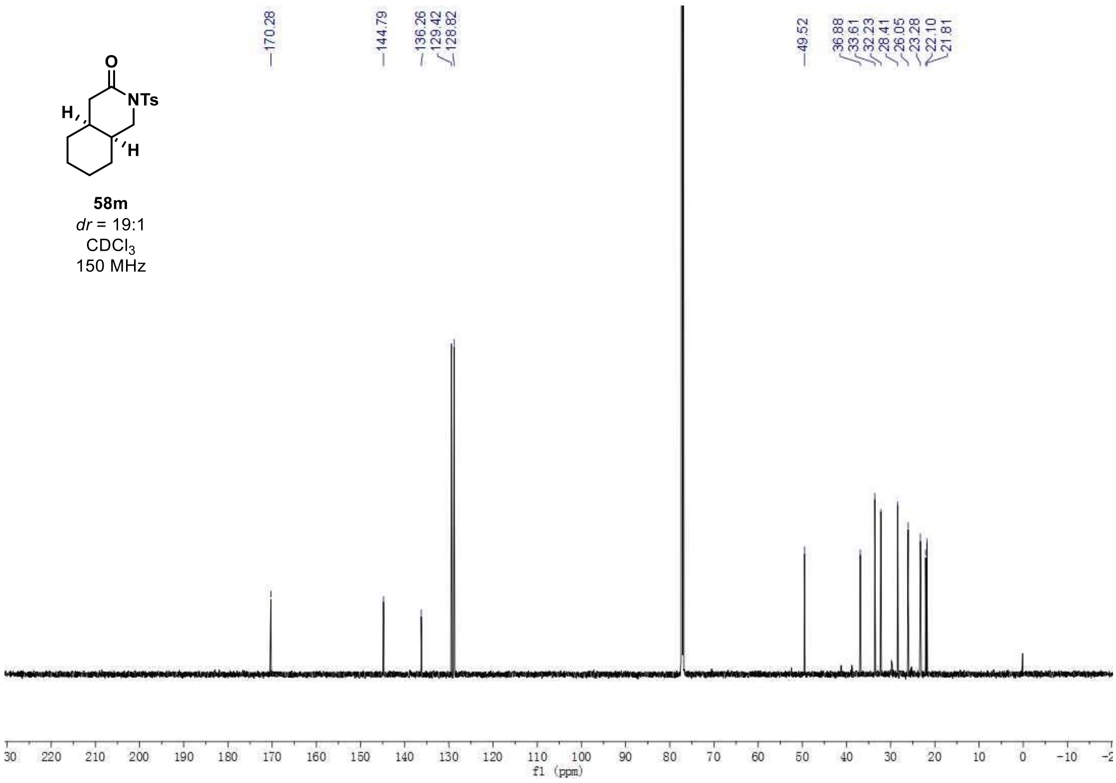

Supplementary Figure 168. ^{13}C NMR of compound 58m. The sample has been recorded in 150 MHz, $CDCl_3$ at 25 °C.

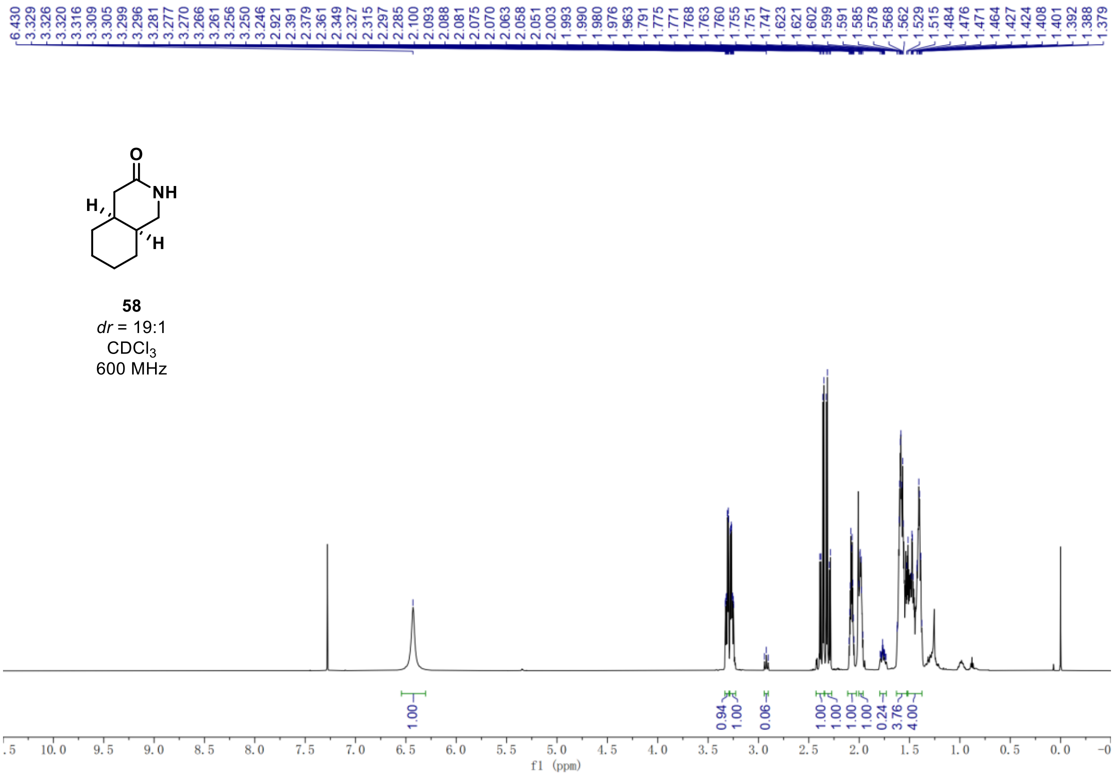

Supplementary Figure 169. ^{1}H NMR of compound 58. The sample has been recorded in 600 MHz, $CDCl_3$ at 25 °C.

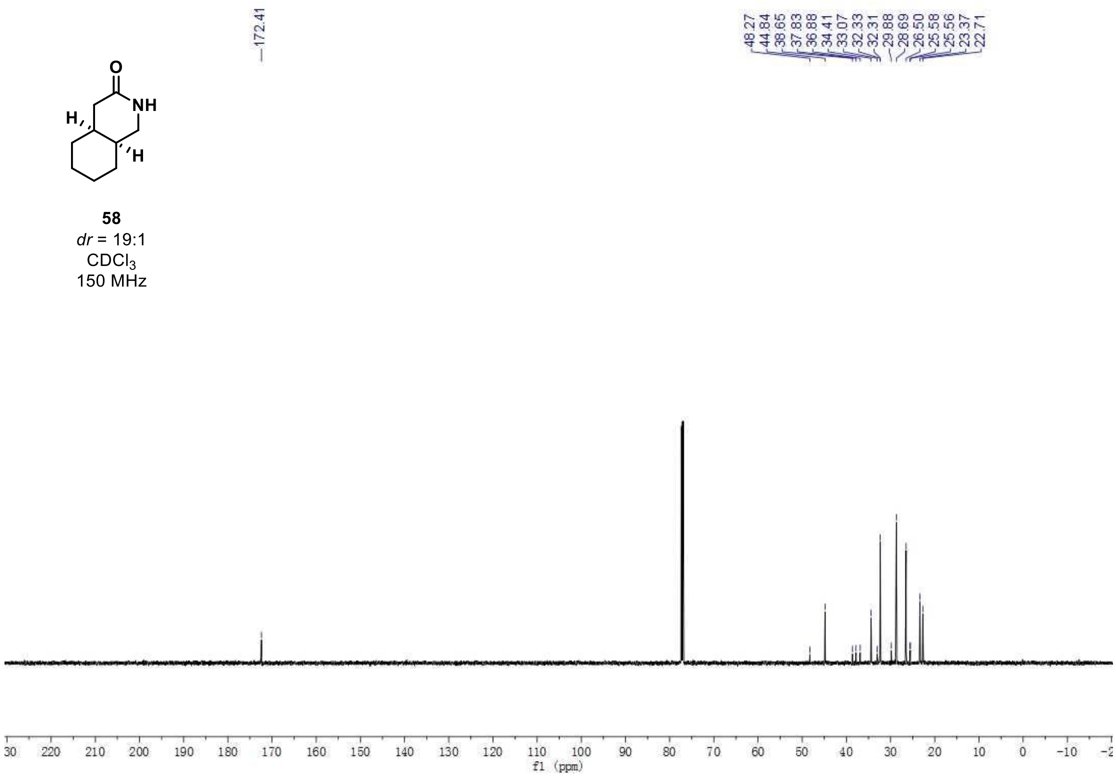

Supplementary Figure 170. ^{13}C NMR of compound 58. The sample has been recorded in 150 MHz, $CDCl_3$ at 25 °C.

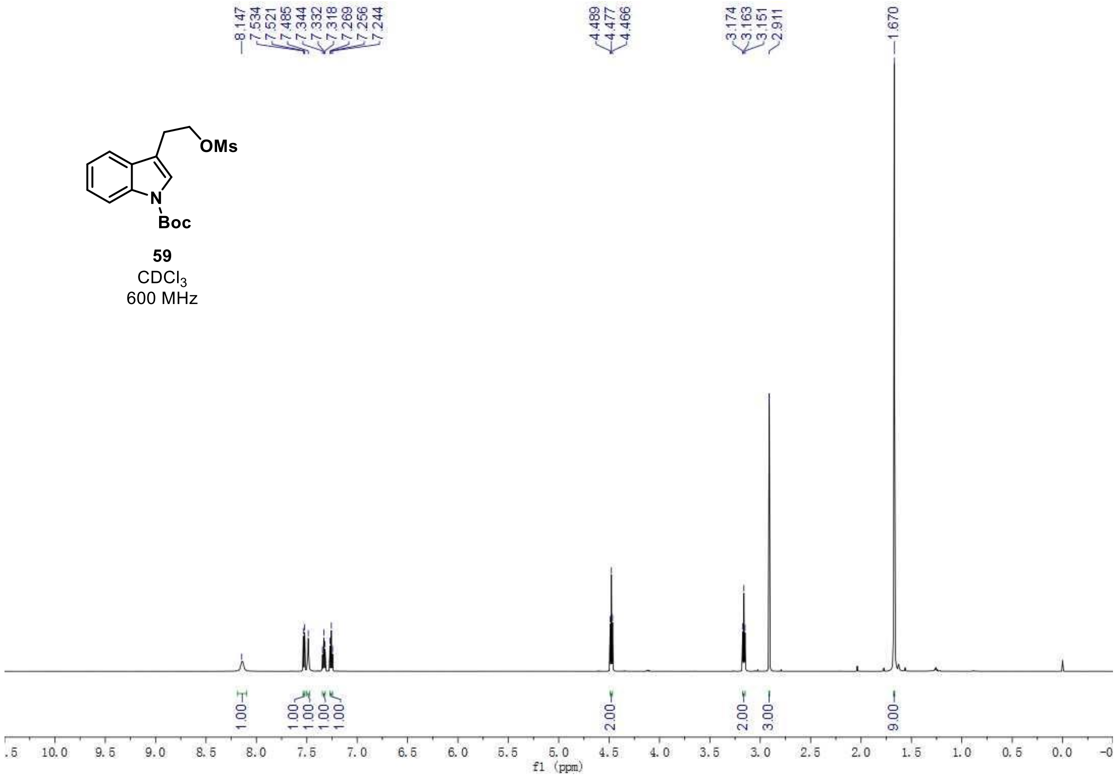

Supplementary Figure 171. ^{1}H NMR of compound 59. The sample has been recorded in 600 MHz, $CDCl_3$ at 25 °C.

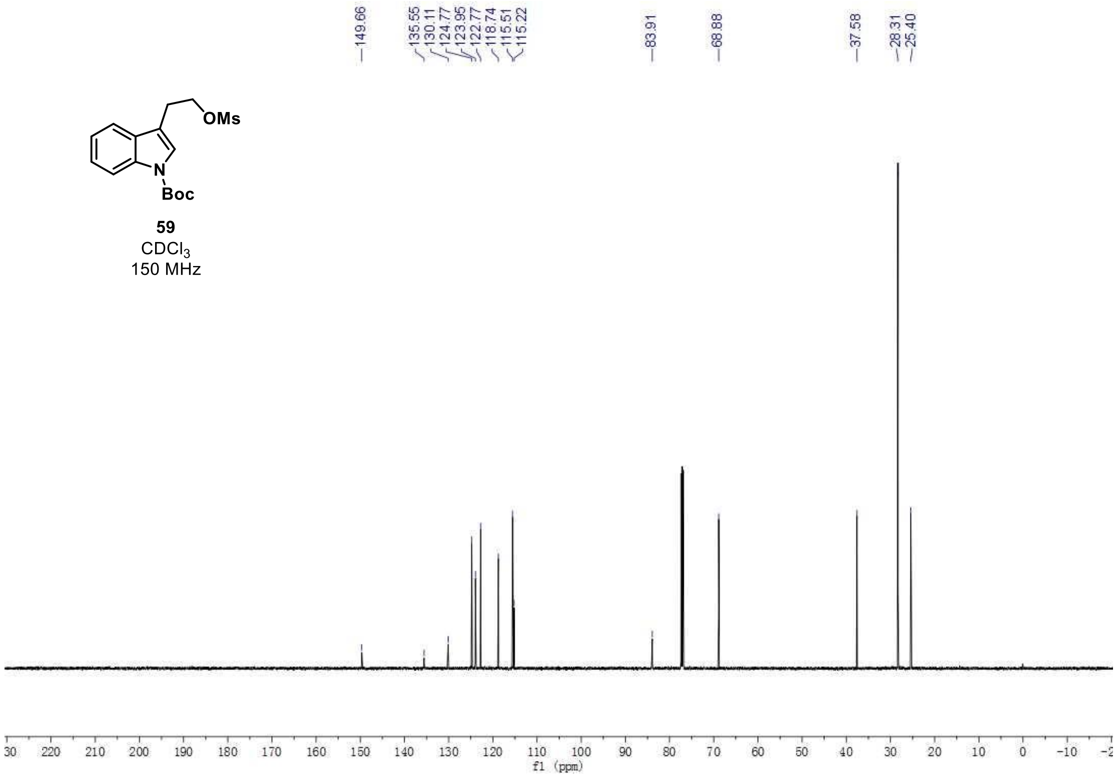

Supplementary Figure 172. ^{13}C NMR of compound 59. The sample has been recorded in 150 MHz, $CDCl_3$ at 25 °C.

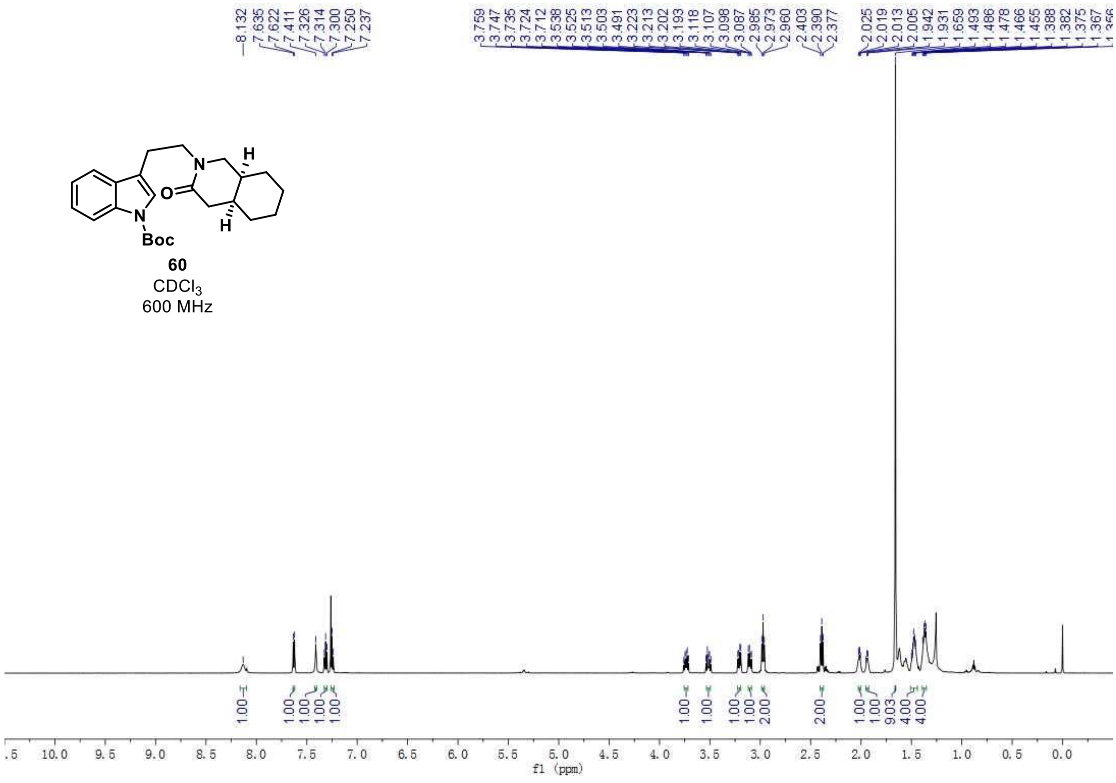

Supplementary Figure 173. ^{1}H NMR of compound 60. The sample has been recorded in 600 MHz, $CDCl_3$ at 25 °C.

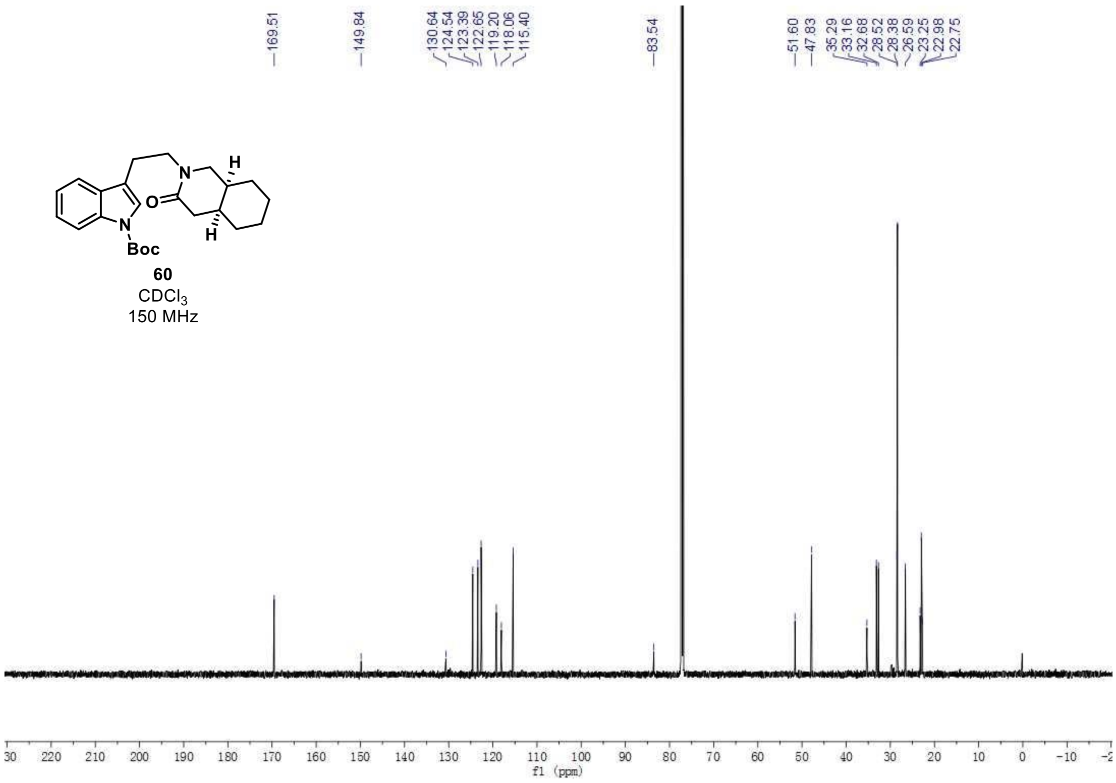

Supplementary Figure 174. ^{13}C NMR of compound 60. The sample has been recorded in 150 MHz, $CDCl_3$ at 25 °C.

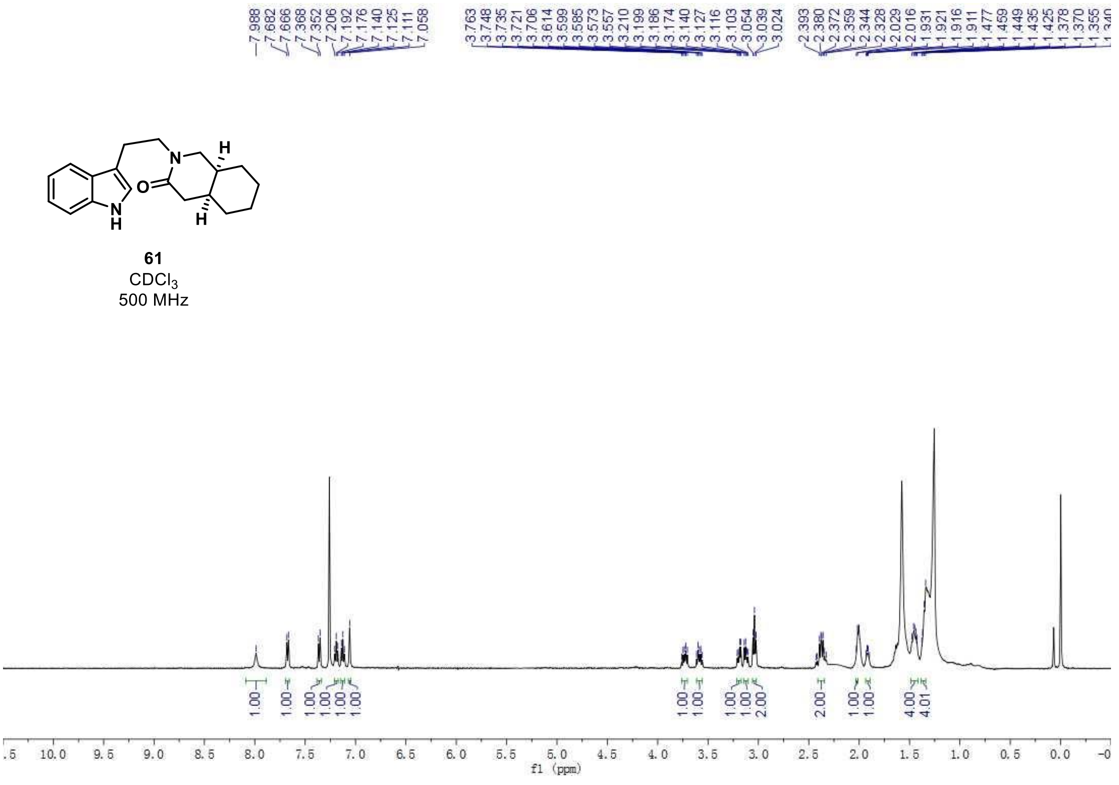

Supplementary Figure 175. ^{1}H NMR of compound 61. The sample has been recorded in 500 MHz, $CDCl_3$ at 25 °C.

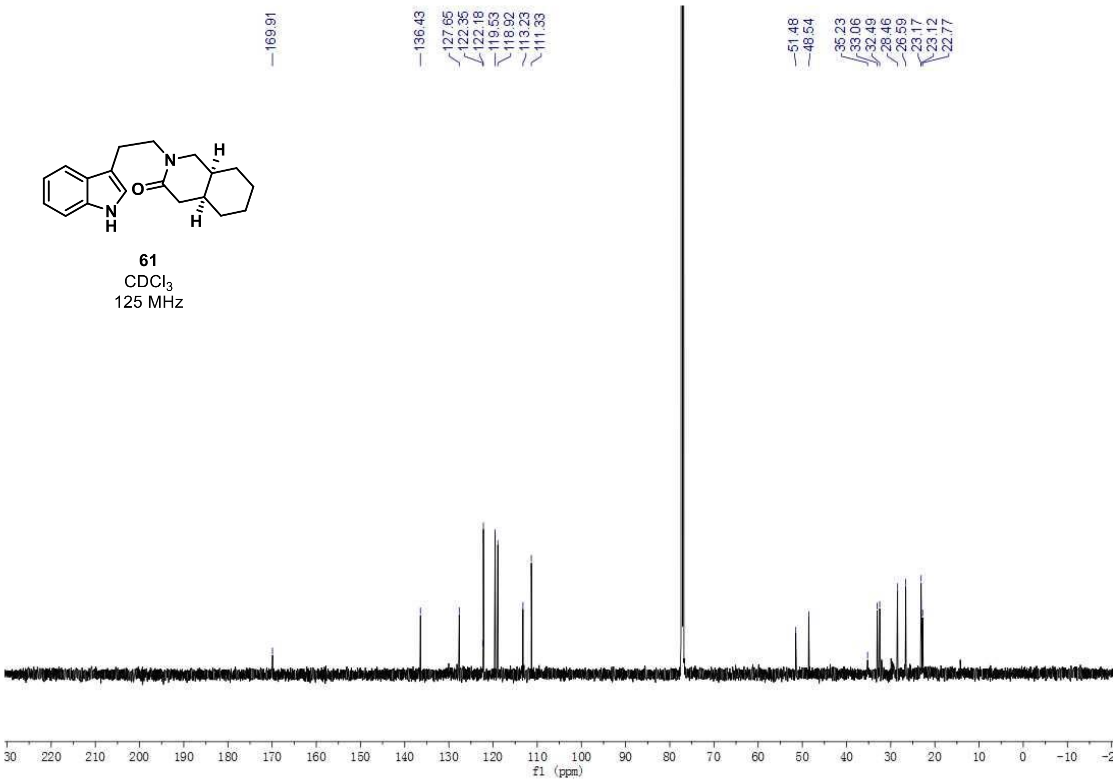

Supplementary Figure 176. ^{13}C NMR of compound 61. The sample has been recorded in 125 MHz, $CDCl_3$ at 25 °C.

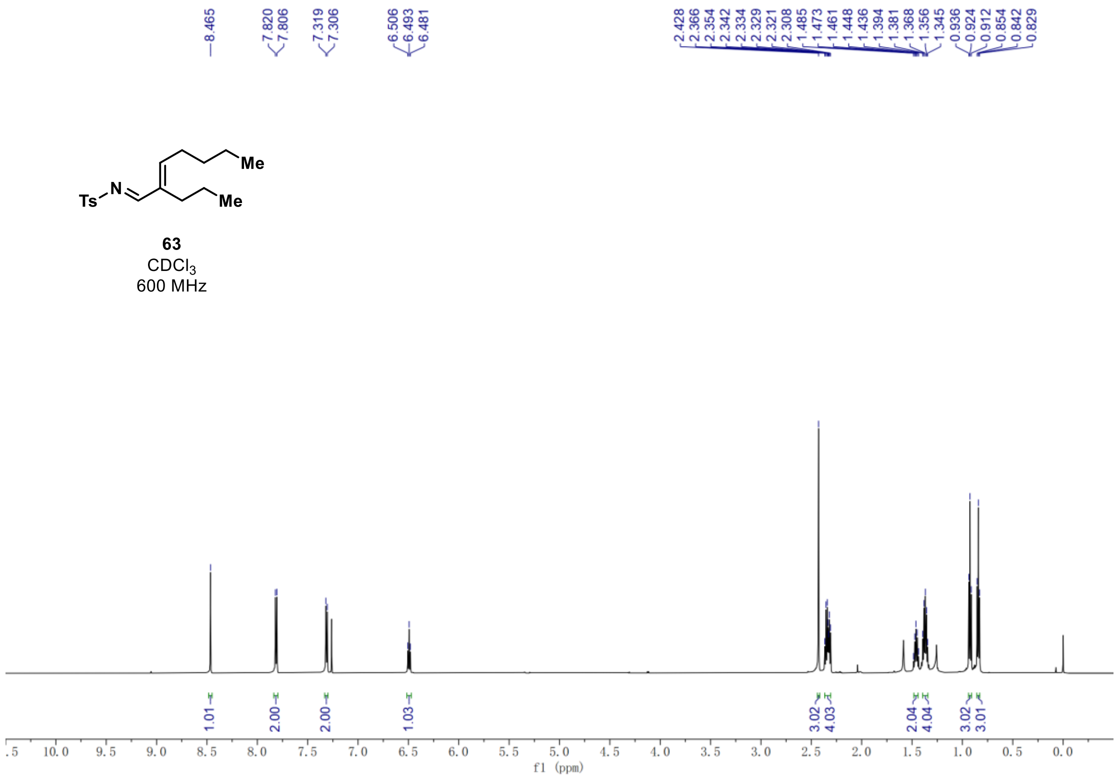

Supplementary Figure 177. ^{1}H NMR of compound 63. The sample has been recorded in 600 MHz, $CDCl_3$ at 25 °C.

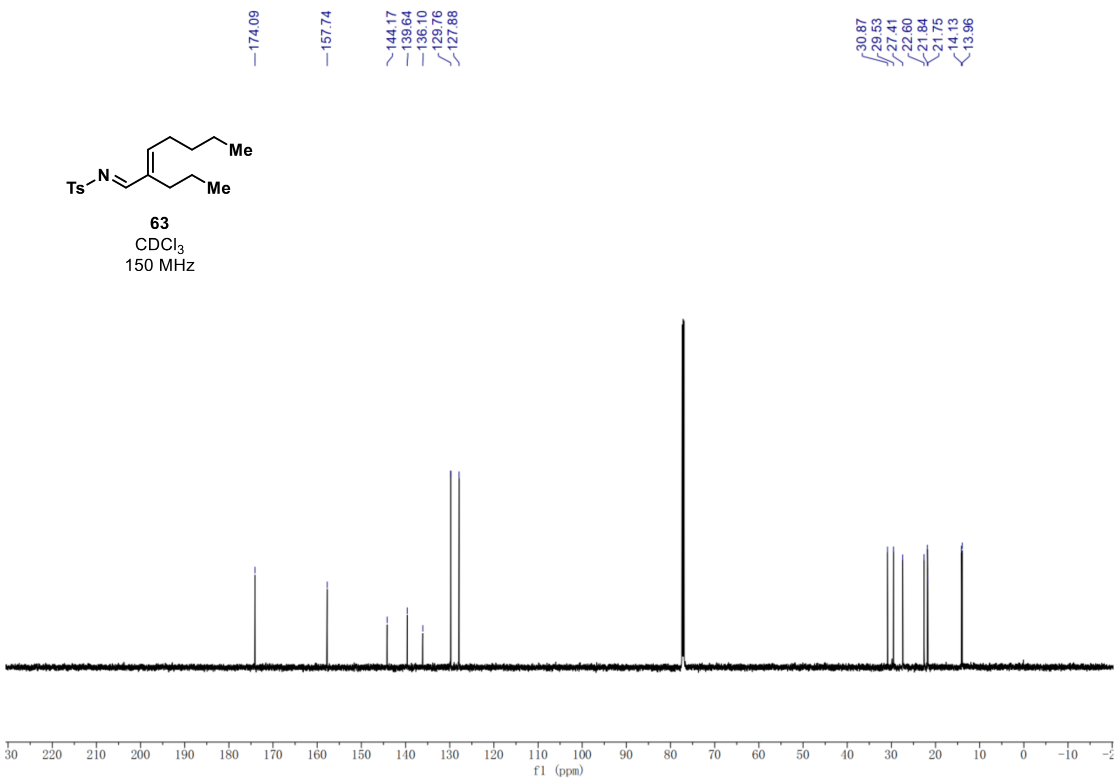

Supplementary Figure 178. ^{13}C NMR of compound 63. The sample has been recorded in 150 MHz, $CDCl_3$ at 25 °C.

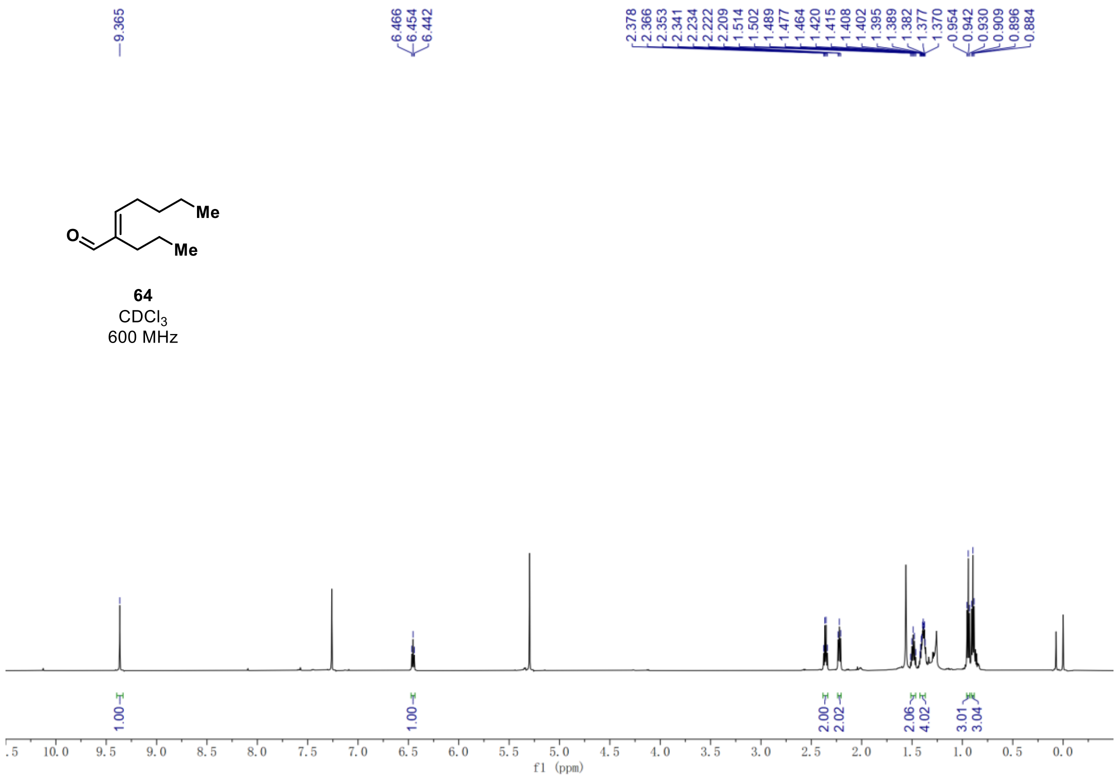

Supplementary Figure 179. ^{1}H NMR of compound 64. The sample has been recorded in 600 MHz, $CDCl_3$ at 25 °C.

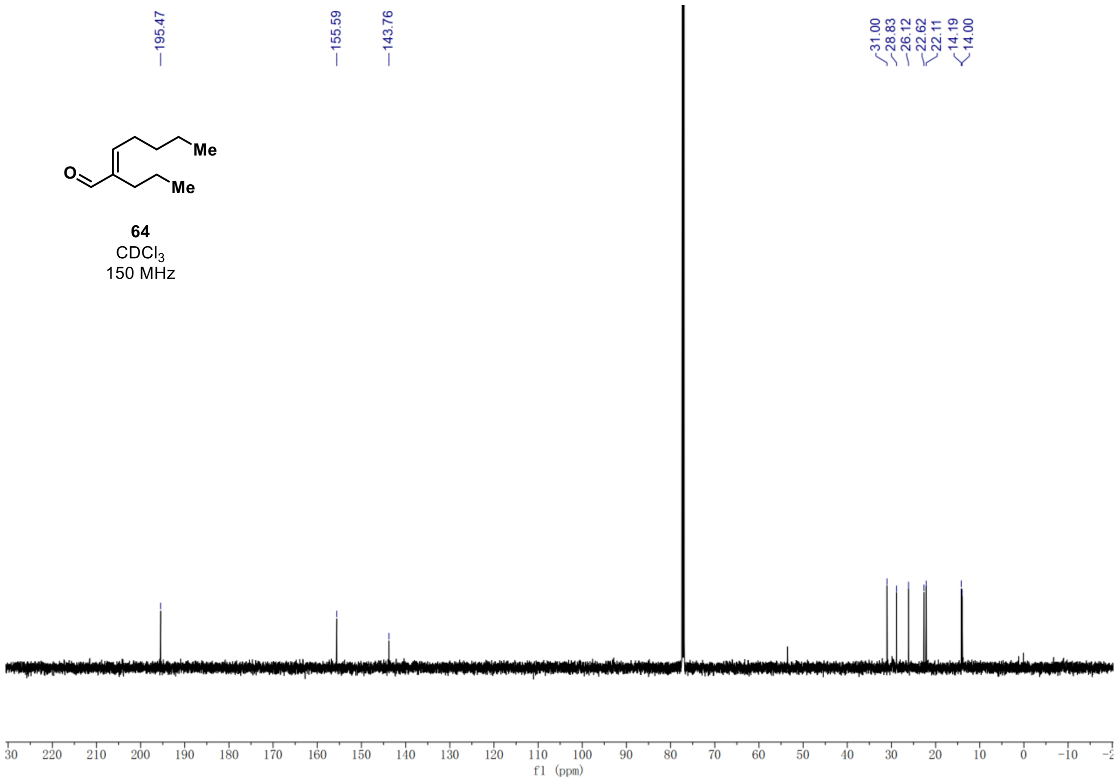

Supplementary Figure 180. ^{13}C NMR of compound 64. The sample has been recorded in 150 MHz, $CDCl_3$ at 25 °C.

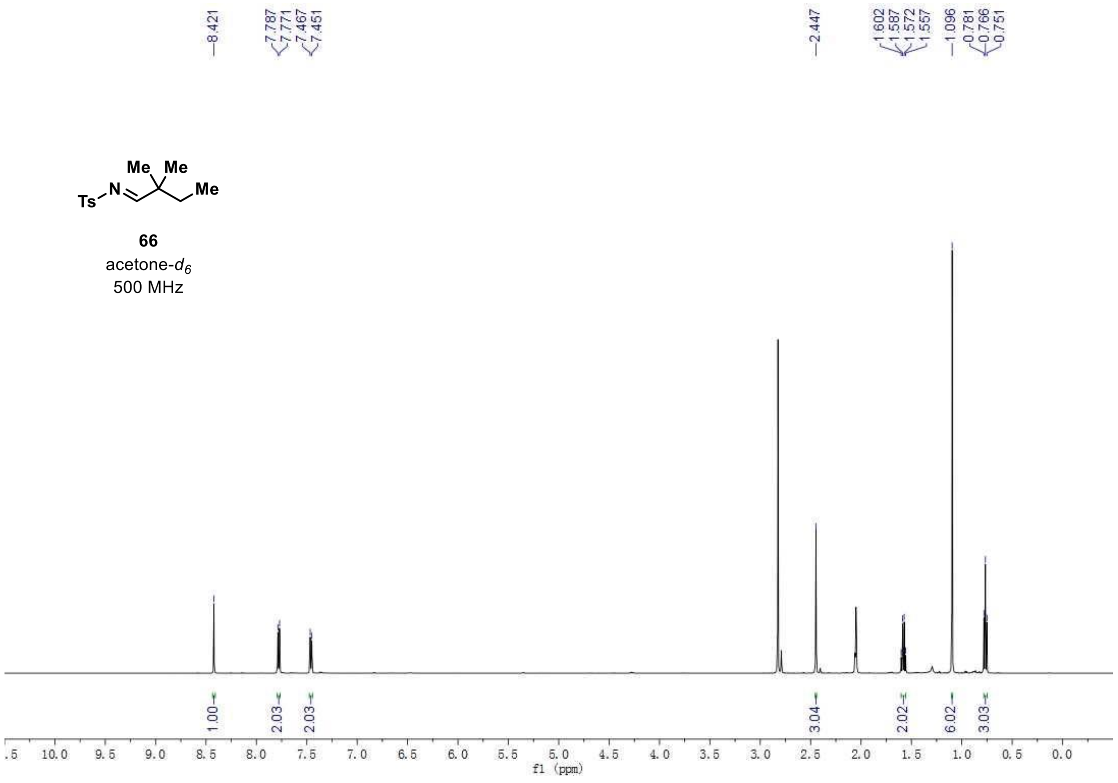

Supplementary Figure 181. ^{1}H NMR of compound 66. The sample has been recorded in 500 MHz, acetone-d_6 at 25 °C.

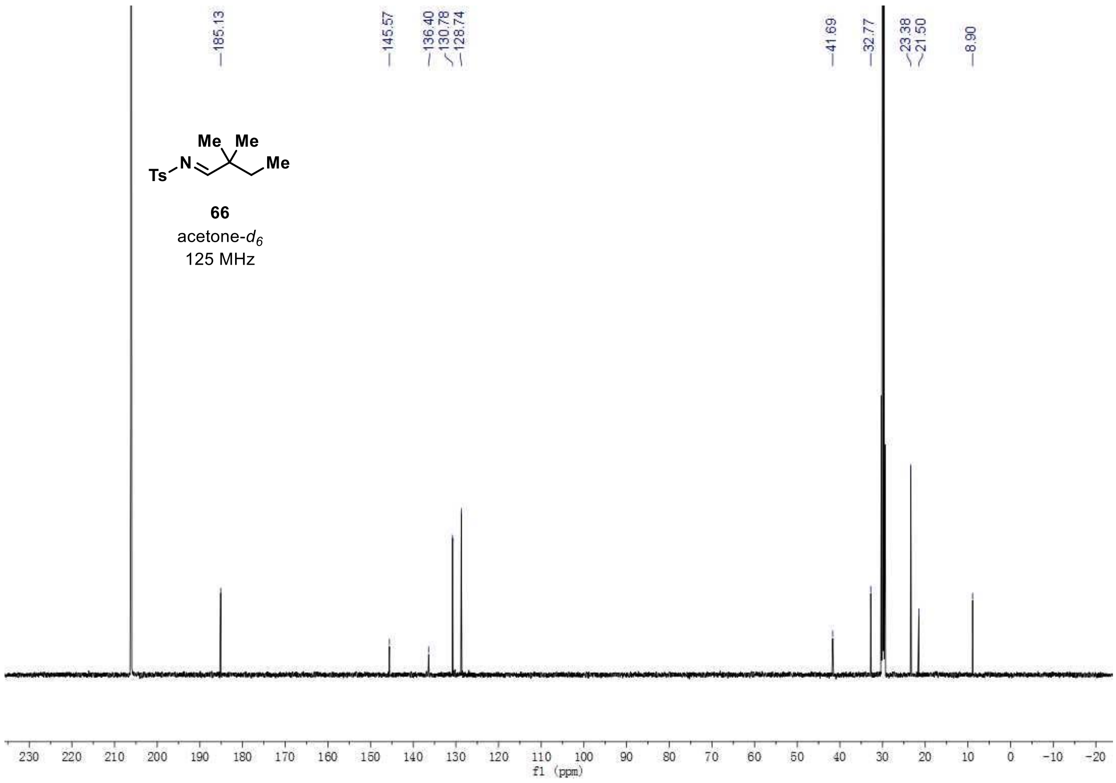

Supplementary Figure 182. ^{13}C NMR of compound 66. The sample has been recorded in 125 MHz, acetone-d_6 at 25 °C.

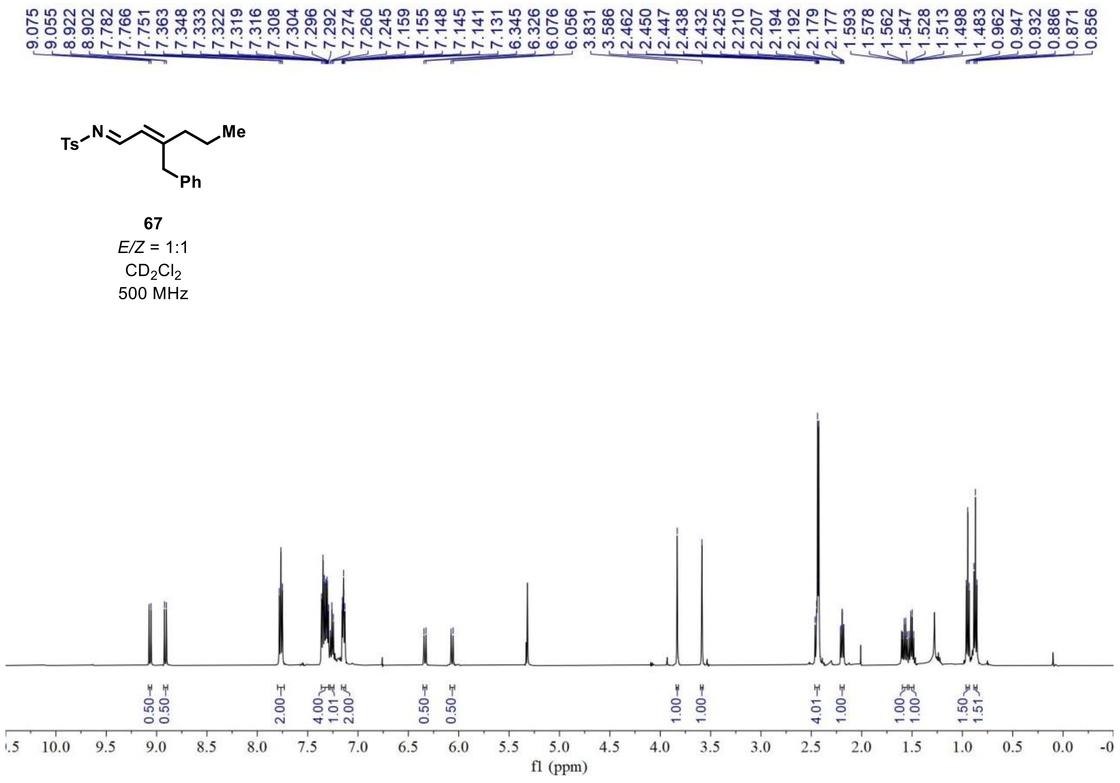

Supplementary Figure 183. ^{1}H NMR of compound 67. The sample has been recorded in 500 MHz, CD_2Cl_2 at 25 °C.

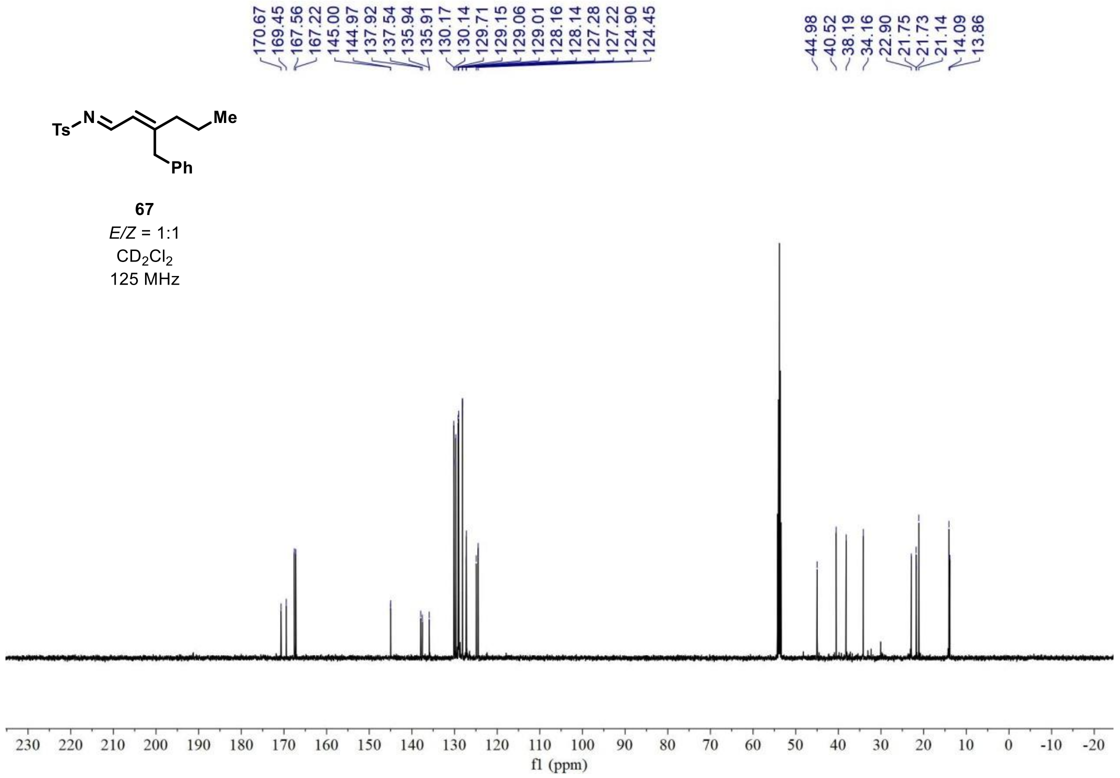

Supplementary Figure 184. ^{13}C NMR of compound 67. The sample has been recorded in 125 MHz, CD_2Cl_2 at 25 °C.

Part 4. Supplementary references

[1] Frisch, M. J. et al. Gaussian 16, Revision C.01, Gaussian, Inc., Wallingford CT (2019).

[2] Fukui, K. A Formulation of the reaction coordinate. *J. Phys. Chem.* **74**, 4161-4163 (1970).

[3] Mammen, M., Shakhnovich, E. I., Deutch, J. M. & Whitesides, G. M. Estimating the entropic cost of self-assembly of multiparticle hydrogen-bonded aggregates based on the cyanuric acid·melamine lattice. *J. Org. Chem.* **63**, 3821-3830 (1998).

[4] Sakaki, S., Ohnishi, Y. Y. & Sato, H. Theoretical and computational studies of organometallic reactions: successful or not? *Chem. Rec.* **10**, 29-45 (2010).

[5] Ishikawa, A., Nakao, Y., Sato, H. & Sakaki, S. Oxygen atom transfer reactions of iridium and osmium complexes: theoretical study of characteristic features and significantly large differences between these two complexes. *lnorg. Chem.* **48**, 8154-8163 (2009).

[6] Ishikawa, A., Nakao, Y., Sato, H. & Sakaki, S. Pd(II)-promoted direct cross-coupling reaction of arenes via highly regioselective aromatic C-H activation: a theoretical study. *Dalton Trans.* **39**, 3279-3289 (2010).

[7] Zhao, C. et al. Divergent regioselective Heck-type reaction of unactivated alkenes and N-fluoro-sulfonamides. *Nat. Commun.* **13**, 6297 (2022).

[8] Zhang, H. et al. Remote directed isocyanation of unactivated C(sp^3)–H bonds: forging seven-membered cyclic ureas enabled by copper catalysis. *Org. Lett.* **22**, 997-1002 (2020).

[9] Zhang, H., Zhou, Y., Tian, P. & Jiang, C. Copper-catalyzed amide radical-directed cyanation of unactivated $C_{sp}{}^3$–H bonds. *Org. Lett.* **21**, 1921-1925 (2019).

[10] Hu, J. et al. Metal-free C(sp^3)–H functionalization of sulfonamides via strain-release rearrangement. *Chem. Sci.* **12**, 4034-4040 (2021).

[11] Qin, Y., Han, Y., Tang, Y., Wei, J. & Yang, M. A general method for site-selective Csp^3–S bond formation via cooperative catalysis. *Chem. Sci.* **11**, 1276-1282 (2020).

[12] Modak, A., Pinter, E. N. & Cook, S. P. Copper-catalyzed, N-directed Csp^3–H trifluoromethylthiolation (−SCF_3) and trifluoromethylselenation (−$SeCF_3$). *J. Am. Chem. Soc.* **141**, 18405-18410 (2019).

[13] Shi, S., Yang, X., Tang, M., Hu, J. & Loh, T.-P. Direct synthesis of α-amino nitriles from sulfonamides via base-mediated C–H cyanation. *Org. Lett.* **23**, 4018-4022 (2021).

[14] Ji, Y.-X., Li, J., Li, C.-M., Qu, S. & Zhang, B. Manganese-catalyzed N–F bond activation for hydroamination and carboamination of alkenes. *Org. Lett.* **23**, 207-212 (2021).

[15] Zhang, H. et al. Iron-catalyzed, site-selective difluoromethylthiolation (−SCF_2H) and difluoromethylselenation (−$SeCF_2H$) of unactivated C(sp^3)–H bonds in N-fluoroamides. *Org. Lett.* **23**, 4721-4725 (2021).

[16] Khrizman, A., Cheng, H. Y. & Moyna, G. Synthesis of sequentially deuterated 1-n-Butyl-3-methylimidazolium ionic liquids. *J. Label Compd. Radiopharm*, **54**, 401-407 (2011).

[17] Trenkle, J. D. & Jamison, T. F. Macrocyclization by nickel-catalyzed, ester-promoted, epoxide–alkyne reductive coupling: total synthesis of (−)-gloeosporone. *Angew. Chem. Int. Ed.* **48**, 5366-5368 (2009).

[18] Shimojo, M., Matsumoto, K. & Hatanaka, M. Enzyme-mediated preparation of optically active 1,2-diols bearing a long chain: enantioselective hydrolysis of cyclic carbonates. *Tetrahedron* **56**, 9281-9288 (2000).

[19] Babu, B. P., Endo, Y. & Bäckvall, J.-E. Biomimetic aerobic oxidation of amino alcohols to lactams. *Chem. Eur. J.* **18**, 11524-11527 (2012).

[20] Green, M. P., Prodger, J. C., Sherlock, A. E. & Hayes, C. J. A convenient method for 3-pyrroline synthesis. *Org. Lett.* **3**, 3377-3379 (2001).

[21] Xu, B. & Tambar, U. K. Remote allylation of unactivated C(sp^3)–H bonds triggered by photogenerated amidyl radicals. *ACS Catal.* **9**, 4627-4631 (2019).

[22] Yang, F., Saiki, Y., Nakaoka, K. & Ema, T. One-pot synthesis of aldehydes or alcohols from CO_2 via formamides or silyl formates. *Adv. Synth. Catal.* **365**, 877-883 (2023).

[23] Chernykh, A. V. et al. Last of the gem-difluorocycloalkanes: synthesis and characterization of 2,2-difluorocyclobutyl-substituted building blocks. *J. Org. Chem.* **84**, 8487-8496 (2019).

[24] Ghosh, N., Nayak, S. & Sahoo, A. K. Gold-catalyzed regioselective hydration of propargyl acetates assisted by a neighboring carbonyl group: access to α-acyloxy methyl ketones and synthesis of (±)-actinopolymorphol B. *J. Org. Chem.* **76**, 500-511 (2011).

[25] Chen, H.-W., Hsu, R.-T., Chang, M.-Y. & Chang, N.-C. Efficient synthesis of fused bicyclic glutarimides. Its application to (±)-alloyohimbane and louisianin D. *Org. Lett*. **8**, 3033-3035 (2006).

[26] Morales, S., Guijarro, F. G., Ruano, J. L. G. & Cid, M. B. A general aminocatalytic method for the synthesis of aldimines. *J. Am. Chem. Soc.* **136**, 1082-1089 (2014).
